# Photocatalytic Reductive Radical‐Polar Crossover for a Base‐Free Corey–Seebach Reaction

**DOI:** 10.1002/chem.202003000

**Published:** 2020-09-17

**Authors:** Karsten Donabauer, Kathiravan Murugesan, Urša Rozman, Stefano Crespi, Burkhard König

**Affiliations:** ^1^ Department of Organic Chemistry University of Regensburg Universitätsstraße 31 93053 Regensburg Germany; ^2^ Stratingh Institute for Chemistry University of Groningen Nijenborgh 4 9747 AG Groningen The Netherlands

**Keywords:** carbanion, Corey–Seebach, HAT-catalysis, photocatalysis, radical-polar crossover

## Abstract

A metal‐free generation of carbanion nucleophiles is of prime importance in organic synthesis. Herein we report a photocatalytic approach to the Corey–Seebach reaction. The presented method operates under mild redox‐neutral and base‐free conditions giving the desired product with high functional group tolerance. The reaction is enabled by the combination of photo‐ and hydrogen atom transfer (HAT) catalysis. This catalytic merger allows a C−H to carbanion activation by the abstraction of a hydrogen atom followed by radical reduction. The generated nucleophilic intermediate is then capable of adding to carbonyl electrophiles. The obtained dithiane can be easily converted to the valuable α‐hydroxy carbonyl in a subsequent step. The proposed reaction mechanism is supported by emission quenching, radical–radical homocoupling and deuterium labeling studies as well as by calculated redox‐potentials and bond strengths.

## Graphical abstract

**Picture perfect**: A photocatalytic approach for the Corey–Seebach reaction is presented. This base‐ and metal‐free reaction exploits the combination of photo‐ and HAT‐catalysis furnishing the desired product under mild conditions with a high functional group tolerance. The reactive intermediate is generated by hydrogen atom abstraction followed by radical reduction rendering a carbanion nucleophile capable of adding to aldehydes and ketones.

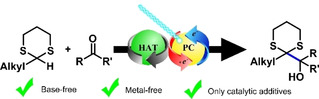

Ketones bearing a hydroxy group in alpha position are a common structural moiety in several natural products and potential drugs.^1^ A classical method to synthesize this class of compound is the well‐known Corey–Seebach umpolung first reported in 1965.^2^ In their seminal and subsequent work, they describe the deprotonation of dithianes by a strong base, yielding an acyl anion equivalent. This intermediate is capable of reacting with various electrophiles, including carbonyl compounds opening easy access to α‐hydroxy ketones upon deprotection (Scheme 1 A).^3^

**Scheme 1:**
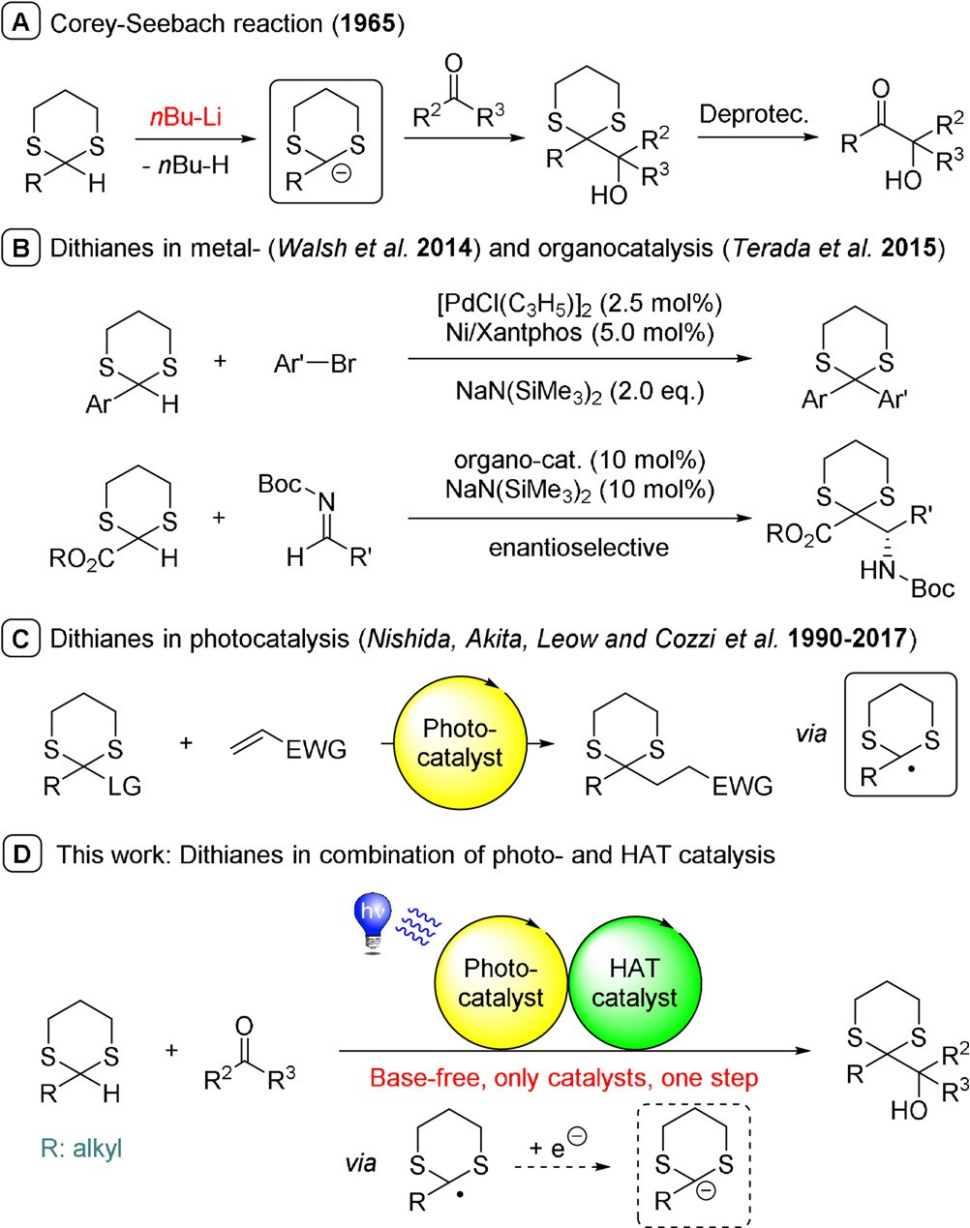
Corey–Seebach reaction and the use of dithianes as substrate in different catalytic systems.

A more novel approach to generate acyl anion equivalents is *N*‐heterocyclic carbene (NHC) catalysis.^4^ However, the benzoin self‐condensation is, in some cases, a severe side reaction, especially when a ketone is the targeted electrophile and so far, only specific activated ketones could be used in an intermolecular reaction using NHC catalysis.^5^ Thus, the Corey–Seebach umpolung is still widely applied for the synthesis of natural products^6^ as well as for the construction of smaller molecules today.^7^ Accordingly, over the last two decades, significant efforts have been made to advance the dithiane method introduced by Corey and Seebach. Among others,^8^ an anion relay strategy^9^ and the combination with metal‐10 and organocatalysis^11^ (Scheme 1 B) have been developed. Additionally, specific dithianes could be used as radical precursors in photocatalytic transformations for an addition to double bonds (Scheme 1 C).^12^

Despite the advances mentioned above, the deprotonation of aliphatic dithianes still requires strong organometallic bases (e.g. *n*BuLi), as the masked acyl anion exhibits a p*K*a value of approx. 40 (1,3‐dithiane in DMSO).^13^ This limitation prohibits the presence of base‐ and nucleophile‐sensitive functional groups such as halides, esters and nitriles. Likewise, the stoichiometric use of an organometallic reagent generates undesired waste including metal salts.

In order to circumvent these issues, the dithiane would need to be activated by other means than direct deprotonation. With the C−H bond being in alpha position to two sulfur atoms it should be susceptible to a facile hydrogen atom transfer (HAT) opening an alluring alternative activation pathway.^14^ This solution is especially attractive, as in recent years, the combination of photo‐ and HAT‐catalysis enabled the development of several novel reactions and allowed the use of catalytic amounts of a hydrogen abstracting reagent (HAT‐catalyst) instead of a stoichiometric quantity.^15^

Last year, we reported that radicals generated by the combination of photo‐ and HAT‐catalysis can be reduced to render an anionic intermediate capable of reacting with electrophiles.^16^ So far, this method was limited to substrates stabilizing the radical and the anion intermediate by an aromatic group. We wondered if this concept is applicable to aliphatic dithianes for a base‐free Corey–Seebach reaction, hence featuring a novel and improved approach for a classic and established strategy (Scheme 1 D). Other photocatalytic methods to access carbon nucleophiles from sp^3^ C−H bonds are rare, and require a catalytic^17^ or stoichiometric^18^ amount of a chromium source. To the best of our knowledge, the here presented method is the first example for a photocatalytic Corey–Seebach reaction via a carbon nucleophile under metal‐ and base‐free reaction conditions.

The investigation was started employing 2‐methyl‐1,3‐dithaine (**1 a**) and acetone (**2 a**) as simple model substrates (Table 1). Gratifyingly, with 3DPA2FBN as photocatalyst and *i*Pr_3_SiSH as HAT‐catalyst the product was obtained in 32 % GC‐yield (entry 1) using DMF as solvent in the presence of 4 Å molecular sieve at 25 °C. Increasing the concentration (entry 2) improved the reaction outcome slightly, while lowering the temperature to 0 °C proved to be crucial (entry 3). The presence of bis(neopentyl glycolato)diboron (B_2_neop_2_) as mild Lewis acid was beneficial as well (entry 4). After completion of the reaction, degradation products of the HAT‐catalyst were observed by GC‐MS. Thus, the addition of a second catalyst loading was tested as well, resulting in a modest yield gain (entry 5). Overall, an almost full conversion with a good GC‐yield of 73 % translating into an isolated yield of 65 % was achieved. Control experiments revealed the necessity of the combination of light, photo‐ and HAT‐catalyst (entries 6–8). The full detailed optimization process is given in the Supporting Information.

**Table 1:** Reaction optimization.^[a]^

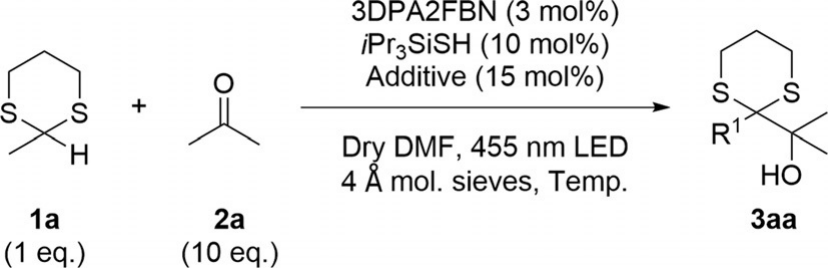					
Entry	Additive	Conc. **1 a** [mm]	Temp. [°C]	Yield^[b]^ [%] **3 aa**	Conv.^[b]^ [%] **1 a**
1	–	100	25	32	48
2	–	200	25	39	59
3	–	200	0	59	76
4	B_2_neop_2_	200	0	65	79
**5** ^[c]^	**B_2_** **neop_2_**	**200**	**0**	**73 (65)**	**87**
6^[d]^	B_2_neop_2_	200	0	n.d.	3
7^[e]^	B_2_neop_2_	200	0	n.d.	1
8^[f]^	B_2_neop_2_	200	0	n.d.	1

[a] Reactions were performed with **1 a** (200 μmol, 1 equiv.) and **2 a** (2.00 mmol, 10 equiv.) in degassed dry DMF (1–2 mL) in the presence of 4 Å molecular sieve (50 mg) and a reaction time of 16 h. [b] Determined by GC‐FID with *n*‐decane as internal standard. [c] After 4 h 3DPA2FBN (2 mol %) and *i*Pr_3_SiSH (10 mol %) were added, the reaction time was prolonged to 19 h. [d] Reaction stirred in the dark. [e] In absence of 3DPA2FBN. [f] In absence of *i*Pr_3_SiSH.

With the optimized conditions, the substrate scope was established (Table 2). The reaction was successful with unsubstituted 1,3‐dithiane (**1 b**) as the nucleophile, the alkyl chain could be prolonged (**1 c**) and an additional heteroatom giving rise to other potentially cleavable C−H bonds did not hamper the reaction (**1 d**). Indeed, the reaction tolerates base or nucleophile sensitive cyano‐ (**1 e**) and ester functionalities (**1 g**) as well as halogen substituted aromatic moieties (**1 h**–**j**) and an unsubstituted phenyl ring (**1 f**).

**Table 2:** Substrate Scope.^[a]^

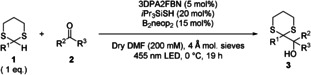
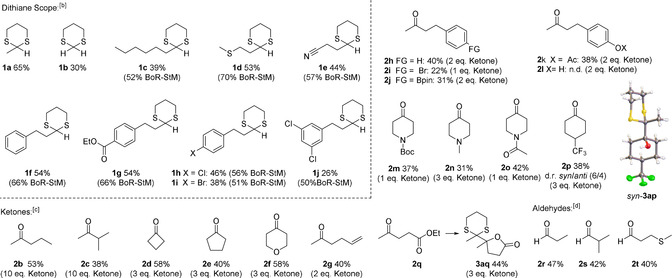

[a] Reactions were run with 1 equiv. set to 200 μmol in degassed dry DMF (1 mL) in presence of 4 Å molecular sieve (50 mg). The reaction was started with 3DPA2FBN (3 mol %) and *i*Pr_3_SiSH (10 mol %). After 4 h, 3DPA2FBN (2 mol %) and *i*Pr_3_SiSH (10 mol %) were added; total reaction time 19 h. [b] Acetone **(2 a)** (10 equiv.) as electrophile. BoR‐StM: Based on recovered starting material. [c] **1 a** (1 equiv.) as nucleophile precursor. [d] **1 a** (3 equiv.) as nucleophile precursor with the aldehyde set to 1 equiv.

The yield generally improves when increasing the equivalents of the electrophile. However, a maximum yield is reached at a specific concentration, which cannot be increased by raising the electrophile concentration any further. Thus, evaluating the electrophile scope, the optimal amount of reactant for every substrate was tested. Prolonging the alkyl chain was viable (**2 b**), while an additional substituent in α‐position decreased the yield due to an increased steric hindrance (**2 c**). Cyclic Ketones (**2 d**–**f**) and the presence of a heteroatom (**2 f**) were both well accepted. A double bond within the electrophile (**2 g**) gave the desired product despite a potential radical addition as side reaction and ketones bearing a phenyl ring with various substituents (**2 h**–**k**) were all tolerated. An exception is the presence of a free ‐OH group (**2 l**), as the anionic dithiane intermediate is likely to be protonated by the fairly acidic alcohol (p*K*a=18.0 for phenol in DMSO)^19^ rather than adding to its ketone moiety. Accordingly, a protected alcohol yielded the wanted product (**2 k**). In terms of nitrogen containing functionalities, a Boc‐protected amine (**2 m**), a tertiary amine (**2 n**) and an amide (**2 o**) were viable electrophiles. A CF_3_‐substituted cyclohexane (**2 p**) gave rise to a diastereomeric product mixture separable by chromatography. An X‐ray crystal structure could verify the structure of the *syn*‐isomer (*syn*‐**3 ap**). With a substrate containing both, an electrophilic ketone and ester group (**2 q**) the nucleophilic addition proceeded exclusively at the more reactive ketone moiety, yielding the corresponding lactone product (**3 aq**) resulting from an attack of the formed alcohol to the ester. Aldehydes were suitable electrophiles as well (**2 r**–**t**). In this case, an excess of dithiane was required, due to a competing hydrogen atom abstraction at the carbonyl group^20^ leading to side reactions (tested low‐ and non‐yielding substrates are listed in section 5 of the Supporting Information).

The described transformation is sensitive to steric hindrance, especially regarding the nucleophile (Scheme 2). A sterically demanding dithiane bearing a cyclohexyl ring (**1 k**) could not be added to acetone giving the desired product **3 ka**. The same was observed with a phenyl ring as substituent (**1 l**). We reasoned that adding an electron donating methoxy substituent (**1 m**) should increase the reactivity of the formed nucleophilic intermediate and indeed isolatable amounts of product **3 ma** were formed in this case.

**Scheme 2:**
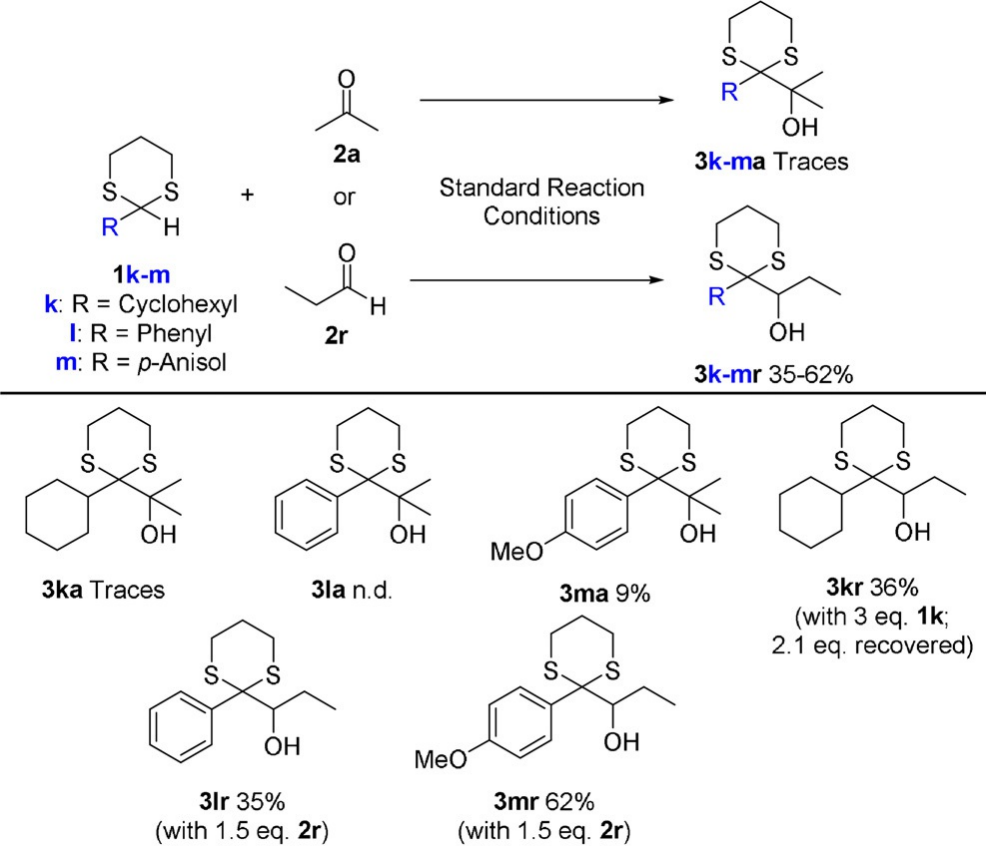
Sterically hindered dithianes as nucleophile precursors.

Consequently, we tested aldehydes as sterically more accessible and reactive electrophiles for **1 k**–**m**, due to their ineffective addition to ketones. As expected, the desired products for all three nucleophile precursors (**3 kr–3 mr**) could be isolated in moderate to good yields. With the reactive C−H bond of **1 l** and **1 m** being in a benzylic position and alpha to both sulfur atoms, it is highly susceptible to HAT and the aldehyde could be added in slight excess. In contrast, an excess of dithiane **1 k** was required to arrive at a reasonable yield of **3 kr**, however, most of the surplus starting material could be recovered after completion of the reaction (Scheme 2).

We decided to exploit these findings testing the selectivity of the reaction (Scheme 3). A substrate bearing both, a sterically demanding and an open dithiane site was selectively functionalized at the more accessible position (**3 na**). A substrate bearing a benzylic as well as an aliphatic dithiane moiety could be selectively functionalized at the benzylic position (**3 or**), as the HAT activation step is likely to occur at the C−H bond with a lower BDE. However, an aldehyde is required as reaction partner in this case. No product could be isolated with acetone as electrophile.

**Scheme 3:**
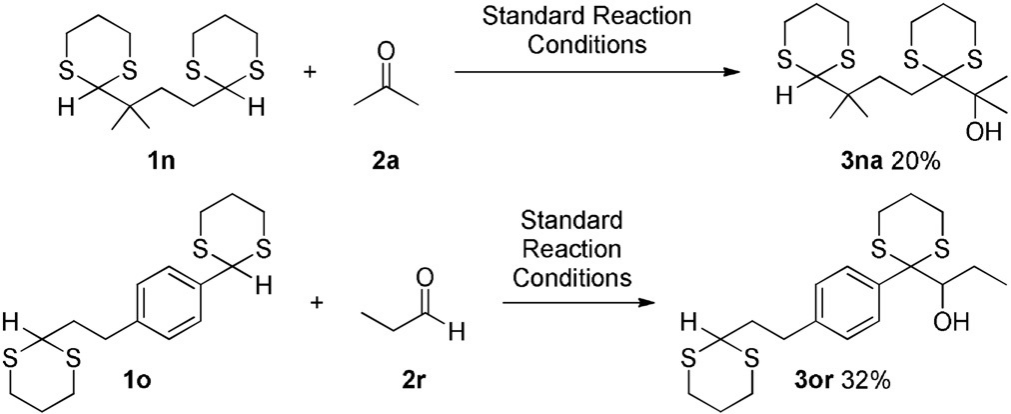
Selective functionalization of dithianes with two reactive sites.

Lastly, the dithiane deprotection to valuable α‐hydroxy ketones was investigated for selected examples. Among others, photocatalytic oxidations,^21^ as well as an elegant Bi(NO_3_)_3_⋅5 H_2_O catalyzed method^22^ have already been developed by other groups. After a preliminary optimization (see Supporting Information), both strategies afforded the desired product in good yields, with the Bi(NO_3_)_3_⋅5 H_2_O method being more efficient (Scheme 4). A one‐pot procedure starting with the photocatalytic Corey–Seebach reaction followed by the deprotection as the second step is possible, yet low yielding (see Supporting Information Scheme S3).

**Scheme 4:**
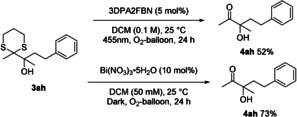
Dithiane deprotection.

In the proposed mechanism, the oxidation of the HAT‐catalyst by the excited photocatalyst is the first step. This hypothesis was supported by emission quenching studies indicating an interaction between the excited 3DPA2FBN and *i*Pr_3_SiSH in the presence of 4 Å molecular sieve (Figure 1 A, left).

**Figure 1:**
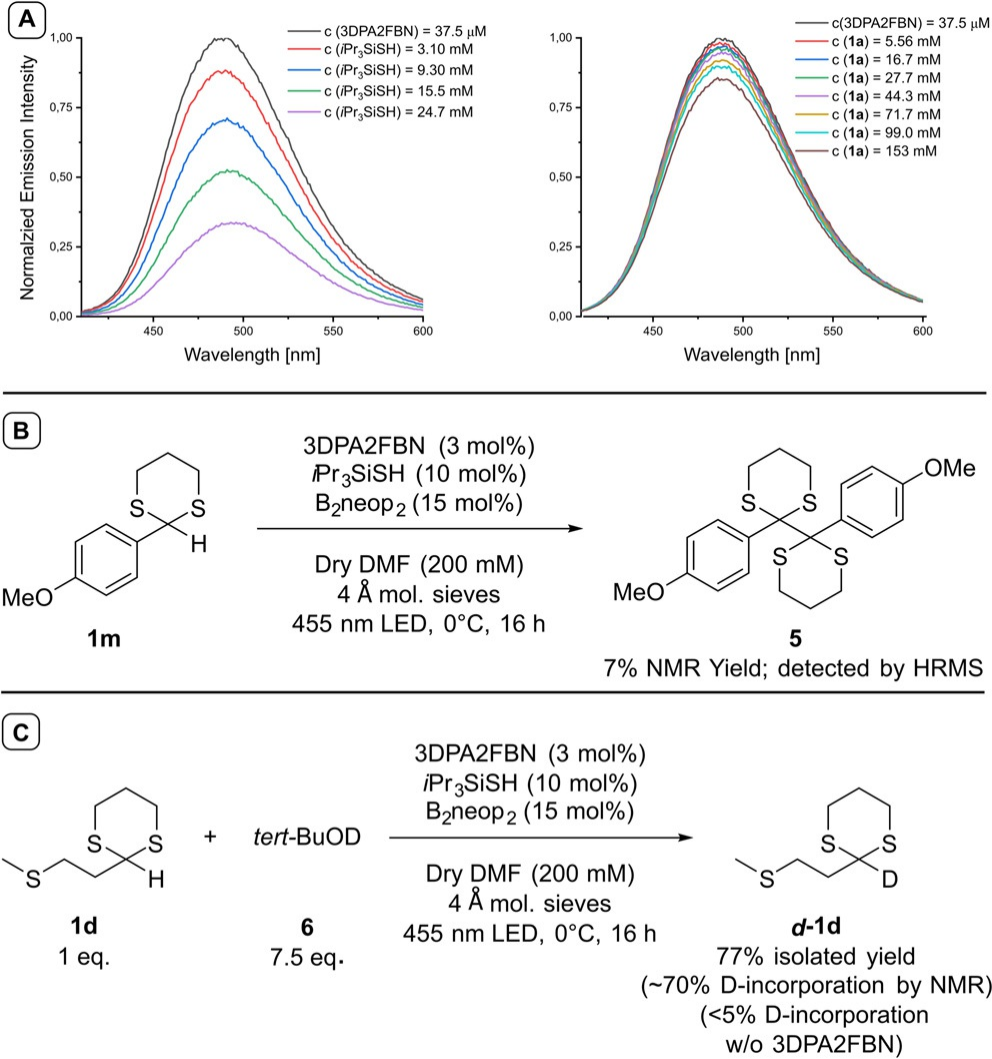
**A**: Emission quenching of 3DPA2FBN by addition of *i*Pr_3_SiSH (left) and **1 a** (right). **B**: Radical–radical homocoupling of **1 m**. The reaction was conducted in 200 μmol scale. **C**: Deuterium labeling experiments. Reactions run with 1 equiv. set to 200 μmol.

The same effect was not observed using **1 a** (Figure 1 A, right) or **2 a** (Figure S5). The activated HAT catalyst (S‐H BDE: 88.9 kcal mol^−1^)^23^ should then abstract a hydrogen atom from the dithiane substrate. The corresponding radical–radical homocoupling side product **5** could be detected by HRMS for **1 m** as a selected example after the reaction reached completion. A higher quantity could be detected by NMR when omitting the electrophile (Figure 1 B).

Ultimately, the reduced photocatalyst (E_1/2_ (3DPA2FBN/3DPA2FBN^⋅−^)=−1.92 V vs. SCE)^24^ should be capable to transfer an electron to the dithiane radical (E_1/2_ (**1 a** ^.^/**1 a** ^−^)=−1.87 V vs. SCE),^23^ giving rise to the carbanion nucleophile. Deuterium labeling studies could experimentally support this step. With deuterated *tert*‐butanol (*tert*BuOD) as electrophile, the expected deuterated dithiane (***d*** **‐1 d**) could be isolated under the standard reaction conditions with a deuterium incorporation of approx. 70 % (Figure 1 C). No deuterium incorporation was observed in absence of the photocatalyst. The dithiane radical (C−H BDE **1 a**: 88.2 kcal mol^−1^)^23^ should not be reactive enough to abstract the deuterium from *tert*BuOD (O−H BDE: 106.3 kcal mol^−1^),20a, 25 whereas an anionic intermediate (p*K*a 1,3‐dithiane approx. 40 in DMSO)^13^ is expected to do so (p*K*a *tert*BuOH=32.2 in DMSO^26^), clearly indicating its formation. The generation of an ionic species from a radical intermediate can be dubbed as radical‐polar crossover and the application of this strategy in photocatalysis is a currently growing field in interest of several research groups.^27^ As shown in Scheme 2, the active nucleophilic species is highly sensitive to steric hindrance and thus less reactive than expected for a classical lithiated dithiane. Therefore, the exact nature of the anionic intermediate is so far unknown and subject of current investigations.

Based on the mechanistic studies and previous reports, following mechanism is proposed (Scheme 5): the excited 3DPA2FBN photocatalyst oxidizes the *i*Pr_3_SiSH HAT catalyst giving rise to the radical anion of 3DPA2FBN and the *i*Pr_3_SiS^.^ radical after deprotonation. The thus activated HAT catalyst abstracts a hydrogen atom from the most labile C−H bond, which is in alpha position to both sulfur atoms within the dithiane **1**, regenerating the HAT‐catalyst and forming the corresponding radical species **1** ^.^. The photocatalytic cycle is then closed by reduction of **1** ^.^, yielding anionic key intermediate **1^−^**. This carbanion nucleophile is capable to attack non‐activated ketones (**2**) to furnish the desired Corey–Seebach product **3** after protonation of the alcoholate.

**Scheme 5:**
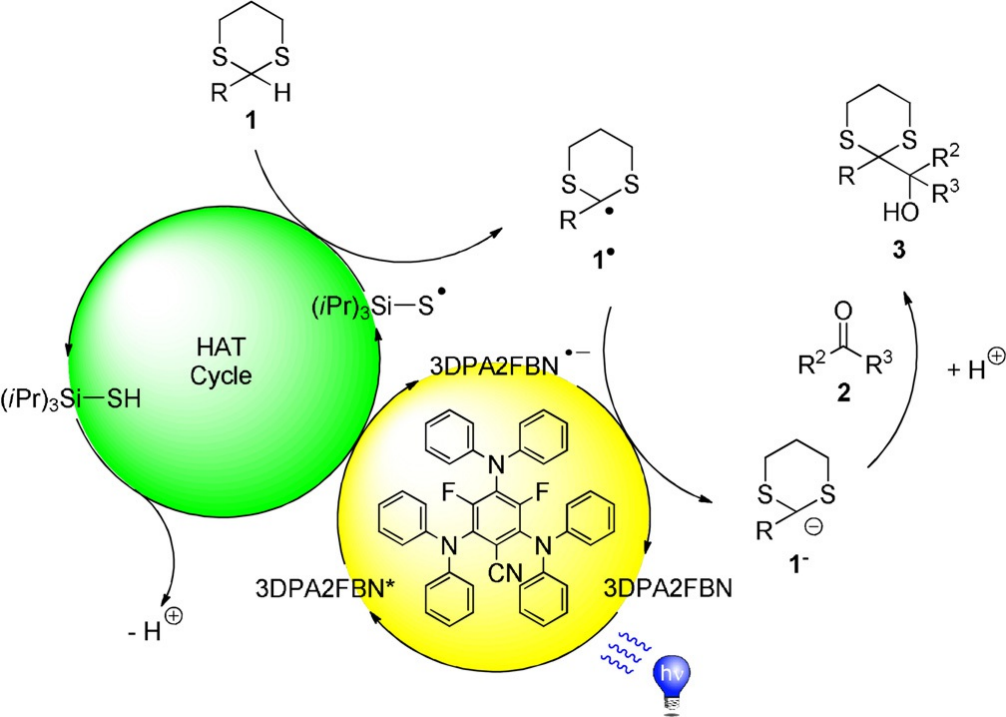
Proposed mechanism.

In summary, we have developed a photocatalytic Corey–Seebach reaction operating under mild metal‐ and base‐free conditions, employing solely catalytic additives. Base‐ and nucleophile sensitive functional groups are tolerated and ketones as well as aldehydes are viable electrophiles giving the desired product in moderate to good yields. The reaction is enabled by the combination of photo‐ and hydrogen atom transfer catalysis. This catalytic merger allows the activation of suitable C−H bonds to carbanions capable of reacting with carbonyl electrophiles, the mechanism of which is supported experimentally as well as by calculated redox potentials and bond strengths. The anionic intermediate seems to be less reactive than the classical lithiated species, allowing regio‐ and chemo‐selective transformations. However, the exact nature of the carbanion species remains as of now unknown and is under current investigation by time resolved spectroscopy and in situ NMR studies.

## Conflict of interest

The authors declare no conflict of interest.

## Supporting information

As a service to our authors and readers, this journal provides supporting information supplied by the authors. Such materials are peer reviewed and may be re‐organized for online delivery, but are not copy‐edited or typeset. Technical support issues arising from supporting information (other than missing files) should be addressed to the authors.

Supplementary

## References

[1a] F. A. Davis , B. C. Chen , Chem. Rev. 1992, 92, 919–934;

[1b] G. Minotti , P. Menna , E. Salvatorelli , G. Cairo , L. Gianni , Pharmacol. Rev. 2004, 56, 185–229; 15169927 10.1124/pr.56.2.6

[1c] A. Tiaden , T. Spirig , H. Hilbi , Trends Microbiol. 2010, 18, 288–297; 20382022 10.1016/j.tim.2010.03.004

[1d] X. L. Sun , H. Takayanagi , K. Matsuzaki , H. Tanaka , K. Furuhata , S. Omura , J. Antibiot. 1996, 49, 689–692; 10.7164/antibiotics.49.689 8784432

[1e] O. B. Wallace , D. W. Smith , M. S. Deshpande , C. Polson , K. M. Felsenstein , Bioorg. Med. Chem. Lett. 2003, 13, 1203–1206; 12643944 10.1016/s0960-894x(02)01058-2

[1f] X. Wang , J. G. Sena Filho , A. R. Hoover , J. B. King , T. K. Ellis , D. R. Powell , R. H. Cichewicz , J. Nat. Prod. 2010, 73, 942–948; 20450206 10.1021/np100142h PMC2878378

[1g] X. Peng , Y. Wang , K. Sun , P. Liu , X. Yin , W. Zhu , J. Nat. Prod. 2011, 74, 1298–1302; 21381678 10.1021/np1008976

[1h] L. Lin , N. Mulholland , Q. Y. Wu , D. Beattie , S. W. Huang , D. Irwin , J. Clough , Y. C. Gu , G. F. Yang , J. Agric. Food Chem. 2012, 60, 4480–4491; 22439963 10.1021/jf300610j

[1i] P. Wang , W. Kim , L. B. Pickens , X. Gao , Y. Tang , Angew. Chem. Int. Ed. 2012, 51, 11136–11140; 10.1002/anie.201205426 PMC4070387 23024027

[1j] Z. Wang , Y. Sheng , H. Duan , Q. Yu , W. Shi , S. Zhang , RSC Adv. 2014, 4, 53788–53794.

[2] E. J. Corey , D. Seebach , Angew. Chem. Int. Ed. Engl. 1965, 4, 1075–1077;

[3a] D. Seebach , E. J. Corey , J. Org. Chem. 1975, 40, 231–237;

[3b] D. Seebach , M. Kolb , Liebigs Ann. Chem. 1977, 811–829;

[3c] B.-T. Gröbel , D. Seebach , Synthesis 1977, 357–402;

[3d] D. Seebach , Angew. Chem. Int. Ed. Engl. 1979, 18, 239–258;

[4a] X. Bugaut , F. Glorius , Chem. Soc. Rev. 2012, 41, 3511–3522; 22377957 10.1039/c2cs15333e

[4b] D. M. Flanigan , F. Romanov-Michailidis , N. A. White , T. Rovis , Chem. Rev. 2015, 115, 9307–9387. 25992594 10.1021/acs.chemrev.5b00060 PMC4986729

[5a] Y.-L. Liu , X.-T. Lin , Adv. Synth. Catal. 2019, 361, 876–918;

[5b] D. Enders , A. Henseler , Adv. Synth. Catal. 2009, 351, 1749–1752;

[5c] C. A. Rose , S. Gundala , C.-L. Fagan , J. F. Franz , S. J. Connon , K. Zeitler , Chem. Sci. 2012, 3, 735–740;

[5d] E. Sánchez-Díez , M. Fernandez , U. Uria , E. Reyes , L. Carrillo , J. L. Vicario , Chem. Eur. J. 2015, 21, 8384–8388; 25907587 10.1002/chem.201501044

[5e] J. Xu , J. Peng , C. He , H. Ren , Org. Chem. Front. 2019, 6, 172–176.

[6a] M. Yus , C. Nájera , F. Foubelo , Tetrahedron 2003, 59, 6147–6212;

[6b] A. B. Smith III , C. M. Adams , Acc. Chem. Res. 2004, 37, 365–377; 15196046 10.1021/ar030245r

[6c] G. K. Murphy , T. Shirahata , N. Hama , A. Bedermann , P. Dong , T. C. McMahon , B. M. Twenter , D. A. Spiegel , I. M. McDonald , N. Taniguchi , M. Inoue , J. L. Wood , J. Org. Chem. 2013, 78, 477–489; 23245580 10.1021/jo302353g

[6d] G. Xiao , B. Yu , Chem. Eur. J. 2013, 19, 7708–7712; 23649953 10.1002/chem.201301186

[6e] R. Liffert , J. Hoecker , C. K. Jana , T. M. Woods , P. Burch , H. J. Jessen , M. Neuburger , K. Gademann , Chem. Sci. 2013, 4, 2851–2857;

[6f] A. L. Hurski , Y. V. Ermolovich , V. N. Zhabinskii , V. A. Khripach , Org. Biomol. Chem. 2015, 13, 1446–1452; 25473936 10.1039/c4ob02197e

[6g] D. Das , T. K. Chakraborty , Org. Lett. 2017, 19, 682–685; 28105814 10.1021/acs.orglett.6b03854

[6h] P. M. Wright , A. G. Myers , Tetrahedron 2011, 67, 9853–9869; 22102762 10.1016/j.tet.2011.09.143 PMC3217274

[6i] M. Henrot , M. E. Richter , J. Maddaluno , C. Hertweck , M. De Paolis , Angew. Chem. Int. Ed. 2012, 51, 9587–9591; 10.1002/anie.201204259 22807210

[6j] H. Matsumoto , M. Yamashita , T. Tahara , S. Hayakawa , S. I. Wada , K. Tomioka , A. Iida , Bioorg. Med. Chem. 2017, 25, 4133–4144; 28619446 10.1016/j.bmc.2017.06.001

[6k] F. A. Almalki , D. C. Harrowven , Eur. J. Org. Chem. 2016, 5738–5746;

[6l] X. H. Li , M. Zhu , Z. X. Wang , X. Y. Liu , H. Song , D. Zhang , F. P. Wang , Y. Qin , Angew. Chem. Int. Ed. 2016, 55, 15667–15671; 10.1002/anie.201609882 27860043

[6m] Q. Liu , Y. Deng , A. B. Smith III , J. Am. Chem. Soc. 2017, 139, 13668–13671; 28933833 10.1021/jacs.7b08683 PMC5655813

[6n] M. H. Nguyen , M. Imanishi , T. Kurogi , X. Wan , J. E. Ishmael , K. L. McPhail , A. B. Smith III , J. Org. Chem. 2018, 83, 4287–4306; 29480727 10.1021/acs.joc.8b00268 PMC5910188

[6o] J. Li , T. S. Ahmed , C. Xu , B. M. Stoltz , R. H. Grubbs , J. Am. Chem. Soc. 2019, 141, 154–158. 30537831 10.1021/jacs.8b12816

[7a] X. Xie , G. Yue , S. Tang , X. Huo , Q. Liang , X. She , X. Pan , Org. Lett. 2005, 7, 4057–4059; 16119966 10.1021/ol051653t

[7b] H. Redlich , Y.-L. Chen , R. Leguijt , R. Fröhlich , Synthesis 2006, 2006, 4212–4218;

[7c] M. Z. Chen , G. C. Micalizio , J. Am. Chem. Soc. 2012, 134, 1352–1356; 22103772 10.1021/ja2105703 PMC3262091

[7d] C. Englert , I. Nischang , C. Bader , P. Borchers , J. Alex , M. Prohl , M. Hentschel , M. Hartlieb , A. Traeger , G. Pohnert , S. Schubert , M. Gottschaldt , U. S. Schubert , Angew. Chem. Int. Ed. 2018, 57, 2479–2482; 10.1002/anie.201710756 29214708

[7e] G. J. Boehlich , N. Schützenmeister , Angew. Chem. Int. Ed. 2019, 58, 5110–5113; 10.1002/anie.201900995 30768828

[7f] N. Trongsiriwat , Y. Pu , Y. Nieves-Quinones , R. A. Shelp , M. C. Kozlowski , P. J. Walsh , Angew. Chem. Int. Ed. 2019, 58, 13416–13420; 10.1002/anie.201905531 PMC6788743 31291500

[8a] S. Tang , J. Han , J. He , J. Zheng , Y. He , X. Pan , X. She , Tetrahedron Lett. 2008, 49, 1348–1351;

[8b] Y. F. Liu , L. Zheng , D. D. Zhai , X. Y. Zhang , B. T. Guan , Org. Lett. 2019, 21, 5351–5356. 31247782 10.1021/acs.orglett.9b01994

[9] A. B. Smith III , M. Xian , J. Am. Chem. Soc. 2006, 128, 66–67. 16390124 10.1021/ja057059w

[10a] W. Du , L. Tian , J. Lai , X. Huo , X. Xie , X. She , S. Tang , Org. Lett. 2014, 16, 2470–2473; 24749868 10.1021/ol500850d

[10b] S. A. Baker Dockrey , A. K. Makepeace , J. R. Schmink , Org. Lett. 2014, 16, 4730–4733; 25192489 10.1021/ol502428h

[10c] B. Yucel , P. J. Walsh , Adv. Synth. Catal. 2014, 356, 3659–3667; 26185491 10.1002/adsc.201400695 PMC4500739

[10d] K. Yao , D. Liu , Q. Yuan , T. Imamoto , Y. Liu , W. Zhang , Org. Lett. 2016, 18, 6296–6299. 27978643 10.1021/acs.orglett.6b03161

[11a] E. Massolo , M. Benaglia , A. Genoni , R. Annunziata , G. Celentano , N. Gaggero , Org. Biomol. Chem. 2015, 13, 5591–5596; 25883074 10.1039/c5ob00492f

[11b] A. Kondoh , M. Oishi , T. Takeda , M. Terada , Angew. Chem. Int. Ed. 2015, 54, 15836–15839; 10.1002/anie.201508178 26480953

[12a] A. Nishida , M. Nishida , O. Yonemitsu , Tetrahedron Lett. 1990, 31, 7035–7038;

[12b] Y. Li , K. Miyazawa , T. Koike , M. Akita , Org. Chem. Front. 2015, 2, 319–323;

[12c] M. Lee , Y.-H. Chen , T.-H. Hung , W. Chang , W.-C. Yan , D. Leow , RSC Adv. 2015, 5, 86402–86406;

[12d] A. Gualandi , E. Matteucci , F. Monti , A. Baschieri , N. Armaroli , L. Sambri , P. G. Cozzi , Chem. Sci. 2017, 8, 1613–1620. 28451291 10.1039/c6sc03374a PMC5364518

[13a] F. G. Bordwell , G. E. Drucker , N. H. Andersen , A. D. Denniston , J. Am. Chem. Soc. 1986, 108, 7310–7313;

[13b] B. D. Süveges , J. Podlech , Eur. J. Org. Chem. 2015, 987–994.

[14a] H.-S. Dang , B. P. Roberts , Tetrahedron Lett. 1999, 40, 8929–8933;

[14b] Y. Deng , M. D. Nguyen , Y. Zou , K. N. Houk , A. B. Smith III , Org. Lett. 2019, 21, 1708–1712. 30807194 10.1021/acs.orglett.9b00271 PMC6518407

[15a] S. Protti , M. Fagnoni , D. Ravelli , ChemCatChem 2015, 7, 1516–1523;

[15b] L. Capaldo , D. Ravelli , Eur. J. Org. Chem. 2017, 2056–2071; 10.1002/ejoc.201601485 PMC6099384 30147436

[15c] D. Ravelli , M. Fagnoni , T. Fukuyama , T. Nishikawa , I. Ryu , ACS Catal. 2018, 8, 701–713;

[15d] L. Capaldo , L. L. Quadri , D. Ravelli , Green Chem. 2020, 22, 3376–3396;

[15e] X. Z. Fan , J. W. Rong , H. L. Wu , Q. Zhou , H. P. Deng , J. D. Tan , C. W. Xue , L. Z. Wu , H. R. Tao , J. Wu , Angew. Chem. Int. Ed. 2018, 57, 8514–8518; 10.1002/anie.201803220 29718584

[15f] Y. Shen , Y. Gu , R. Martin , J. Am. Chem. Soc. 2018, 140, 12200–12209; 30184423 10.1021/jacs.8b07405

[15g] A. Dewanji , P. E. Krach , M. Rueping , Angew. Chem. Int. Ed. 2019, 58, 3566–3570; 10.1002/anie.201901327 30776185

[15h] M. D. Vu , M. Das , A. X. Guo , Z. E. Ang , M. Dokic , H. S. Soo , X. W. Liu , ACS Catal. 2019, 9, 9009–9014;

[15i] Y. Li , M. Lei , L. Gong , Nat. Catal. 2019, 2, 1016–1026;

[15j] Q. An , Z. Wang , Y. Chen , X. Wang , K. Zhang , H. Pan , W. Liu , Z. Zuo , J. Am. Chem. Soc. 2020, 142, 6216–6226. 32181657 10.1021/jacs.0c00212

[16a] A. L. Berger , K. Donabauer , B. König , Chem. Sci. 2019, 10, 10991–10996; 10.1039/c9sc04987h PMC8133029 34040714

[16b] Q. Y. Meng , T. E. Schirmer , A. L. Berger , K. Donabauer , B. König , J. Am. Chem. Soc. 2019, 141, 11393–11397. 31280561 10.1021/jacs.9b05360 PMC6686948

[17a] J. L. Schwarz , F. Schafers , A. Tlahuext-Aca , L. Luckemeier , F. Glorius , J. Am. Chem. Soc. 2018, 140, 12705–12709; 30216059 10.1021/jacs.8b08052

[17b] H. Mitsunuma , S. Tanabe , H. Fuse , K. Ohkubo , M. Kanai , Chem. Sci. 2019, 10, 3459–3465. 30996935 10.1039/c8sc05677c PMC6430092

[18a] K. Yahata , S. Sakurai , S. Hori , S. Yoshioka , Y. Kaneko , K. Hasegawa , S. Akai , Org. Lett. 2020, 22, 1199–1203; 31939300 10.1021/acs.orglett.0c00096

[18b] K. Yahata , S. Yoshioka , S. Hori , S. Sakurai , Y. Kaneko , K. Hasegawa , S. Akai , Chem. Pharm. Bull. 2020, 68, 336–338. 10.1248/cpb.c20-00075 32074521

[19] F. G. Bordwell , R. J. McCallum , W. N. Olmstead , J. Org. Chem. 1984, 49, 1424–1427.

[20a] A. Banerjee , Z. Lei , M. Y. Ngai , Synthesis 2019, 51, 303–333; 31057188 10.1055/s-0037-1610329 PMC6497162

[20b] K. Yoshikai , T. Hayama , K. Nishimura , K. Yamada , K. Tomioka , J. Org. Chem. 2005, 70, 681–683; 15651818 10.1021/jo048275a

[20c] B. P. Roberts , Chem. Soc. Rev. 1999, 28, 25–35.

[21a] M. Kamata , Y. Murakami , Y. Tamagawa , M. Kato , E. Hasegawa , Tetrahedron 1994, 50, 12821–12828;

[21b] P. D. Dharpure , A. Bhowmick , P. K. Warghude , R. G. Bhat , Tetrahedron Lett. 2020, 61, 151407.

[22] N. Komatsu , A. Taniguchi , S. Wada , H. Suzuki , Adv. Synth. Catal. 2001, 343, 473–480.

[23] Calculated value, see Supporting Information.

[24] E. Speckmeier , T. G. Fischer , K. Zeitler , J. Am. Chem. Soc. 2018, 140, 15353–15365. 30277767 10.1021/jacs.8b08933

[25] K. M. Ervin , V. F. DeTuri , J. Phys. Chem. A 2002, 106, 9947–9956.

[26] W. N. Olmstead , Z. Margolin , F. G. Bordwell , J. Org. Chem. 1980, 45, 3295–3299.

[27a] R. J. Wiles , G. A. Molander , Isr. J. Chem. 2020, 60, 281–293; 10.1002/ijch.201900166 PMC8115720 33986554

[27b] L. Pitzer , J. L. Schwarz , F. Glorius , Chem. Sci. 2019, 10, 8285–8291; 32055300 10.1039/c9sc03359a PMC7003961

[27c] J. P. Phelan , S. B. Lang , J. S. Compton , C. B. Kelly , R. Dykstra , O. Gutierrez , G. A. Molander , J. Am. Chem. Soc. 2018, 140, 8037–8047; 29916711 10.1021/jacs.8b05243 PMC6540794

[27d] C. Shu , R. S. Mega , B. J. Andreassen , A. Noble , V. K. Aggarwal , Angew. Chem. Int. Ed. 2018, 57, 15430–15434; 10.1002/anie.201808598 PMC6282618 30204292

[27e] L. L. Liao , G. M. Cao , J. H. Ye , G. Q. Sun , W. J. Zhou , Y. Y. Gui , S. S. Yan , G. Shen , D. G. Yu , J. Am. Chem. Soc. 2018, 140, 17338–17342. 30518213 10.1021/jacs.8b08792

