# Supplementary material for: Photocatalytic Reductive Radical‐Polar Crossover for a Base‐Free Corey–Seebach Reaction
Source: Chemistry. 2020 Sep 17;26(57):12945–50. doi: 10.1002/chem.202003000 (PMC7589390; doi:10.1002/chem.202003000)
Supplement: Supplementary file 1 — Supplementary [file CHEM-26-12945-s001.pdf]

Chemistry–A European Journal

Supporting Information

Photocatalytic Reductive Radical-Polar Crossover for a Base-Free Corey–Seebach Reaction

Karsten Donabauer,[a] Kathiravan Murugesan,[a] Urša Rozman,[a] Stefano Crespi,[b] and Burkhard König*[a]

Table of contents

1. General information 1

2. Synthetic procedures 3

2.1 Synthesis of photocatalysts 3

2.2 Synthesis of starting materials 6

3. Photocatalytic base-free Corey-Seebach reaction 18

4. Detailed reaction optimization process 35

5. Lower-yielding or unsuccessful electrophiles and 1,3-dithanes 44

6. 1,3-Dithian deprotection 47

7. Mechanistic investigations 50

7.1 Emission quenching studies 50

7.2 Radical-radical homocoupling 54

7.3 Deuterium labeling studies 56

8. Computational analysis 61

8.1 Cartesian coordinates and energies 62

9. NMR-spectra 68

10. References 131

1. General information

Starting materials and reagents were purchased from commercial suppliers (Sigma Aldrich, Alfa Aesar, Fluorochem, Acros, Fluka, TCI or VWR) and used without further purification. Solvents for reactions were distilled or the commercially available p.a. grade was used. Dry solvents were prepared by storing the according p.a. grade solvent over molecular sieves. Dry DMF was purchased from Acros. For automated flash column chromatography distilled or p.a. grade solvents were used. All reactions with oxygen- or moisture-sensitive reagents were carried out in glassware, which was dried before use by heating under vacuum. Dry nitrogen was used as inert gas atmosphere. Liquids were added *via* syringe, needle and septum techniques unless otherwise stated.

All NMR spectra were measured at room temperature using a Bruker Avance 300 (300 MHz for ^{1}H, 75 MHz for ^{13}C, 282 MHz for ^{19}F) or a Bruker Avance 400 (400 MHz for ^{1}H, 101 MHz for ^{13}C, 376 MHz for ^{19}F)[1] NMR spectrometer. All chemical shifts are reported in δ-scale as parts per million [ppm] (multiplicity, coupling constant *J*, number of protons) relative to the solvent residual peaks as the internal standard.[2] Coupling constants *J* are given in Hertz [Hz]. Abbreviations used for signal multiplicity: ^{1}H-NMR: b = broad, s = singlet, d = doublet, t = triplet, q = quartet, p = quintet, and m = multiplet;

^{13}C-NMR: (+) = primary/tertiary, (–) = secondary, (C_q) = quaternary carbon.

HRMS (high resolution mass spectra) were measured at the Central Analytical Laboratory of the University of Regensburg. These mass spectra were recorded on a Finnigan MAT 95, ThermoQuest Finnigan TSQ 7000, Finnigan MAT SSQ 710 A or an Agilent Q-TOF 6540 UHD instrument.

GC measurements were performed on a GC 7890 from Agilent Technologies. Data acquisition and evaluation was done with Agilent ChemStation Rev.C.01.04. GC/MS measurements were performed on a 7890A GC system from Agilent Technologies with an Agilent 5975 MSD Detector. Data acquisition and evaluation was done with MSD ChemStation E.02.02.1431. A capillary column HP-5MS/30 m x 0.25 mm/0.25 μM film and helium as carrier gas (flow rate of 1 mL/min) were used. The injector temperature (split injection: 40:1 split) was 280 °C, detection temperature 300 °C (FID). GC measurements were made and investigated *via* integration of the signal obtained. The GC oven temperature program was adjusted as follows: initial temperature 40 °C was kept for 3 minutes, the temperature was increased at a rate of 15 °C/min over a period of 16 minutes until 280 °C was reached and kept for 5 minutes, the temperature was again increased at a rate of 25 °C/min over a period of 48 seconds until the final temperature (300 °C) was reached and kept for 5 minutes. *n*-Decane was used as an internal standard.

Analytical TLC was performed on silica gel coated alumina plates (MN TLC sheets ALUGRAM® Xtra SIL G/UV_{254}). Visualization was done by UV light (254 or 366 nm). If necessary, potassium permanganate or a cerium/molybdate stain was used for chemical staining.

Purification by column chromatography was performed with silica gel 60 M (40-63 µm, 230-440 mesh, Merck) or with a pre-packed Biotage® Snap Ultra HP-Sphere™ 25 *u*m column on a Biotage® Isolera™ Spektra One device.

For irradiation with blue light OSRAM Oslon SSL 80 LDCQ7P-1U3U (blue, λ_{max} = 455 nm, I_{max} = 1000 mA, 1.12 W) was used. For irradiation with 400 nm Edison EDEV-SLC1-03 (λ_{max} = 400 nm, I_{max} = 700 mA, 400 mW) was used.

Fluorescence spectra were measured on a HORIBA FluoroMax®-4 Spectrofluorometer at room temperature. Gas tight 10 mm Hellma® quartz fluorescence cuvettes with a screw cap with PTFE-coated silicon septum were used. FluorEssence Version 3.5.1.20 was used as a software for measurement and analysis.

2. Synthetic procedures

2.1 Synthesis of photocatalysts

2,4,5,6-Tetrakis(carbazol-9-yl)-4,6-dicyanobenzene (4CzIPN)

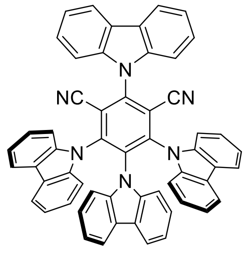

4CzIPN

The photocatalyst was synthesized according to a literature procedure.[3]

NaH (60% in paraffin oil, 600 mg, 15 mmol, 7.5 eq.) was slowly added to a stirred solution of carbazole (1.67 g, 10 mmol, 5 eq.) in dry THF (40 mL). The reaction mixture was heated to 35 °C and stirred for 1 h before adding tetrafluoroisophthalonitrile (400 mg, 2 mmol, 1 eq.). The reaction mixture was stirred at r.t for 24 h, afterwards quenched by H_2O (2 mL) and concentrated *in vacuo*. The solvent was removed and the solid residue was taken up in DCM (50 mL). The organic phase was washed with brine (2x50 mL) and the organic phase was dried over Na_2SO_4. The solvent was removed and the residue was purified by automated flash column chromatography (PE/DCM, 20-70%) to give 2,4,5,6-tetrakis(carbazol-9-yl)-4,6-dicyano-benzene (4CzIPN) as bright yellow powder (840 mg, 1.06 mmol, 53%).

^{1}H-NMR (400 MHz, $CDCl_3$, δ_H): 8.22 (d, *J* = 7.7 Hz, 2H), 7.75 – 7.67 (m, 8H), 7.52 – 7.47 (m, 2H), 7.33 (d, *J* = 7.8 Hz, 2H), 7.25 – 7.19 (m, 4H), 7.12 – 7.05 (m, 8H), 6.82 (t, *J* = 8.2 Hz, 4H), 6.63 (td, *J* = 7.6, 1.2 Hz, 2H).

^{13}C-NMR (101 MHz, $CDCl_3$, δ_C): 145.3, 144.7, 140.1, 138.3, 137.1, 134.9, 127.1, 125.9, 125.1, 124.9, 124.7, 124.0, 122.5, 122.1, 121.5, 121.1, 120.6, 119.8, 116.5, 111.8, 110.1, 109.6, 109.6.

2,4,6-Tris(diphenylamino)-3,5-difluorobenzonitrile (3DPA2FBN)

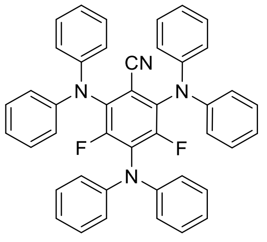

3DPA2FBN

The photocatalyst was synthesized according to a literature procedure.[3]

NaH (60% in paraffin oil, 450 mg, 11.3 mmol, 5.65 eq.) was slowly added to a stirred solution of diphenylamine (1.27 g, 7.5 mmol, 3.75 eq.) in dry THF (40 mL). The reaction mixture was heated to 50 °C and stirred for 1 h before adding pentafluorobenzonitirle (252 µL, 2 mmol, 1 eq.) at r.t. The reaction mixture was stirred at r.t for 24 h, afterwards quenched by H_2O (2 mL) and concentrated *in vacuo*. The solvent was removed and the solid residue was taken up in DCM (50 mL). The organic phase was washed with brine (2x50 mL) and the organic phase was dried over Na_2SO_4. The solvent was removed and the residue was purified by automated flash column chromatography (PE/DCM, 20-80%) to give 2,4,6-tris(diphenylamino)-3,5-difluorobenzonitrile (3DPA2FBN) as bright yellow powder (893 mg, 1.40 mmol, 70%).

^{1}H-NMR (300 MHz, $CDCl_3$, δ_H) 7.26 – 7.20 (m, 12H), 7.07 – 7.00 (m, 6H), 6.99 – 6.95 (m, 12H).

^{19}F-NMR (282 MHz, $CDCl_3$, δ_F) -120.72 (s).

2,4,6-Tris(diphenylamino)-5-fluoroisophthalonitrile (3DPAFIPN)

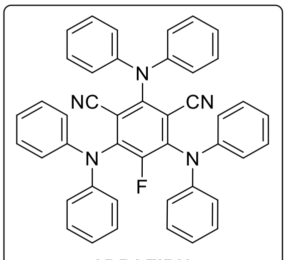

The photocatalyst was synthesized according to a literature procedure.[3]

NaH (60% in paraffin oil, 750 mg, 18.8 mmol, 9.4 eq.) was slowly added to a stirred solution of diphenylamine (1.27 g, 10 mmol, 5 eq.) in dry THF (40 mL). The reaction mixture was heated to 50 °C and stirred for 1 h before adding tetrafluoroisophthalonitrile (400 mg, 2 mmol, 1 eq.) at r.t. The reaction mixture was stirred at r.t for 24 h, afterwards quenched by H_2O (2 mL) and concentrated *in vacuo*. The solvent was removed and the solid residue was taken up in DCM (50 mL). The organic phase was washed with brine (2x50 mL) and the organic phase was dried over Na_2SO_4. The solvent was removed and the residue was purified by automated flash column chromatography (PE/DCM, 20-80%) to give 2,4,6-tris(diphenylamino)-5-fluoroisophthalonitrile (3DPAFIPN) (910 mg, 1.40 mmol, 70%) as bright yellow powder

^{1}H-NMR (400 MHz, $CDCl_3$, δ_H): 7.30 – 7.23 (m, 12H), 7.11-7.02 (m, 6H), 7.02 – 6.96 (m, 12H).

^{19}F-NMR (377 MHz, $CDCl_3$, δ_F): -121.81 (s).

2,3,5,6-Tetrakis(carbazol-9-yl)benzonitrile (4Cz(*p*H)BN)

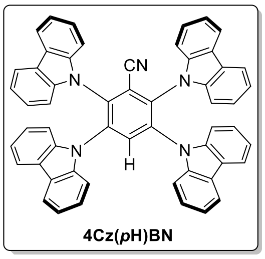

The photocatalyst was synthesized analogous to 4CzIPN with 2,3,5,6-tetrafluorobenzonitirle (350 mg, 2 mmol, 1 eq.) instead of of tetrafluoroisophthalonitrile. The crude product was purified by automated flash column chromatography (PE/DCM 20-80%). 2,3,5,6-Tetrakis(carbazol-9-yl)benzonitrile (4Cz(*p*H)BN) (1.00 g, 1.31 mmol, 66%) was obtained as pale yellow powder.

^{1}H-NMR (300 MHz, $CDCl_3$, δ_H): 8.44 (s, 1H), 7.82-7.74 (m, 8H), 7.39-7.280 (m, 8H), 7.23-7.08 (m, 16H).

^{13}C-NMR (75 MHz, $CDCl_3$, δ_C): 139.3, 139.0, 137.9, 136.7, 125.9, 124.4, 124.0, 121.4, 121.1, 120.5, 120.4, 110.0, 109.4.

[Ir(dF-CF_3-ppy)$_2$(dtbpy)](PF_6)

The photocatalyst was synthesized according to a literature procedure.[4]

2.2 Synthesis of starting materials

General Procedure for the synthesis of 1,3-Dithianes (1) (General Procedure A)

1,3-Dithianes were synthesized according to a modified literature procedure.[5]

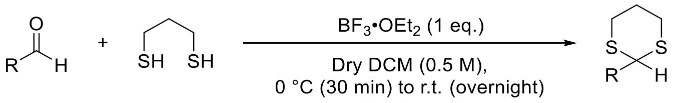

The corresponding aldehyde (1.0 eq.) was dissolved in dry DCM (0.5 M) and propane-1,3-dithiol (1.05 eq.) was added. The solution was cooled to 0 °C and BF_3 (48 wt% solution in OEt_2) (1 eq.) was introduced dropwise. After completed addition the mixture was stirred at the same temperature for 30 min, warmed to room temperature and further stirred overnight. The reaction mixture was then washed with brine (25 mL), followed by aq. sat. $NaHCO_3$ (2x25 mL) and brine (25 mL). The organic phase was dried over Na_2SO_4, the solvent removed under reduced pressure and the residue was purified by column automated flash column chromatography.

2-Hexyl-1,3-dithiane (1c)[6]

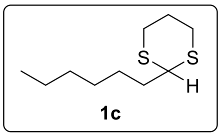

The titled compound was synthesized according to General Procedure A using *n*-heptanal (10 mmol) as starting material.

Column chromatography: PE/EtO_2 (0-5% EtO_2)

Yield: 87% (colorless liquid)

^{1}H-NMR (300 MHz, $CDCl_3$, δ_H): 4.04 (t, J = 6.9 Hz, 1H), 2.93-2.76 (m, 4H), 2.16-2.06 (m, 1H), 1.93-1.80 (m, 1H), 1.79-1.68 (m, 2H), 1.55-1.43 (m, 2H), 1.37-1.20 (m, 6H), 0.92-0.82 (m, 3H).

^{13}C-NMR (75 MHz, $CDCl_3$, δ_C): 47.8 (+), 35.6 (–), 31.7 (–), 30.7 (–), 29.0 (–), 26.7 (–), 26.2 (–), 22.7 (–), 14.2 (+).

HRMS (EI) (m/z): [M^+] ($C_{10}H_{20}S_2^+$) calc. 204.1001; observed 204.1001.

2-(2-(Methylthio)ethyl)-1,3-dithiane (1d)[7]

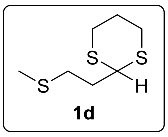

The titled compound was synthesized according to General Procedure A using 3-(methylthio)-propanal (10 mmol) as starting material.

Column chromatography: PE/EtOAc (20/1)

Yield: 91% (colorless liquid)

^{1}H-NMR (400 MHz, $CDCl_3$, δ_H): 4.18 (t, J = 7.0 Hz, 1H), 2.92-2.80 (m, 4H), 2.68 (t, J = 7.3 Hz, 2H), 2.15-2.07 (m, 4H), 2.03 (q, J = 7.1 Hz, 2H), 1.92-1.81 (m, 1H).

^{13}C-NMR (101 MHz, $CDCl_3$, δ_C): 46.0 (+), 34.7 (–), 31.2 (–), 30.3 (–), 26.1 (–), 15.6 (+).

HRMS (EI) (m/z): [M^+] ($C_7H_{14}S_3^+$) calc. 194.0252; observed 194.0255.

3-(1,3-Dithian-2-yl)propanenitrile (1e)[8]

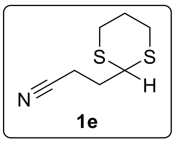

The titled compound was synthesized by following two-step procedure:

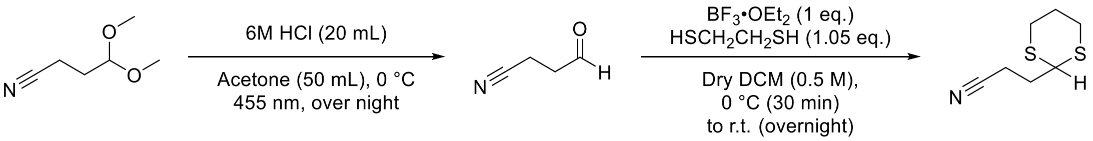

3-Cyanopropionaldehyde dimethyl acetal (1.3 mL, 10 mmol, 1 eq.) was dissolved in acetone (50 mL) under a N_2 atmosphere. The solution was cooled to 0 °C and half-concentrated HCl (6M, 20 mL) was added. The mixture was stirred for 2 h at 0 °C and then at r.t. overnight. The mixture was concentrated to approximately 5 mL and extracted with $CHCl_3$ (4x15 mL). The combined organic phases were dried over Na_2SO_4 and the solvent was removed under reduced pressure to obtain the crude aldehyde.[9]

The crude aldehyde (4.14 mmol) was used for the synthesis of **1e** according to General Procedure A.

Column chromatography: PE/EtOAc (10-20% EtOAc)

Yield (combined over two steps): 19% (colorless liquid)

^{1}H-NMR (300 MHz, $CDCl_3$, δ_H): 4.10 (t, J = 7.2 Hz, 1H), 2.92-2.83 (m, 4H), 2.60 (t, J = 7.2 Hz, 2H), 2.18-2.08 (m, 3H), 1.95-1.81 (m, 1H).

^{13}C-NMR (75 MHz, $CDCl_3$, δ_C): 118.8 (C_q), 45.2(+), 31.0 (–), 29.9 (–), 25.7 (–), 15.0 (–).

HRMS (EI) (m/z): [M^+] ($C_7H_{11}NS_2^+$) calc. 173.0327; observed 173.0329.

2-Phenethyl-1,3-dithiane (1f)[10]

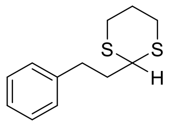

1f

The titled compound was synthesized according to General Procedure A using benzenepropanal (10 mmol) as starting material.

Column chromatography: PE/EtOAc (50/1)

Yield: 64% (colorless liquid)

^{1}H-NMR (400 MHz, $CDCl_3$, δ_H): 7.32-7.27 (m, 2H), 7.24-7.18 (m, 3H), 4.00 (t, J = 7.0 Hz, 1H), 2.90-2.81 (m, 6H), 2.16-2.05 (m, 3H), 1.94-1.83 (m, 1H).

^{13}C-NMR (101 MHz, $CDCl_3$, δ_C): 141.0, 128.6, 128.6, 126.2, 46.7, 37.1, 32.7, 30.4, 26.2.

HRMS (EI) (m/z): [M^+] ($C_{12}H_{16}S_2^+$): calc. 224.0688; observed 224.0693.

Ethyl 4-(2-(1,3-dithian-2-yl)ethyl)benzoate (1g)

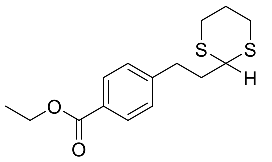

1g

The titled compound was synthesized by following two-step procedure:

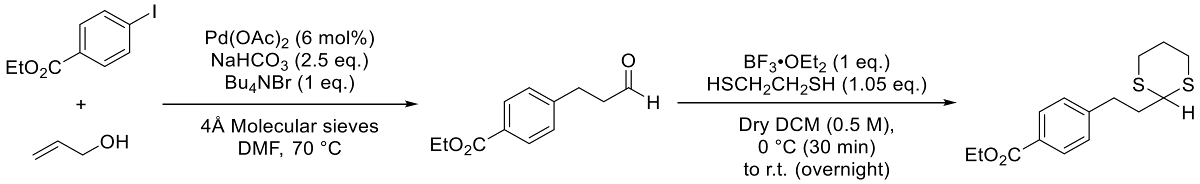

A Schlenk falsk was charged with $Pd(OAc)_2$ (101 mg, 450 µmol, 6 mol%), $NaHCO_3$ (1.58 g, 18.8 mmol, 2.5 eq.), Bu_4NBr (2.42 g, 7.5 mmol, 1 eq.) and 4 Å molecular sieves (1.2 g). The flask was set under a N_2-atmoshphere before adding dry DMF (15 mL), allyl alcohol (765 µL, 11.3 mmol, 1.5 eq.) and ethyl 4-iodobenzoate (1.26 mL, 7.5 mmol, 1 eq.). The mixture was stirred at 70 °C for 4 h. After cooling to room temperature, the mixture was filtered through a pad of Celite, which was washed with ethyl acetate. Water was added and the aqueous phase was extracted with ethyl acetate. The combined organic phases were washed with water and brine, dried over Na_2SO_4, filtered and concentrated under reduced pressure. The residue was purified by column chromatography (PE/EtOAc 20-40%) to yield ethyl-4-(3-oxopropyl)benzoate (489 mg, 2.37 mmol, 31%).[11]

Ethyl-4-(3-oxopropyl)benzoate was subjected to General Procedure A to obtain the titled compound.

Column chromatography: PE/EtOAc (5-10% EtOAc)

Yield (combined over two steps): 20% (colorless highly viscous liquid)

^{1}H-NMR (400 MHz, $CDCl_3$, δ_H): 7.92 (d, *J* = 8.2 Hz, 2H), 7.23 (d, *J* = 8.2 Hz, 2H), 4.32 (q, *J* = 7.1 Hz, 2H), 3.91 (t, *J* = 7.0 Hz, 1H), 2.88-2.73 (m, 6H), 2.09-1.99 (m, 3H), 1.88-1.76 (m, 1H), 1.34 (t, *J* = 7.1 Hz, 3H).

^{13}C-NMR (101 MHz, $CDCl_3$, δ_C): 166.4 (C_q), 146.2 (C_q), 129.7 (+), 128.4 (+), 128.4 (C_q), 60.7 (–), 46.3 (+), 36.5 (–), 32.4 (–), 30.1 (–), 25.9 (–), 14.3 (+).

HRMS (EI) (m/z): [M^+] ($C_{15}H_{20}O_2S_2^+$) calc. 296.0899; observed 296.0896.

2-(4-Chlorophenethyl)-1,3-dithiane (1h)

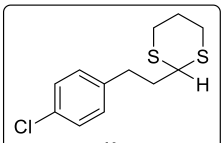

1h

The titled compound was synthesized by following two-step procedure:

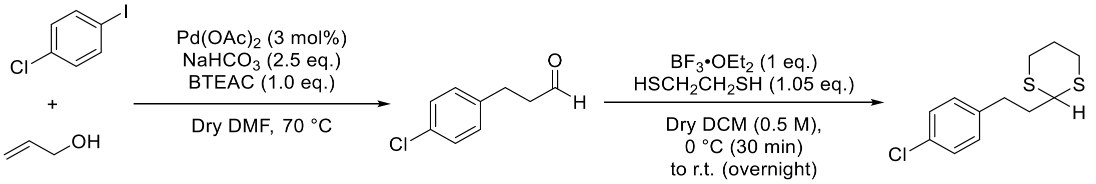

A Schlenk falsk was charged with $Pd(OAc)_2$ (26.6 mg, 118.3 μmol, 3 mol%), $NaHCO_3$ (828 mg, 9.87 mmol, 2.5 eq.), benzyltriethylammonium chloride (BTEAC) (898 mg, 3.94 mmol, 1 eq.) and 1-chloro-4-iodobenzene (940 mg, 3.94 mmol, 1 eq.). The flask was set under a N_2-atmoshphere before adding dry DMF (20 mL) and allyl alcohol (404 μL, 5.91 mmol, 1.5 eq.). The mixture was stirred at 50 °C overnight and after cooling to room temperature filtered through a pad of Celite, which was washed with ethyl acetate (80 mL). The filtrate was washed with water (2x30 mL) and the organic phase was dried over Na_2SO_4. The solvent was evaporated under reduced pressure and the residue purified by column chromatography (PE/EtOAc 5-20%) to yield 3-(4-chlorophenyl)propanal (588 mg, 3.49 mmol, 88%) (still shows impurity).[12]

3-(4-Chlorophenyl)propanal was subjected to General Procedure A to obtain the titled compound.

Column chromatography: PE/EtO_2 (2-5% EtO_2)

Yield (combined over two steps): 47% (colorless liquid)

^{1}H-NMR (300 MHz, $CDCl_3$, δ_H): 7.28-7.22 (m, 2H), 7.17-7.11 (m, 2H), 3.94 (t, *J* = 7.0 Hz, 1H), 2.90-2.75 (m, 6H), 2.16-1.98 (m, 3H), 1.94-1.79 (m, 1H).

^{13}C-NMR (75 MHz, $CDCl_3$, δ_C): 139.4 (C_q), 131.9 (C_q), 130.0 (+), 128.6 (+), 46.4 (+), 36.8 (–), 31.9 (–), 30.3 (–), 26.1 (–).

HRMS (EI) (m/z): [M^+] ($C_{12}H_{15}ClS_2^+$) calc. 258.0298; observed 258.0294.

2-(4-Bromophenethyl)-1,3-dithiane (1i)

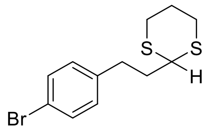

1i

The titled compound was synthesized by following two-step procedure:

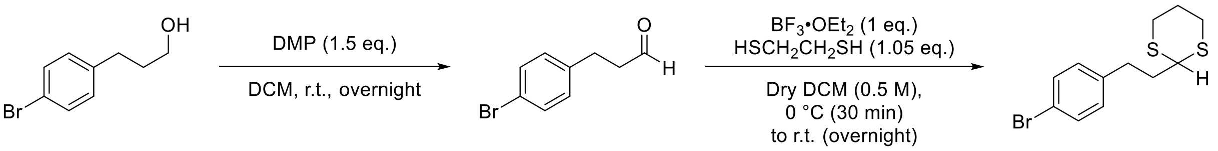

3-(4-Bromophenyl)propan-1-ol (4 mmol, 860 mg, 1 eq.) was dissolved in DCM (30 mL) under a N_2-atmosphere. Dess-Martin periodane (DMP) (4.8 mmol, 2.04 g, 1.2 eq.) was slowly added to this solution and the resulting reaction mixture was stirred overnight. Afterwards, the mixture was filtered through a pad of celite, washed with DCM and the filtrate was concentrated under reduced pressure. The residue was purified by column chromatography (PE/EtOAc 5-15%) to yield 3-(4-bromophenyl)propanal (638 mg, 2.99 mmol, 75%) (still shows impurity).[13]

3-(4-Bromophenyl)propanal was subjected to General Procedure A to obtain the titled compound.

Column chromatography: PE/EtOAc (2-10% EtOAc)

Yield (combined over two steps): 47% (colorless liquid)

^{1}H-NMR (300 MHz, $CDCl_3$, δ_H): 7.41-7.36 (m, 2H), 7.11-7.05 (m, 2H), 3.93 (t, J = 7.0 Hz, 1H), 2.89-2.73 (m, 6H), 2.15-1.97 (m, 3H), 1.93-1.77 (m, 1H).

^{13}C-NMR (75 MHz, $CDCl_3$, δ_C): 139.8 (C_q), 131.5 (C_q), 130.3 (+), 119.9 (+), 46.3 (+), 36.7 (–), 31.9 (–), 30.3 (–), 26.0 (–).

HRMS (EI) (m/z): [M^+] ($C_{12}H_{15}BrS_2^+$) calc. 301.9793; observed 301.9791.

2-(3,5-Dichlorophenethyl)-1,3-dithiane (1j)

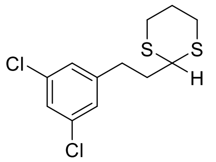

1j

The titled compound was synthesized by following two-step procedure:

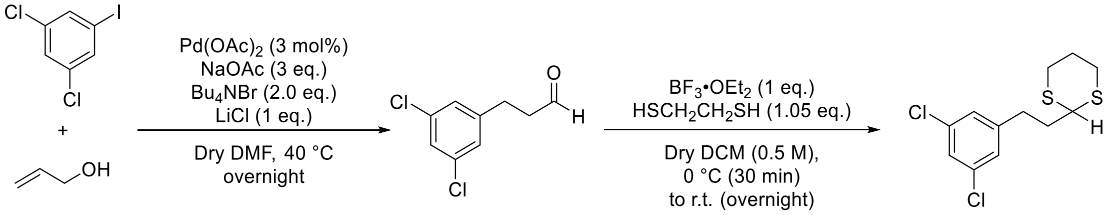

A Schlenk falsk was charged with $Pd(OAc)_2$ (33.7 mg, 150 µmol, 3 mol%), NaOAc (1.23 g, 15.0 mmol, 3 eq.), Bu_4NBr (3.22 g, 10.0 mmol, 2 eq.), LiCl (212 mg, 5.0 mmol, 1 eq.) and 1-iodo-3,5-dichlorobenzene (1.36 g, 5.0 mmol, 1 eq.). The flask was set under a N_2-atmoshphere before adding dry DMF (20 mL) and allyl alcohol (444 µL, 6.5 mmol, 1.3 eq.). The mixture was stirred at 40 °C overnight and after cooling to room temperature filtered through a pad of Celite, which was washed with ethyl acetate (80 mL). The filtrate was washed with water (2x30 mL) followed by brine (20 mL) and the organic phase was dried over Na_2SO_4. The solvent was evaporated under reduced pressure and the residue purified by column chromatography (PE/EtOAc 2-10%) to yield 3-(3,5-Dichlorophenyl)propanal (501 mg, 2.47 mmol, 49%) (still shows impurity).[14]

3-(3,5-Dichlorophenyl)propanal was subjected to General Procedure A to obtain the titled compound.

Column chromatography: PE/EtO_2 (2-5% EtO_2)

Yield (combined over two steps): 24% (colorless liquid) (93 wt% purity determined by NMR with trimethoxybenzene as standard)

^{1}H-NMR (300 MHz, $CDCl_3$, δ_H): 7.21 (t, J = 1.9 Hz, 1H), 7.10 (d, J = 1.9 Hz, 1H), 2.88-2.75 (m, 6H), 2.18-1.99 (m, 3H), 1.95-1.82 (m, 1H).

^{13}C-NMR (75 MHz, $CDCl_3$, δ_C): 144.3 (C_q), 134.9 (C_q), 127.2 (+), 126.5 (+), 46.3 (+), 36.4 (–), 32.1 (–), 30.3 (–), 26.0 (–).

HRMS (EI) (m/z): [M^+] ($C_{12}H_{14}Cl_2S_2^+$) calc. 291.9909; observed 291.9901.

2-Cyclohexyl-1,3-dithiane (1k)[15]

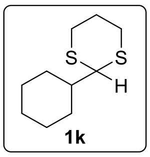

The titled compound was synthesized according to General Procedure A using Cyclohexanecarboxaldehyde (10 mmol) as starting material.

Column chromatography: PE/EtOAc (20:1)

Yield: 83% (white solid)

^{1}H-NMR (400 MHz, $CDCl_3$, δ_H): 4.03 (d, *J* = 5.4 Hz, 1H), 2.92-2.80 (m, 4H), 2.13-2.05 (m, 1H), 1.94-1.59 (m, 7H), 1.31-1.06 (m, 5H).

^{13}C-NMR (101 MHz, $CDCl_3$, δ_H): 55.5, 43.2, 31.1, 30.6, 26.6, 26.4, 26.3.

HRMS (EI) (m/z): [M^+] ($C_{10}H_{18}S_2^+$) calc. 202.0844; observed 202.0847.

2-(4-Methoxyphenyl)-1,3-dithiane (1m)[6]

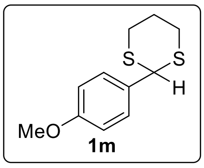

The titled compound was synthesized according to General Procedure A using 4-methoxybenzaldehyde (10 mmol) as starting material.

Column chromatography: PE/EtOAc (20:1)

Yield: 96% (white solid)

^{1}H-NMR (400 MHz, $CDCl_3$, δ_H): 7.39 (d, *J* = 8.8 Hz, 2H), 6.86 (d, *J* = 8.8 Hz, 2H), 5.13 (s, 1H), 3.80 (s, 3H), 3.10-3.01 (m, 2H), 2.94-2.86 (m, 2H), 2.21-2.11 (m, 1H), 1.99-1.86 (m, 1H).

^{13}C-NMR (101 MHz, $CDCl_3$, δ_H): 159.7, 131.5, 129.1, 114.2, 55.4, 50.9, 32.4, 25.2.

HRMS (APCI) (m/z): [$M+H^+$] ($C_{11}H_{14}OS_2^+$) calc. 226.0481; observed 226.0478.

2,2'-(3-methylbutane-1,3-diyl)bis(1,3-dithiane) (1n)

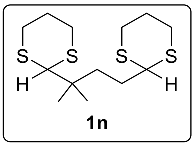

The titled compound was synthesized in following three-step-procedure:

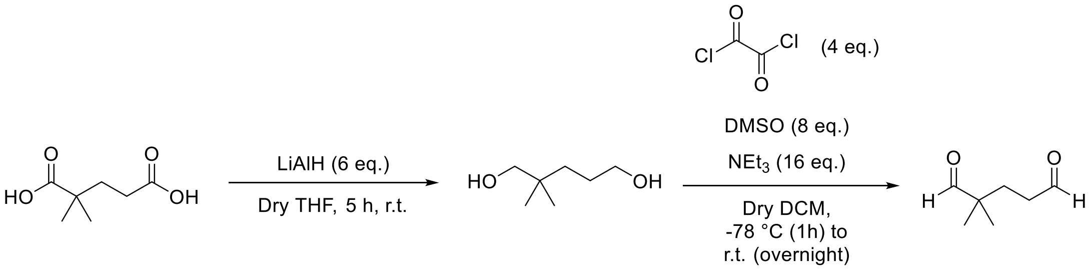

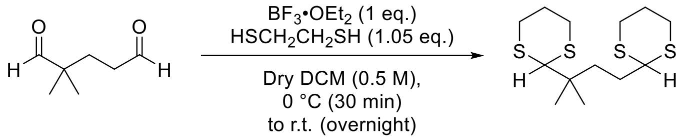

The diacid was reduced according to a modified literature procedure.[16] Lithium aluminum hydride (2.10 g, 60.0 mmol, 6 eq.) was added to a solution of 2,2-dimethylpentanedioic acid (1.60 g, 10.0 mmol, 1 eq.) in dry THF (120 mL). The reaction mixture was stirred at room temperature for 5 h and was afterwards quenched by the addition of H_2O (150 mL). The mixture was transferred to a separation funnel and 2M HCl (200 mL) together with EtOAc (50 mL) were added. The phases were separated and the water phase was extracted with Et_2O (3x50 mL). The combined organic phases were washed with aq. sat. $NaHCO_3$ (2x100 mL) followed by brine (50 mL) and then dried over Na_2SO_4. The drying agent was filtered, the solvent was evaporated under reduced pressure and the crude product (791 mg, 5.98 mmol) was used for the next step without further purification.

The crude diol was oxidized according to a modified literature procedure.[17] Oxalyl chloride (2.05 mL, 23.9 mmol, 4 eq.) was dissolved in dry DCM (50 mL) under a N_2-atmosphere and the solution was cooled to -78 °C. Dry DMSO (3.40 mL, 47.8 mmol, 8 eq.) was slowly added over 5 min. After completed addition the mixture was stirred for 20 min at -78 °C and a solution of the crude diol (791 mg, 5.98 mmol, 1 eq.) in dry DCM (10 mL) was slowly added over 5 min. The mixture was further stirred at -78 °C for 1 h after which NEt_3 (13.3 mL, 95.6 mmol, 16 eq.) was added. The temperature of the reaction mixture was gradually raised to r.t. over 2 h and afterwards stirred at r.t. overnight. After the given time, Et_2O (75 mL) and H_2O (25 mL) were added and the phases were separated. The organic phase was washed with H_2O (25 mL), followed by 2M HCl (3x25 mL), sat. aq. $NaHCO_3$ (3x25 mL) and brine (3x25 mL) and afterwards dried over Na_2SO_4. The solvent was evaporated to yield the crude dialdehyde product (668 mg, 5.21 mmol).

The obtained crude dialdehyde was subjected to General Procedure A to obtain the titled compound.

Column chromatography: PE/EtO_2 (5-15% EtO_2)

Yield (combined over three steps): 9% (colorless highly viscous liquid)

^{1}H-NMR (300 MHz, $CDCl_3$, δ_H): 4.00-3.94 (m, 2H), 2.93-2.76 (m, 8H), 2.15-2.00 (m, 2H), 1.93-1.63 (m, 6H), 1.07 (s, 6H).

^{13}C-NMR (75 MHz, $CDCl_3$, δ_C): 60.4 (+), 48.2 (+), 38.1 (C_q), 37.7 (–), 31.5 (–), 30.6 (–), 30.1 (–), 26.3 (–), 26.2 (–), 25.4 (+).

HRMS (EI) (m/z): [M^+] ($C_{13}H_{24}S_4^+$) calc. 308.0755; observed 308.0753.

2-(4-(2-(1,3-Dithian-2-yl)ethyl)phenyl)-1,3-dithiane (1o)

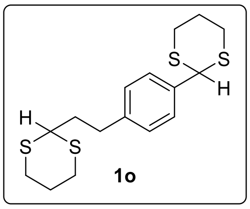

The titled compound was synthesized in following three-step-procedure:

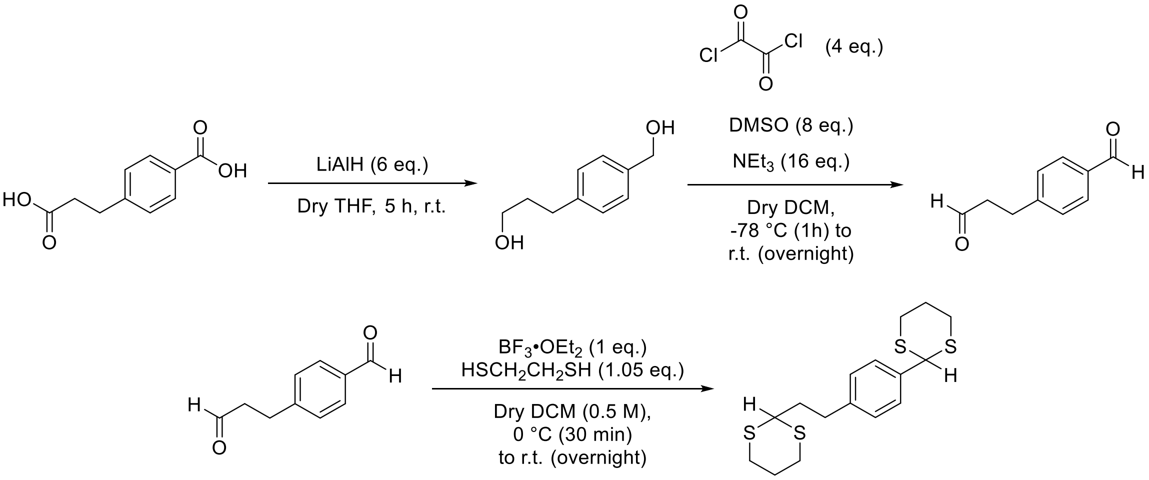

The diacid was reduced according to a modified literature procedure.[16] Lithium aluminum hydride (3.14 g, 90.0 mmol, 6 eq.) was added to a solution of 4-carboxy-benzene-propanoic acid (2.91 g, 15.0 mmol, 1 eq.) in dry THF (120 mL). The reaction mixture was stirred at room temperature for 5 h and was afterwards quenched by the addition of H_2O (150 mL). The mixture was transferred to a separation funnel and 2M HCl (200 mL) together with EtOAc (50 mL) were added. The phases were separated and the water phase was extracted with Et_2O (3x50 mL). The combined organic phases were washed with aq. sat. $NaHCO_3$ (2x100 mL), followed by brine (50 mL) and then dried over Na_2SO_4. The drying agent was filtered, the solvent was evaporated under reduced pressure and the crude product was purified by column chromatography (PE/EtOAc 50-70%) to obtain the corresponding diol (1.40 g, 8.42 mmol, 56%).

The diol was oxidized according to a modified literature procedure.[17] Oxalyl chloride (2.90 mL, 33.7 mmol, 4 eq.) was dissolved in dry DCM (60 mL) under a N_2-atmosphere and the solution was cooled to -78 °C. Dry DMSO (4.80 mL, 67.4 mmol, 8 eq.) was slowly added over 10 min. After completed addition, the mixture was stirred for 30 min at -78 °C and a solution of the diol (1.40 g, 8.42 mmol, 1 eq.) in dry DCM (15 mL) was slowly added over 10 min. The mixture was further stirred at -78 °C for 1.5 h after which NEt_3 (18.8 mL, 135 mmol, 16 eq.) was added. The temperature of the reaction mixture was gradually raised to r.t. over 2 h and afterwards stirred at r.t. overnight. After the given time, Et_2O (100 mL) and H_2O (40 mL) were added and the phases were separated. The organic phase was washed with H_2O (40 mL), followed by 2M HCl (3x40 mL), sat. aq. $NaHCO_3$ (3x40 mL) and brine (3x25 mL) and afterwards dried over Na_2SO_4. The solvent was evaporated to yield the crude dialdehyde product (1.45 g, 8.94 mmol, 106%, contains impurities).

The obtained crude dialdehyde was subjected to General Procedure A without further purification and with 8.42 mmol being set to 1 eq. to obtain the titled compound. Instead of a column chromatography, the crude product after reaction and evaporation of solvent was purified by recrystallization from EtOAc/PE. Alternatively, the crude product can be purified by vaper diffusion from $CHCl_3$/PE as well.

Yield (combined over three steps): 29% (white solid)

^{1}H-NMR (300 MHz, $CDCl_3$, δ_H): 7.38 (d, J = 8.2 Hz, 2H), 7.18 (d, J = 8.1 Hz, 2H), 5.15 (s, 1H), 3.96 (t, J = 7.0 Hz, 1H), 3.12-2.99 (m, 2H), 2.96-2.76 (m, 8H), 2.22-1.78 (m, 6H),

^{13}C-NMR (75 MHz, $CDCl_3$, δ_C): 141.3 (C_q), 137.0 (C_q), 129.0 (+), 127.9 (+), 51.3 (+), 46.5(+), 36.8 (–), 32.3 (–), 30.3 (–), 26.1 (–), 25.2 (–).

HRMS (EI) (m/z): [M^+] ($C_{16}H_{22}S_4^+$) calc. 342.0599; observed 342.0599.

4-(4-Bromophenyl)butan-2-one (2i)[18]

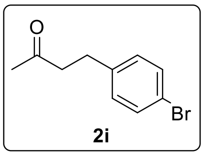

The compound was synthesized according to a modified literature procedure.[18]

K_2CO_3 (1.26 g, 9.09 mmol, 1 eq.) was added to a solution of acetylacetone (0.93 mL, 9.09 mmol, 1 eq.) in MeOH (15 mL). 4-Bromobenzyl bromide (2.5 g, 10.0 mmol. 1.1 eq.) was added and the reaction mixture was stirred under reflux for 24 h. After completed reaction time, the mixture was diluted with H_2O and extracted three times with EtOAc. The combined organic phases were washed with brine, dried over $MgSO_4$ and concentrated under reduced pressure. The residue was purified by column chromatography (PE/EtOAc 9:1) to afford the titled compound.

Yield: 48% (colorless liquid)

^{1}H-NMR (400 MHz, $CDCl_3$, δ_H): 7.39 (d, J = 8.3 Hz, 2H), 7.06 (d, J = 8.3 Hz, 2H), 2.87-2.81 (m, 2H), 2.76-2.70 (m, 2H), 2.13 (s, 3H).

^{13}C-NMR (101 MHz, $CDCl_3$, δ_C): 207.6, 140.2, 131.7, 130.3, 120.0, 45.0, 30.2, 29.2.

4-(4-(4,4,5,5-tetramethyl-1,3,2-dioxaborolan-2-yl)phenyl)butan-2-one (2j)[19]

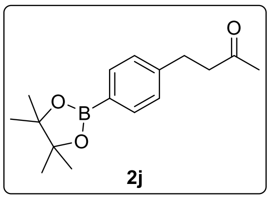

The compound was synthesized according to a modified literature procedure.[19]

A 20 mL crimp cap vial was charged with Pd(dppf)Cl_2 (22.0 mg, 30 µmol, 3 mol%), KOAc (294.4 mg, 3 mmol, 3 eq.), B_2pin_2 (304.7 mg, 1.2 mmol, 1.2 eq.) and 4-(4-Bromophenyl)butan-2-one (**2i**) (168.6 µL, 1 mmol, 1 eq.). The vial was set under a N_2-atmosphere, DMF (6 mL) was added and the reaction mixture was stirred at 95 °C overnight. After cooling to room temperature, H_2O (15 mL) and EtOAc (10 mL) were added. The phases were separated and the water phase was extracted with EtOAc (2x10 mL). The combined organic phases were washed with H_2O (3x10 ml) followed by brine (3x10 mL) and dried over $MgSO_4$. The solvent was removed under reduced pressure and the residue was purified by column chromatography (PE/EtOAc 5:1) to yield the titled compound.

Yield: 71% (colorless liquid)

^{1}H-NMR (400 MHz, $CDCl_3$, δ_H): 7.73 (d, J = 7.8 Hz, 2H), 7.19 (d, J = 7.9 Hz, 2H), 2.93-2.87 (m, 2H), 2.86-2.78 (m, 2H), 2.13 (s, 3H), 1.33 (s, 12H).

^{13}C-NMR (101 MHz, $CDCl_3$, δ_C): 207.9, 144.5, 135.2, 127.9, 83.8, 45.1, 30.2, 30.1, 25.0.

4-(3-Oxobutyl)phenyl acetate (2k)[20]

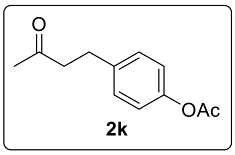

The compound was synthesized according to a modified literature procedure.[20]

Acetyl chloride (393 mL, 5.50 mmol, 1.1 eq.) was added dropwise to a mixture of 4-(4-hydroxyphenyl)butan-2-one (821 mg, 5.00 mmol, 1.0 eq.) and triethylamine (2.09 mL, 15.0 mmol, 3.0 eq.) in dry DCM and the mixture was stirred at room temperature overnight. The mixture was poured into 100 mL ice-water and the organic phase was washed with $NaHCO_3$ (aq., 5 wt%, 50 mL) followed by H_2O (50 mL), dried over Na_2SO_4 and filtered. The solvent was evaporated and the residue purified by column chromatography (PE/EtOAc 15-30%) to afford the titled compound.

Yield: 73% (colorless liquid)

^{1}H-NMR (400 MHz, $CDCl_3$, δ_H): 7.18 (d, J = 8.5 Hz, 2H), 6.98 (d, J = 8.5 Hz, 2H), 2.88 (t, J = 7.5 Hz, 2H), 2.75 (t, J = 7.5 Hz, 2H), 2.28 (s, 3H), 2.14 (s, 3H).

^{13}**C-NMR** (101 MHz, $CDCl_3$, δ_C): 207.8 (C_q), 169.7 (C_q), 149.1 (C_q), 138.7 (C_q), 129.4 (+), 121.6 (+), 45.2 (–), 30.3 (+), 29.2 (–), 21.2 (+).

HRMS (EI) (m/z): [M^+] ($C_{12}H_{14}O_3^+$) calc. 206.0938; observed 206.0934.

3. Photocatalytic base-free Corey-Seebach reaction

General procedure for the photocatalytic base-free Corey-Seebach Reaction (General Procedure B)

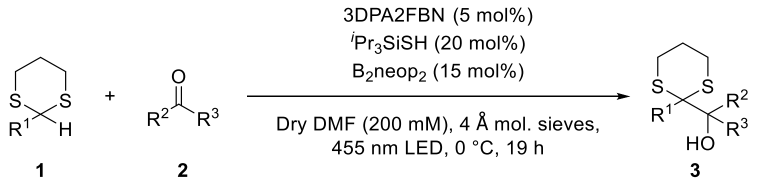

A 5 mL crimp cap vial equipped with a magnetic stirring bar was loaded with 3DPA2FBN (3.8 mg, 6.0 µmol, 3 mol%), bis(neopentyl glycolato)diboron (B_2neop_2) (6.8 mg, 40 µmol, 15 mol%), 4 Å molecular sieves (50 mg), the corresponding 1,3-dithiane (200 µmol, 1 eq.), *i*Pr_3SiSH (4.3 µL, 20 µmol, 10 mol%), the corresponding aldehyde or ketone (equivalents as noted in the tables or text) and dry DMF (1 mL). In doing so, all solid compounds were added before capping the vial, whereas all liquid compounds were added *via* syringe after setting the capped vial under a N_2-atmosphere (highly viscous liquids were added before capping the vial as well). The reaction mixture was degassed by three cycles of freeze-pump-thaw and subsequently stirred under light irradiation using a 455 nm (± 15 nm) LED for 4 h at 0 °C using a cryostat. After this time, a solution of 3DPA2FBN in dry DMF (20 mM, 200 µL, 2 mol%) degassed by three cycles of freeze pump-thaw and *i*Pr_3SiSH (4.3 µL, 20 µmol, 10 mol%) were added to the reaction mixture and it was further stirred at 0 °C overnight (total reaction time approx. 19 h).

Two reaction batches were combined, filtered and diluted with brine (10 mL), water (10 mL) and ethyl acetate (15 mL). The phases were separated, the water phase was extracted with ethyl acetate (3x7 mL) and the combined organic phase dried over Na_2SO_4. The solvent was removed under reduced pressure and the crude product was purified by automated flash column chromatography (see specific compounds for the used solvent mixtures).

If specified in the table or text, the unreacted starting material was re-isolated during the purification.

If an aldehyde was used as electrophile, the reaction was performed in absence of bis(neopentyl glycolato)diboron.

2-(2-Methyl-1,3-dithian-2-yl)propan-2-ol (3aa)[21]

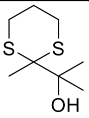

3aa

Equivalents: 1 eq. **1a**, 10 eq. **2a**

Column chromatography: DCM (for 1 CV) followed by PE/EtOAc (0-20% EtOAc gradient)

Yield: 65% (slightly yellow liquid)

^{1}H-NMR (300 MHz, $CDCl_3$, δ_H): 2.99-2.79 (m, 4H), 2.45 (s, 1H), 2.07-1.81 (m, 2H), 1.78 (s, 3H), 1.41 (s, 6H).

^{13}C-NMR (75 MHz, $CDCl_3$, δ_C): 77.0 (C_q), 60.6 (C_q), 26.9 (+), 25.4 (–), 25.0 (–), 24.8 (+).

HRMS (EI) (m/z): [M^+] ($C_8H_{16}OS_2^+$) calc. 192.0637; observed 192.0639.

2-(1,3-Dithiane-2-yl)propan-2-ol (3ba)[22]

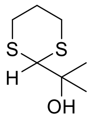

3ba

Equivalents: 1 eq. **1b**, 10 eq. **2a**

Column chromatography: DCM (for 2 CV) followed by PE/EtOAc (0-20% EtOAc gradient)

Yield: 30% (slightly yellow liquid)

^{1}H-NMR (300 MHz, $CDCl_3$, δ_H): 4.16 (s, 1H), 2.98-2.82 (m, 4H), 2.32 (s, 1H), 2.15-2.05 (m, 1H), 1.92-1.78 (m, 1H), 1.38 (s, 6H).

^{13}C-NMR (75 MHz, $CDCl_3$, δ_C): 73.3 (C_q), 61.0 (+), 30.8 (–), 27.3 (+), 25.8 (–).

HRMS (EI) (m/z): [M^+] ($C_7H_{14}OS_2^+$) calc. 178.0481; observed 178.0485.

2-(2-Hexyl-1,3-dithian-2-yl)propan-2-ol (3ca)

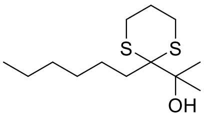

3ca

Equivalents: 1 eq. **1c**, 10 eq. **2a**

Column chromatography: PE/EtO_2 (0-20% EtO_2 gradient)

Yield: 39% (slightly yellow liquid) (32% Recovered starting material; isolated)

^{1}H-NMR (300 MHz, $CDCl_3$, δ_H): 2.97-2.74 (m, 4H), 2.37 (s, 1H), 1.96-1.84 (m, 4H), 1.71-1.59 (m, 2H), 1.40 (s, 6H), 1.33-1.23 (m, 6H), 0.92-0.83 (m, 3H).

^{13}C-NMR (75 MHz, $CDCl_3$, δ_C): 79.0 (C_q), 64.2 (C_q), 38.4 (–), 31.9 (–), 30.2 (–), 27.3 (–), 26.6 (–), 26.4 (+), 23.8 (–), 22.8 (–), 14.2 (+).

HRMS (APCI) (m/z): [M+H$^+$] ($C_{13}H_{27}OS_2^+$) calc. 263.1498; observed 263.1496.

2-(2-(2-(Methylthio)ethyl)-1,3-dithian-2-yl)propan-2-ol (3da)

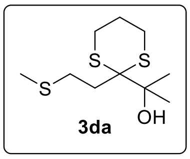

Equivalents: 1 eq. **1d**, 10 eq. **2a**

Column chromatography: DCM (for 2 CV) followed by PE/EtOAc (0-20% EtOAc gradient)

Yield: 53% (slightly yellow liquid) (24% Recovered starting material; isolated)

^{1}H-NMR (300 MHz, $CDCl_3$, δ_H): 2.94-2.74 (m, 6H), 2.39 (s, 1H), 2.23-2.16 (m, 2H), 2.11 (s, 3H), 1.92 (p, *J* = 5.9 Hz, 2H), 1.41 (s, 6H).

^{13}C-NMR (75 MHz, $CDCl_3$, δ_C): 78.8 (C_q), 62.9 (C_q), 37.7 (–), 31.3 (–), 27.2 (–), 26.4 (+), 23.8 (–), 15.8 (+).

HRMS (APCI) (m/z): [M+H$^+$] ($C_{10}H_{21}OS_3^+$) calc. 235.0643; observed 235.0649.

3-(2-(2-Hydroxypropan-2-yl)-1,3-dithian-2-yl)propanenitrile (3ea)

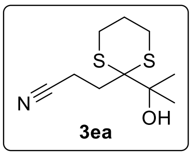

Equivalents: 1 eq. **1e**, 10 eq. **2a**

Column chromatography: DCM (for 2 CV) followed by PE/EtOAc (15-25% EtOAc gradient)

Yield: 44% (colorless highly viscous liquid) (31% Recovered starting material; isolated)

^{1}H-NMR (300 MHz, $CDCl_3$, δ_H): 2.94-2.74 (m, 6H), 2.35-2.28 (m, 2H), 2.21 (s, 1H), 1.93 (p, *J* = 6.0 Hz, 2H), 1.44 (s, 6H).

^{13}C-NMR (75 MHz, $CDCl_3$, δ_C): 120.5 (C_q), 79.0 (C_q), 61.1 (C_q), 33.0 (–), 27.1 (–), 26.5 (+), 23.7 (–), 14.9 (–).

HRMS (APCI) (m/z): [M+H$^+$] ($C_{10}H_{18}NOS_2^+$) calc. 232.0824; observed 232.0828.

2-(2-Phenethyl-1,3-dithian-2-yl)propan-2-ol (3fa)

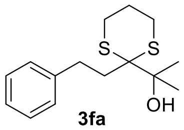

3fa

Equivalents: 1 eq. **1f**, 10 eq. **2a**

Column chromatography: DCM (for 1.5 CV) followed by PE/EtOAc (0-15% EtOAc gradient)

Yield: 54% (slightly yellow highly viscous liquid) (21% Recovered starting material; isolated)

^{1}H-NMR (400 MHz, $CDCl_3$, δ_H): 7.32-7.27 (m, 2H), 7.25-7.17 (m, 3H), 3.07-2.94 (m, 4H), 2.90-2.82 (m, 2H), 2.51 (bs, 1H), 2.21-2.15 (m, 2H), 1.97 (p, *J* = 6.3 Hz, 2H), 1.47 (s, 6H).

^{13}C-NMR (101 MHz, $CDCl_3$, δ_C): 142.7 (C_q), 128.6 (+), 128.5 (+), 125.9 (+), 79.1 (C_q), 63.6 (C_q), 40.5 (–), 33.2 (–), 27.3 (–), 26.4 (+), 23.7 (–).

HRMS (APCI) (m/z): [M+H$^+$] ($C_{15}H_{23}OS_2^+$) calc. 283.1185; observed 283.1186.

Ethyl 4-(2-(2-(2-hydroxypropan-2-yl)-1,3-dithian-2-yl)ethyl)benzoate (3ga)

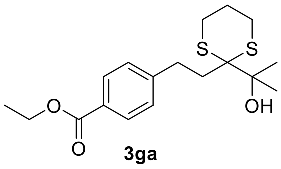

3ga

Equivalents: 1 eq. **1g**, 10 eq. **2a**

Column chromatography: DCM (for 1.5 CV) followed by PE/EtOAc (0-20% EtOAc gradient)

Yield: 54% (slightly yellow highly viscous liquid) (18% Recovered starting material; isolated)

^{1}H-NMR (300 MHz, $CDCl_3$, δ_H): 7.95 (d, *J* = 8.3 Hz, 2H), 7.26 (d, *J* = 8.4 Hz, 2H), 4.34 (q, *J* = 7.1 Hz, 2H), 3.11-3.03 (m, 2H), 3.01-2.90 (m, 2H), 2.86-2.76 (m, 2H), 2.54 (s, 1H), 2.17-2.10 (m, 2H), 1.94 (p, *J* = 6.2 Hz, 2H), 1.44 (s, 6H), 1.37 (t, *J* = 7.1 Hz, 3H).

^{13}C-NMR (75 MHz, $CDCl_3$, δ_C): 166.7 (C_q), 148.2 (C_q), 129.8 (+), 128.6 (+), 128.2 (C_q), 79.1 (C_q), 63.2 (C_q), 60.9 (–), 39.9 (–), 33.2 (–), 27.3 (–), 26.4 (+), 23.6 (–), 14.4 (+).

HRMS (APCI) (m/z): [M+H$^+$] ($C_{18}H_{27}O_3S_2^+$) calc. 355.1396; observed 355.1397.

2-(2-(4-Chlorophenethyl)-1,3-dithian-2-yl)propan-2-ol (3ha)

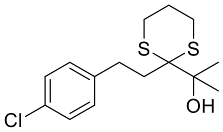

3ha

Equivalents: 1 eq. **h**, 10 eq. **2a**

Column chromatography: DCM (for 1.5 CV) followed by PE/EtOAc (0-10% EtOAc gradient)

Yield: 46% (Turbid white highly viscous liquid) (18% Recovered starting material; isolated)

^{1}H-NMR (300 MHz, $CDCl_3$, δ_H): 7.27-7.22 (m, 2H), 7.16-7.11 (m, 2H), 3.03-2.92 (m, 4H), 2.87-2.77 (m, 2H), 2.45 (s, 1H), 2.15-2.08 (m, 2H), 2.01-1.90 (m, 2H), 1.45 (s, 6H).

^{13}C-NMR (75 MHz, $CDCl_3$, δ_C): 141.2 (C_q), 131.6 (C_q), 130.0 (+), 128.6 (+), 79.1 (C_q), 63.3 (C_q), 40.4 (–), 32.5 (–), 27.4 (–), 26.4 (+), 23.7 (–).

HRMS (ESI) (m/z): [M+Na$^+$] ($C_{15}H_{21}ClOS_2Na^+$) calc. 339.0615; observed 339.0616.

2-(2-(4-Bromophenethyl)-1,3-dithian-2-yl)propan-2-ol (3ia)

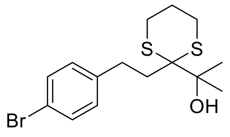

3ia

Equivalents: 1 eq. **1i**, 10 eq. **2a**

Column chromatography: DCM (for 1.5 CV) followed by PE/EtOAc (0-15% EtOAc gradient)

Yield: 38% (Slightly yellow highly viscous liquid) (25% Recovered starting material; isolated)

^{1}H-NMR (400 MHz, $CDCl_3$, δ_H): 7.41-7.37 (m, 2H), 7.10-7.06 (m, 2H), 3.01-2.93 (m, 4H), 2.86-2.78 (m, 2H), 2.47 (bs, 1H), 2.14-2.08 (m, 2H), 1.99-1.92 (m, 2H), 1.45 (s, 6H).

^{13}C-NMR (101 MHz, $CDCl_3$, δ_C): 141.7 (C_q), 131.6 (+), 130.4 (+), 119.7 (C_q), 79.1 (C_q), 63.3 (C_q), 40.3 (–), 32.6 (–), 27.4 (–), 26.5 (+), 23.7 (–).

HRMS (APCI) (m/z): [M+H$^+$] ($C_{15}H_{22}BrOS_2^+$) calc. 361.0290; observed 361.0294.

2-(2-(3,5-Dichlorophenethyl)-1,3-dithian-2-yl)propan-2-ol (3ja)

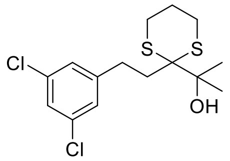

3ja

Equivalents: 1 eq. **1j**, 10 eq. **2a**

Column chromatography: DCM (for 1.5 CV) followed by PE/EtOAc (0-10% EtOAc gradient)

Yield: 26% (Slightly yellow highly viscous liquid) (50% Recovered starting material; isolated)

^{1}H-NMR (400 MHz, $CDCl_3$, δ_H): 7.18 (t, J = 1.9 Hz, 1H), 7.09 (d, J = 1.9 Hz, 2H), 3.03-2.93 (m, 4H), 2.85-2.77 (m, 2H), 2.34 (bs, 1H), 2.14-2.07 (m, 2H), 2.02-1.91 (m, 2H), 1.45 (s, 6H).

^{13}C-NMR (101 MHz, $CDCl_3$, δ_C): 146.2 (C_q), 134.9 (C_q), 127.2 (+), 126.2 (+), 79.2 (C_q), 63.0 (C_q), 39.9 (–), 32.7 (–), 27.5 (–), 26.5 (+), 23.7 (–).

HRMS (ESI) (m/z): [M+H$^+$] ($C_{15}H_{20}Cl_2OS_2Na^+$) calc. 373.0225; observed 373.0234.

2-(2-(4-Methoxyphenyl)-1,3-dithian-2-yl)propan-2-ol (3ma)

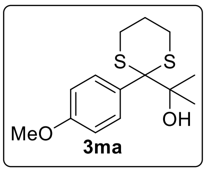

Equivalents: 1 eq. **1m**, 10 eq. **2a**

Column chromatography: First column: DCM (for 1.5 CV) followed by PE/EtOAc (0-25% EtOAc gradient). Second column: DCM/EtOAc (3-10%).

Yield: 9% (white solid)

^{1}H-NMR (300 MHz, $CDCl_3$, δ_H): 7.97-7.92 (m, 2H), 6.95-6.90 (m, 2H), 3.84 (s, 3H), 2.72-2.56 (m, 4H), 2.24 (s, 1H), 1.89-1.80 (m, 2H), 1.31 (s, 6H).

^{13}C-NMR (75 MHz, $CDCl_3$, δ_C): 158.8 (C_q), 132.9 (+), 129.9 (C_q), 113.7 (+), 76.5 (C_q), 71.1 (C_q), 55.4 (+), 27.9 (–), 25.8 (+), 25.1 (–).

HRMS (APCI) (m/z): [$M+H^+$] ($C_{14}H_{21}O_2S_2^+$) calc. 285.0977; observed 285.0971.

2-(2-(3-(1,3-Dithian-2-yl)-3-methylbutyl)-1,3-dithian-2-yl)propan-2-ol (3na)

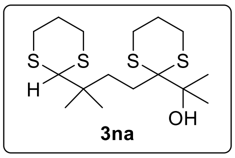

Equivalents: 1 eq. **1n**, 10 eq. **2a**

Column chromatography: DCM (for 2 CV) followed by PE/EtOAc (0-15% EtOAc gradient).

Yield: 20% (white solid)

^{1}H-NMR (400 MHz, $CDCl_3$, δ_H): 4.02 (s, 1H), 2.93-2.80 (m, 8H), 2.33 (s, 1H), 2.12-1.75 (m, 8H), 1.44 (s, 6H), 1.11 (s, 6H).

^{13}C-NMR (101 MHz, $CDCl_3$, δ_C): 78.7 (C_q), 64.0 (C_q), 60.4 (+), 38.5 (C_q), 37.1 (–), 31.7 (–), 31.5 (–), 27.3 (–), 26.5 (+), 26.3 (–), 25.7 (+), 24.1 (–).

HRMS (ESI) (m/z): [$M+H^+$] ($C_{16}H_{31}OS_4^+$) calc. 367.1252; observed 367.1257.

2-(2-Methyl-1,3-dithian-2-yl)pentan-2-ol (3ab)

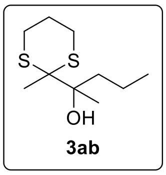

Equivalents: 1 eq. **1a**, 10 eq. **2b**

Column chromatography: DCM (for 1 CV) followed by PE/EtOAc (0-20% EtOAc gradient)

Yield: 53% (Slightly yellow liquid)

^{1}H-NMR (300 MHz, $CDCl_3$, δ_H): 3.01-2.89 (m, 2H), 2.88-2.78 (m, 2H), 2.22 (bs, 1H), 2.08-1.97 (m, 1H), 1.93-1.72 (m, 5H), 1.69-1.37 (m, 3H), 1.35 (d, J = 0.9 Hz, 3H), 0.93 (t, J = 7.2 Hz, 3H).

^{13}C-NMR (75 MHz, $CDCl_3$, δ_C): 78.6 (C_q), 61.8 (C_q), 38.7 (–), 26.9 (–), 26.8 (–), 25.0 (–), 24.7 (+), 21.3 (+), 17.2(–), 14.8 (+).

HRMS (APCI) (m/z): [M+H$^+$] ($C_{10}H_{21}OS_2^+$) calc. 221.1028; observed 221.1028.

3-Methyl-2-(2-methyl-1,3-dithian-2-yl)butan-2-ol (3ac)

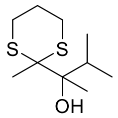

3ac

Equivalents: 1 eq. **1a**, 10 eq. **2c**

Column chromatography: DCM (for 1 CV) followed by PE/EtOAc (0-20% EtOAc gradient)

Yield: 38% (slightly yellow liquid)

^{1}H-NMR (300 MHz, $CDCl_3$, δ_H): 3.01-2.89 (m, 2H), 2.88-2.78 (m, 2H), 2.43 (s, 1H), 2.32 (p, J = 6.8 Hz, 1H), 2.07-1.97 (m, 1H), 1.96-1.81 (m, 4H), 1.27 (s, 3H), 1.08-0.97 (m, 6H).

^{13}C-NMR (75 MHz, $CDCl_3$, δ_C): 80.4 (C_q), 62.8 (C_q), 34.7 (+), 27.0 (–), 26.9 (–), 25.4 (+), 25.0 (–), 20.6 (+), 19.5 (+), 17.6 (+).

HRMS (APCI) (m/z): [M+H$^+$] ($C_{10}H_{21}OS_2^+$) calc. 221.1028; observed 221.1029.

1-(2-Methyl-1,3-dithian-2-yl)cyclobutan-1-ol (3ad)

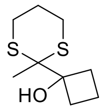

3ad

Equivalents: 1 eq. **1a**, 3 eq. **2d**

Column chromatography: DCM (for 1 CV) followed by PE/EtOAc (0-20% EtOAc gradient)

Yield: 58% (slightly yellow liquid)

^{1}H-NMR (300 MHz, $CDCl_3$, δ_H): 2.96-2.80 (m, 4H), 2.76 (s, 1H), 2.67-2.50 (m, 2H), 2.08-1.82 (m, 5H), 1.76-1.60 (m, 4H).

^{13}C-NMR (75 MHz, $CDCl_3$, δ_C): 81.8 (C_q), 57.4 (C_q), 31.5 (–), 26.6 (–), 25.0 (–), 23.8 (+), 13.3 (–).

HRMS (EI) (m/z): [M$^+$] ($C_9H_{16}OS_2^+$) calc. 204.0637; observed 204.0639.

1-(2-Methyl-1,3-dithian-2-yl)cyclopentan-1-ol (3ae)

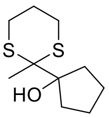

3ae

Equivalents: 1 eq. **1a**, 3 eq. **2e**

Column chromatography: DCM (for 1 CV) followed by PE/EtOAc (0-20% EtOAc gradient)

Yield: 40% (slightly yellow liquid)

^{1}H-NMR (300 MHz, $CDCl_3$, δ_H): 3.04-2.88 (m, 2H), 2.87-2.74 (m, 2H), 2.26 (s, 1H), 2.20-1.99 (m, 3H), 1.94-1.74 (m, 6H), 1.72-1.57 (m, 4H).

^{13}C-NMR (75 MHz, $CDCl_3$, δ_C): 88.4 (C_q), 59.0 (C_q), 36.0 (–), 26.8 (–), 25.2 (–), 25.0 (+), 24.6 (–).

HRMS (EI) (m/z): [M^+] ($C_{10}H_{18}OS_2^+$) calc. 218.0794; observed 218.0787.

4-(2-Methyl-1,3-dithian-2-yl)tetrahydro-2H-pyran-4-ol (3af)

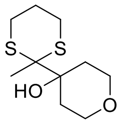

3af

Equivalents: 1 eq. **1a**, 3 eq. **2f**

Column chromatography: First column: DCM (for 1.5 CV) followed by PE/EtOAc (0-40% EtOAc gradient). Second column: DCM/MeOH (0-10%).

Yield: 58% (white solid)

^{1}H-NMR (300 MHz, $CDCl_3$, δ_H): 3.86-3.69 (m, 4H), 2.98-2.77 (m, 4H), 2.39 (s, 1H), 2.13-1.96 (m, 3H), 1.95-1.82 (m, 1H), 1.77 (s, 3H), 1.74-1.65 (m, 2H).

^{13}C-NMR (75 MHz, $CDCl_3$, δ_C): 75.4 (C_q), 63.9 (–), 60.5 (C_q), 32.5 (–), 26.7 (–), 24.9 (–), 24.2 (+).

HRMS (APCI) (m/z): [$M+H^+$] ($C_{10}H_{19}O_2S_2^+$) calc. 235.0821; observed 235.0820.

2-(2-Methyl-1,3-dithian-2-yl)hex-5-en-2-ol (3ag)

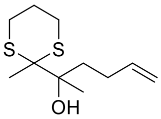

3ag

Equivalents: 1 eq. **1a**, 2 eq. **2g**

Column chromatography: DCM (for 1 CV) followed by PE/EtOAc (0-20% EtOAc gradient)

Yield: 40% (colorless liquid)

^{1}H-NMR (300 MHz, $CDCl_3$, δ_H): 5.93-5.77 (m, 1H), 5.09-4.90 (m, 2H), 3.01-2.78 (m, 4H), 2.35-1.65 (m, 10H), 1.37 (d, J = 0.8 Hz, 3H).

^{13}C-NMR (75 MHz, $CDCl_3$, δ_C): 139.1 (+), 114.6 (–), 78.5 (C_q), 61.7 (C_q), 35.7 (–), 28.4 (–), 26.9 (–), 26.9 (–), 25.0 (–), 24.7 (+), 21.3 (+).

HRMS (EI) (m/z): [M^+] ($C_{11}H_{20}OS_2^+$) calc. 232.0950; observed 232.0949.

2-(2-Methyl-1,3-dithian-2-yl)-4-phenylbutan-2-ol (3ah)[23]

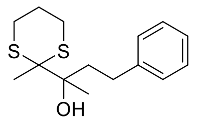

3ah

Equivalents: 1 eq. **1a**, 2 eq. **2h**

Column chromatography: DCM (for 1.5 CV) followed by PE/EtOAc (0-20% EtOAc gradient)

Yield: 40% (slightly yellow highly viscous liquid)

^{1}H-NMR (300 MHz, $CDCl_3$, δ_H): 7.33-7.15 (m, 5H), 3.02-2.66 (m, 6H), 2.39 (s, 1H), 2.28-2.16 (m, 1H), 2.09-1.77 (m, 6H), 1.48 (s, 3H).

^{13}C-NMR (75 MHz, $CDCl_3$, δ_C): 142.7 (C_q), 128.6 (+), 128.5 (+), 125.8 (+), 78.5 (C_q), 61.8 (C_q), 38.7 (–), 30.4 (–), 26.9 (–), 26.8 (–), 24.9 (–), 24.8 (+), 21.4 (+).

HRMS (APCI) (m/z): [$M+H^+$] ($C_{15}H_{23}OS_2^+$) calc. 283.1185; observed 283.1181.

4-(4-Bromophenyl)-2-(2-methyl-1,3-dithian-2-yl)butan-2-ol (3ai)

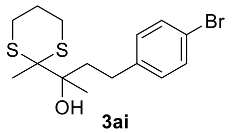

3ai

Equivalents: 1 eq. **1a**, 1 eq. **2i**

Column chromatography: DCM (for 1.5 CV) followed by PE/EtOAc (0-15% EtOAc gradient)

Yield: 31% (slightly yellow highly viscous liquid)

^{1}H-NMR (300 MHz, $CDCl_3$, δ_H): 7.42-7.35 (m, 2H), 7.13-7.06 (m, 2H), 3.01-2.90 (m, 2H), 2.89-2.76 (m, 3H), 2.72-2.60 (m, 1H), 2.28 (s, 1H), 2.22-2.11 (m, 1H), 2.09-1.83 (m, 3H), 1.80 (s, 3H), 1.45 (d, J = 0.8 Hz, 3H).

^{13}C-NMR (101 MHz, $CDCl_3$, δ_C): 141.8 (C_q), 131.5 (+), 130.4 (+), 119.6 (C_q), 78.4 (C_q), 61.7 (C_q), 38.5 (–), 29.9 (–), 26.9 (–), 26.9 (–), 24.9 (–), 24.8 (+), 21.5 (+).

HRMS (APCI) (m/z): [$M+NH_4^+$] ($C_{15}H_{25}BrNOS_2^+$) calc. 378.0555; observed 378.0555.

2-(2-Methyl-1,3-dithian-2-yl)-4-(4-(4,4,5,5-tetramethyl-1,3,2-dioxaborolan-2-yl)phenyl)butan-2-ol (3aj)

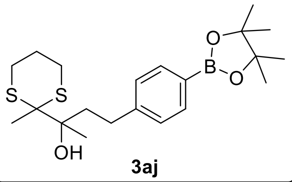

3aj

Equivalents: 1 eq. **1a**, 2 eq. **2j**

Column chromatography: DCM (for 2 CV) followed by PE/EtOAc (0-15% EtOAc gradient)

Yield: 31% (white solid)

^{1}H-NMR (400 MHz, $CDCl_3$, δ_H): 7.74 (d, J = 7.9 Hz, 2H), 7.24 (d, J = 8.0 Hz, 2H), 2.99-2.80 (m, 5H), 2.73 (td, J = 12.8 Hz, 5.3 Hz, 1H), 2.28 (s, 1H), 2.20 (td, J = 12.9 Hz, 4.6 Hz, 1H), 2.07-1.82 (m, 3H), 1.80 (s, 3H), 1.47 (s, 3H), 1.33 (s, 12H).

^{13}C-NMR (101 MHz, $CDCl_3$, δ_C): 146.3 (C_q), 135.1 (+), 128.1 (+), 83.7 (C_q), 78.5 (C_q), 61.8 (C_q), 38.6 (–), 30.7 (–), 26.9 (–), 26.9 (–), 25.0 (+), 24.9 (–), 24.9 (+), 21.5 (+).

HRMS (APCI) (m/z): [$M+NH_4^+$] ($C_{21}H_{37}BNO_3S_2^+$) calc. 425.2339; observed 425.2334.

4-(3-Hydroxy-3-(2-methyl-1,3-dithian-2-yl)butyl)phenyl acetate (3ak)

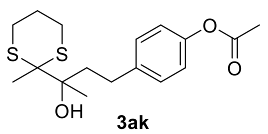

3ak

Equivalents: 1 eq. **1a**, 2 eq. **2k**

Column chromatography: DCM (for 1 CV) followed by PE/EtOAc (0-15% EtOAc gradient)

Yield: 38% (slightly yellow highly viscous liquid)

^{1}H-NMR (400 MHz, $CDCl_3$, δ_H): 7.24-7.18 (m, 2H), 7.01-6.96 (m, 2H), 2.99-2.80 (m, 5H), 2.74-2.65 (m, 1H), 2.37 (s, 1H), 2.28 (s, 3H), 2.24-2.15 (s, 1H), 2.07-1.82 (m, 3H), 1.80 (s, 3H), 1.46 (s, 3H).

^{13}C-NMR (101 MHz, $CDCl_3$, δ_C): 169.8 (C_q), 148.8 (C_q), 140.3 (C_q), 129.5 (+), 121.5 (+), 78.4 (C_q), 61.8 (C_q), 38.7 (–), 29.9 (–), 26.9 (–), 26.8 (–), 24.9 (–), 24.8 (+), 21.4 (+), 21.2 (+).

HRMS (ESI) (m/z): [$M+NH_4^+$] ($C_{17}H_{28}NO_3S_2^+$) calc. 358.1505; observed 358.1511.

tert-Butyl 4-hydroxy-4-(2-methyl-1,3-dithian-2-yl)piperidine-1-carboxylate (3am)

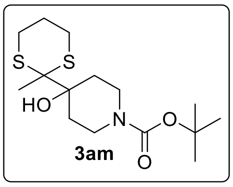

Equivalents: 1 eq. **1a**, 1 eq. **2m**

Column chromatography: First column: DCM (for 1.5 CV) followed by PE/EtOAc (0-40% EtOAc gradient). Second column: DCM/MeOH (0-5%).

Yield: 37% (white solid)

^{1}H-NMR (300 MHz, $CDCl_3$, δ_H): 4.04-3.90 (m, 2H), 3.12-2.98 (m, 2H), 2.98-2.76 (m, 4H), 2.24 (s, 1H), 2.08-1.78 (m, 6H), 1.76 (s, 3H), 1.44 (s, 9H).

^{13}C-NMR (101 MHz, $CDCl_3$, δ_C): 154.9 (C_q), 79.5 (C_q), 76.2 (C_q), 60.7 (C_q), 39.8 (–), 31.7 (–), 28.6 (+), 26.8 (–), 24.8 (–), 24.5 (+).

HRMS (ESI) (m/z): [M+H$^+$] ($C_{15}H_{28}NO_3S_2^+$) calc. 334.1505; observed 334.1509.

1-Methyl-4-(2-methyl-1,3-dithian-2-yl)piperidin-4-ol (3an)

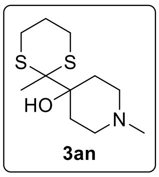

Equivalents: 1 eq. **1a**, 3 eq. **2n**

Column chromatography: DCM(with 1% NEt_3)/MeOH (0-5%).

Yield: 31% (slightly yellow solid)

^{1}H-NMR (400 MHz, $CDCl_3$, δ_H): 2.98-2.80 (m, 4H), 2.78-2.70 (m, 2H), 2.38-2.22 (m, 6H), 2.13-1.97 (m, 3H), 1.96-1.81 (m, 3H), 1.80 (s, 3H).

^{13}C-NMR (101 MHz, $CDCl_3$, δ_C): 75.3 (C_q), 60.7 (C_q), 51.5 (–), 46.1 (+), 31.8 (–), 26.9 (–), 24.9 (–), 24.6 (+).

HRMS (APCI) (m/z): [M+H$^+$] ($C_{11}H_{22}NOS_2^+$) calc. 248.1137; observed 248.1132.

1-(4-Hydroxy-4-(2-methyl-1,3-dithian-2-yl)piperidin-1-yl)ethan-1-one (3ao)

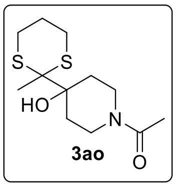

Equivalents: 1 eq. **1a**, 1 eq. **2o**

Column chromatography: DCM/NEt_3/MeOH (98:1:1)

Yield: 42% (white solid)

^{1}H-NMR (300 MHz, C_6D_6, δ_H): 4.88-4.77 (m, 1H), 3.16-2.99 (m, 2H), 2.80 (td, *J* = 12.8 Hz, 3.1 Hz, 1H), 2.42-2.24 (m, 5H), 1.86-1.74 (m, 1H), 1.72 (s, 3H), 1.71-1.62 (m, 3H), 1.58 (m, 3H), 1.45-1.35 (m, 2H).

^{13}C-NMR (101 MHz, $CDCl_3$, δ_C): 168.7 (C_q), 76.0 (C_q), 60.2 (C_q), 42.5 (–), 37.5 (–), 32.2 (–), 31.6 (–), 26.8 (–), 26.7 (–), 24.7 (–), 24.3 (+), 21.5 (+).

HRMS (APCI) (m/z): [M+H$^+$] ($C_{12}H_{22}NO_2S_2^+$) calc. 276.1086; observed 276.1092.

1-(2-Methyl-1,3-dithian-2-yl)-4-(trifluoromethyl)cyclohexan-1-ol (3ap)

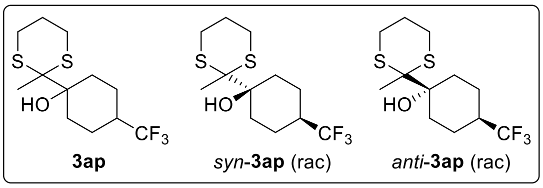

Equivalents: 1 eq. **1a**, 3 eq. **2p**

Column chromatography: First column: PE/Diethylether (10-15% Diethylether). Pure *syn*-diastereomer as fraction 1 and mixture of diastereomers as fraction 2 obtained. Second column for mixed fraction: DCM/NEt_3 (99:1).

Yield: 38% Combined yield of diastereomers. D.r. syn/anti (6/4) based on weight of separated isomers. *syn*-Diastereomer was obtained as white solid and *anti*-diastereomer as slightly yellow liquid.

***syn*-Diastereomer (*syn*-3ap**; -OH and –CF_3 on the same side of the cyclohexyl ring):

^{1}H-NMR (400 MHz, C_6D_6, δ_H): 2.45-2.29 (m, 4H), 1.91-1.87 (m, 1H), 1.87-1.82 (m, 2H), 1.81-1.69 (m, 2H), 1.61 (s, 3H), 1.59-1.52 (m, 2H), 1.52-1.36 (m, 5H).

^{19}F-NMR (376 MHz, C_6D_6, δ_F): -73.5 (s).

^{13}C-NMR (101 MHz, $CDCl_3$, δ_C): 127.8 (q, *J* = 278.6 Hz, C_q), 76.5 (C_q), 60.9 (C_q), 41.49 (q, *J* = 26.7 Hz, +), 30.5 (–), 26.9 (–), 24.9 (–), 24.5 (+), 20.62 (q, *J* = 2.6 Hz, –).

HRMS (EI) (m/z): [M$^+$] ($C_{12}H_{19}F_3OS_2^+$) calc. 300.0824; observed 300.0815.

Crystal Structure (CCDC Deposit Number 2001176):

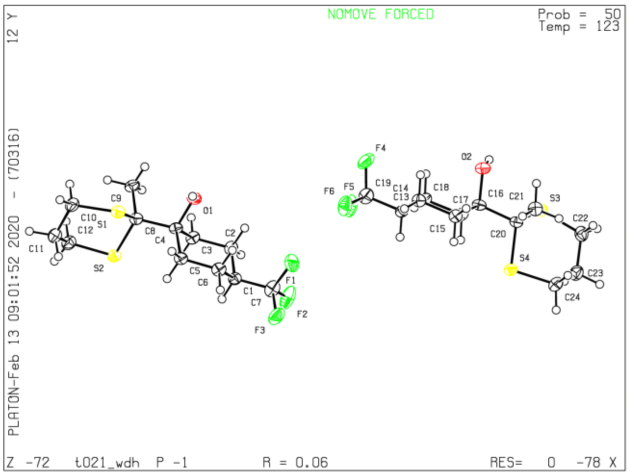

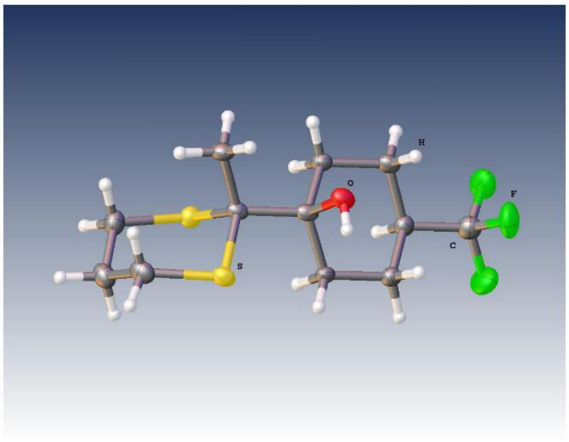

Experimental: Single clear colorless plate crystals of *syn*-**3aq** were used as supplied. A suitable crystal with dimensions 0.17 × 0.14 × 0.06 mm^3 was selected and mounted on a MITIGEN holder with inert oil on a GV1000, TitanS2 diffractometer. The crystal was kept at a steady T = 123.00(10) K during data collection. The structure was solved with the **olex2.solve** 1.3 (Bourhis et al., 2015) solution program using iterative methods and by using **Olex2** (Dolomanov et al., 2009) as the graphical interface. The model was refined with **ShelXL** 2018/3 (Sheldrick, 2015) using full matrix least squares minimization on $\boldsymbol{F}^2$.

Crystal Data. $C_{12}H_{19}F_3OS_2$, M_r = 300.39, triclinic, *P*-1 (No. 2), a = 9.6719(4) Å, b = 12.6401(3) Å, c = 12.6492(4) Å, α = 117.350(3)°, β = 92.492(3)°, γ = 90.004(3)°, V = 1371.89(9) Å^3, T = 123.00(10) K, Z = 4, Z' = 2, μ(Cu K) **= 2.736, 25067 reflections measured, 5154 unique (R_{int} = 0.0689) which were used in all calculations. The final wR_2 was 0.1836 (all data) and R_1 was 0.0576 (I > 2(I)).**

Formula	$C_{12}H_{19}F_3OS_2$	c/Å	12.6492(4)	**Ind't Refl's**	**5154**
$D_{calc.}$/ g cm^{-3}	1.454	α/°	117.350(3)	**Refl's with I > 2(I)**	**4249**
μ/mm^{-1}	2.736	β/°	92.492(3)	$\boldsymbol{R_{int}}$	**0.0689**
Formula Weight	300.39	γ/°	90.004(3)	**Parameters**	**332**
Colour	clear colourless	V/Å^3	1371.89(9)	**Restraints**	**0**
Shape	plate	Z	4	**Largest Peak**	**1.200**
Size/mm^3	0.17×0.14×0.06	Z'	2	**Deepest Hole**	**-0.472**
T/K	123.00(10)	Wavelength/Å	1.39222	**GooF**	**1.069**
Crystal System	triclinic	Radiation type	Cu K	$\boldsymbol{wR_2}$ **(all data)**	**0.1836**
Space Group	*P*-1	$\boldsymbol{\Theta_{min}}$/°	**3.556**	$\boldsymbol{wR_2}$	**0.1627**
a/Å	9.6719(4)	$\boldsymbol{\Theta_{max}}$/°	**60.082**	$\boldsymbol{R_1}$ **(all data)**	**0.0702**
b/Å	12.6401(3)	**Measured Refl's.**	**25067**	$\boldsymbol{R_1}$	**0.0576**

anti-**Diastereomer (*anti*-3ap)**

^{1}H-NMR (400 MHz, C_6D_6, δ_H): 2.43-2.27 (m, 4H), 2.06-1.88 (m, 4H), 1.85-1.66 (m, 6H), 1.65 (s, 3H), 1.47-1.33 (m, 2H).

^{19}F-NMR (376 MHz, C_6D_6, δ_F): -67.3 (s).

^{13}C-NMR (101 MHz, $CDCl_3$, δ_C): 129.04 (q, J = 281.2 Hz, C_q), 76.7 (C_q), 61.6 (C_q), 35.73 (q, J = 25.5 Hz, +), 27.4 (–), 26.8 (–), 25.0 (–), 24.6 (+), 19.61 (q, J = 2.2 Hz, –).

HRMS (APCI) (m/z): [M+H$^+$] ($C_{12}H_{20}F_3OS_2^+$) calc. 301.0902; observed 301.0903.

5-Methyl-5-(2-methyl-1,3-dithian-2-yl)dihydrofuran-2(3H)-one (3aq)[24]

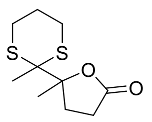

3aq

Equivalents: 1 eq. **1a**, 3 eq. **2q**

Column chromatography: DCM (for 1 CV) followed by PE/EtOAc (0-20% EtOAc gradient)

Yield: 44% (white solid)

^{1}H-NMR (300 MHz, $CDCl_3$, δ_H): 3.22 (ddd, J = 14.1 Hz, 10.1 Hz, 3.1 Hz, 1H), 3.09 (ddd, J = 13.8 Hz, 10.3 Hz, 3.4 Hz, 1H), 2.86-2.73 (m, 1H), 2.70-2.59 (m, 4H), 2.10-1.80 (m, 3H), 1.59 (s, 3H), 1.52 (s, 3H).

^{13}C-NMR (75 MHz, $CDCl_3$, δ_C): 176.3 (C_q), 94.6 (C_q), 54.8 (C_q), 30.6 (–), 29.0 (–), 28.0 (–), 27.3 (–), 25.3 (+), 24.3 (–), 23.8 (+).

HRMS (APCI) (m/z): [M+H$^+$] ($C_{10}H_{17}O_2S_2^+$) calc. 233.0664; observed 233.0661.

1-(2-Methyl-1,3-dithian-2-yl)propan-1-ol (3ar)[25]

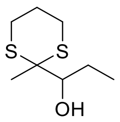

3ar

Equivalents: 3 eq. **1a**, 1 eq. (200 μmol) **2r**

Column chromatography: DCM (for 1.5 CV) followed by PE/EtOAc (0-15% EtOAc gradient)

Yield: 47% (colorless liquid)

^{1}H-NMR (300 MHz, $CDCl_3$, δ_H): 3.84 (dt, J = 10.0 Hz, 1.6 Hz, 1H), 3.05-2.92 (m, 2H), 2.76 (t, J = 1.9 Hz, 1H), 2.65-2.55 (m, 2H), 2.13-1.94 (m, 2H), 1.91-1.76 (m, 1H), 1.41-1.27 (m, 4H), 1.08 (t, J = 7.3 Hz, 3H).

^{13}C-NMR (75 MHz, $CDCl_3$, δ_C): 73.1 (+), 54.1 (C_q), 26.3 (–), 25.7 (–), 24.5 (–), 23.3 (–), 21.8 (+), 12.4 (+).

HRMS (APCI) (m/z): [M+H$^+$] ($C_8H_{17}OS_2^+$) calc. 193.0715; observed 193.0716.

2-Methyl-1-(2-methyl-1,3-dithian-2-yl)propan-1-ol (3as)

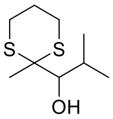

3as

Equivalents: 3 eq. **1a**, 1 eq. (200 µmol) **2s**
Column Chromatography: DCM (for 2 CV) followed by PE/EtOAc (0-15% EtOAc gradient).
Yield: 42% (slightly yellow liquid)
^{1}H-NMR (300 MHz, $CDCl_3$, δ_H): 3.79 (d, *J* = 2.4 Hz, 1H), 3.03-2.90 (m, 2H), 2.69-2.57 (m, 2H), 2.51 (bs, 1H), 2.36-2.21 (m, 1H), 2.13-2.00 (m, 1H), 1.93-1.77 (m, 1H), 1.45 (s, 3H), 1.11 (d, *J* = 7.0 Hz, 3H), 0.96 (d, *J* = 6.8 Hz, 3H).
^{13}C-NMR (75 MHz, $CDCl_3$, δ_C): 75.0 (+), 55.0 (C_q), 28.3 (+), 26.4 (–), 26.0 (–), 24.5 (–), 24.3 (+), 22.9 (+), 17.5 (+).
HRMS (EI) (m/z): [M$^+$] ($C_9H_{18}OS_2^+$) calc. 206.0794; observed 206.0789.

1-(2-Methyl-1,3-dithian-2-yl)-3-(methylthio)propan-1-ol (3at)

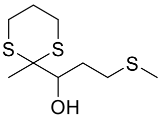

3at

Equivalents: 3 eq. **1a**, 1 eq. (200 µmol) **2t**
Column chromatography: DCM (for 1.5 CV) followed by PE/EtOAc (0-20% EtOAc gradient)
Yield: 40% (slightly yellow liquid)
^{1}H-NMR (300 MHz, $CDCl_3$, δ_H): 4.22 (dd, *J* = 9.8 Hz, 1.5 Hz, 1H), 3.15-2.96 (m, 2H), 2.83-2.49 (m, 5H), 2.31-2.20 (m, 1H), 2.16-2.04 (m, 4H), 1.90-1.75 (m, 1H), 1.72-1.59 (m, 1H), 1.36 (s, 3H).
^{13}C-NMR (75 MHz, $CDCl_3$, δ_C): 69.1 (+), 53.6 (C_q), 32.3 (–), 29.2 (–), 26.1 (–), 25.7 (–), 24.4 (–), 21.6 (+), 15.3 (+).
HRMS (APCI) (m/z): [M+H$^+$] ($C_9H_{19}OS_3^+$) calc. 239.0593; observed 239.0592.

1-(2-Cyclohexyl-1,3-dithian-2-yl)propan-1-ol (3kr)

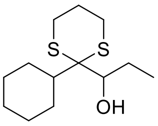

3kr

Equivalents: 3 eq. **1k**, 1 eq. (200 μmol) **2r**

Column chromatography: PE/Diethylether (5-15% gradient)

Yield: 36% (highly viscous yellow liquid) (2.1 eq. of **1k** recovered after the reaction)

^{1}H-NMR (400 MHz, $CDCl_3$, δ_H): 3.96 (dd, J = 10.2 Hz, 1.7 Hz, 1H), 3.06-2.86 (m, 3H), 2.68-2.56 (m, 2H), 2.23-2.13 (m, 2H), 2.08-1.96 (m, 2H), 1.89-1.36 (m, 7H), 1.29-1.07 (m, 7H).

^{13}C-NMR (101 MHz, $CDCl_3$, δ_C): 74.9 (+), 63.6 (C_q), 45.3 (+), 29.3 (–), 28.9 (–), 27.1 (–), 26.9 (–), 26.9 (–), 26.5 (–), 25.0 (–), 24.8 (–), 23.7 (–), 12.7 (+).

HRMS (APCI) (m/z): [M-H_2O+H^+] ($C_{13}H_{23}S_2^+$) calc. 243.1236; observed 243.1237.

1-(2-Phenyl-1,3-dithian-2-yl)propan-1-ol (3lr)[26]

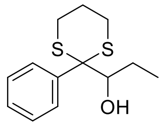

3lr

Equivalents: 1 eq. **1l**, 1.5 eq. **2r**

Column chromatography: First column: PE/Diethylether (10-20% gradient). Second column: PE/EtOAc (5-10% gradient).

Yield: 35% (colorless highly viscous liquid)

^{1}H-NMR (300 MHz, $CDCl_3$, δ_H): 8.00-7.92 (m, 2), 7.45-7.35 (m, 2H), 7.33-7.26 (m, 1H), 3.72 (dd, J = 10.5 Hz, 2.1 Hz, 1H), 2.79-2.61 (m, 4H), 2.06 (s, 1H), 1.98-1.87 (m, 2H), 1.69-1.55 (m, 1H), 1.28-1.11 (m, 1H), 0.90 (t, J = 7.3 Hz, 3H).

^{13}C-NMR (75 MHz, $CDCl_3$, δ_C): 138.8 (C_q), 130.1 (+), 128.7 (+), 127.5 (+), 80.3 (+), 66.3 (C_q), 27.4 (–), 27.2 (–), 25.2 (–), 24.7 (–), 11.3 (+).

HRMS (APCI) (m/z): [M+H^+] ($C_{13}H_{19}OS_2^+$) calc. 255.0872; observed 255.0874.

1-(2-(4-Methoxyphenyl)-1,3-dithian-2-yl)propan-1-ol (3mr)[26]

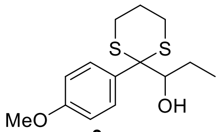

3mr

Equivalents: 1 eq. **1m**, 1.5 eq. **2r**

Column chromatography: DCM (for 1.5 CV) followed by PE/EtOAc (0-15% EtOAc gradient)

Yield: 62% (colorless solid)

^{1}H-NMR (400 MHz, $CDCl_3$, δ_H): 7.87-7.81 (m, 2H), 6.93-6.88 (m, 2H), 3.81 (s, 3H), 3.68 (ddd, J = 10.5 Hz, 5.6 Hz, 2.0 Hz, 1H), 2.75-2.62 (m, 4H), 2.16 (d, J = 5.6 Hz, 1H), 1.95-1.86 (m, 2H), 1.68-1.56 (m, 1H), 1.22-1.11 (m, 1H), 0.89 (t, J = 7.3 Hz, 3H)

^{13}**C-NMR** (101 MHz, $CDCl_3$, δ_C): 158.8 (C_q), 131.4 (+), 130.5 (+), 113.8 (C_q), 80.3 (+), 65.8 (C_q), 55.4 (+), 27.3 (–), 27.1 (–), 25.3 (–), 24.6 (–), 11.2 (+).

HRMS (APCI) (m/z): [M+H$^+$] ($C_{14}H_{21}O_2S_2^+$) calc. 285.0977; observed 285.0979.

1-(2-(4-(2-(1,3-dithian-2-yl)ethyl)phenyl)-1,3-dithian-2-yl)propan-1-ol (3or)

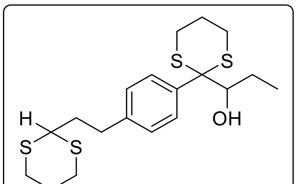

Equivalents: 1 eq. **1o**, 1.5 eq. **2r**

Column chromatography: DCM (for 2 CV) followed by PE/EtOAc (0-15% EtOAc gradient).

Yield: 32% (white solid)

^{1}**H-NMR** (400 MHz, $CDCl_3$, δ_H): 7.84 (d, J = 8.3 Hz, 2H), 7.22 (d, J = 8.3 Hz, 2H), 4.03 (t, J = 7.0 Hz, 1H), 3.71 (dd, J = 10.5, 2.0 Hz, 1H), 2.92-2.80 (m, 6H), 2.77-2.63 (m, 4H), 2.17-1.99 (m, 4H), 1.96-1.82 (m, 3H), 1.69-1.58 (m, 1H), 1.23-1.12 (m, 1H), 0.90 (t, J = 7.3 Hz, 3H).

^{13}**C-NMR** (101 MHz, $CDCl_3$, δ_C): 140.3 (C_q), 136.5 (C_q), 130.2 (+), 128.8(+), 80.2 (+), 66.0 (C_q), 46.8 (+), 36.9 (–), 32.2 (–), 30.4 (–), 27.4 (–), 27.2 (–), 26.1 (–), 25.3 (–), 24.7 (–), 11.3 (+).

HRMS (ESI) (m/z): [M+Na$^+$] ($C_{19}H_{28}OS_4Na^+$) calc. 423.0915; observed 423.0917.

4. Detailed reaction optimization process

General procedure for the reaction optimization process (General Procedure C)

A 5 mL crimp cap vial equipped with a magnetic stirring bar was loaded with photocatalyst, 2-methyl-1,3-dithiane (**1a**), acetone (**2a**), HAT-catalyst, 4 Å molecular sieves (50 mg), solvent and if noted an additive all in the amounts given in the corresponding tables. In doing so, all solid compounds were added before capping the vial, whereas all liquid compounds were added *via* syringe after setting the capped vial under a N_2-atmosphere. The reaction mixture was degassed by three cycles of freeze-pump-thaw and subsequently stirred under light irradiation for the given time at the described temperature. Subsequently, an aliquot of the reaction mixture was submitted to GC-FID analysis to determine the product yield with *n*-decane as internal standard.

Table S1. Initial product observation and control experiments.[a]

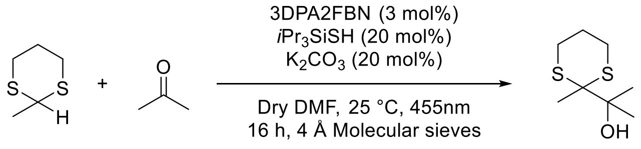

Entry	Light source	PC	HAT catalyst	Base	Yield[b] [%]	Conv. [%]
1	455	3DPA2FBN (3 mol%)	*i*Pr_3SiSH (20 mol%)	K_2CO_3 (20 mol%)	11 (9)[c]	31
2	400	3DPA2FBN (3 mol%)	*i*Pr_3SiSH (20 mol%)	K_2CO_3 (20 mol%)	9	30
3	–	3DPA2FBN (3 mol%)	*i*Pr_3SiSH (20 mol%)	K_2CO_3 (20 mol%)	n.d.	7
4	455	–	*i*Pr_3SiSH (20 mol%)	K_2CO_3 (20 mol%)	n.d.	4
5	455	3DPA2FBN (3 mol%)	–	K_2CO_3 (20 mol%)	16	26
**6**	**455**	**3DPA2FBN (3 mol%)**	***i*Pr_3SiSH (20 mol%)**	–	**30**	**52**
7	455	3DPA2FBN (3 mol%)	–	–	n.d.	8

[a] Reactions were performed with 2-methyl-1,3-dithiane (**1a**) (200 µmol, 1 eq.) and acetone (**2a**) (2.0 mmol, 10 eq.) in degassed dry DMF (2 mL) in the presence of 4 Å molecular sieves (50 mg). [b] Determined by GC-FID analysis with *n*-decane as internal standard. [c] Isolated yield.

Table S2. Photocatalyst screening.[a]

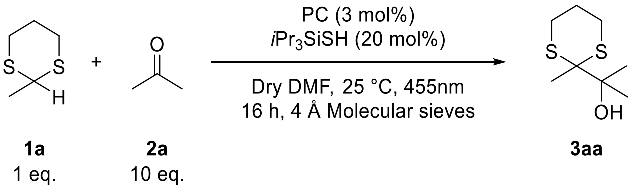

Entry	Photocatalyst (3 mol%)	Yield[b] [%]	Conv. [%]
**1**	**3DPA2FBN**	**30**	**52**
2	3DPAFIPN	7	36
3	4Cz*p*HBN	7	34
4	4CzIPN	2	27
5	(Ir[dF(CF_3)ppy]$_2$(dtbpy))PF_6	n.d.	7
6	Ir(ppy)$_3$	n.d.	10

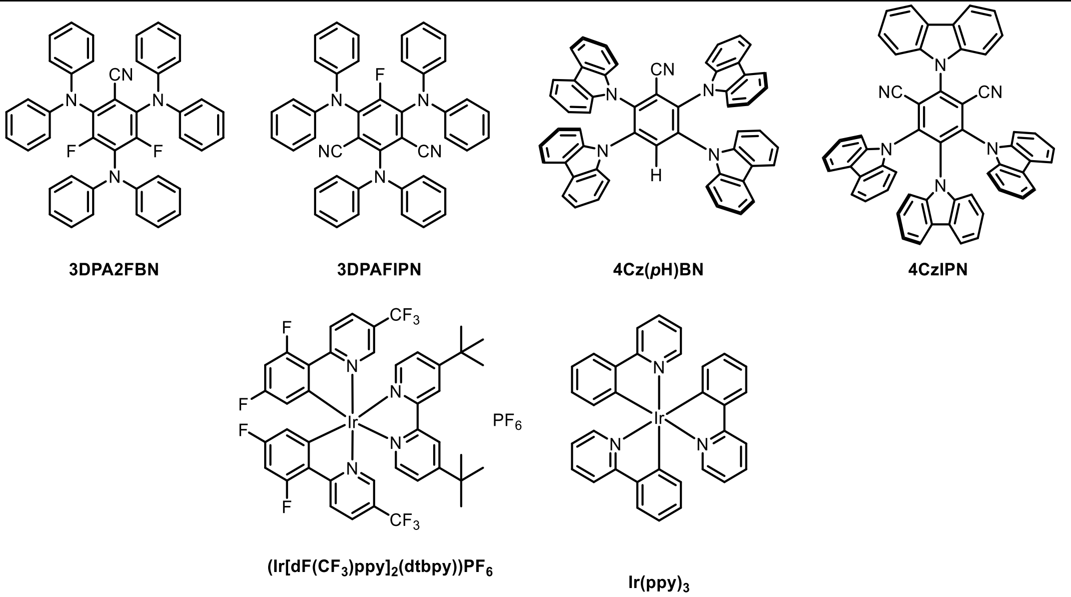

[a] Reactions were performed with 2-methyl-1,3-dithiane (**1a**) (200 µmol, 1 eq.) and acetone (**2a**) (2.0 mmol, 10 eq.) in degassed dry DMF (2 mL) in the presence of 4 Å molecular sieves (50 mg). [b] Determined by GC-FID analysis with *n*-decane as internal standard.

Table S3. HAT-Catalyst screening.[a]

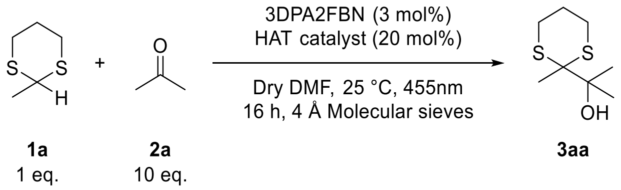

Entry	HAT catalyst (20 mol%)	Base (20 mol%)	Yield[b] [%]	Conv. [%]
**1**	***i*Pr$_3$SiSH**	**–**	**30**	**52**
2	Quinucledin	–	2	12
3	Quinucledin	K_2CO_3	9	16

4	3-Acetoxyquinucledin	-	n.d.	6
5	3-Acetoxyquinucledin	K_2CO_3	10	20
6	NaBr	–	n.d.	7
7	Sulfonamide HAT	–	n.d.	6
8	*i*Pr_3SiOH	–	n.d.	1

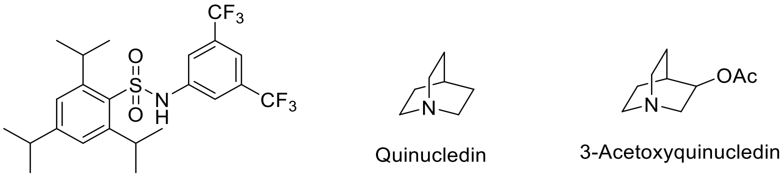

Sulfonamide HAT   Quinucledin   3-Acetoxyquinucledin

[a] Reactions were performed with 2-methyl-1,3-dithiane (**1a**) (200 µmol, 1 eq.) and acetone (**2a**) (2.0 mmol, 10 eq.) in degassed dry DMF (2 mL) in the presence of 4 Å molecular sieves (50 mg). [b] Determined by GC-FID analysis with *n*-decane as internal standard.

Table S4. Brønsted Base/Acid screening.[a]

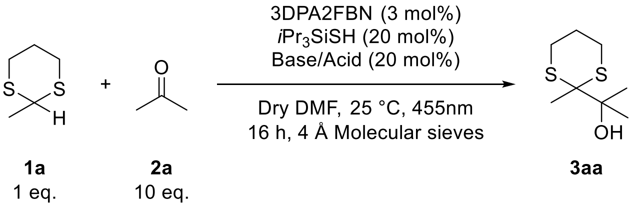

Entry	Base/acid (20 mol%)	Yield[b] [%]	Conv. [%]
**1**	**–**	**30**	**52**
2	K_2CO_3	11	31
3	Lutidin	17	64
4	DBU	6	14
5	Benzoic acid	Traces	6

[a] Reactions were performed with 2-methyl-1,3-dithiane (**1a**) (200 µmol, 1 eq.) and acetone (**2a**) (2.0 mmol, 10 eq.) in degassed dry DMF (2 mL) in the presence of 4 Å molecular sieves (50 mg). [b] Determined by GC-FID analysis with *n*-decane as internal standard.

Table S5. Molecular sieve and acetone co-solvent test.[a]

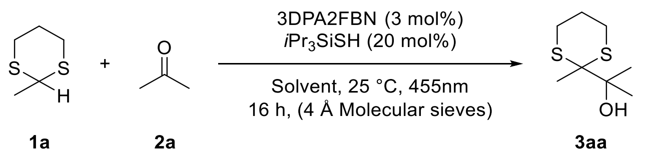

Entry	Condition variation	Yield[b] [%]	Conv. [%]
**1**	**–**	**30**	**52**
2	No molecular sieve	3	14
3	Dry DMF/Acetone (1:1) solvent mixture	12	32

[a] Reactions were performed with 2-methyl-1,3-dithiane (**1a**) (200 µmol, 1 eq.) and acetone (**2a**) (2.0 mmol, 10 eq.) in degassed dry DMF (2 mL) and if noted in the presence of 4 Å molecular sieves (50 mg). [b] Determined by GC-FID analysis with *n*-decane as internal standard.

Table S6. Catalyst loading. [a]

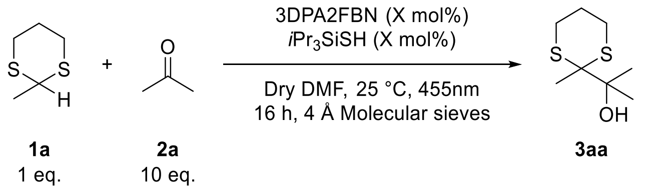

Entry	3DPA2FBN (X mol%)	*i*Pr$_3$SiSH (X mol%)	Yield[b] [%]	Conv. [%]
1	1	20	15	32
2	3	20	30	52
3	5	20	32	62
**4**	**3**	**10**	**32**	**48**

[a] Reactions were performed with 2-methyl-1,3-dithiane (**1a**) (200 µmol, 1 eq.) and acetone (**2a**) (2.0 mmol, 10 eq.) in degassed dry DMF (2 mL) in the presence of 4 Å molecular sieves (50 mg). [b] Determined by GC-FID analysis with *n*-decane as internal standard.

Table S7. Concentration variation.

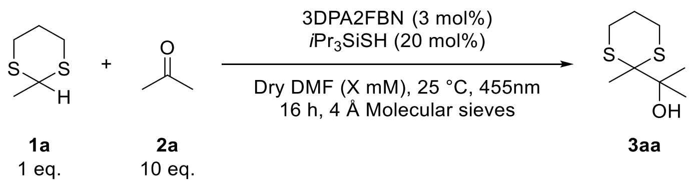

Entry	Concentration	Yield[a] [%]	Conv. [%]
1[b]	50 mM	16	35
2[c]	100 mM	30	52
**3**[d]	**200 mM**	**39**	**59**

[a] Determined by GC-FID analysis with *n*-decane as internal standard. [b] Reaction was performed with 2-methyl-1,3-dithiane (**1a**) (100 µmol, 1 eq.) and acetone (**2a**) (1.0 mmol, 10 eq.) in degassed dry DMF (2 mL) in the presence of 4 Å molecular sieves (50 mg). [c] Reaction was performed with 2-methyl-1,3-dithiane (**1a**) (200 µmol, 1 eq.) and acetone (**2a**) (2.0 mmol,

10 eq.) in degassed dry DMF (2 mL) in the presence of 4 Å molecular sieves (50 mg). [d] Reaction performed with 2-methyl-1,3-dithiane (**1a**) (200 µmol, 1 eq.) and acetone (**2a**) (2.0 mmol, 10 eq.) in degassed dry DMF (1 mL) in the presence of 4 Å molecular sieves (50 mg).

Table S8. Time variation.[a]

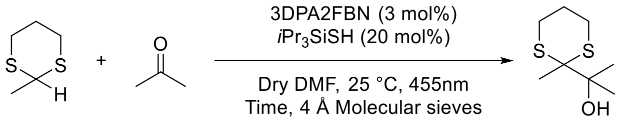

1a
1 eq.
2a
10 eq.
3aa

Entry	Time [h]	Yield[b] [%]	Conv. [%]
1	1	26	40
2	2	31	45
**3**	**3**	**31**	**46**
4	16	30	52
5	40	30	55

[a] Reactions were performed with 2-methyl-1,3-dithiane (**1a**) (200 µmol, 1 eq.) and acetone (**2a**) (2.0 mmol, 10 eq.) in degassed dry DMF (2 mL) in the presence of 4 Å molecular sieves (50 mg). [b] Determined by GC-FID analysis with *n*-decane as internal standard.

Table S9. Solvent screening.[a]

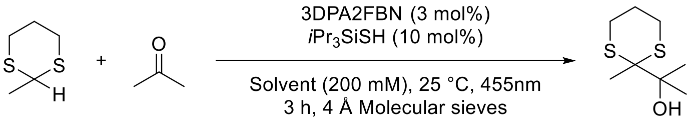

1a
1 eq.
2a
10 eq.
3aa

Entry	Solvent	Yield[b] [%]	Conv. [%]
**1**	**Dry DMF**	**44**	**57**
2	Dry DMSO	29	45
3	Dry MeCN	2	26
4	Dry THF	1	9
5	Dry DCM	0	17

[a] Reactions were performed with 2-methyl-1,3-dithiane (**1a**) (200 µmol, 1 eq.) and acetone (**2a**) (2.0 mmol, 10 eq.) in degassed dry solvent (1 mL) in the presence of 4 Å molecular sieves (50 mg). [b] Determined by GC-FID analysis with *n*-decane as internal standard.

Table S10. Lewis acid screening.[a]

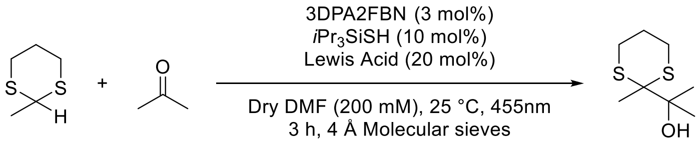

Entry	Lewis Acid (20 mol%)	Yield[b] [%]	Conv. [%]
1	–	44	57
2	$FeCl_3$	n.d.	5
3	Schreiner Thiourea	n.d.	1
**4**	**B_2pin_2**	**49**	**65**
5[c]	$LiBF_4$ (50 mol%)	36	57
6[c]	NaCl (50 mol%)	38	58
7[c]	$Mg(ClO_4)_2$	18	45

[a] Reactions were performed with 2-methyl-1,3-dithiane (**1a**) (200 µmol, 1 eq.) and acetone (**2a**) (2.0 mmol, 10 eq.) in degassed dry DMF (1 mL) in the presence of 4 Å molecular sieves (50 mg). [b] Determined by GC-FID analysis with *n*-decane as internal standard. [c] Experiment run at 0 °C for 16 h.

Table S11. Temperature and power variation.[a]

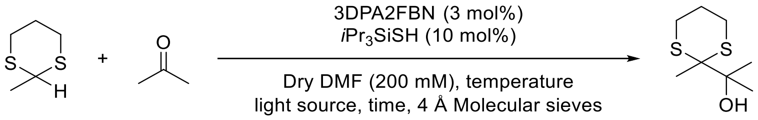

Entry	Temperature [°C]	Light source (455 nm)	Time [h]	Yield[b] [%]	Conv. [%]
1	25	standard	3	44	57
2	0	standard	3	38	48
3	0	standard	6	56	71
**4**	**0**	**standard**	**16**	**59**	**76**
5	0	high power	3	42	56
6	0	high power	16	31	54

[a] Reactions were performed with 2-methyl-1,3-dithiane (**1a**) (200 µmol, 1 eq.) and acetone (**2a**) (2.0 mmol, 10 eq.) in degassed dry DMF (1 mL) in the presence of 4 Å molecular sieves (50 mg). [b] Determined by GC-FID analysis with *n*-decane as internal standard.

Table S12. Inhibition test.[a]

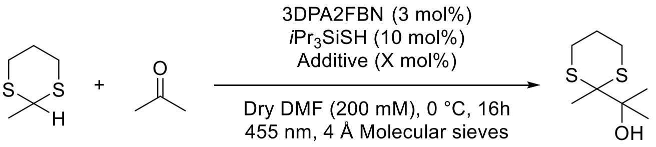

1a 1 eq. **2a** 10 eq. **3aa**

Entry	Additive	Yield[b] [%]	Conv. [%]
1	–	59	76
2	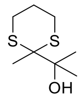 (50 mol%)	52	70
3	iPr$_3$SiOH (10 mol%)	58	77
4	Diphenylamine (6 mol%)	56	74
5	H_2O (1 eq.)	50	70

[a] Reactions were performed with 2-methyl-1,3-dithiane (**1a**) (200 µmol, 1 eq.) and acetone (**2a**) (2.0 mmol, 10 eq.) in degassed dry DMF (1 mL) in the presence of 4 Å molecular sieves (50 mg). [b] Determined by GC-FID analysis with *n*-decane as internal standard.

*i*Pr$_3$SiOH was observed by GC/MS after the reaction, illustrating a possible degradation product of the *i*Pr$_3$SiSH HAT catalyst. Thus, the addition of *i*Pr$_3$SiOH prior to irradiation was tested to determine whether its presence hampers the reaction. The same was done with diphenylamine. Diphenylamine was observed by GC/MS after the reaction, likely due to it being cleaved off of the 3DPA2FBN photocatalyst, leaving a possible photocatalyst degradation product.

Table S13. Boronic Lewis acid screening.[a]

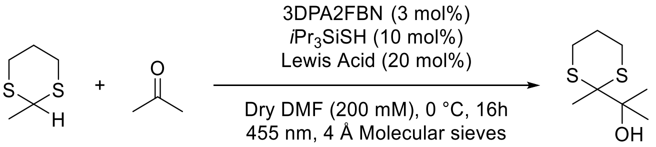

1a 1 eq. **2a** 10 eq. **3a**

Entry	Lewis Acid (20 mol%)	Yield[b] [%]	Conv. [%]
1	B_2pin_2	57	77
2	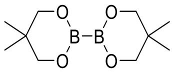 B_2neop_2	65	79
3	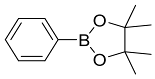	54	74
4	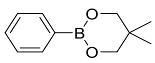	51	71

5	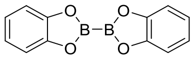	n.d.	11
6	pinB$(CH_2)_3$Bpin	57	73
7	B_2neogly$_2$ (10 mol%)	64	81
**8**	**B_2neogly$_2$ (15 mol%)**	**65**	**79**
**9**	B_2neogly$_2$ (30 mol%)	53	82

[a] Reactions were performed with 2-methyl-1,3-dithiane (**1a**) (200 µmol, 1 eq.) and acetone (**2a**) (2.0 mmol, 10 eq.) in degassed dry DMF (1 mL) in the presence of 4 Å molecular sieves (50 mg). [b] Determined by GC-FID analysis with *n*-decane as internal standard.

Table S14. Catalyst addition test.[a]

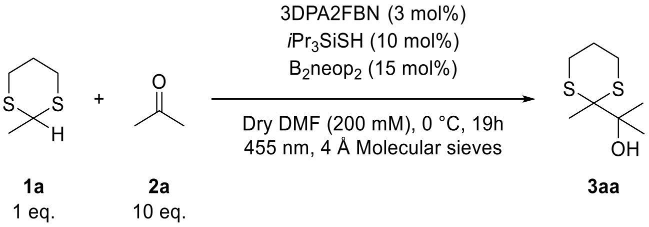

Entry	Catalyst addition (after 4h)	Yield[b] [%]	Conv. [%]
1[c]	–	65	79
2[d]	–	61	81
3	3DPA2FBN (2 mol%)	68	83
4	*i*Pr$_3$SiSH (10 mol%)	69	83
**5**	**3DPA2FBN (2 mol%) and *i*Pr$_3$SiSH (10 mol%)**	**73**	**87**
6[e]	3DPA2FBN (2 mol%) and *i*Pr$_3$SiSH (10 mol%)	72	94

[a] Reactions were performed with 2-methyl-1,3-dithiane (**1a**) (200 µmol, 1 eq.) and acetone (**2a**) (2.0 mmol, 10 eq.) in degassed dry DMF (1 mL) in the presence of 4 Å molecular sieves (50 mg). [b] Determined by GC-FID analysis with *n*-decane as internal standard. [c] 16h reaction time. [d] 38h reaction time. [e] 300 mM reaction concentration by executing the reaction with **1** (300 µmol, 1 eq.) and **2** (3.0 mmol, 10 eq.) in degassed dry DMF (1 mL).

Table S15. Electrophile amount variation.[a]

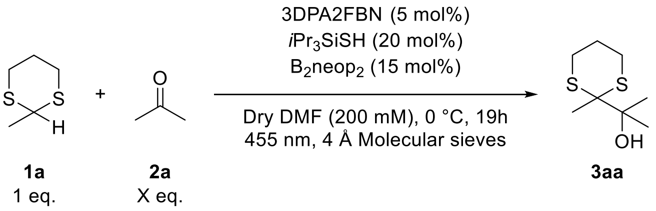

Entry	Acetone (2a) amount	Yield[b] [%]	Conv. [%]
**1**	**10 eq.**	**73**	**87**
2	7.5 eq.	72	85
3	5.0 eq.	66	80
4	2.5 eq.	56	74

[a] Reactions were performed with 2-methyl-1,3-dithiane (**1a**) (200 µmol, 1 eq.) and acetone (**2a**) in degassed dry DMF (1 mL) in the presence of 4 Å molecular sieves (50 mg). [b] Determined by GC-FID analysis with *n*-decane as internal standard.

Table S16. Repetition of control experiments with optimized reaction conditions.[a]

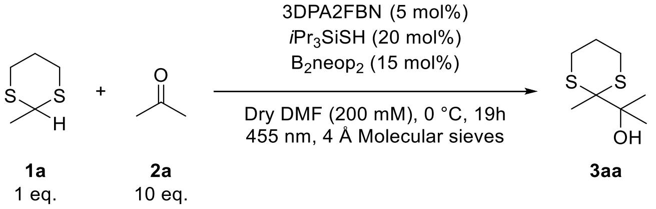

Entry	Light source	PC	HAT catalyst	Yield[b] [%]	Conv. [%]
**1**[c]	**455**	**3DPA2FBN (3 mol%)**	***i*Pr$_3$SiSH (10 mol%)**	**73 (65)**[d]	**87**
2	–	3DPA2FBN (3 mol%)	*i*Pr$_3$SiSH (10 mol%)	n.d.	3
3	455	–	*i*Pr$_3$SiSH (10 mol%)	n.d.	1
4	455	3DPA2FBN (3 mol%)	–	n.d.	1

[a] Reactions were performed with 2-methyl-1,3-dithiane (**1a**) (200 µmol, 1 eq.) and acetone (**2a**) (2.0 mmol, 10 eq.) in degassed dry DMF (2 mL) in the presence of 4 Å molecular sieves (50 mg). [b] Determined by GC-FID analysis with *n*-decane as internal standard. [c] Addition of another 3DPA2FBN (2 mol%) and *i*Pr$_3$SiSH (10 mol%) after 4h. [d] Isolated yield.

5. Lower-yielding or unsuccessful electrophiles and 1,3-dithanes

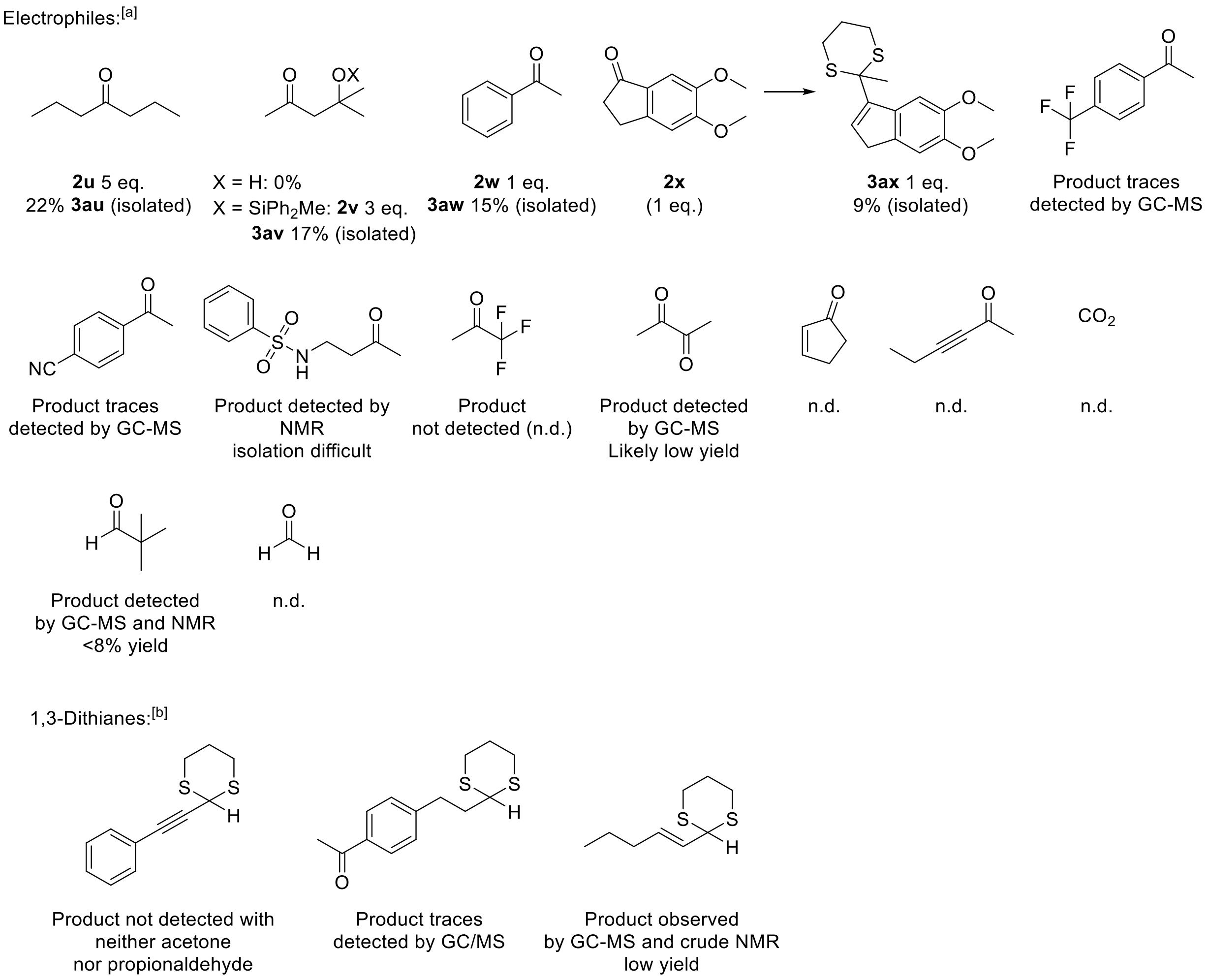

Scheme S1. Unsuccessful or low-yielding electrophiles and 1,3-dithianes. [a] Reactions were performed according to General Procedure B. The second catalyst loading was only given if an isolated yield is noted. 2-Methyl-1,3-dithiane (**1a**) (200 µmol, 1 eq.) was used as nucleophile precursor. [b] Reactions were performed according to General Procedure B without the second catalyst loading. Acetone (**2a**) (2.0 mmol, 10 eq.) was used electrophile.

4-(2-Methyl-1,3-dithian-2-yl)heptan-4-ol (3au)

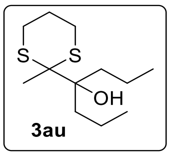

Equivalents: 1 eq. **1a**, 5 eq. **2u**

Column chromatography: DCM (for 1.5 CV) followed by PE/EtOAc (0-15% gradient).

Yield: 22% (colorless liquid)

^{1}H-NMR (300 MHz, $CDCl_3$, δ_H): 3.03-2.76 (m, 4H), 2.23 (s, 1H), 2.09-1.96 (m, 1H), 1.94-1.65 (m, 8H), 1.53-1.37 (m, 4H), 0.92 (t, J = 7.3 Hz, 6H).

^{13}C-NMR (75 MHz, $CDCl_3$, δ_C): 79.5 (C_q), 62.8 (C_q), 38.0 (–), 26.8 (–), 25.1 (–), 24.8 (+), 18.2 (–), 15.0 (+).

HRMS (APCI) (m/z): [$M+NH_4^+$] ($C_{12}H_{28}NOS_2^+$) calc. 266.1607; observed 266.1606.

4-Methyl-2-(2-methyl-1,3-dithian-2-yl)-4-((methyldiphenylsilyl)oxy)pentan-2-ol (3av)

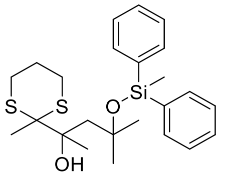

3av

Equivalents: 1 eq. **1a**, 3 eq. **2v**

Column chromatography: PE/EtOAc (5-50% EtOAc gradient).

Yield: 17% (white solid)

^{1}H-NMR (400 MHz, C_6D_6, δ_H): 7.89-7.77 (m, 4H), 7.26-7.13 (m, 6H), 2.58-2.47 (m, 3H), 2.39-2.33 (m, 1H), 2.32-2.23 (m, 2H), 2.17 (s, 1H), 1.91 (s, 3H), 1.87 (s, 3H), 1.52-1.32 (m, 2H), 1.22 (s, 3H), 1.13 (s, 3H), 0.91 (s, 3H).

^{13}C-NMR (75 MHz, $CDCl_3$, δ_C): 137.8 (C_q), 134.9 (+), 134.8 (+), 130.0 (+), 129.6 (+), 128.0 (+), 127.8 (+), 84.9 (C_q), 70.6 (C_q), 62.0 (C_q), 50.3 (–), 32.6 (+), 31.1 (+), 26.9 (–), 26.7 (–), 25.3 (–), 24.9 (+), 23.8 (+), 1.12 (+).

HRMS (APCI) (m/z): [$M+H^+$] ($C_{24}H_{35}O_2S_2Si^+$) calc. 447.1842; observed 447.1837.

1-(2-Methyl-1,3-dithian-2-yl)-1-phenylethan-1-ol (3aw)[23]

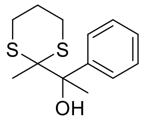

3aw

Equivalents: 1 eq. **1a**, 1 eq. **2w**

Column chromatography: PE/DCM (50-70% gradient).

Yield: 15% (slightly yellow highly viscous liquid)

^{1}H-NMR (400 MHz, CD_2Cl_2, δ_H): 7.64-7.58 (m, 2H), 7.35-7.24 (m, 3H), 3.16 (s, 1H), 2.88-2.69 (m, 4H), 1.98-1.88 (m, 1H), 1.87-1.75 (m, 4H), 1.68 (s, 3H).

^{13}C-NMR (75 MHz, $CDCl_3$, δ_C): 142.9 (C_q), 127.7 (+), 127.5 (+), 127.4 (+), 79.7 (C_q), 59.7 (C_q), 27.3 (–), 27.2 (–), 25.7 (+), 25.6 (+), 24.6 (–).

HRMS (APCI) (m/z): [$M+H^+$] ($C_{13}H_{19}OS_2^+$) calc. 255.0872; observed 255.0875.

2-(5,6-Dimethoxy-1*H*-inden-3-yl)-2-methyl-1,3-dithiane (3ax)

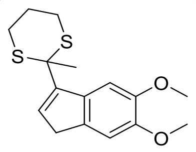

3ax

Equivalents: 1 eq. **1a**, 1 eq. **2x**

Column chromatography: DCM (for 1.5 CV) followed by PE/EtOAc (0-30% EtOAc gradient).

Yield: 9% (yellow solid)

^{1}H-NMR (400 MHz, $CDCl_3$, δ_H): 7.66 (s, 1H), 7.03 (s, 1H), 6.67 (t, *J* = 2.1 Hz, 1H), 3.92 (s, 3H), 3.90 (s, 3H), 3.35 (d, *J* = 1.6 Hz, 2H), 2.97-2.87 (m, 2H), 2.83-2.75 (m, 2H), 2.07-1.89 (m, 5H).

^{13}C-NMR (100 MHz, $CDCl_3$, δ_C): 147.4 (C_q), 147.3 (C_q), 145.4 (C_q), 138.2 (C_q), 135.0 (C_q), 132.6 (+), 107.7 (+), 107.6 (+), 56.4 (+), 56.3 (+), 49.6 (C_q), 37.2 (–), 29.2 (+), 28.1 (–), 25.2 (–).

HRMS (EI) (m/z): [M^+] ($C_{16}H_{20}O_2S_2^+$) calc. 308.0899; observed 308.0892.

6. 1,3-Dithian deprotection

Optimization

Table S17. Solvent screening for photocatalytic deprotection.[a]

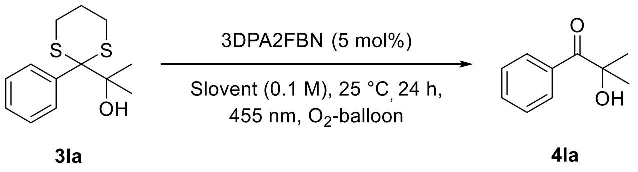

Entry	Solvent	Yield[b] [%]
1	DMF	20
2	MeCN	22
3	H_2O	2
**4**	**DCM**	**49**
5	DCM/H_2O (1:1)	23
6	DCM[c]	25
7	DCM[d]	38

[a] Reactions were performed with **3la** (100 µmol, 1 eq.) in the noted solvent (1 mL) (not degassed or dried prior to use). [b] Determined by GC-FID analysis with *n*-decane as internal standard. [c] H_2O (10 µmol) added. [d] Reaction run in 2 mL solvent.

Table S18. Photocatalyst screening for photocatalytic deprotection.[a]

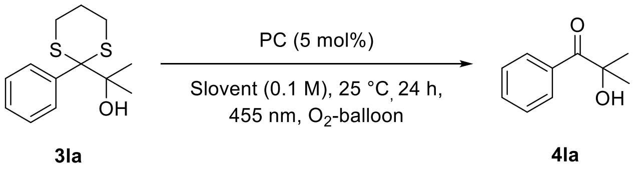

Entry	Photocatalyst	Yield[b] [%]
**1**	**3DPA2FBN**	**49**
2	4CzIPN	38
3	3CzClIPN[3]	35
4	2,4,6-triphenylpyrylium tetrafluoroborate	39
5	I_2 ($CHCl_3$ as solvent)	13
6	I_2 ($CHCl_3$ as solvent)[c]	11
7	3DPA2FBN[d]	5

[a] Reactions were performed with **3la** (100 µmol, 1 eq.) in the noted solvent (1 mL) (not degassed or dried prior to use). [b] Determined by GC-FID analysis with n-decane as internal standard. [c] H_2O (10 µmol) added. [d] Ammonium persulphate (1 eq.) instead of O_2-balloon.

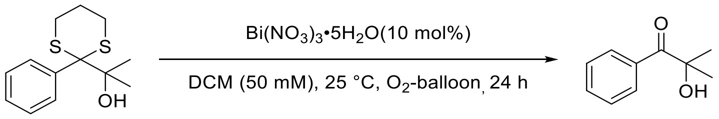

Scheme S2. 1,3-Dithiane deprotection with $Bi(NO_3)_3•5H_2O$ as catalyst.[23] The reaction was performed with **3la** (100 µmol, 1 eq.) in DCM (2 mL) (not degassed or dried prior to use) in absence of a light source.

General procedure for the photocatalytic 1,3-dithiane deprotection (General Procedure D)

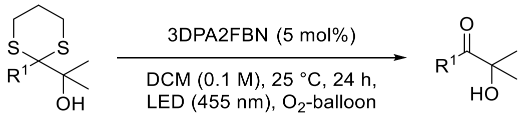

A 5 mL crimp cap vial equipped with a magnetic stirring bar was loaded with 3DPA2FBN (3.2 mg, 5.0 µmol, 5 mol%), the corresponding 1,3-dithiane **3** (100 µmol, 1 eq.) and DCM (1 mL). The vial was capped and an O_2-filled balloon was connected to the vial *via* syringe and septum. The reaction mixture was stirred under light irradiation using a 455 nm (± 15 nm) LED for 24 h at 25 °C.

After the given reaction time *n*-decane was added as internal standard prior to the workup process and an aliquot of the reaction mixture was analyzed by GC-FID to determine the GC-yield. The reaction mixture was filtered off and concentrated under reduced pressure. The crude product was purified by flash column chromatography (hexane/ethyl acetate).

General procedure for the metal-catalyzed 1,3-dithiane deprotection (General Procedure E)

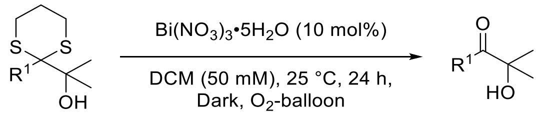

A 5 mL crimp cap vial equipped with a magnetic stirring bar was loaded with $Bi(NO_3)_3•5H_2O$ (4.9 mg, 10 µmol, 10 mol%), the corresponding 1,3-dithiane **3** (100 µmol, 1 eq.) and DCM (2 mL). The vial was capped and an O_2-filled balloon was connected to the vial *via* syringe and septum. The reaction mixture was stirred for 24 h at 25 °C.

After the given reaction time *n*-decane was added as internal standard prior to the workup process and an aliquot of the reaction mixture was analyzed by GC-FID to determine the GC-yield. The reaction mixture was filtered off and concentrated under reduced pressure. The crude product was purified by flash column chromatography (hexane/ethyl acetate).

Table S19. 1,3-Dithiane deprotection of **3aa**.

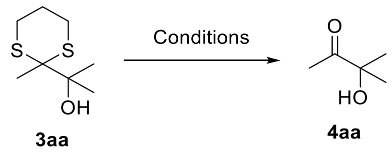

Entry	Conditions	Yield[a] [%]
1	Photocatalytic deprotection (General Procedure D)	53
2	$Bi(NO_3)_3$ catalyzed deprotection (General Procedure E)	86

[a] Determined by GC-FID analysis with *n*-decane as internal standard.

3-Hydroxy-3-methyl-5-phenylpentan-2-one (4ag)[23]

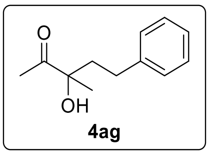

Physical State: colorless liquid.

Yield: 52% (Photocatalytic deprotection; General Procedure D) and 73% ($Bi(NO_3)_3$ catalyzed deprotection; General Procedure E).

^{1}H-NMR (400 MHz, $CDCl_3$, δ_H): 7.41-7.33 (m, 2H), 7.31-7.22 (m, 3H), 3.64 (bs, 1H), 2.92-2.81 (m, 1H), 2.52-2.41 (m, 1H), 2.30 (s, 3H), 2.20-2.05 (m, 2H), 1.50 (s, 3H).

^{13}C-NMR (101 MHz, $CDCl_3$, δ_C): 212.1, 141.6, 128.6, 128.5, 126.2, 78.7, 41.5, 30.0, 25.6, 23.7.

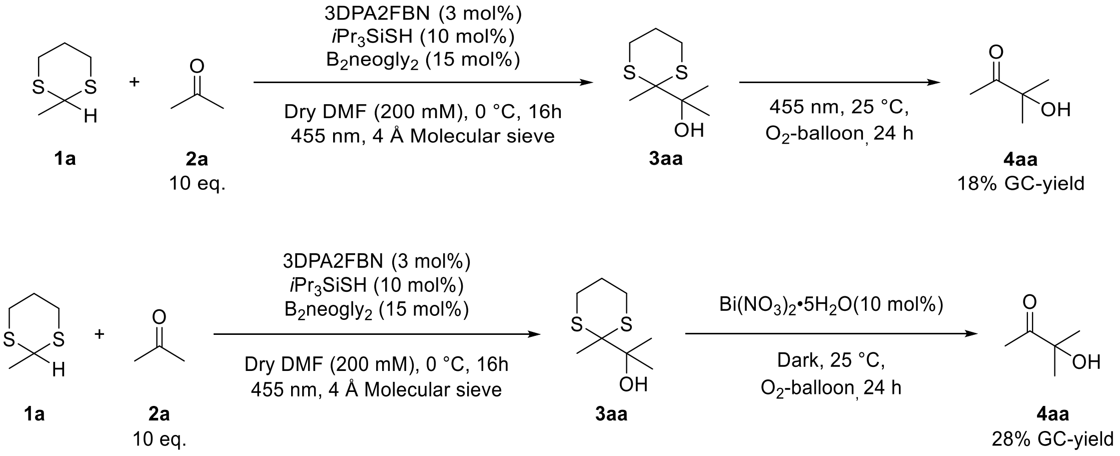

Scheme S3. Photocatalytic Corey-Seebach reaction followed by 1,3-dithiane deprotection in one pot. The given yield is the total over two steps. Upper: With photocatalytic 1,3-dithiane deprotection. Lower: With $Bi(NO_3)_3{\bullet}5H_2O$ catalyzed 1,3-dithiane deprotection.

7. Mechanistic investigations

7.1 Emission quenching studies

Emission quenching of 3DPA2FBN with *i*Pr$_3$SiSH

A 37.5 µM solution of 3DPA2FBN in degassed dry DMF was prepared under a nitrogen atmosphere in a gas-tight 10 mm quartz cuvette (1.5 mL). The photocatalyst was irradiated at 400 nm and the change of the fluorescence emission upon addition of different amounts of quencher was measured (Figure S1). *i*Pr$_3$SiSH was added directly with a Hamilton syringe through the septum of the gas-tight quartz cuvette.

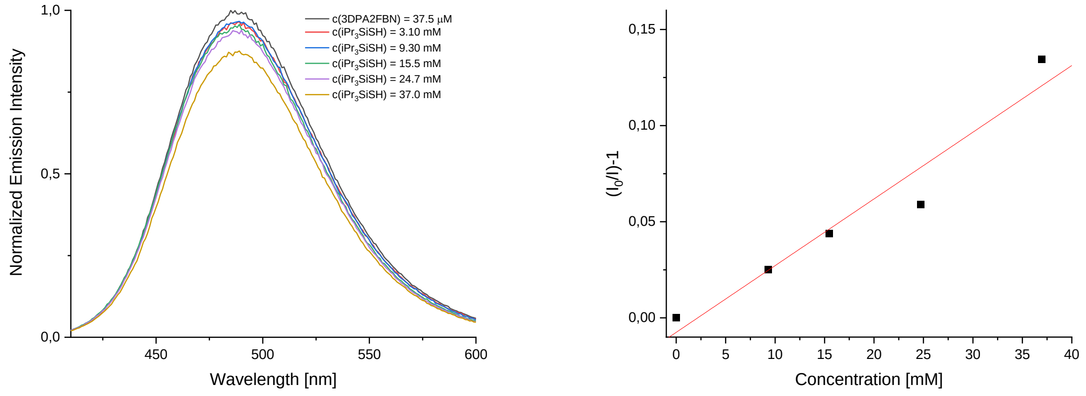

Figure S1. Left: Emission quenching of 3DPA2FBN (37.5 µM in dry DMF) upon addition of *i*Pr$_3$SiSH. Right: Corresponding Stern-Volmer-plot.

*i*Pr$_3$SiSH seems to an inefficient quencher for 3DPA2FBN (Figure S1). Still, a Stern-Volmer for subsequent comparisons was calculated. Therefore, I_0/I-1 versus the quencher concentration was mapped, resulting in a Stern-Volmer constant of K_{SV} = 3.47 M^{-1} determined from the slope of the linear fit.

$$\frac{I_0}{I} - 1 = K_{SV} \cdot [Q]$$

(With I_0 being the fluorescence intensity at 488 nm in absence of the quencher, I the fluorescence intensity at 488 nm in presence of the quencher and [Q] the quencher concentration)

Emission quenching of 3DPA2FBN with *i*Pr$_3$SiSH in presence of 4 Å molecular sieve

A 37.5 µM solution of 3DPA2FBN in degassed dry DMF was prepared under a nitrogen atmosphere in a gas-tight 10 mm quartz cuvette (1.5 mL) and 4 Å molecular sieve (50 mg) were added. The photocatalyst was irradiated at 400 nm and the change of the fluorescence emission upon addition of different amounts of quencher was measured (Figure S1). *i*Pr$_3$SiSH was added directly with a Hamilton

syringe through the septum of the gas-tight quartz cuvette. After each addition the suspension was shaken and 20 min was waited prior measuring the emission spectra in order to let the molecular sieve deposit on the bottom of the cuvette. The suspension was allowed to settle for another 20 min before measuring the emission spectrum again. If approximately the same value for the maximum emission intensity was obtained, the next quencher loading was added. Otherwise the emission spectrum was measured again after 10 min.

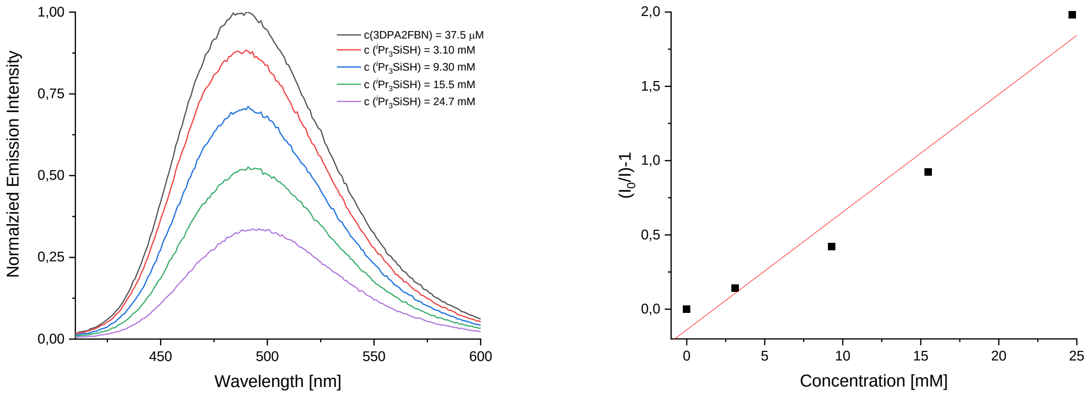

Figure S2. Left: Emission quenching of 3DPA2FBN (37.5 µM in dry DMF) upon addition of *i*Pr_3SiSH in the presence of 4 Å molecular sieve. Right: Corresponding Stern-Volmer-plot.

iPr_3SiSH seems to be a much more potent and efficient quencher for 3DPA2FBN in the presence of 4 Å molecular sieve (K_{SV} = 79.3 M^{-1}) (Figure S2). This indicates, that the molecular sieve actives the HAT catalyst, possibly by the generation of small amounts of deprotonated *i*Pr_3SiSH. A slight shift in the emission maxima upon addition of *i*Pr_3SiSH and the rather non-linear Stern-Volmer-plot may indicate that *i*Pr_3SiSH is reacting with 3DPA2FBN under light irradiation and in the presence of 4 Å molecular sieve. However, the turbidity of the molecular sieve can be the reason for this as well.

Emission quenching of 3DPA2FBN with 2-methyl-1,3-dithiane (1a)

A 37.5 µM solution of 3DPA2FBN in degassed dry DMF was prepared under a nitrogen atmosphere in a gas-tight 10 mm quartz cuvette (1.5 mL). The photocatalyst was irradiated at 400 nm and the change of the fluorescence emission upon addition of different amounts of quencher was measured (Figure S1). 2-Methyl-1,3-dithiane (**1a**) was added directly with a Hamilton syringe through the septum of the gas-tight quartz cuvette.

1a seems to be an inefficient quencher for 3DPA2FBN (Figure S3). A Stern-Volmer plot for subsequent comparisons was calculated. Therefore, $I_0/I-1$ (488nm Intensity maximum) versus the quencher concentration was mapped, resulting in a Stern-Volmer constant of K_{SV} = 1.37 M^{-1} determined from the slope of the linear fit.

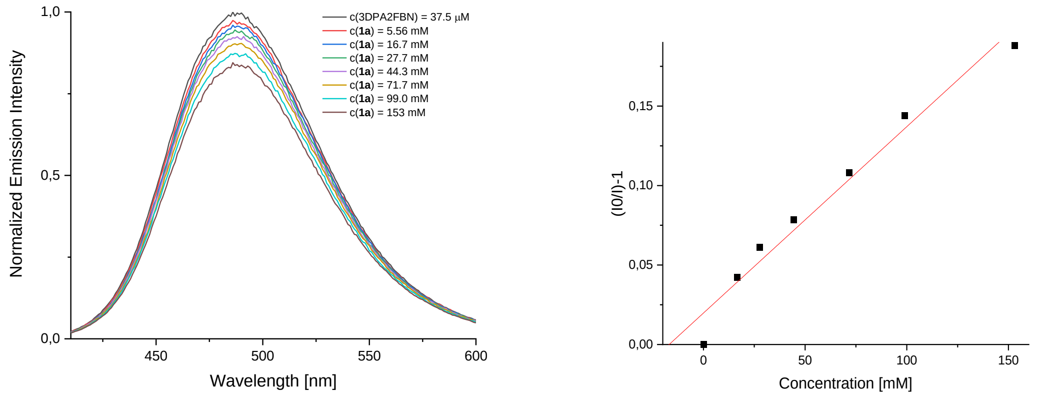

Figure S3. Left: Emission quenching of 3DPA2FBN (37.5 μM in dry DMF) upon addition of 2-methyl-1,3-dithiane (**1a**). Right: Corresponding Stern-Volmer-plot.

Emission quenching of 3DPA2FBN with 2-methyl-1,3-dithiane (1a) in presence of 4 Å molecular sieves

A 37.5 μM solution of 3DPA2FBN in degassed dry DMF was prepared under a nitrogen atmosphere in a gas-tight 10 mm quartz cuvette (1.5 mL) and 4 Å molecular sieves (50 mg) were added. The photocatalyst was irradiated at 400 nm and the change of the fluorescence emission upon addition of different amounts of quencher was measured (Figure S4). 2-Methyl-1,3-dithiane (**1a**) was added directly with a Hamilton syringe through the septum of the gas-tight quartz cuvette. After each addition the suspension was shaken and 20 min was waited prior measuring the emission spectra in order to let the molecular sieves deposit on the bottom of the cuvette. The suspension was allowed to settle for another 20 min before measuring the emission spectrum again. If approximately the same value for the maximum emission intensity was obtained, the next quencher loading was added. Otherwise the emission spectrum was measured again after 10 min.

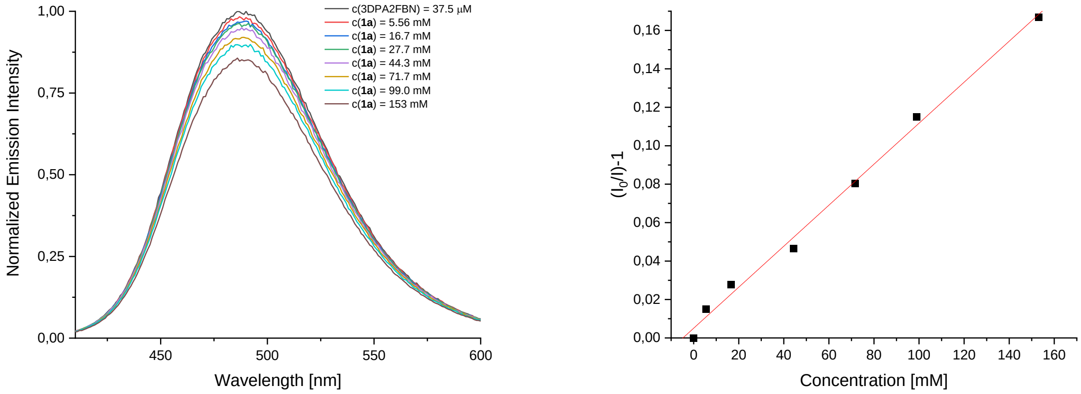

Figure S4. Left: Emission quenching of 3DPA2FBN (37.5 μM in dry DMF) upon addition of 2-methyl-1,3-dithiane (**1a**) in the presence of 4 Å molecular sieves. Right: Corresponding Stern-Volmer-plot.

In presence of molecular sieves, a similar quenching was observed. Opposed to *i*Pr_3SiSH, **1a** is an inefficient quencher in both, absence and presence of molecular sieve (compare Figures S3 and S4). A Stern-Volmer for subsequent comparisons was calculated. Therefore, $I_0/I-1$ (488nm Intensity maximum) versus the quencher concentration was mapped, resulting in a Stern-Volmer constant of K_{SV} = 1.07 M^{-1} determined from the slope of the linear fit.

Emission quenching of 3DPA2FBN with acetone (2a) in presence of 4 Å molecular sieves

A 37.5 µM solution of 3DPA2FBN in degassed dry DMF was prepared by diluting 0.2 mL of a 375 µM 3DPA2FBN to 2 mL. In the same way, an acetone quencher solution was prepared by diluting 0.2 mL of the same 375 µM 3DPA2FBN solution (in dry DMF) to 2 mL using acetone. The resulting quencher solution is thus 37.5 µM in 3DPA2FBN and 12.1 M in acetone.

1.5 mL of the 37.5 µM solution of 3DPA2FBN in degassed dry DMF was transferred to a gas-tight 10 mm quartz cuvette and 4 Å molecular sieves (50 mg) were added. The photocatalyst was irradiated at 400 nm and the change of the fluorescence emission upon addition of different amounts of quencher solution (37.5 µM in 3DPA2FBN and 12.1 M in acetone) was measured (Figure S5). After each addition the suspension was shaken and 20 min was waited prior measuring the emission spectra in order to let the molecular sieves deposit on the bottom of the cuvette. The suspension was allowed to settle for another 20 min before measuring the emission spectrum again. If approximately the same value for the maximum emission intensity was obtained, the next quencher loading was added. Otherwise the emission spectrum was measured again after 10 min.

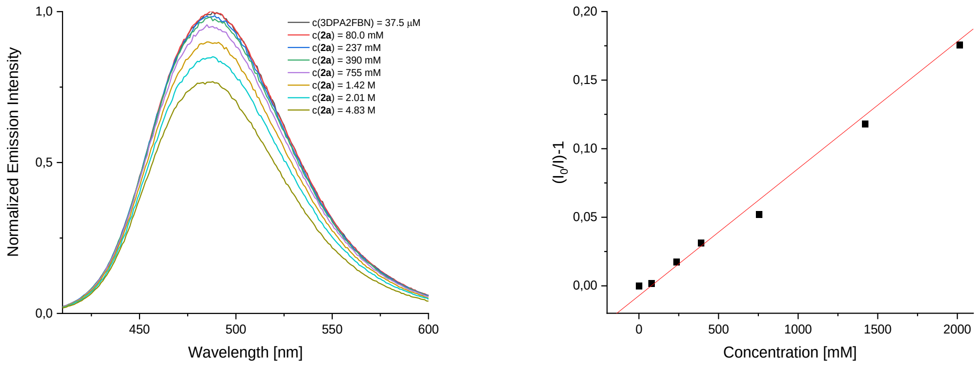

Figure S5. Left: Emission quenching of 3DPA2FBN (37.5 µM in dry DMF) upon addition of acetone (**2a**) in the presence of 4 Å molecular sieves. Right: Corresponding Stern-Volmer-plot.

A Stern-Volmer constant of $K_{SV} = 9.26 \cdot 10^{-2}$ M^{-1} could be determined (Figure S5), showing that acetone is an inefficient quencher. By charting all Stern-Volmer plots into one diagram, the quenching efficiency of all systems can be compared qualitatively.

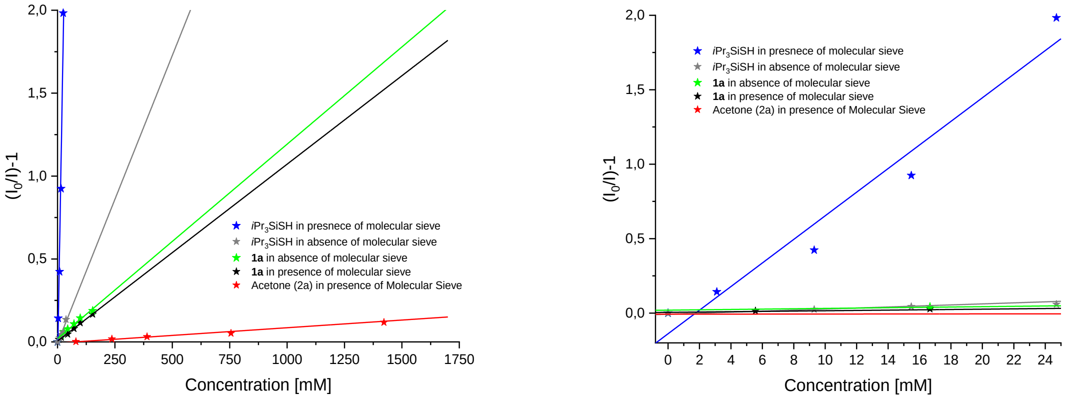

Figure S6. Stern-Volmer plots in comparison.

Comparing the Stern-Volmer plots indicates that the excited 3DPA2FBN almost exclusively interacts with the HAT catalyst (*i*Pr_3SiSH) under the applied standard reaction conditions (c(*i*Pr_3SiSH) = 17.4 mM; c(**1a**) = 174 mM; c(**2a**) = 1740 mM).

7.2 Radical-radical homocoupling

In order to investigate if the activated HAT-catalyst is abstracting a hydrogen atom from the 1,3-dithiane, a selected reaction mixture was analyzed by HRMS seeking for the corresponding radical homocoupling product (Scheme S4).

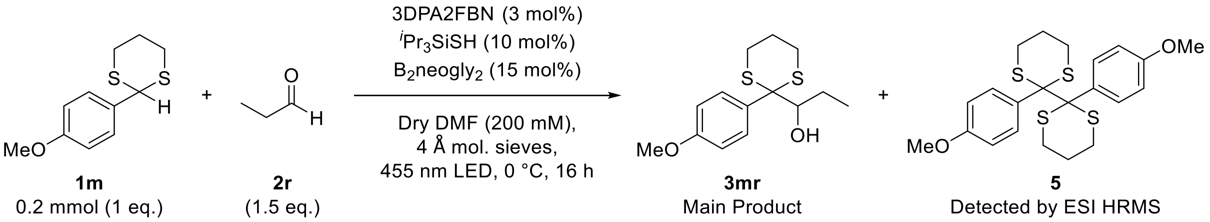

Scheme S4. Detection of the radical-radical homocoupling side-product **5** of **1m** by HRMS.

The same reaction was run without the electrophile (**2r**) in order to obtain an isolatable amount of **5**. The desired homocoupling product could be obtained in 7% NMR yield (Scheme S5 and Figure S5).

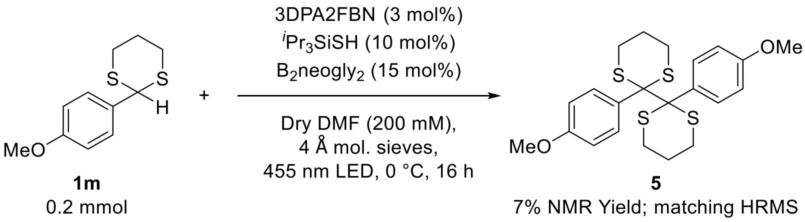

Scheme S5. Detection of the radical-radical homocoupling side-product **5** by NMR when omitting an electrophile.

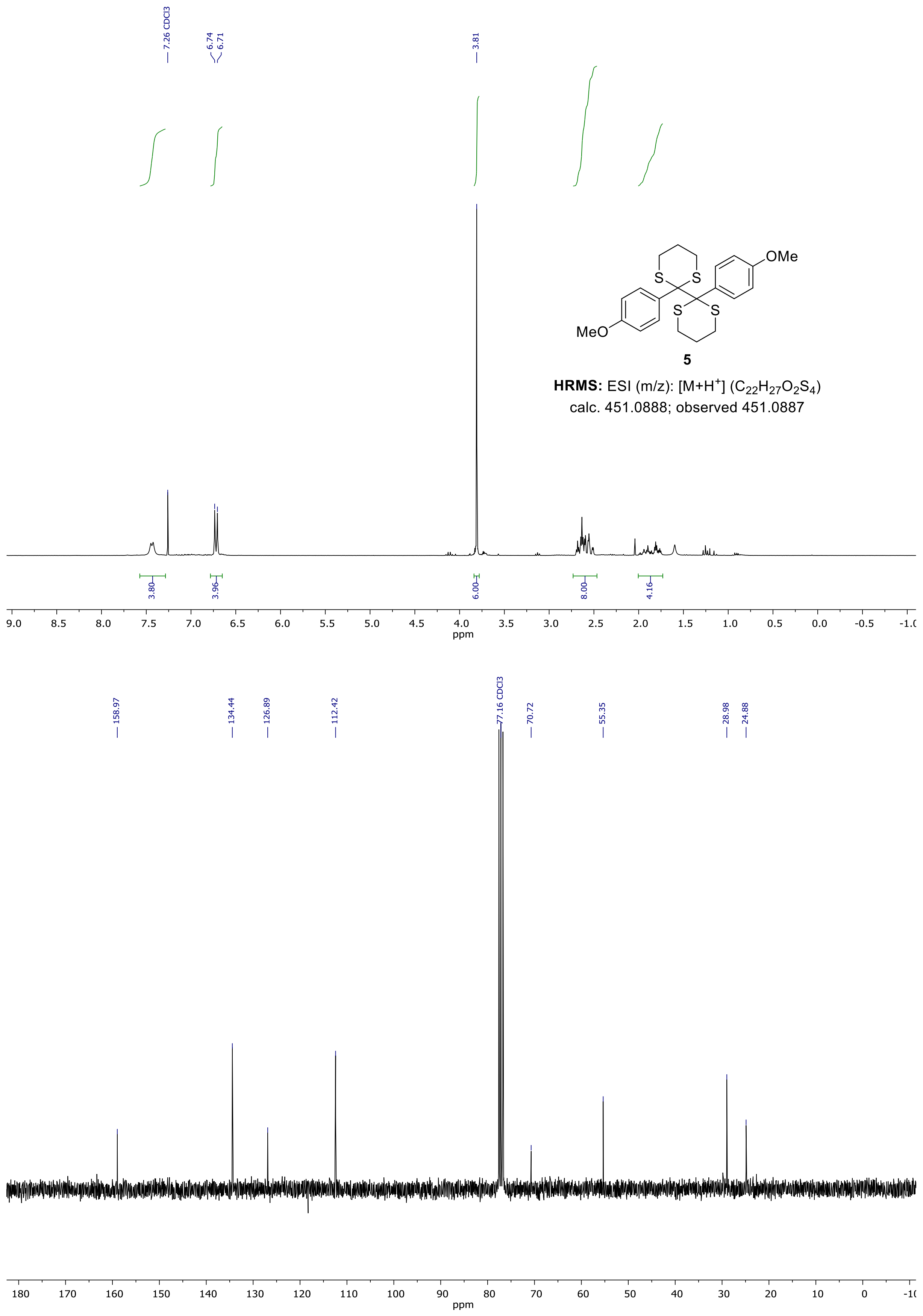

Figure S7. NMR of reaction shown in Scheme S5 after column chromatography (PE/EtOAc 5-10%). The reaction was run according to General Procedure B without second catalyst addition.

7.3 Deuterium labeling studies

tert-BuOD (6)

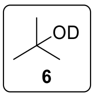

Potassium *tert*-butoxide (5.61 g, 50.0 mmol, 1 eq.) was cooled with an ice bath and D_2O (3.5 mL, 175 mmol, 3.5 eq) was added. After the addition the ice bath was removed and the suspension was stirred for 1 h leading to the formation of a clear biphasic system. The mixture was distilled and the obtained deuterated *tert*-butanol was stored over 4 Å molecular sieves.

^{1}H-NMR (300 MHz, $CDCl_3$, δ_H): 1.26 (s, 9H).

^{13}C-NMR (75 MHz, $CDCl_3$, δ_C): 69.3, 31.3.

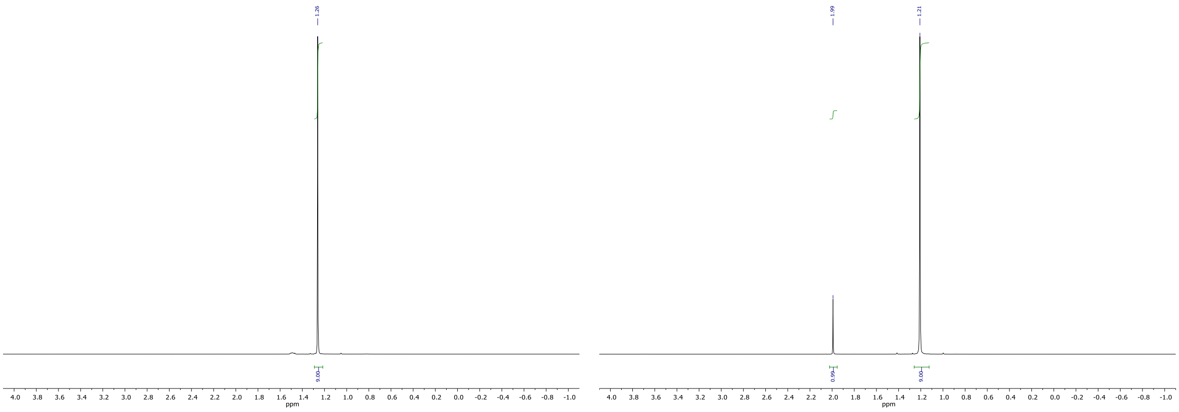

Figure S8. NMR comparison of *tert*-BuOD (**6**) (left) and *tert*-BuOH starting material (right) in $CDCl_3$.

If *tert*-BuOD was used as electrophile, the deuterated starting material could be observed (Scheme S6).

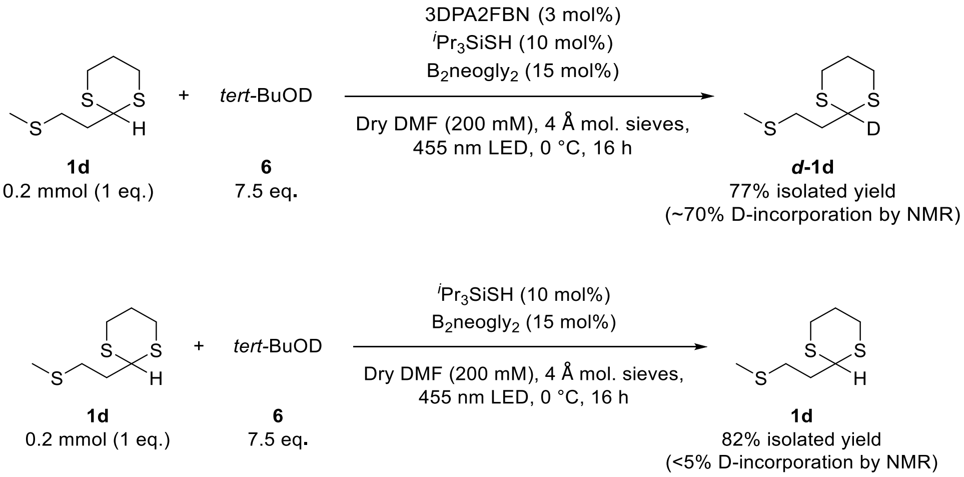

Scheme S6. Deuterium labeling experiment (upper) and control reaction without photocatalyst (lower). Reactions run as described in General Procedure B (column chromatography: PE/Diethylether 5-10%) without second catalyst addition and *tert*-BuOD as electrophile (7.5 eq.).

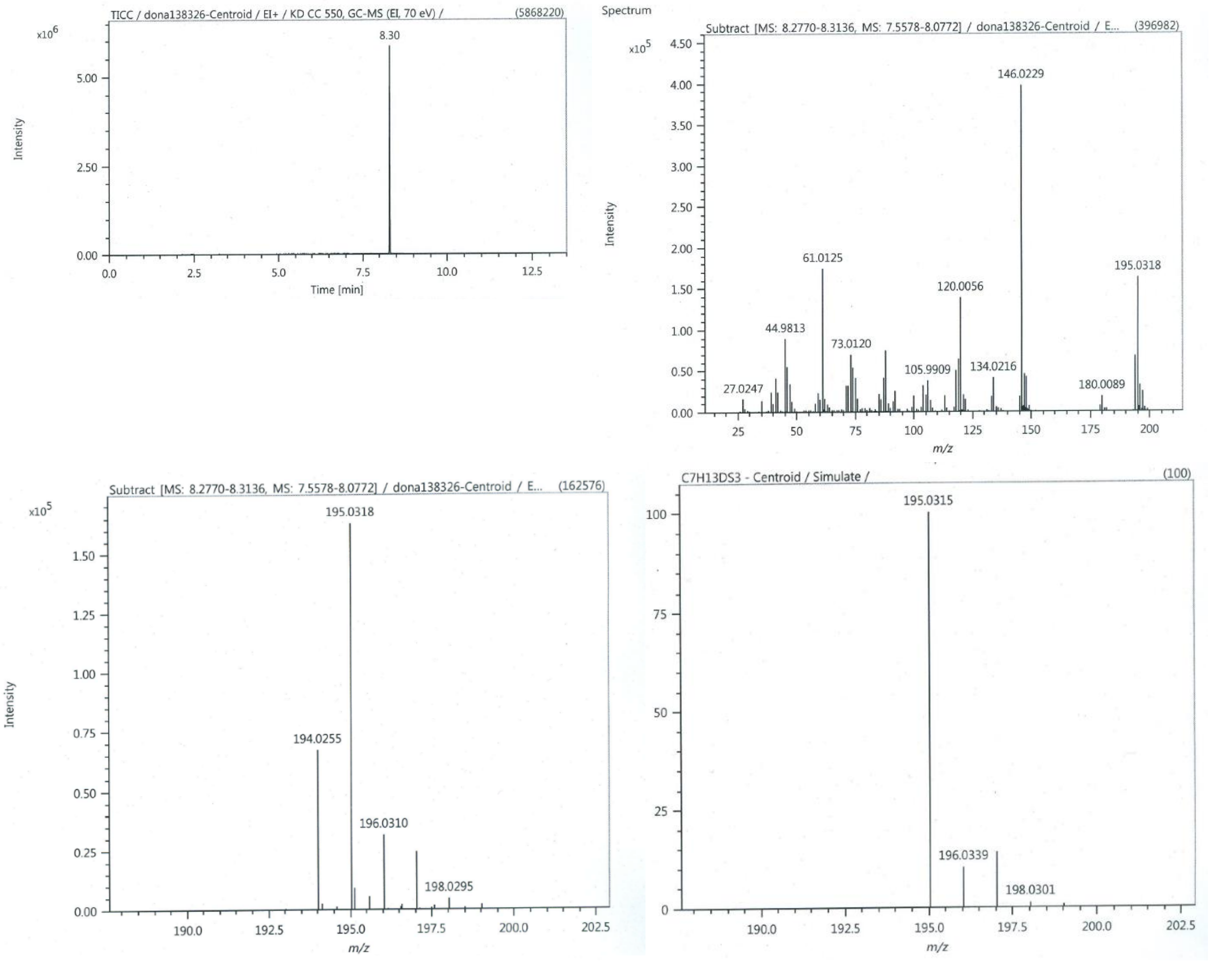

Figure S9. HRMS (EI+) of ***d*-1d** (isolated from Scheme S6 top). Top left: Total ion count; Top right: Mass spectrum; Bottom left: Zoomed in mass spectrum; Bottom right: Calculated mass spectrum of ***d*-1d** ($C_7H_{13}DS_3$).

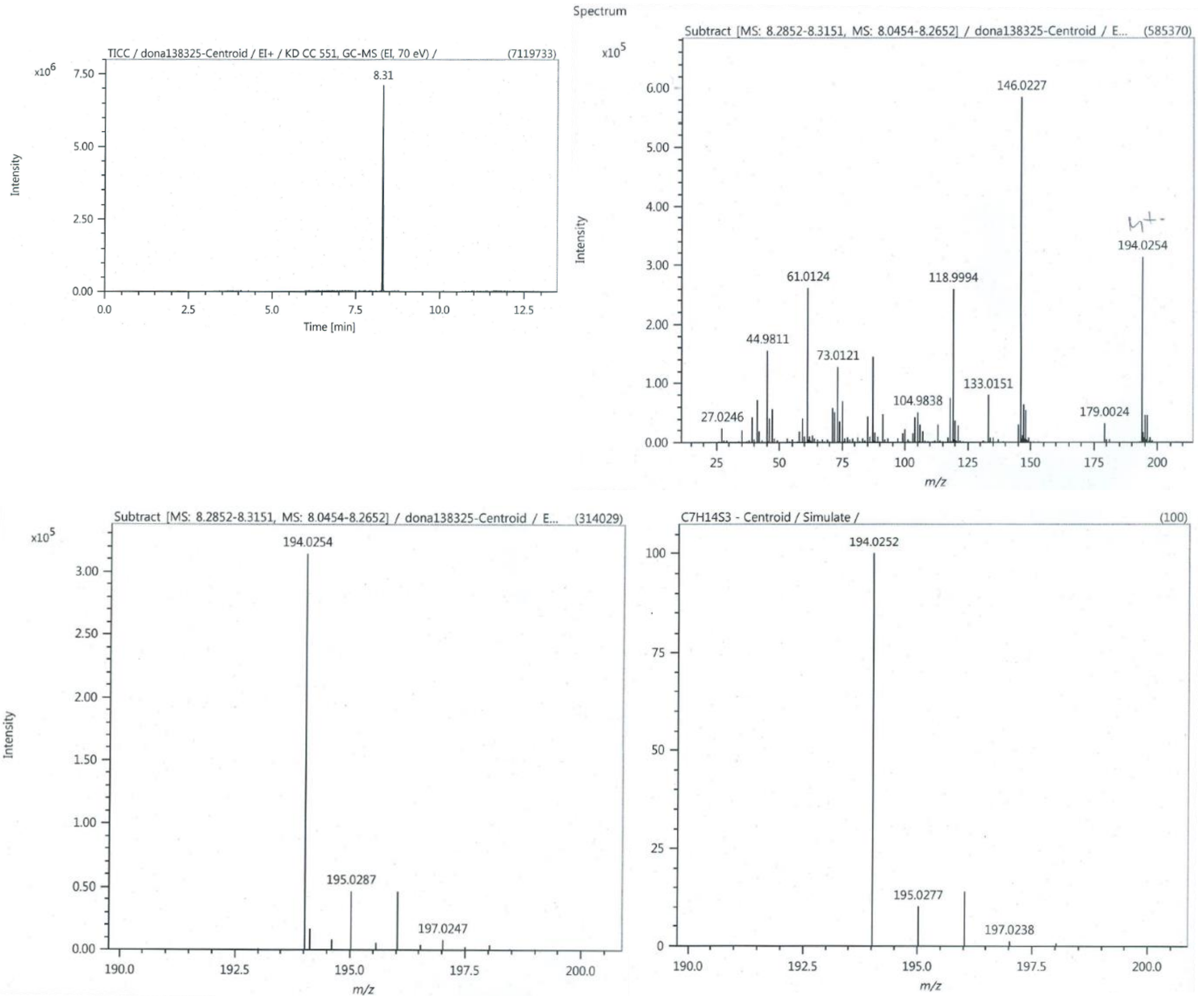

Figure S10. HRMS (EI+) of **1d** (isolated from Scheme S6 bottom). Top left: Total ion count; Top right: Mass spectrum; Bottom left: Zoomed in mass spectrum; Bottom right: Calculated mass spectrum of **1d** ($C_7H_{14}S_3$).

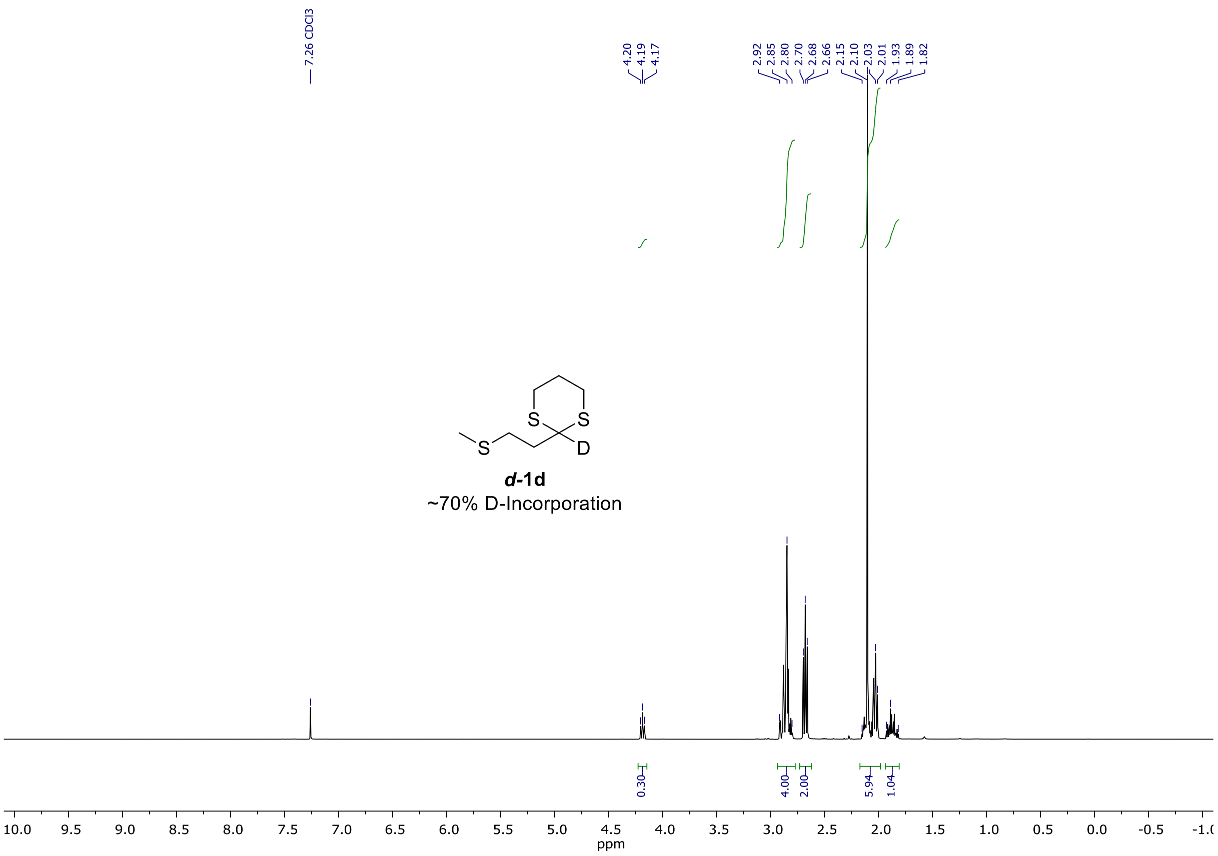

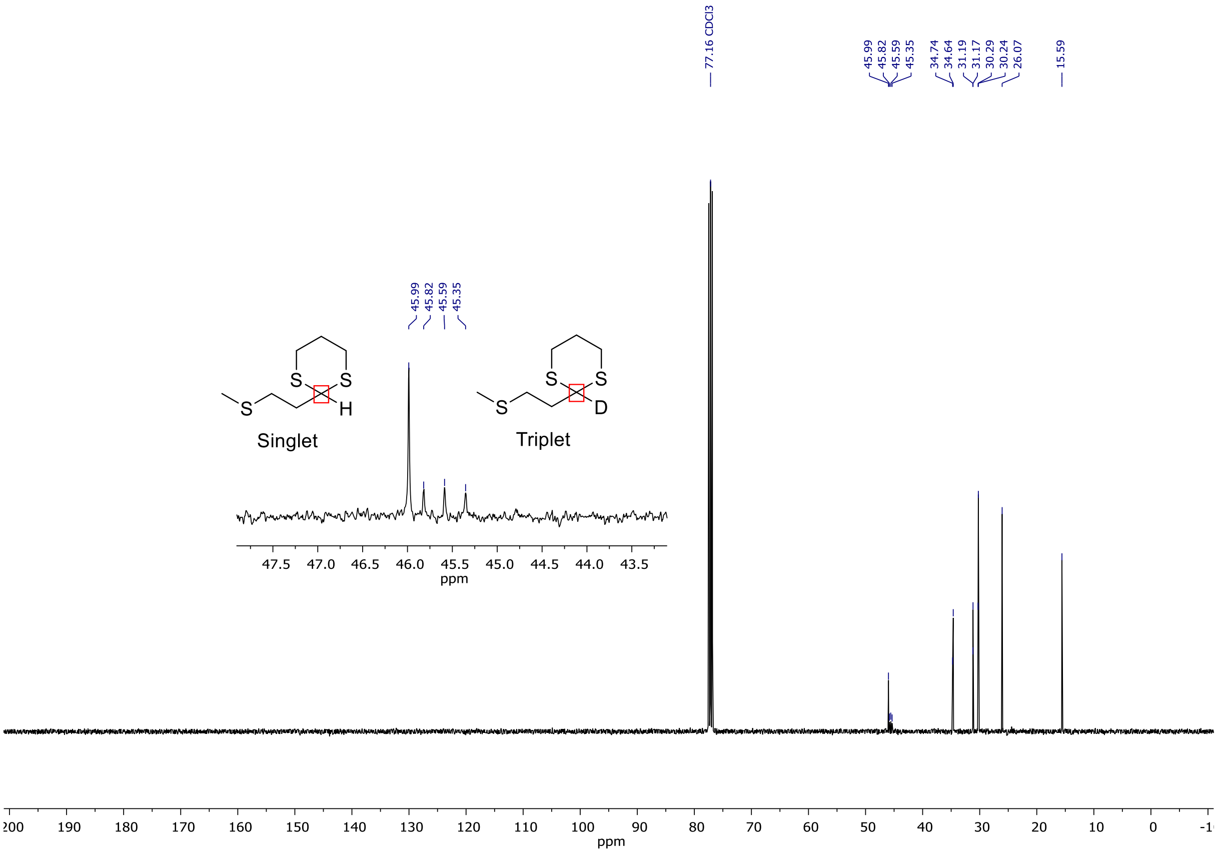

Figure S11. ^{1}H- and ^{13}C-NMR of ***d*-1d** (isolated from Scheme S6 top).

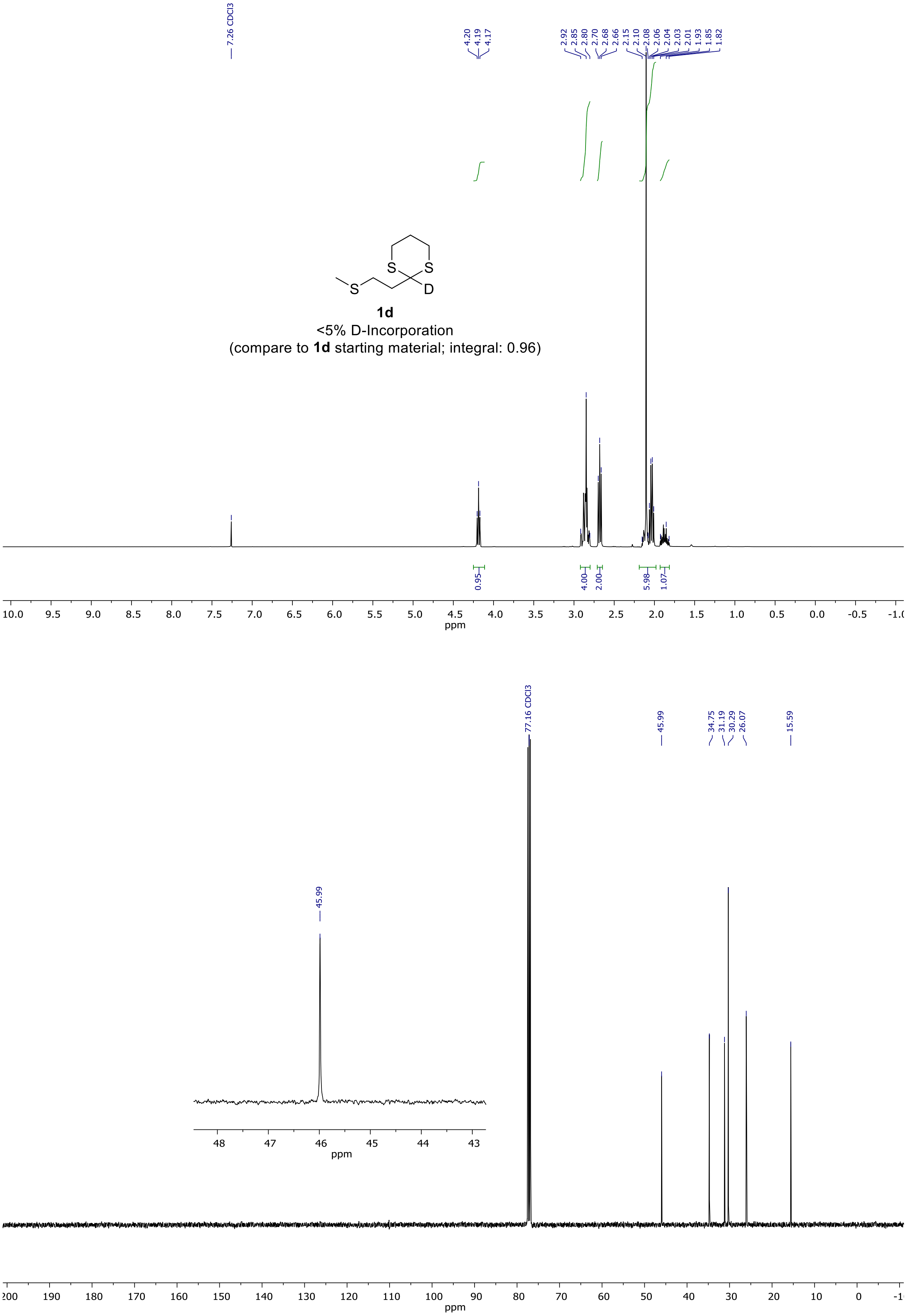

Figure S12. ^{1}H- and ^{13}C-NMR of **1d** (isolated from Scheme S6 bottom).

8. Computational analysis

All the calculations were carried out using the Gaussian 16 Rev. B.01.[27] The nature of the stationary points was confirmed by the number of imaginary frequencies found (0 for minima, 1 for transition states). All the optimizations were carried out at the M06-2X/def2-TZVP[28] level of theory. Single point energy corrections were obtained at the M06-2X/def2-TZVPP level of theory.

The bond dissociation enthalpies were calculated in vacuo. Two different conformations were evaluated for **1a**, *viz.* the conformation with the methyl substituent in equatorial and axial position. **1a** with the substituent in equatorial position was found to be more stable (the Boltzmann distribution predicts a preference > 93% for the equatorial compared to the axial position), hence only the equatorial position was taken in consideration for the thermochemical analysis.

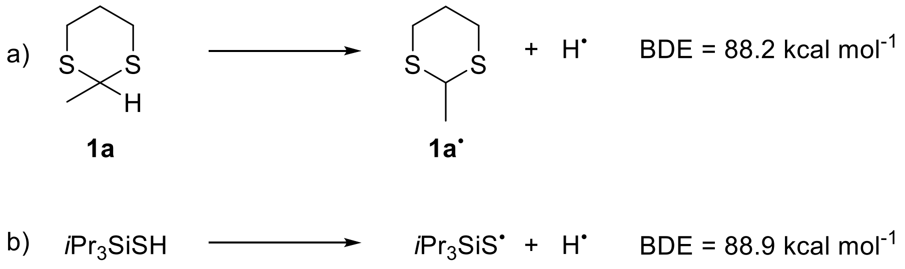

values at the M06-2X/def-TZVPP//M06-2X/def2-TZVP level

The thermochemical values confirm the feasibility of the hydrogen atom extraction from **1a** operated by the sulfur radical of the HAT catalyst.

The reduction potential of **1a•** was calculated using an implicit solvent model, *i.e.* SMD(DMF), following a procedure reported in the literature[29] using a Born-Haber cycle:

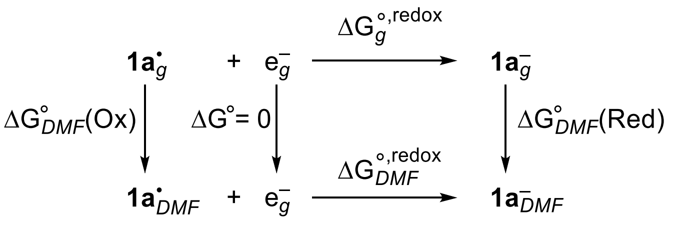

while the Nernst equation was used to determine the standard one-electron redox potential *E*°:

$$\Delta G^{o,redox}_{DMF} = -FE^{\circ}$$

where F is the Faraday constant, 23.06 kcal mol^{-1} V^{-1}.

All the values are calculated at 298.15 K. The results are referenced versus SHE (-4.28V)[29] and SCE (-4.504V), obtaining *E*° = -1.62V *vs* SHE and -1.87V *vs* SCE.

8.1 Cartesian coordinates and energies

All the energies are reported in Hartrees, if not reported otherwise. The coordinates are in Ångstroms.

1a⁻ (DMF)

C	-2.0239361824	-2.3000394535	0.0224969522
C	0.300207254	-0.8350707635	-0.0210418976
C	-2.0223028471	-0.0334165406	1.2089853467
C	-2.5733747645	-1.4551222098	1.1674361316
H	-2.2451614222	-1.8177944724	-0.9344171105
H	-2.494948451	-3.2848327561	0.024293967
H	-2.4922020143	0.5268183964	2.0195439153
H	-2.2435065053	0.4783751701	0.2675377512
H	-2.3619366908	-1.952715892	2.117729362
H	-3.6623733879	-1.3983317806	1.0604448826
S	-0.2363571532	0.0454068041	1.4804887874
S	-0.2382417489	-2.5700848404	0.1113806122
C	1.8216106379	-0.8488604769	0.003207245
H	2.212735364	-1.3904374789	-0.8643696147
H	2.2138613108	0.1722446656	-0.0463660398
H	2.2687470309	-1.3182348415	0.8992663293

Energy= -992.36705
Zero-point correction= 0.129936 (Hartree/Particle)
Thermal correction to Gibbs Free Energy= 0.098044
Sum of electronic and zero-point Energies= -992.237114
Sum of electronic and thermal Energies= -992.229469
Sum of electronic and thermal Enthalpies= -992.228525
Sum of electronic and thermal Free Energies= -992.269006
Imaginary Frequencies 0
Energy(SMD(DMF)-M06-2X/def2-TZVPP)= -992.3707599

1a⁻ (gas)

C	-2.009289258	-2.3050353661	0.0246786954
C	0.2895555451	-0.8873601342	0.0788597387
C	-2.0076546303	-0.0323806398	1.2143299762
C	-2.5848189905	-1.4474274374	1.1527489303
H	-2.1602757685	-1.8000793183	-0.9342536969
H	-2.5128663805	-3.2754105659	0.0022424778
H	-2.5101133908	0.5395779666	1.9992630248
H	-2.1586582091	0.4680588264	0.2530355414
H	-2.396616081	-1.9473839336	2.1075843983
H	-3.6728460779	-1.3762122107	1.0182000238
S	-0.2360807909	0.0000100659	1.5680398024
S	-0.2379675979	-2.6161644779	0.1985849568
C	1.8015838972	-0.8423067457	-0.0092877847
H	2.131791002	-1.3703582217	-0.9102991228
H	2.1329172587	0.1986329024	-0.0890331344
H	2.3541599026	-1.2882571801	0.841922793

Energy= -992.2770847
Zero-point correction= 0.129135 (Hartree/Particle)
Thermal correction to Gibbs Free Energy= 0.097047
Sum of electronic and zero-point Energies= -992.147950
Sum of electronic and thermal Energies= -992.140166
Sum of electronic and thermal Enthalpies= -992.139222
Sum of electronic and thermal Free Energies= -992.180038
Imaginary Frequencies 0
Energy (SMD(DMF)-M06-2X/def2-TZVPP)= -992.2809822

H• (gas)

H	2.439555	-0.056186	-0.653484

Energy= -0.4981385
Zero-point correction= 0.000000 (Hartree/Particle)
Thermal correction to Gibbs Free Energy= -0.010654
Sum of electronic and zero-point Energies= -0.498139
Sum of electronic and thermal Energies= -0.496722
Sum of electronic and thermal Enthalpies= -0.495778
Sum of electronic and thermal Free Energies= -0.508793
Imaginary Frequencies 0
Energy (SMD(DMF)-M06-2X/def2-TZVPP)= -0.4981385

1a (gas)-Me equatorial

C	-2.039811119	-2.2783960734	0.0039399891
C	0.3216753247	-0.8534995219	0.0143259858
C	-2.0382050289	-0.0304615234	1.1807758204
C	-2.5517158089	-1.4637605813	1.1839278263
H	-2.3064033588	-1.7922275718	-0.9384285176
H	-2.4877031941	-3.2717627479	-0.0019446571
H	-2.4849851857	0.540870322	1.99403262
H	-2.3047813466	0.4672725707	0.2444597601
H	-2.2721797417	-1.9532398188	2.1185261448
H	-3.6445836892	-1.4364680273	1.1332837759
H	-0.0579056119	-0.378168414	-0.8931009718
S	-0.2523746533	0.1160602875	1.4315780374
S	-0.2542907782	-2.5699850554	0.0253694292
C	1.8417591282	-0.8541216808	0.0134422474
H	2.2168597376	-1.4035252948	-0.8502425812
H	2.2179818835	0.1682711988	-0.0273698193
H	2.2199480023	-1.3287321082	0.9194955506

Energy= -992.9072184
Zero-point correction= 0.144292 (Hartree/Particle)

Thermal correction to Gibbs Free Energy= 0.112104
Sum of electronic and zero-point Energies= -992.762927
Sum of electronic and thermal Energies= -992.755194
Sum of electronic and thermal Enthalpies= -992.754249
Sum of electronic and thermal Free Energies= -992.795114
Imaginary Frequencies 0
Energy (SMD(DMF)-M06-2X/def2-TZVPP)= -992.9113244

1a (gas)-Me axial

C	-2.0608739527	-2.294472408	0.034495728
C	0.2736289403	-0.7869330023	-0.1118787253
C	-2.0595008585	-0.046405804	1.2118154126
C	-2.514879694	-1.4988633047	1.2508057992
H	-2.4298707601	-1.8352791569	-0.8853495211
H	-2.4583294246	-3.3086523642	0.0696168854
H	-2.4560056776	0.5027181706	2.0656378182
H	-2.4284703552	0.4485054913	0.3106733935
H	-2.142864121	-1.9751456209	2.1598235253
H	-3.6084543789	-1.5164773272	1.2857117929
S	-0.2670785079	0.1668062875	1.3368054731
S	-0.2687153304	-2.5203591889	-0.0704862624
H	1.3549670755	-0.8534962231	0.0139727152
C	-0.0448915763	-0.0958226155	-1.4311497592
H	0.3730605586	0.9109689216	-1.4370057599
H	0.3720892205	-0.6648073554	-2.2622522429
H	-1.1208466277	-0.0138379898	-1.5864331227

Energy= -992.9046896
Zero-point correction= 0.144443 (Hartree/Particle)
Thermal correction to Gibbs Free Energy= 0.112461
Sum of electronic and zero-point Energies= -992.760246
Sum of electronic and thermal Energies= -992.752600
Sum of electronic and thermal Enthalpies= -992.751656
Sum of electronic and thermal Free Energies= -992.792229
Imaginary Frequencies 0
Energy (SMD(DMF)-M06-2X/def2-TZVPP)= -992.9088596

1a$^{\bullet}$ (DMF)

C	-2.0604360867	-2.264734645	-0.0070787318
C	0.3901678372	-0.9835647265	0.2625190301
C	-2.0588317205	-0.0291951915	1.1631390932
C	-2.5605129096	-1.4620423198	1.1806287284
H	-2.3182585976	-1.7640649193	-0.9421631142
H	-2.5046941927	-3.2599677033	-0.0200522572
H	-2.5019610163	0.5490939524	1.9738428141
H	-2.3166659954	0.4541992655	0.2190116089

H	-2.2801275993	-1.9518165244	2.1158904571
H	-3.6523945503	-1.4364813434	1.1332951571
S	-0.2711629415	0.1335001039	1.4172421882
S	-0.2731018198	-2.5682686385	0.0029706507
C	1.8673347667	-0.8723064304	0.0479494982
H	2.161219674	-1.3946234972	-0.8645360547
H	2.1623464198	0.1748384817	-0.0429848626
H	2.4398991621	-1.3066623343	0.8769424145

Energy= -992.2692153
Zero-point correction= 0.131074 (Hartree/Particle)
Thermal correction to Gibbs Free Energy= 0.097688
Sum of electronic and zero-point Energies= -992.138141
Sum of electronic and thermal Energies= -992.130159
Sum of electronic and thermal Enthalpies= -992.129215
Sum of electronic and thermal Free Energies= -992.171528
Imaginary Frequencies 0
Energy (SMD(DMF)-M06-2X/def2-TZVPP)= -992.2727565

1a• (gas)

C	-2.0535607906	-2.2643878409	-0.0085717507
C	0.3846481288	-0.9938622258	0.2822150261
C	-2.0519418302	-0.0281749655	1.161992166
C	-2.5618008776	-1.4604200658	1.177535806
H	-2.3006714017	-1.7598151886	-0.9446976181
H	-2.504591109	-3.2560313951	-0.0282562565
H	-2.5018660478	0.5536162988	1.965894973
H	-2.2990228158	0.4538211658	0.21403649
H	-2.2764331504	-1.9493907204	2.1112599966
H	-3.6543009013	-1.4364790863	1.1333020561
S	-0.2687794451	0.1248159854	1.4361668506
S	-0.270723823	-2.5788521007	0.0208312034
C	1.8584933087	-0.8715860672	0.0465973565
H	2.1377982688	-1.3927332078	-0.8700837238
H	2.1389335299	0.1783576118	-0.0476441911
H	2.4466393862	-1.3009746676	0.866038236

Energy= -992.2585533
Zero-point correction= 0.131259 (Hartree/Particle)
Thermal correction to Gibbs Free Energy= 0.097831
Sum of electronic and zero-point Energies= -992.127294
Sum of electronic and thermal Energies= -992.119296
Sum of electronic and thermal Enthalpies= -992.118352
Sum of electronic and thermal Free Energies= -992.160722
Imaginary Frequencies 0
Energy (SMD(DMF)-M06-2X/def2-TZVPP)= -992.2621928

$^{i}Pr_3SiSH$ (gas)

Si	-0.0108530228	-0.0232631964	0.3146760391
S	-0.2190628239	-0.2351146314	2.4666137582
H	1.0566766664	0.0634657406	2.7442033033
C	-1.6726784763	-0.6577487105	-0.3173445266
C	-1.7016185439	-0.9644160111	-1.8187773625
C	-2.8053220349	0.3022935862	0.0643400406
H	-1.8347988048	-1.6008860426	0.2177643183
H	-0.9955137097	-1.7495589344	-2.0900074119
H	-2.6981111891	-1.3018921566	-2.1160386265
H	-1.4644801998	-0.0842950134	-2.4197270364
H	-2.8266166745	0.5073125355	1.1362221688
H	-2.7001138684	1.258987204	-0.4529968218
H	-3.7751554471	-0.1156682867	-0.2165903946
C	0.2617105327	1.8093178165	-0.0615820111
C	0.0346563572	2.2044954694	-1.5252580975
C	1.6408163259	2.2704716133	0.4194799671
H	-0.4917335134	2.3242806874	0.5452706641
H	-0.9932523447	2.0257630037	-1.8419729421
H	0.2388778243	3.2695885606	-1.6647429357
H	0.6901551443	1.6571141309	-2.2051427616
H	1.7918695877	2.0629385156	1.4806073119
H	2.441706782	1.7764640211	-0.1359734991
H	1.7602755318	3.3470820913	0.2750242349
C	1.4506922844	-1.1000899815	-0.2128387336
C	1.9376520512	-0.8508097068	-1.6448383514
C	1.1442643044	-2.5847889811	0.0095835604
H	2.2658725497	-0.82246083	0.466973926
H	2.3063808729	0.1659566455	-1.7820418459
H	2.7562926328	-1.5328451626	-1.8896117307
H	1.1461907898	-1.017894211	-2.3784392609
H	0.8213539298	-2.7818001684	1.0328341138
H	0.3535510534	-2.928010077	-0.6619531442
H	2.0285784666	-3.1945526076	-0.1914650499

Energy= -1043.8809824
Zero-point correction= 0.293676 (Hartree/Particle)
Thermal correction to Gibbs Free Energy= 0.252029
Sum of electronic and zero-point Energies= -1043.587306
Sum of electronic and thermal Energies= -1043.570915
Sum of electronic and thermal Enthalpies= -1043.569971
Sum of electronic and thermal Free Energies= -1043.628954
Imaginary Frequencies 1
Energy (SMD(DMF)-M06-2X/def2-TZVPP)= -1043.8900532

$^{i}Pr_3SiS^{\bullet}$ (gas)

Si	0.0004516528	0.0306338675	0.3234299714
S	-0.0202615816	0.3283561333	2.4629032199
C	1.7662411184	0.4117025591	-0.2322166333
C	1.9381070332	0.6011085866	-1.7429975432
C	2.7319272491	-0.6514790751	0.3023861006
H	2.0079387119	1.3620960545	0.2562094678
H	1.3608995654	1.447660619	-2.116348139
H	2.9882503962	0.7899867612	-1.9820729199
H	1.6313903597	-0.2825870308	-2.3053409466
H	2.6605660253	-0.7578787626	1.3876249263
H	2.5285482989	-1.6295995633	-0.1405552939
H	3.7656159496	-0.3919828247	0.0609922621
C	-0.4998615986	-1.7629955701	-0.0001803081
C	-0.2266286845	-2.2698775237	-1.420350477
C	-1.9603060794	-1.9960027715	0.3991102883
H	0.1260132353	-2.3451556583	0.6870191142
H	0.8375454724	-2.2634563247	-1.6581125486
H	-0.5799236258	-3.2988469367	-1.5288906714
H	-0.7385677905	-1.6699131295	-2.1754370499
H	-2.1546042221	-1.6692373268	1.4222656181
H	-2.643283264	-1.4562422445	-0.2611518843
H	-2.2135039407	-3.0568614806	0.3315887596
C	-1.2664853981	1.2873192307	-0.312102889
C	-1.7024979019	1.0251391212	-1.7588218849
C	-0.7490620644	2.7203951391	-0.1508147463
H	-2.1479437662	1.1693927808	0.3288025139
H	-2.19708544	0.0604790568	-1.8716300592
H	-2.4058642325	1.7963749291	-2.0840534842
H	-0.8554072115	1.0465193606	-2.4476051625
H	-0.4582535095	2.9358619363	0.8781423509
H	0.1223521904	2.8968879419	-0.7859661573
H	-1.517127185	3.4399490354	-0.4449402239

Energy= -1043.2363556
Zero-point correction= 0.285971 (Hartree/Particle)
Thermal correction to Gibbs Free Energy= 0.243683
Sum of electronic and zero-point Energies= -1042.950385
Sum of electronic and thermal Energies= -1042.934113
Sum of electronic and thermal Enthalpies= -1042.933169
Sum of electronic and thermal Free Energies= -1042.992673
Imaginary Frequencies 0
Energy (SMD(DMF)-M06-2X/def2-TZVPP)= -1043.2447725

9. NMR-spectra

4CzIPN, ^{1}H- and ^{13}C-NMR ($CDCl_3$):

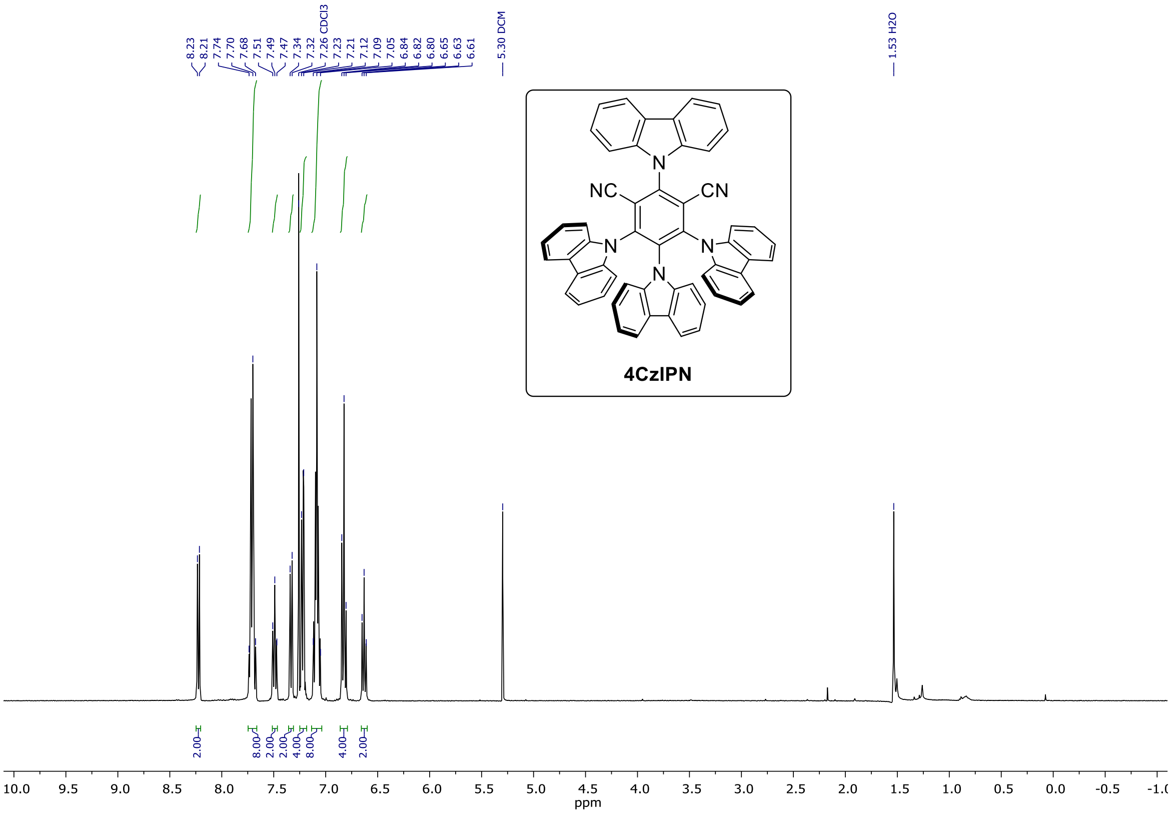

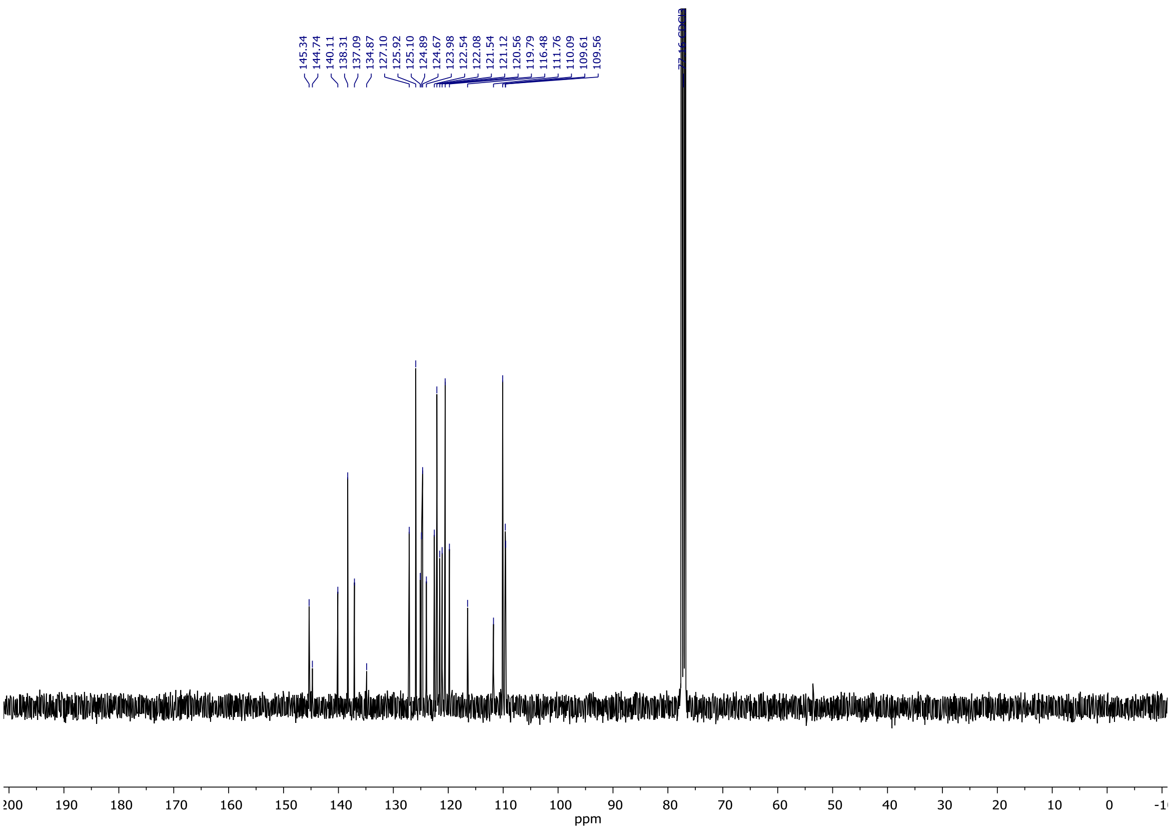

3DPA2FBN, ^{1}H- and ^{19}F-NMR ($CDCl_3$):

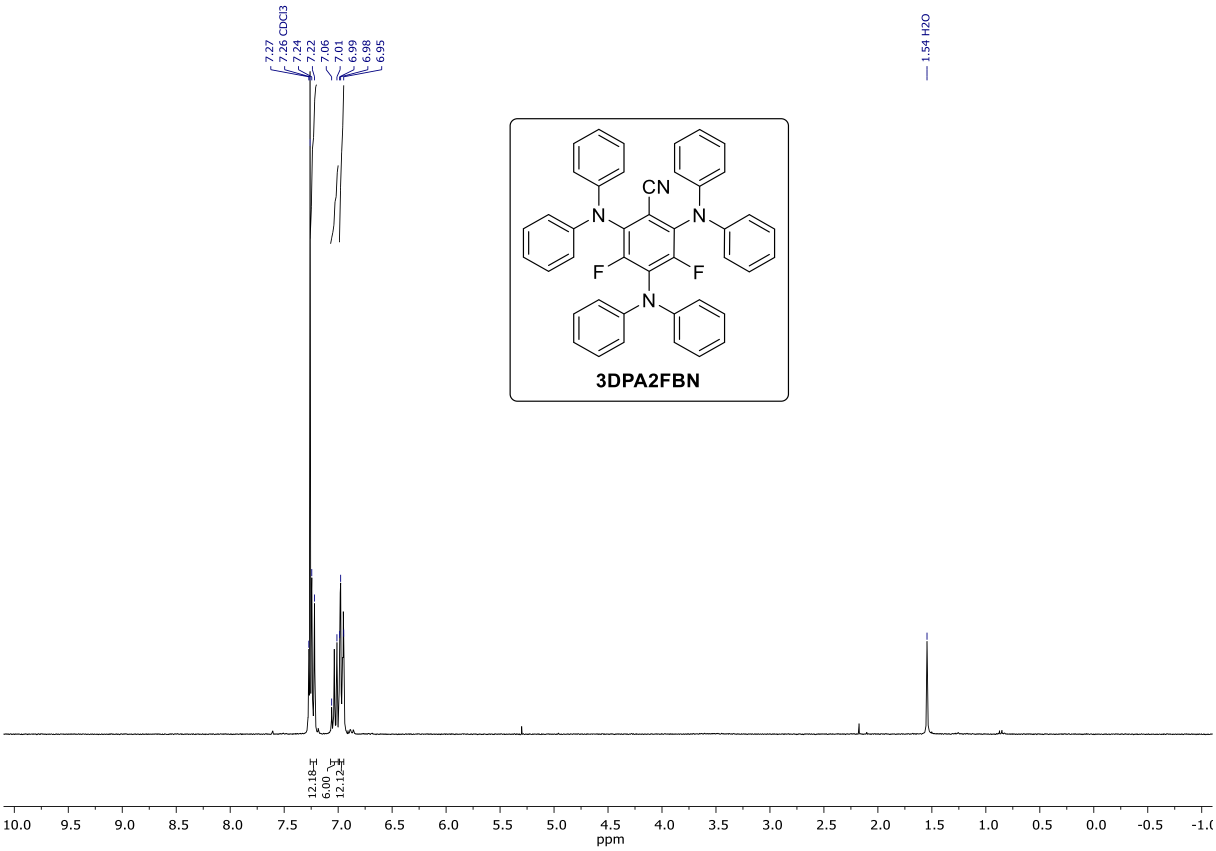

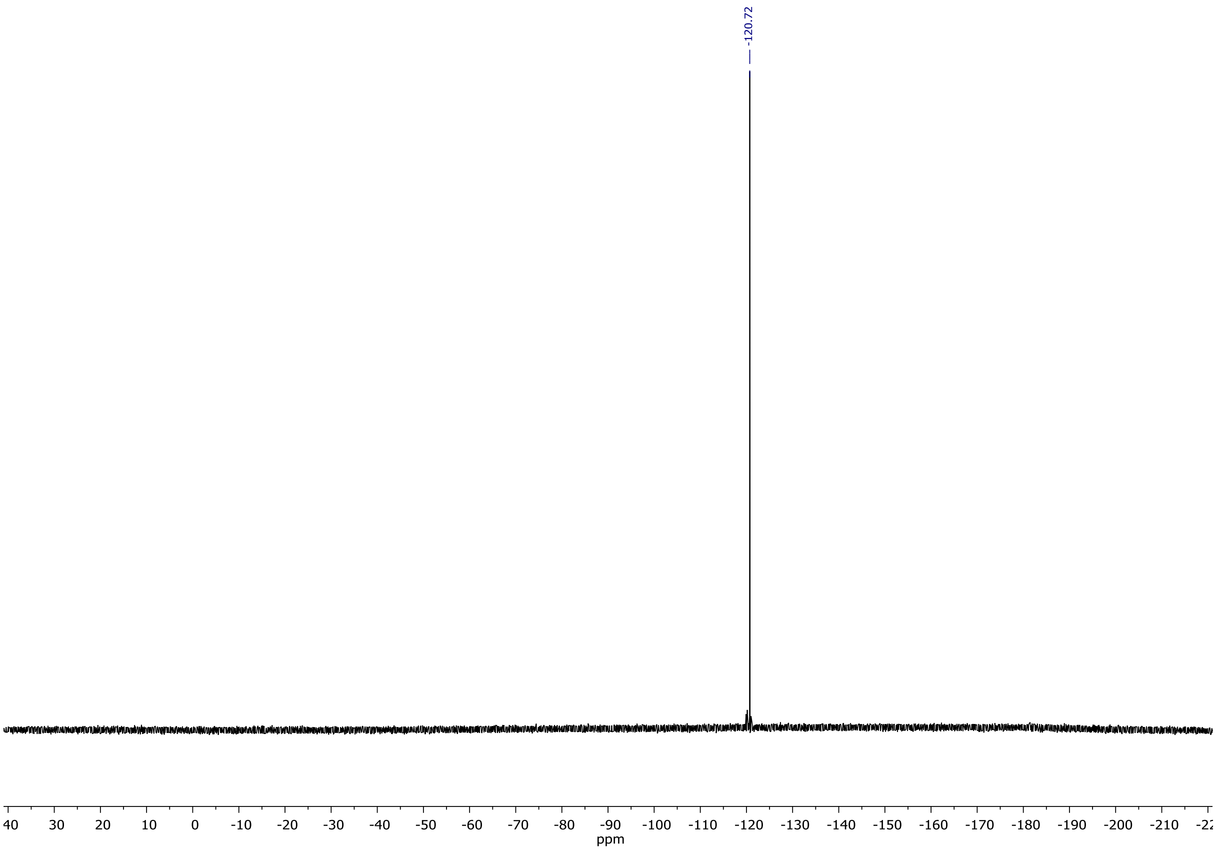

3DPAFIPN, ^{1}H- and ^{19}F-NMR ($CDCl_3$):

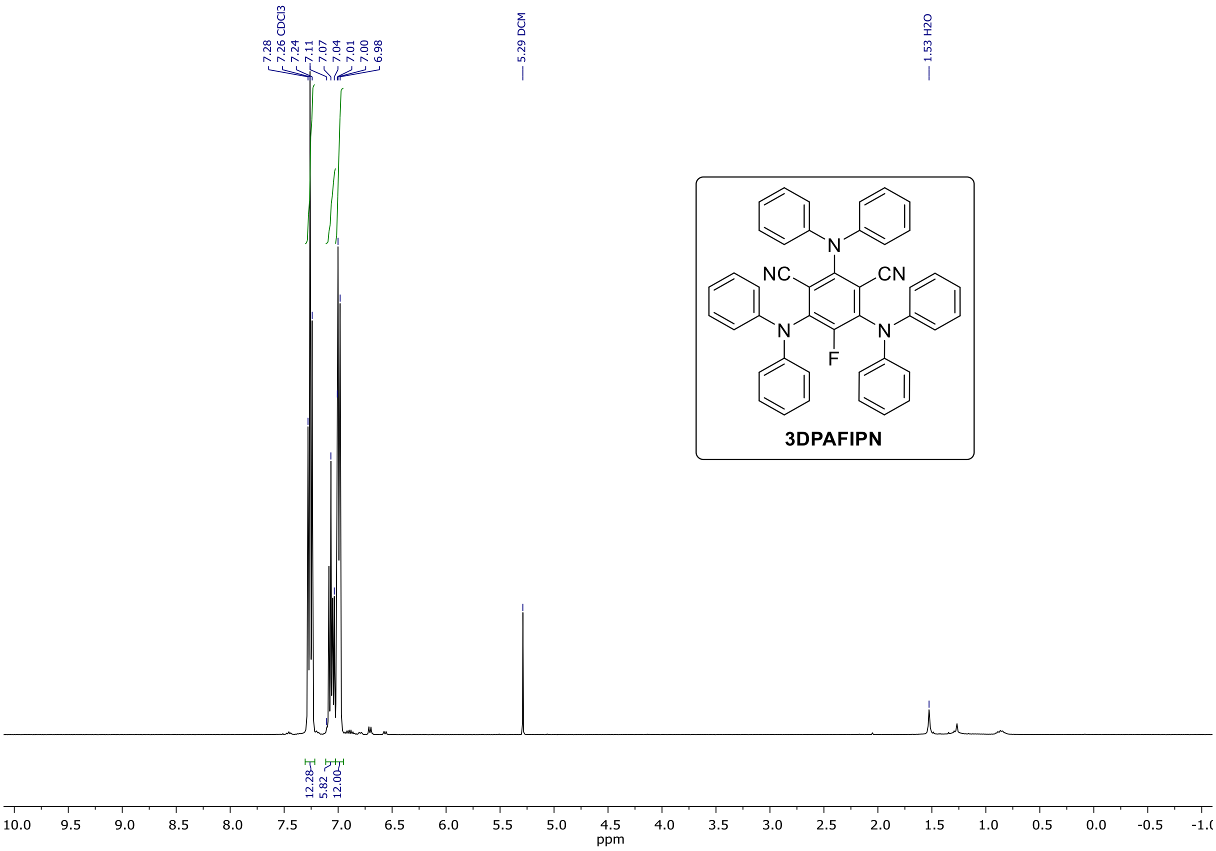

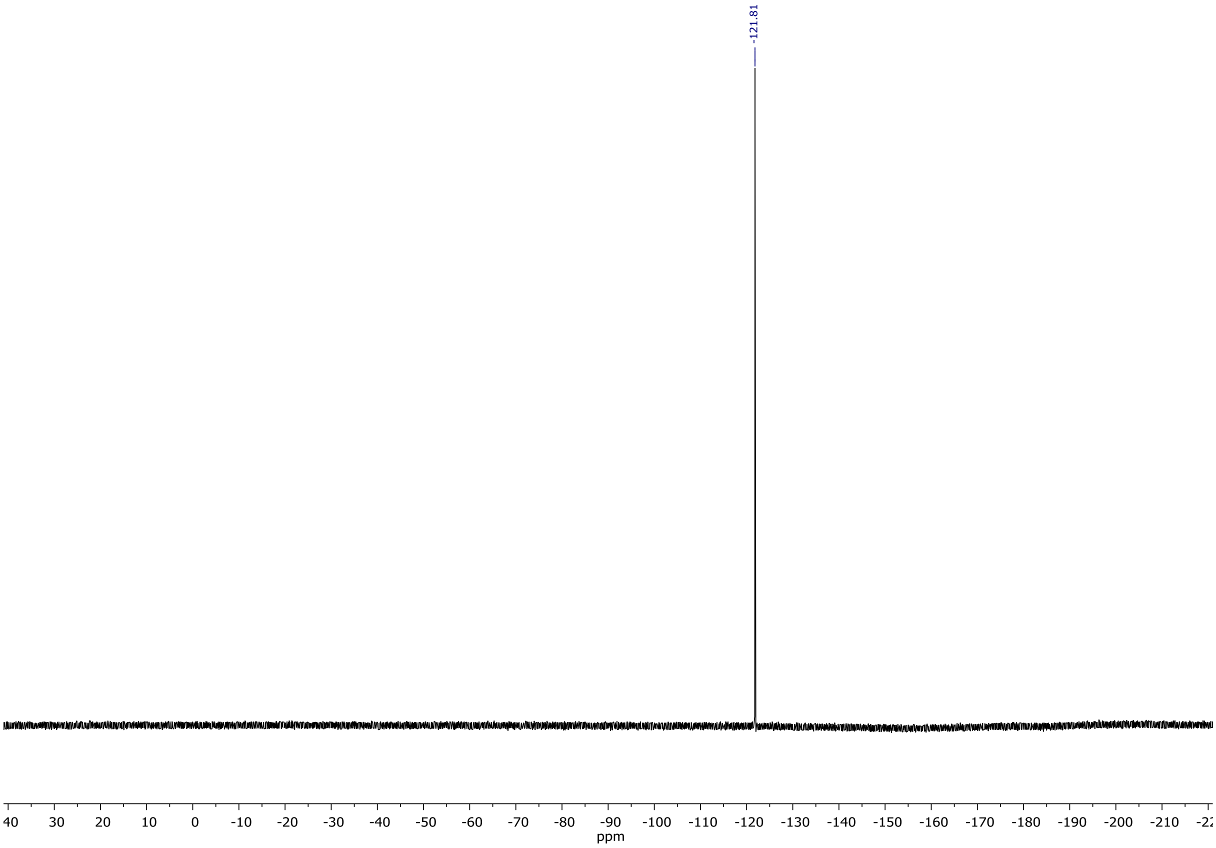

4Cz(*p*H)BN, ^{1}H- and ^{19}C-NMR ($CDCl_3$):

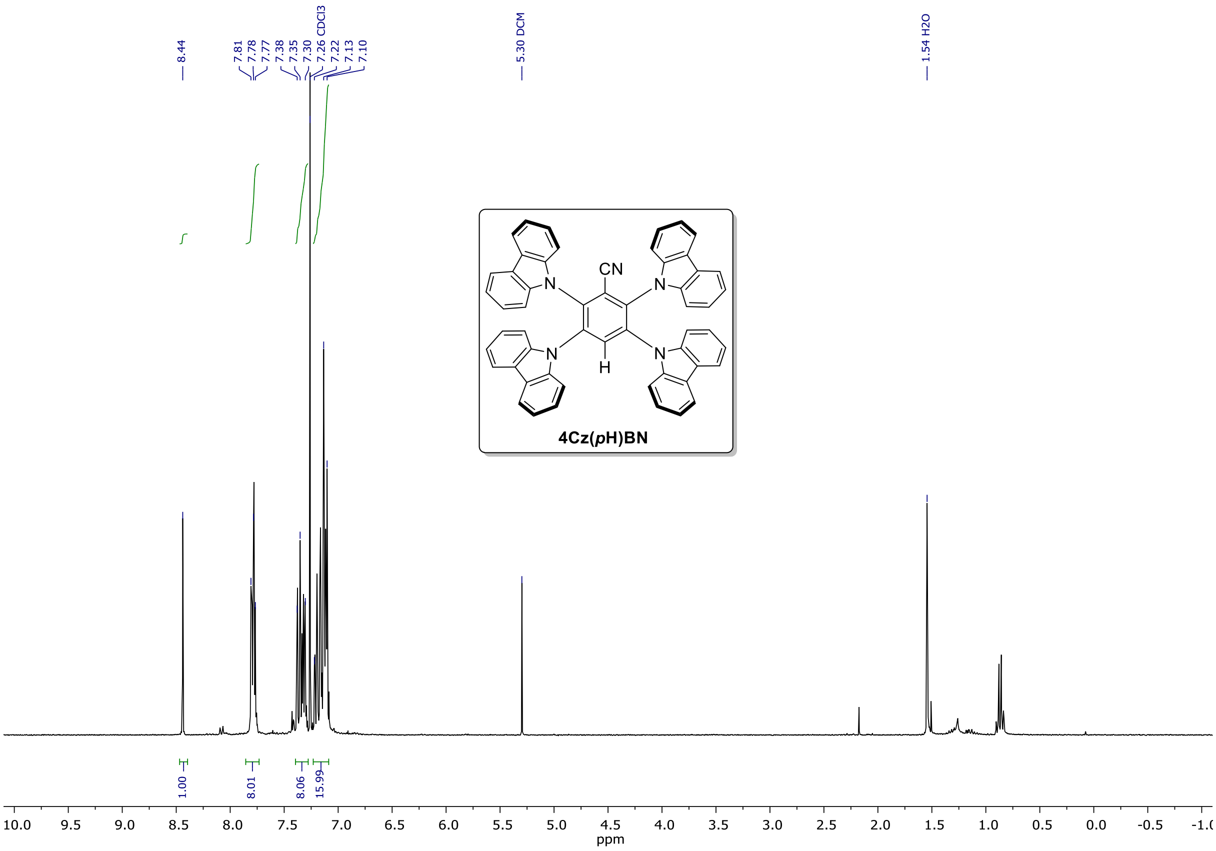

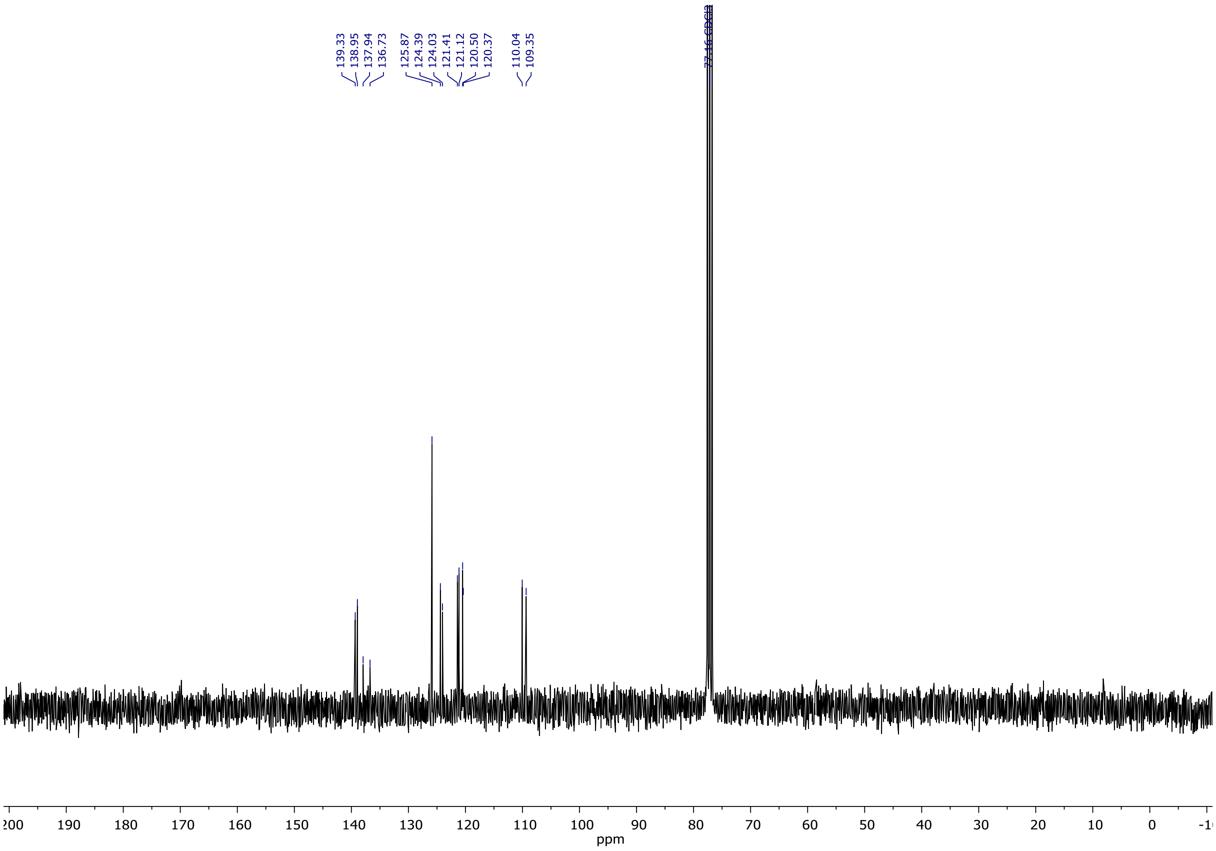

Compound **1c**, ^{1}H- and ^{13}C-NMR ($CDCl_3$):

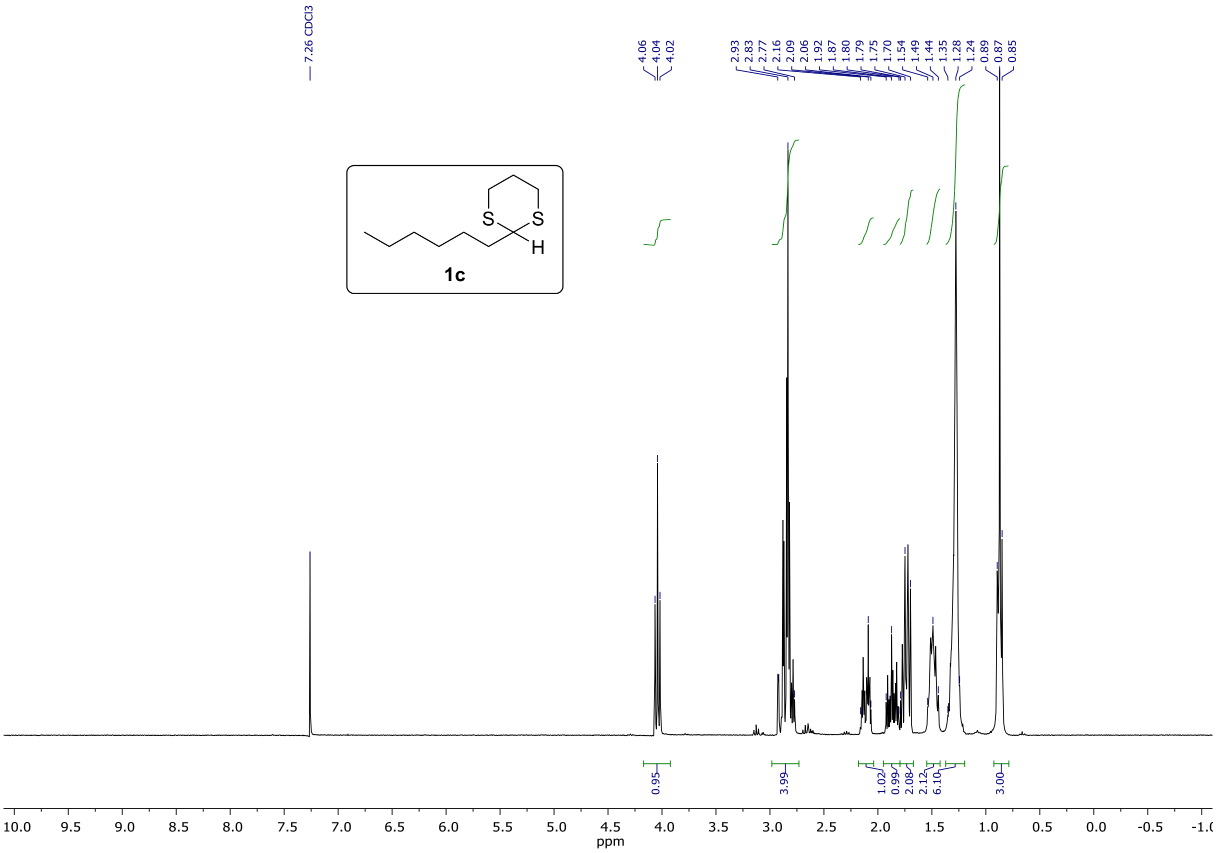

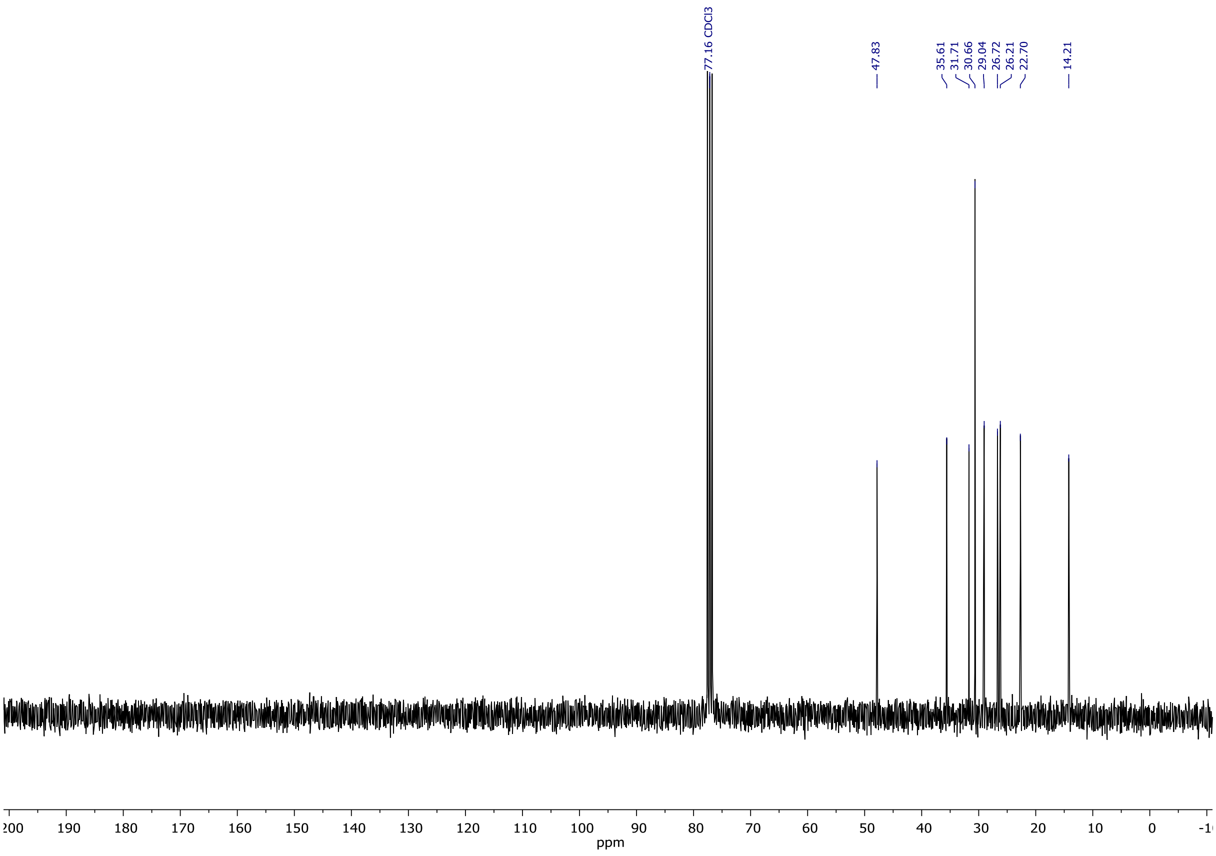

Compound **1d**, ^{1}H- and ^{13}C-NMR ($CDCl_3$):

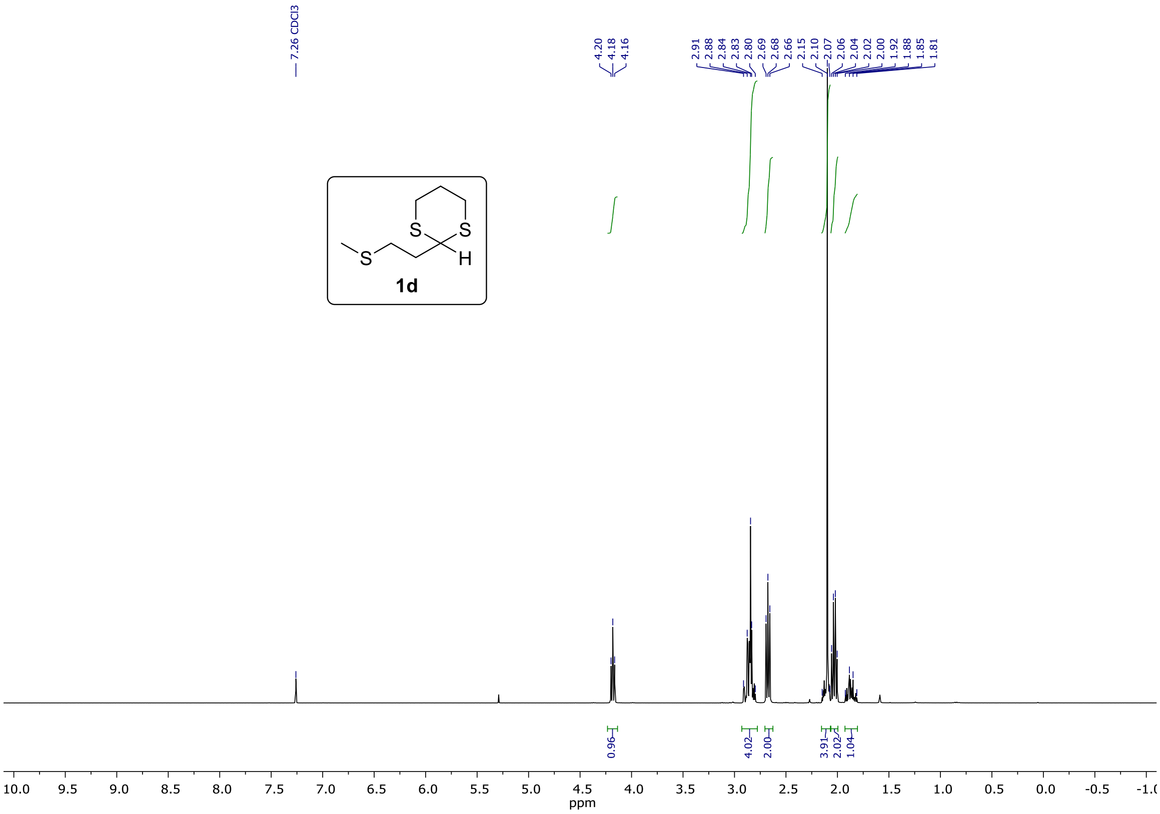

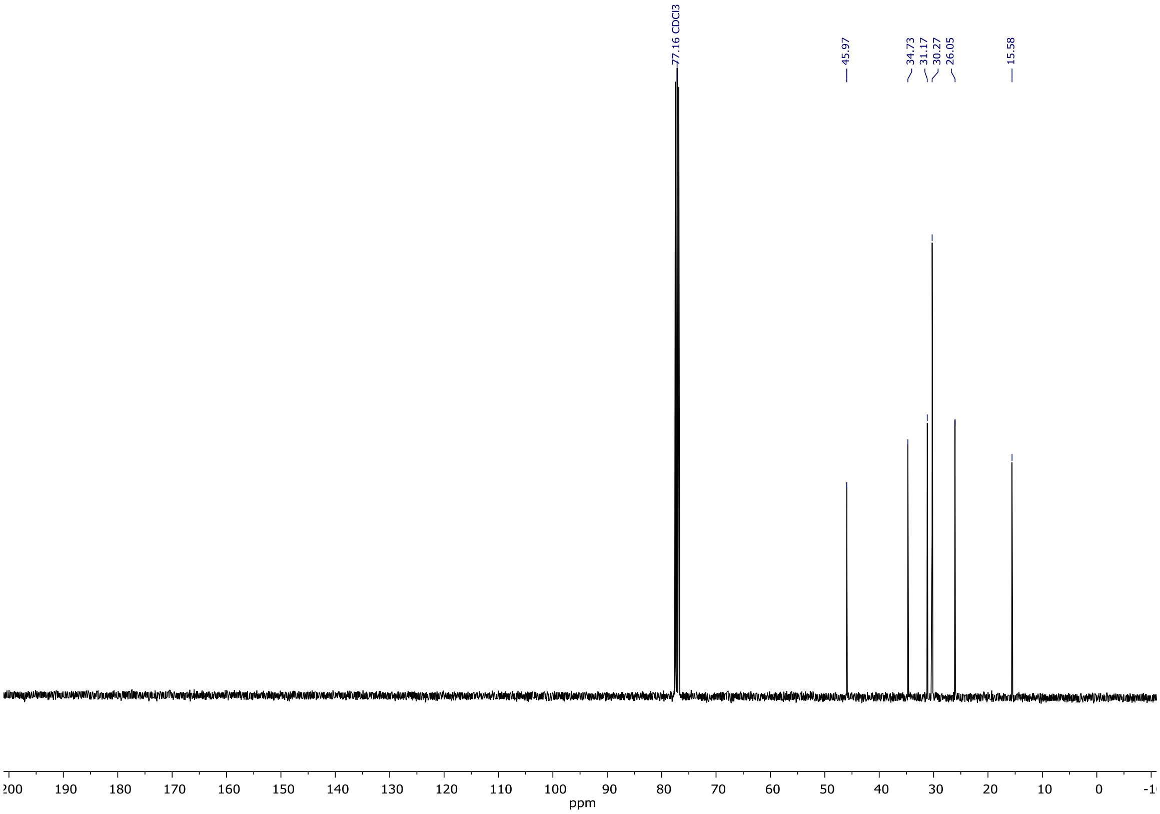

Compound **1e**, ^{1}H- and ^{13}C-NMR ($CDCl_3$):

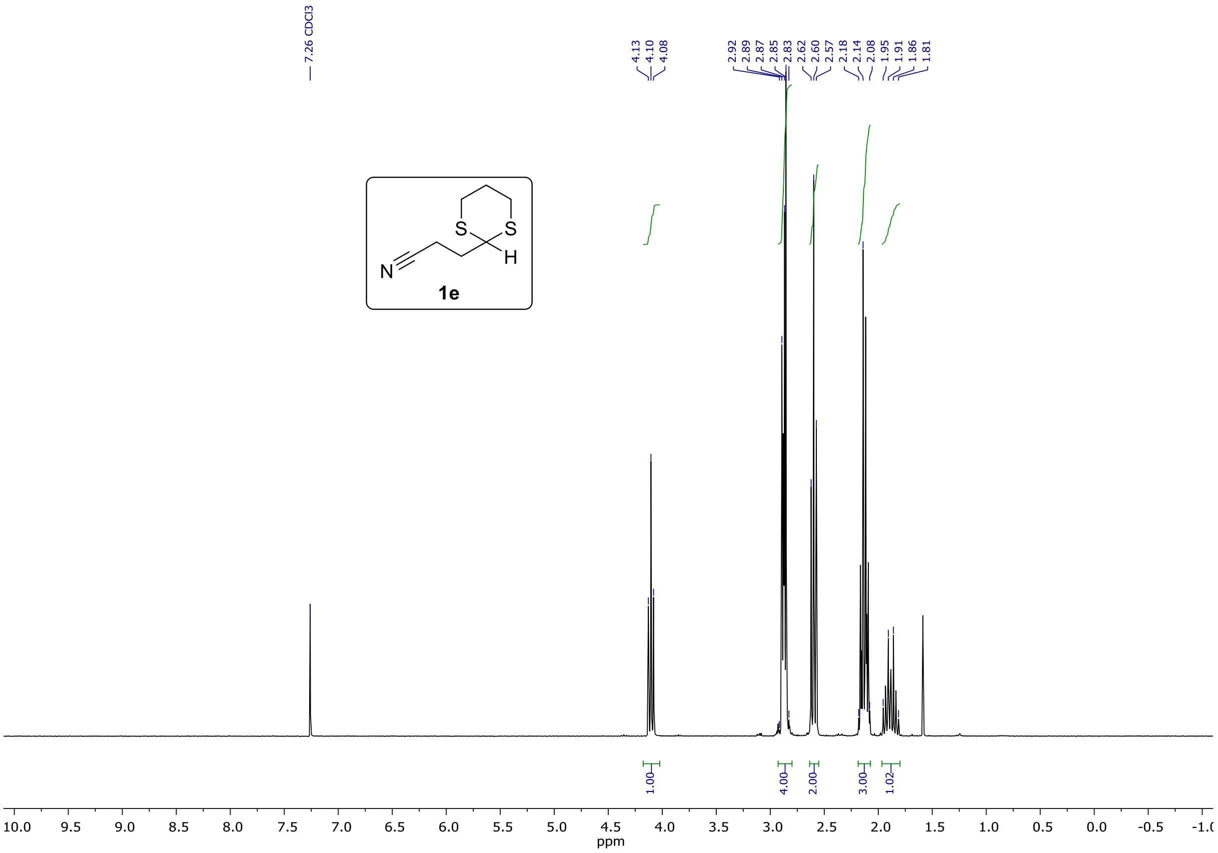

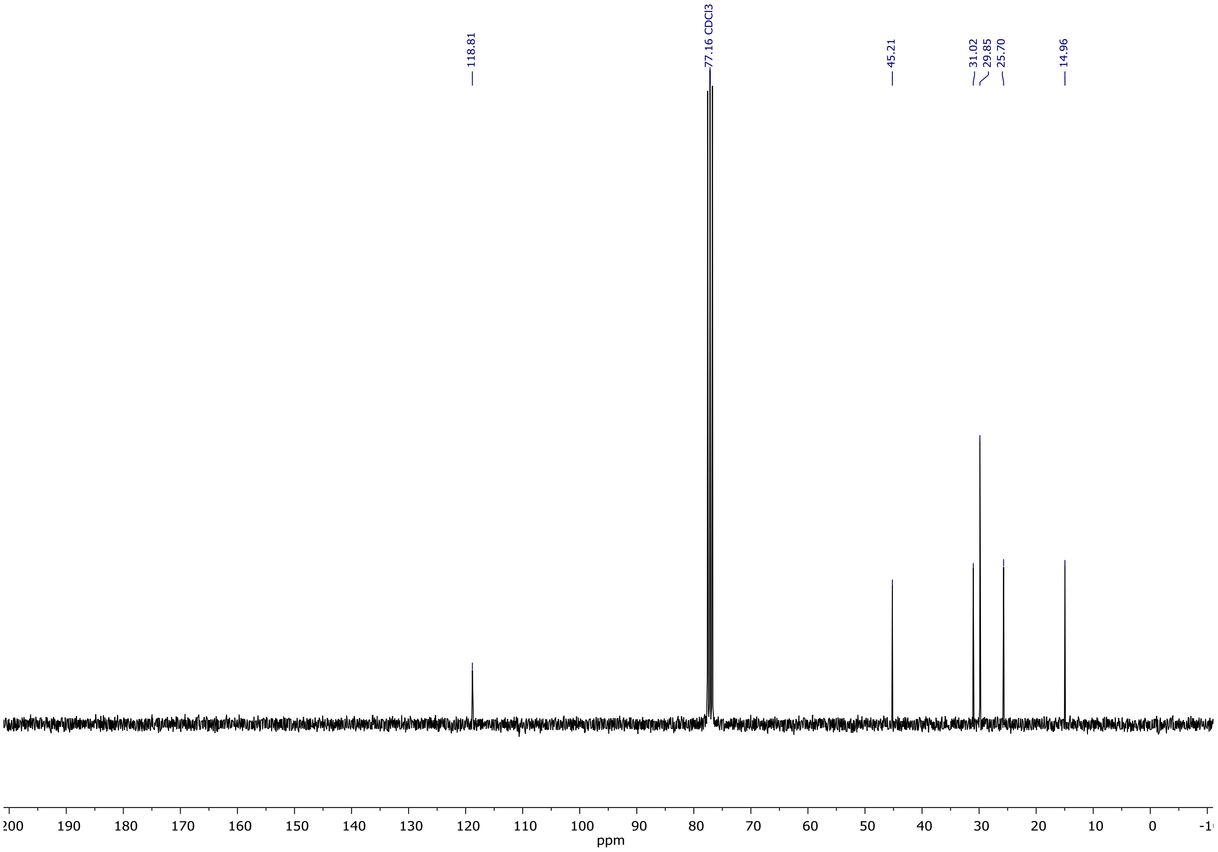

Compound **1f**, ^{1}H- and ^{13}C-NMR ($CDCl_3$):

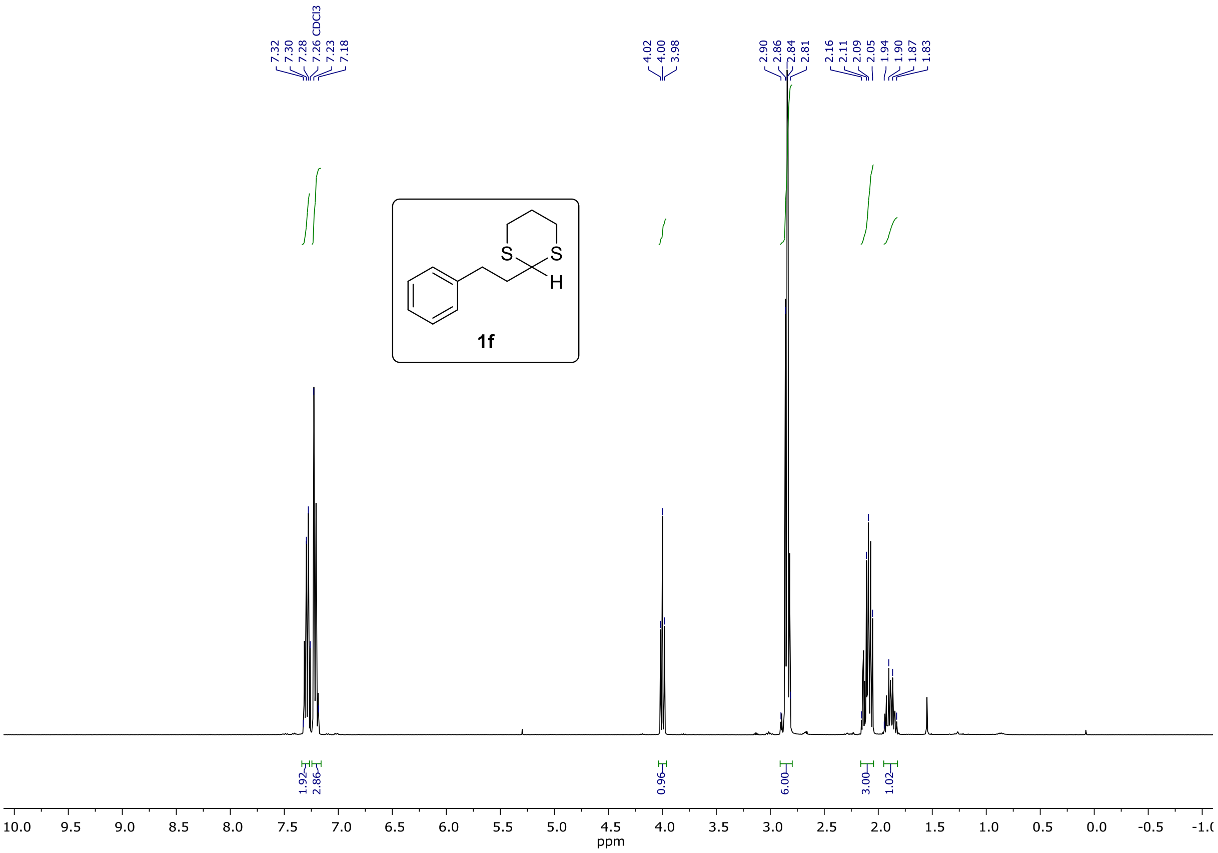

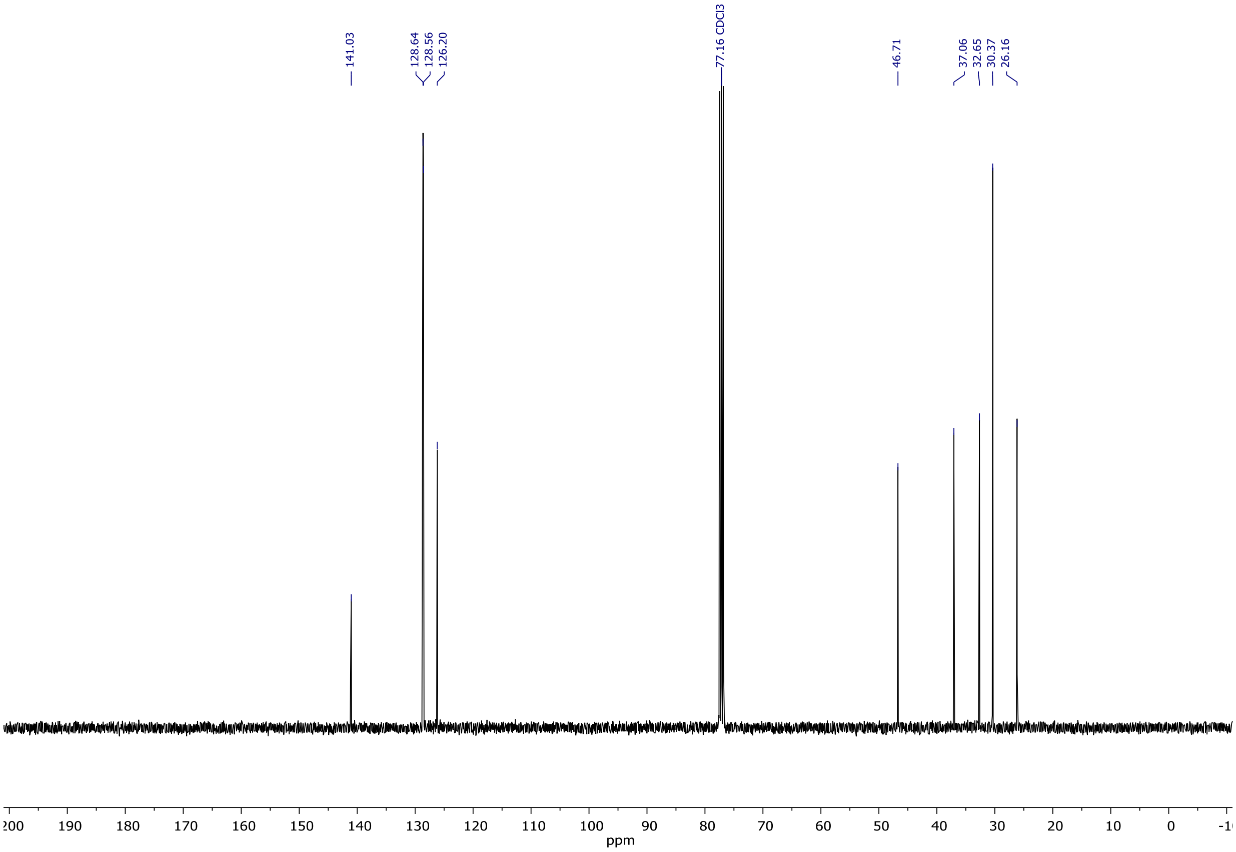

Compound **1g**, ^{1}H- and ^{13}C-NMR ($CDCl_3$):

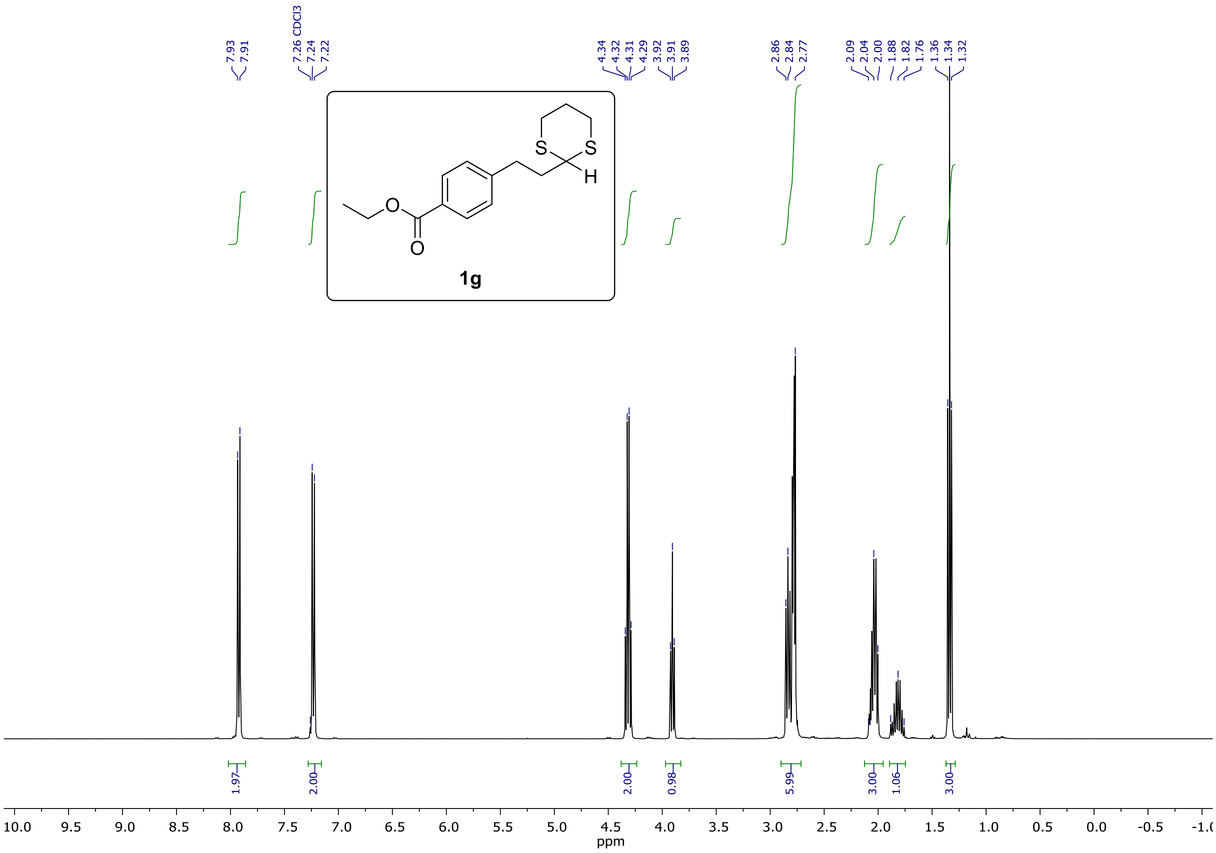

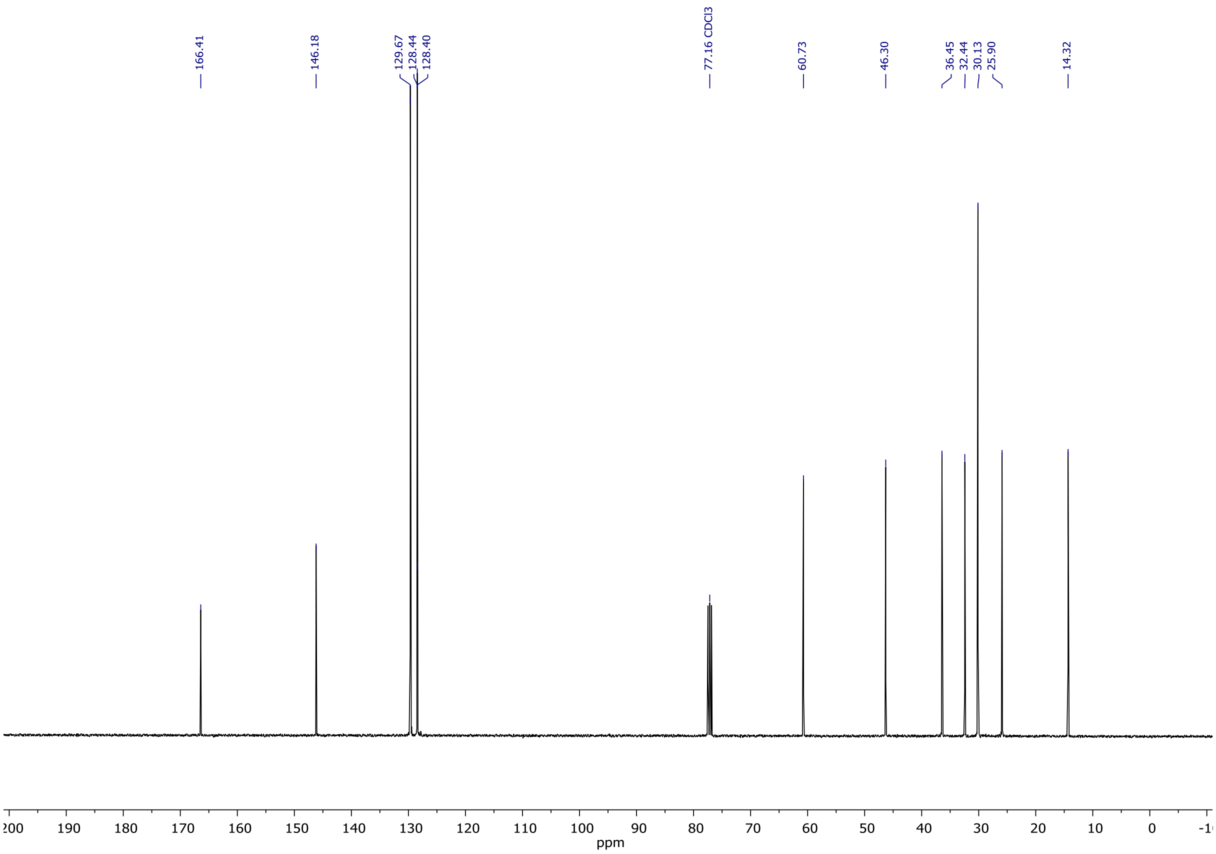

Compound **1h**, ^{1}H- and ^{13}C-NMR ($CDCl_3$):

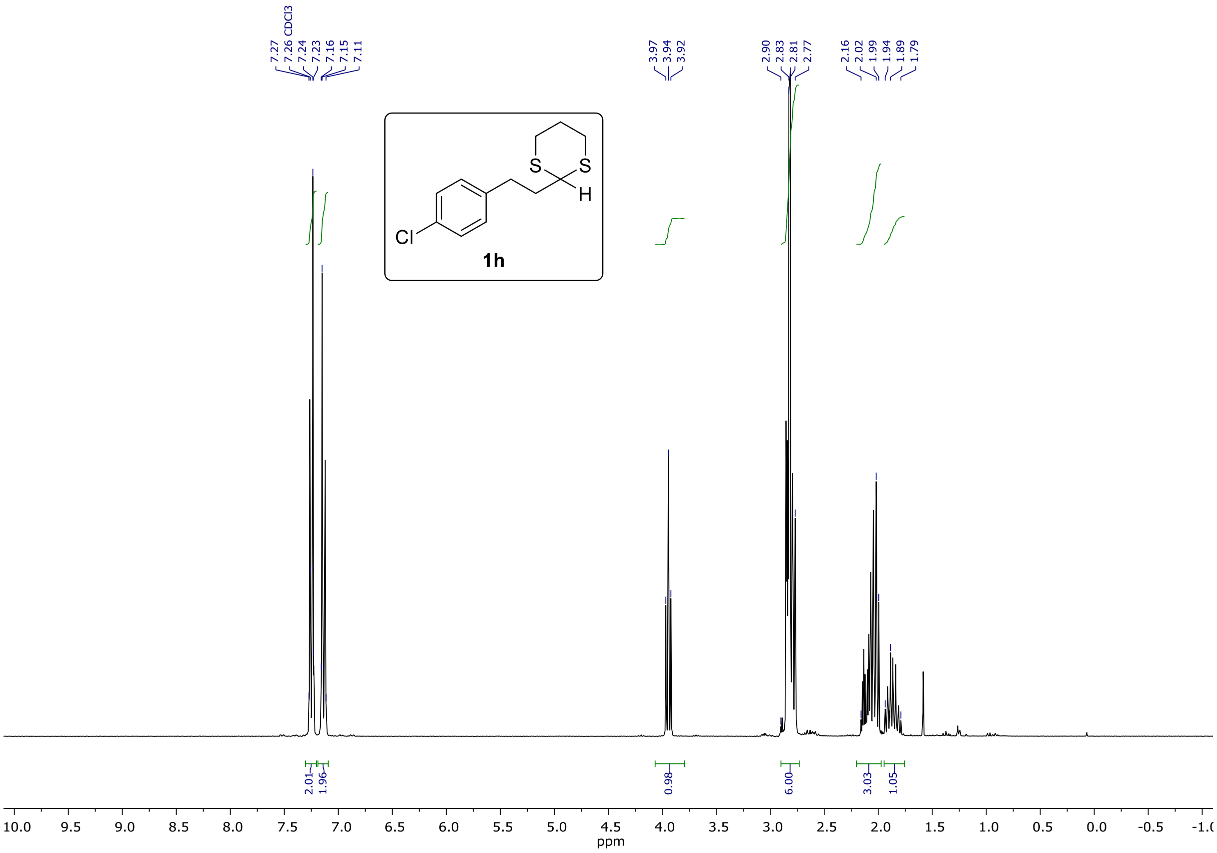

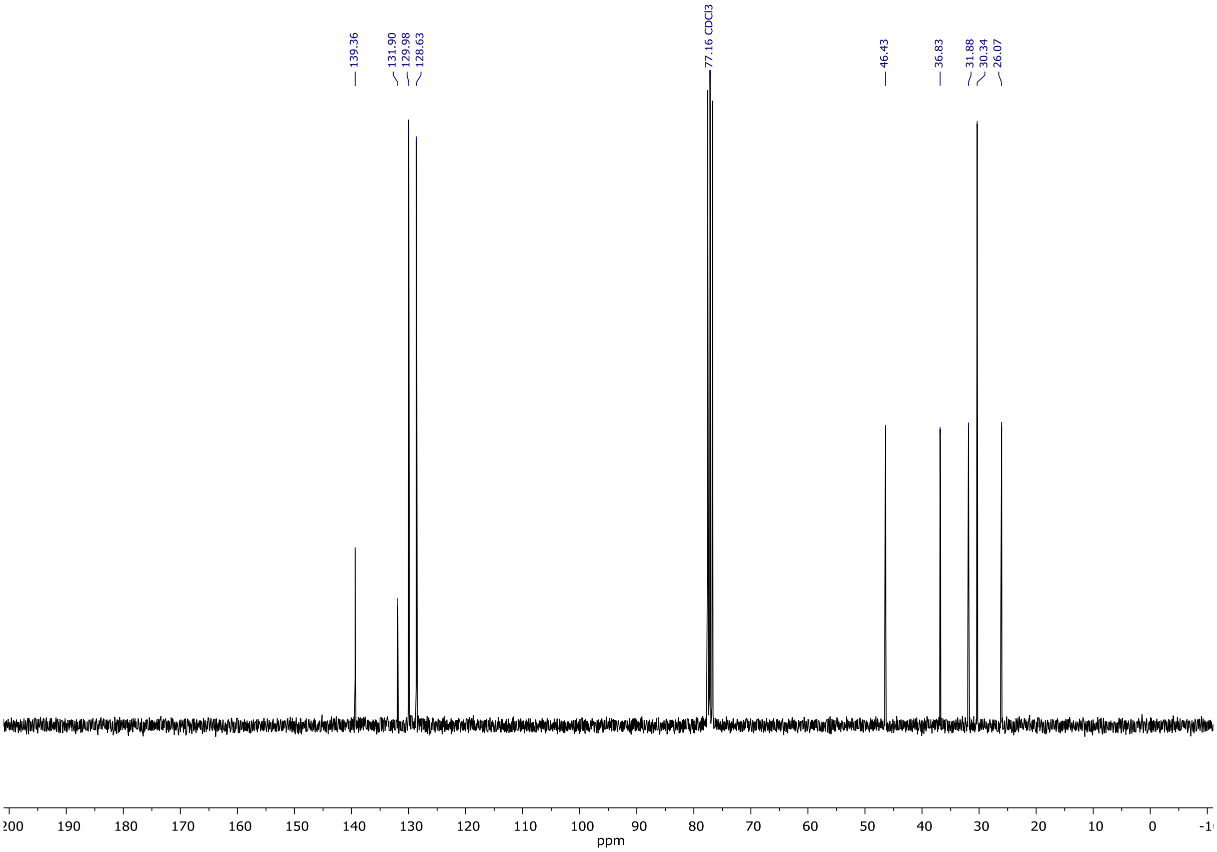

Compound **1i**, ^{1}H- and ^{13}C-NMR ($CDCl_3$):

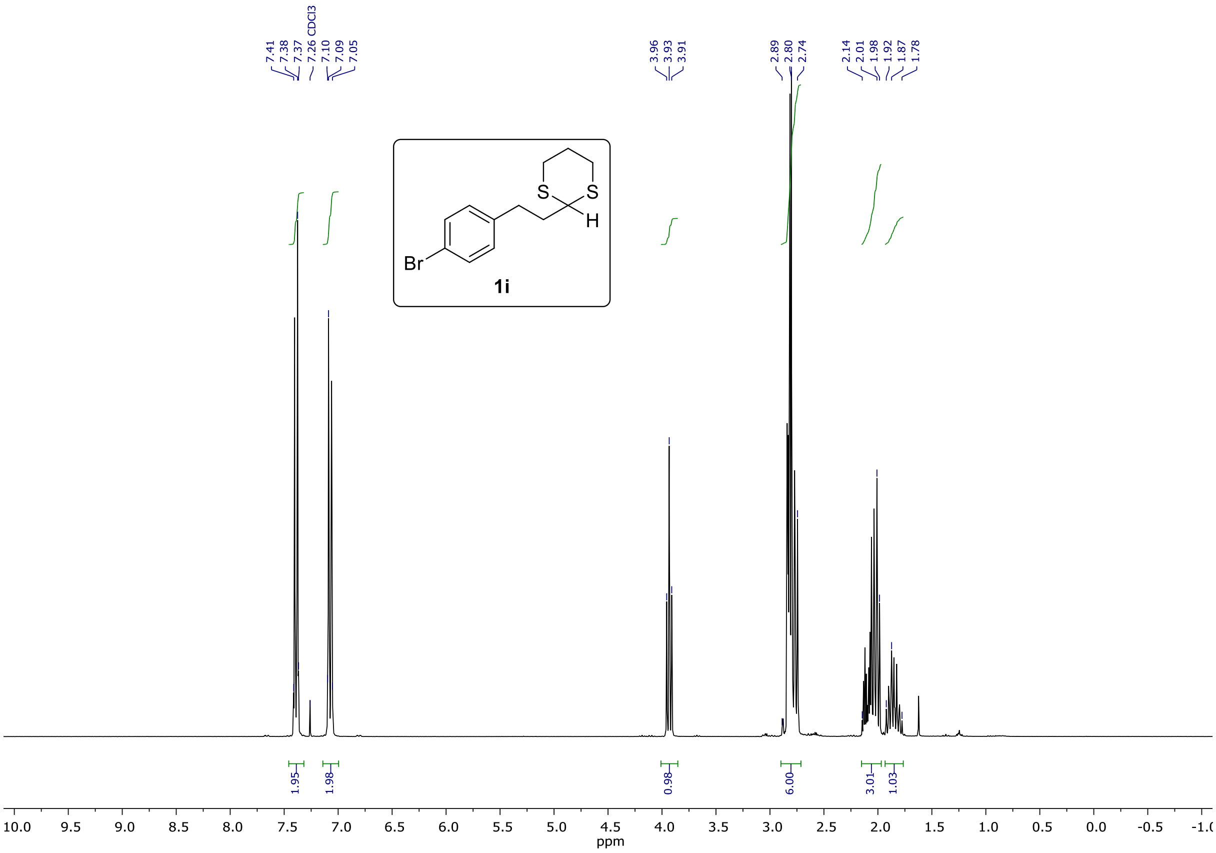

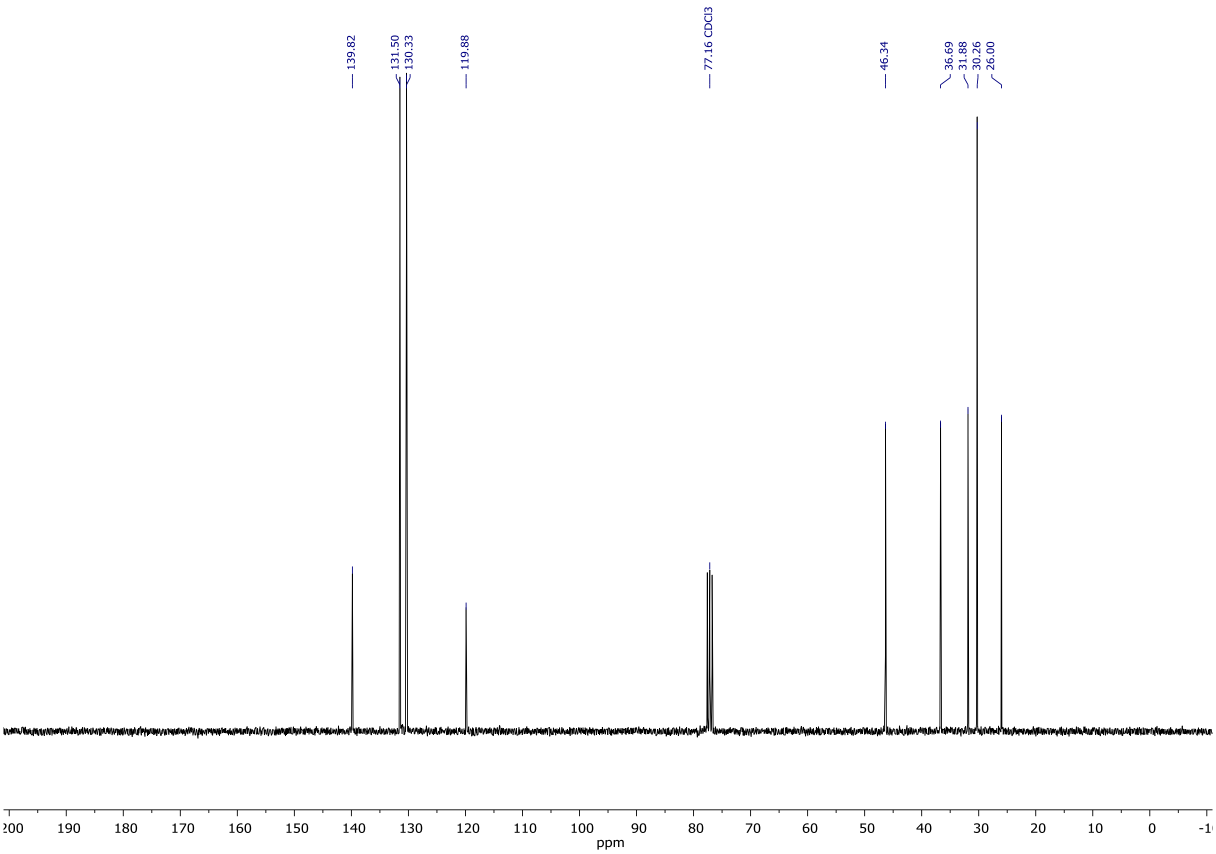

Compound **1j**, ^{1}H- and ^{13}C-NMR ($CDCl_3$) (93 wt% purity):

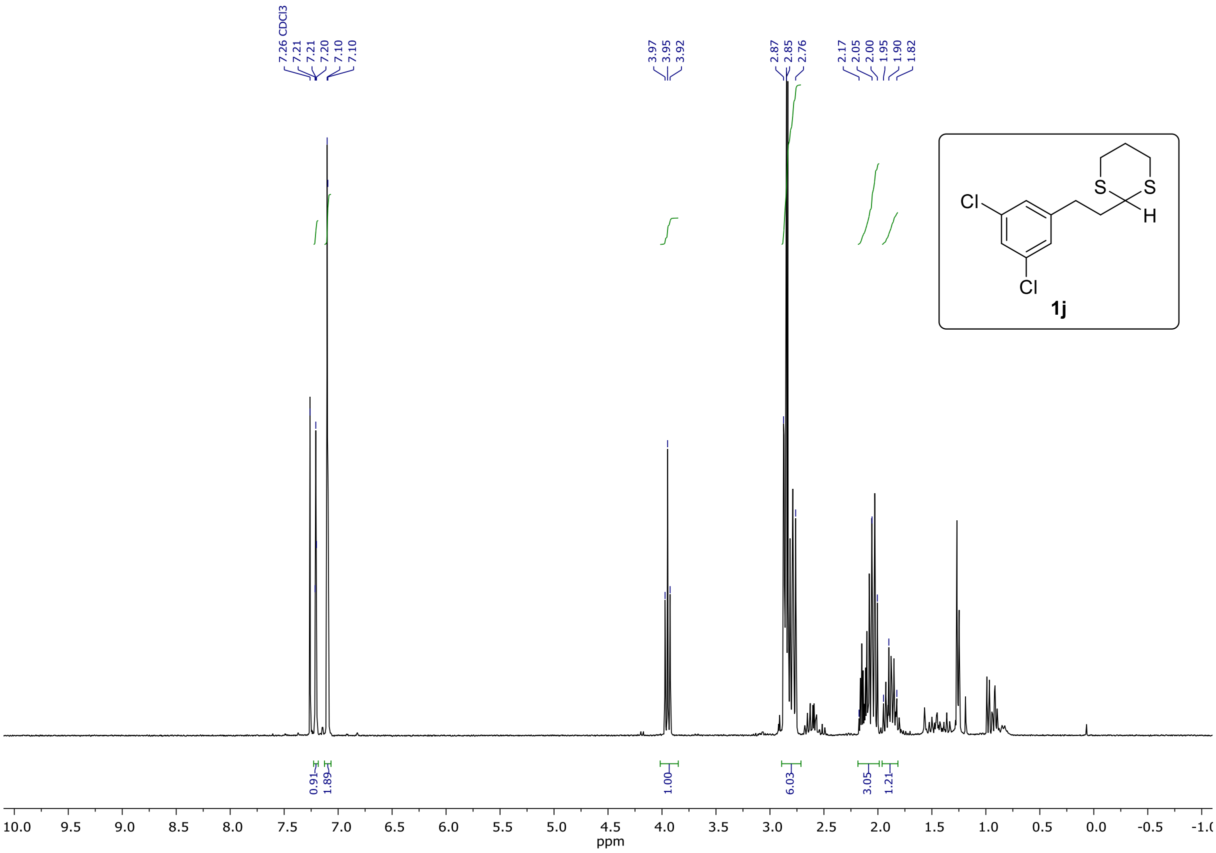

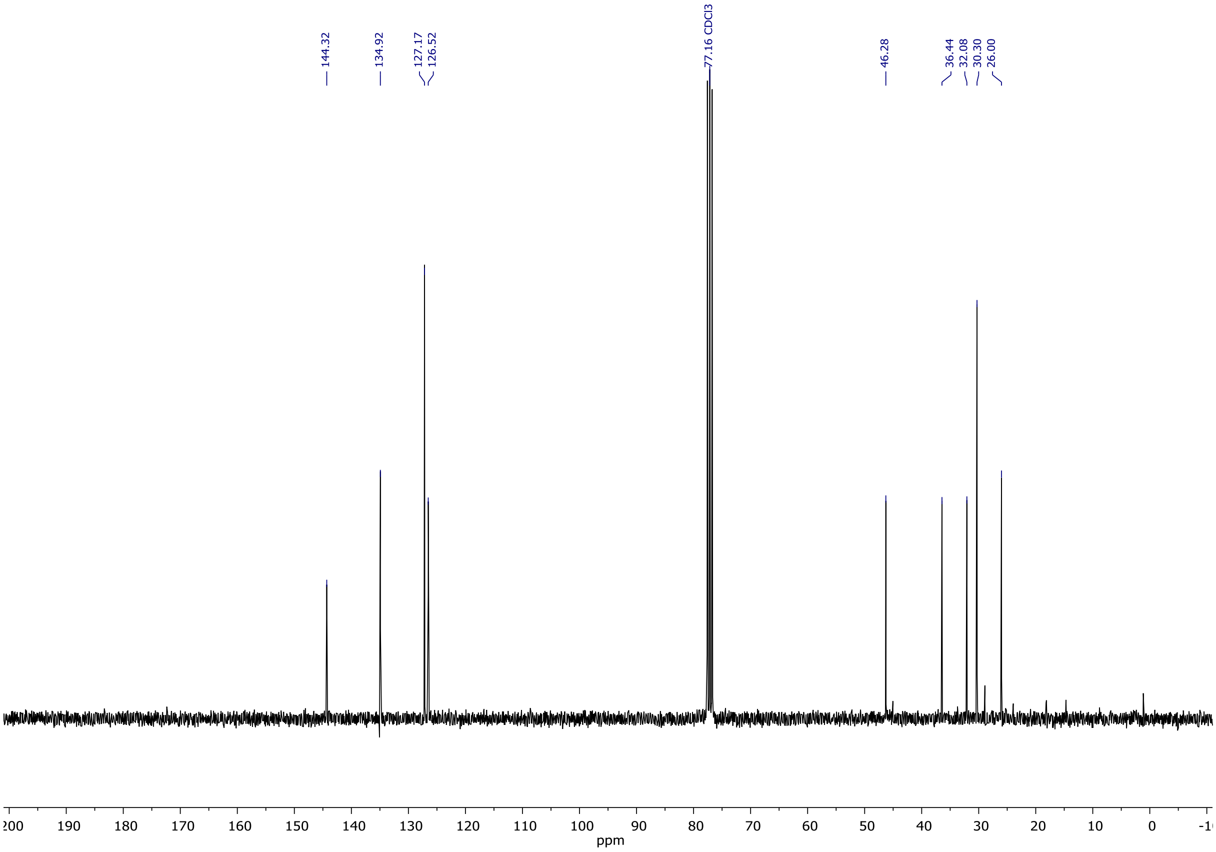

Compound **1k**, ^{1}H- and ^{13}C-NMR ($CDCl_3$):

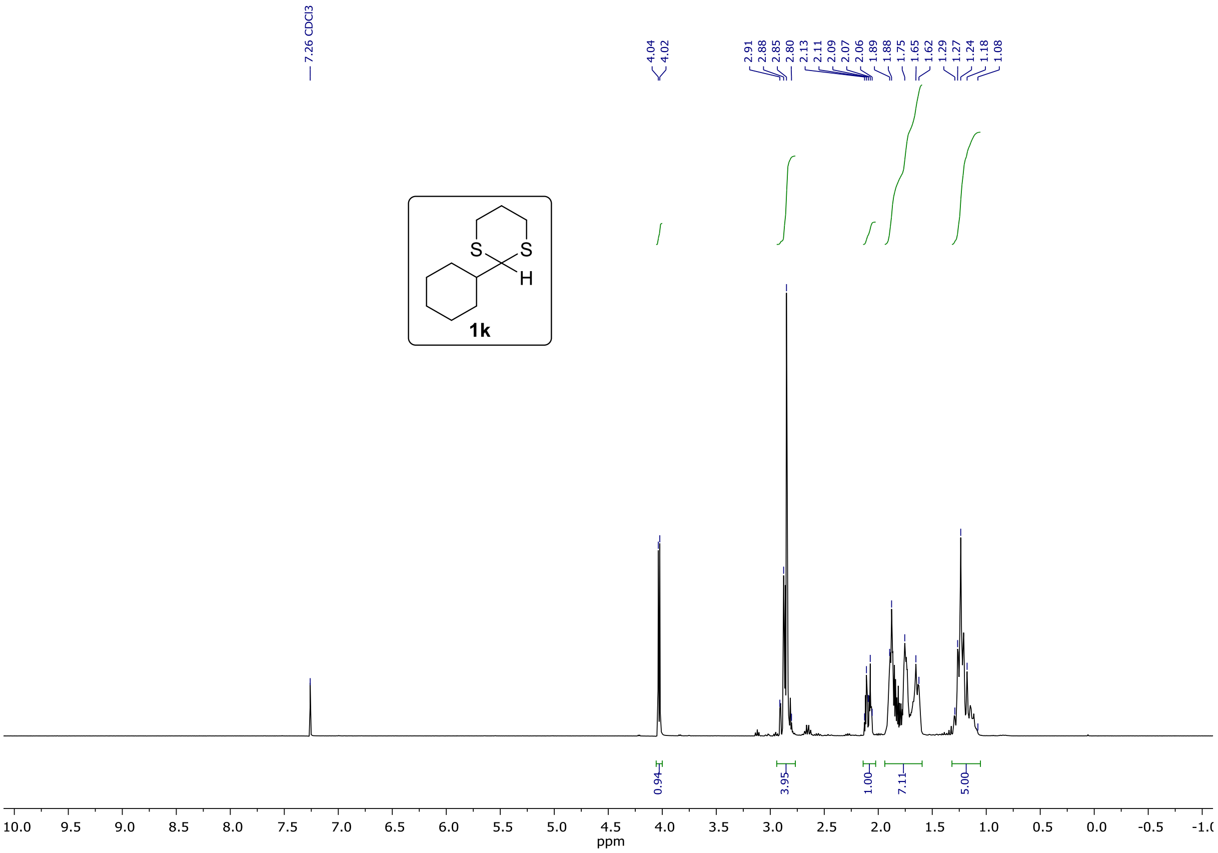

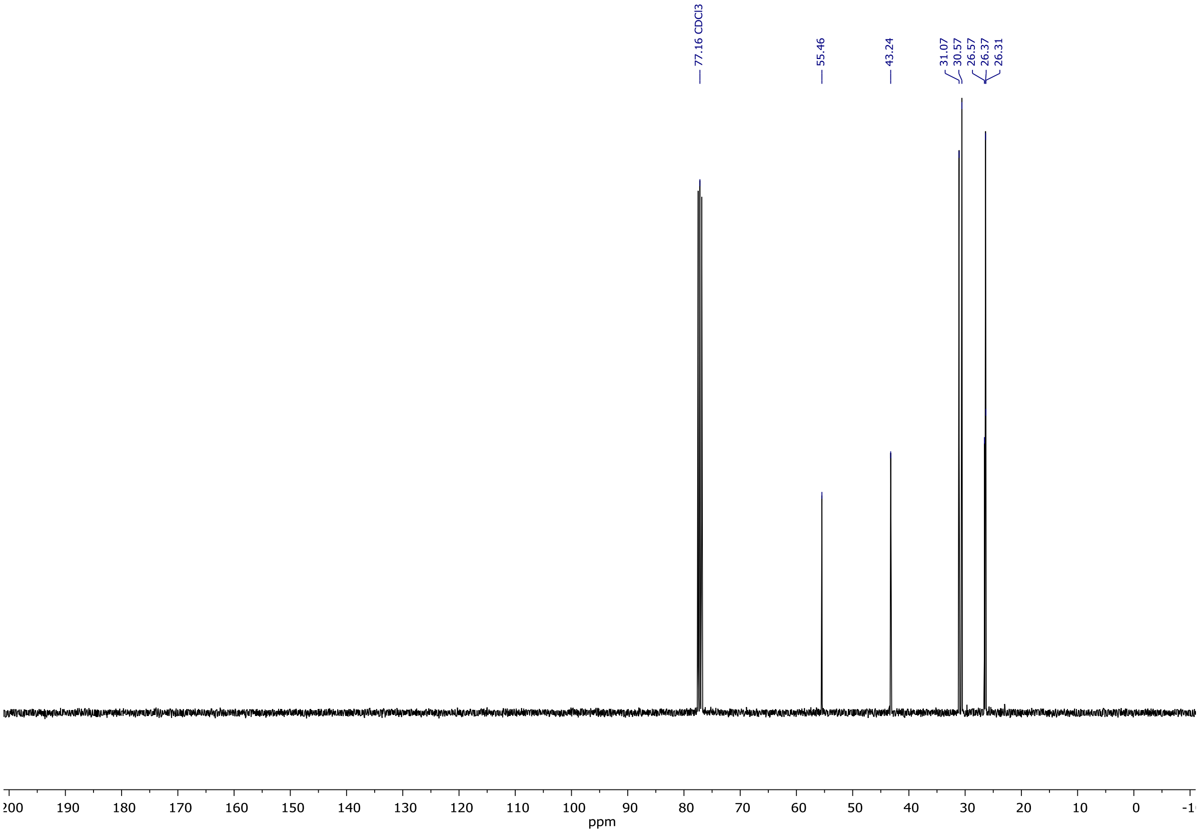

Compound **1m**, ^{1}H- and ^{13}C-NMR ($CDCl_3$):

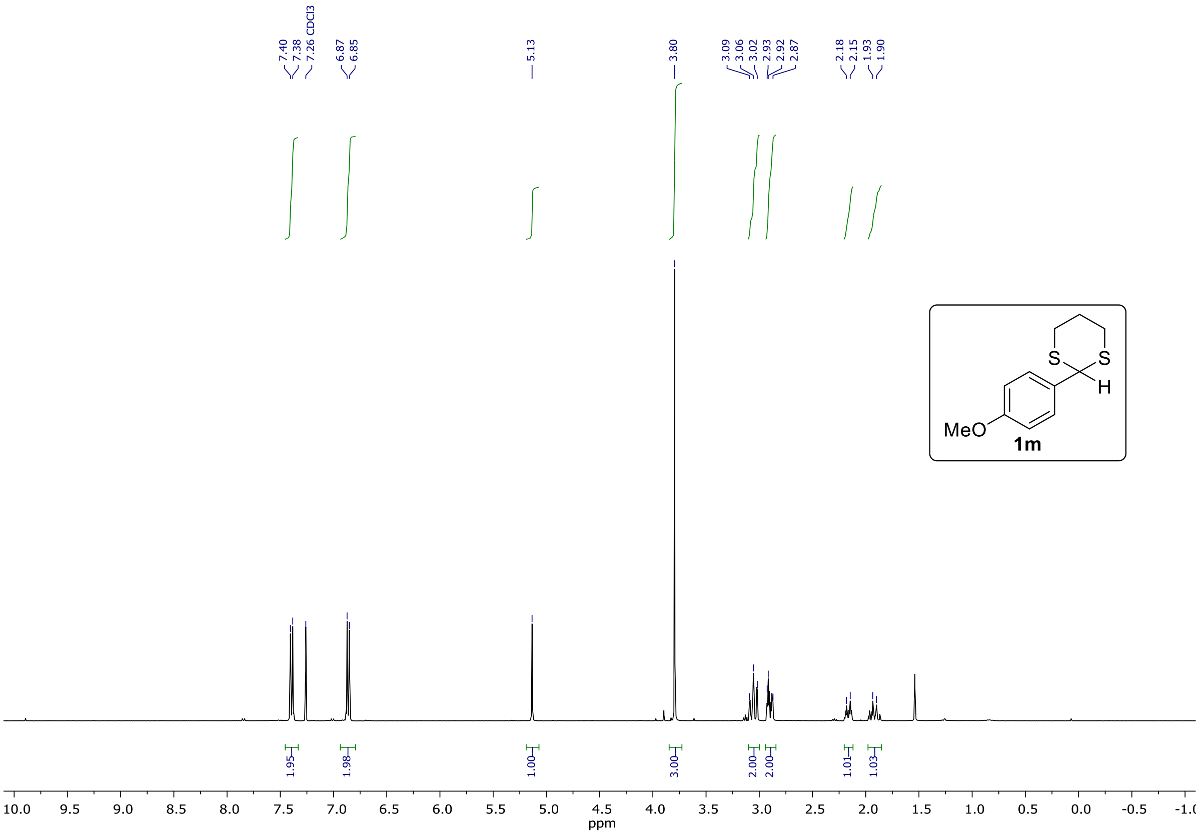

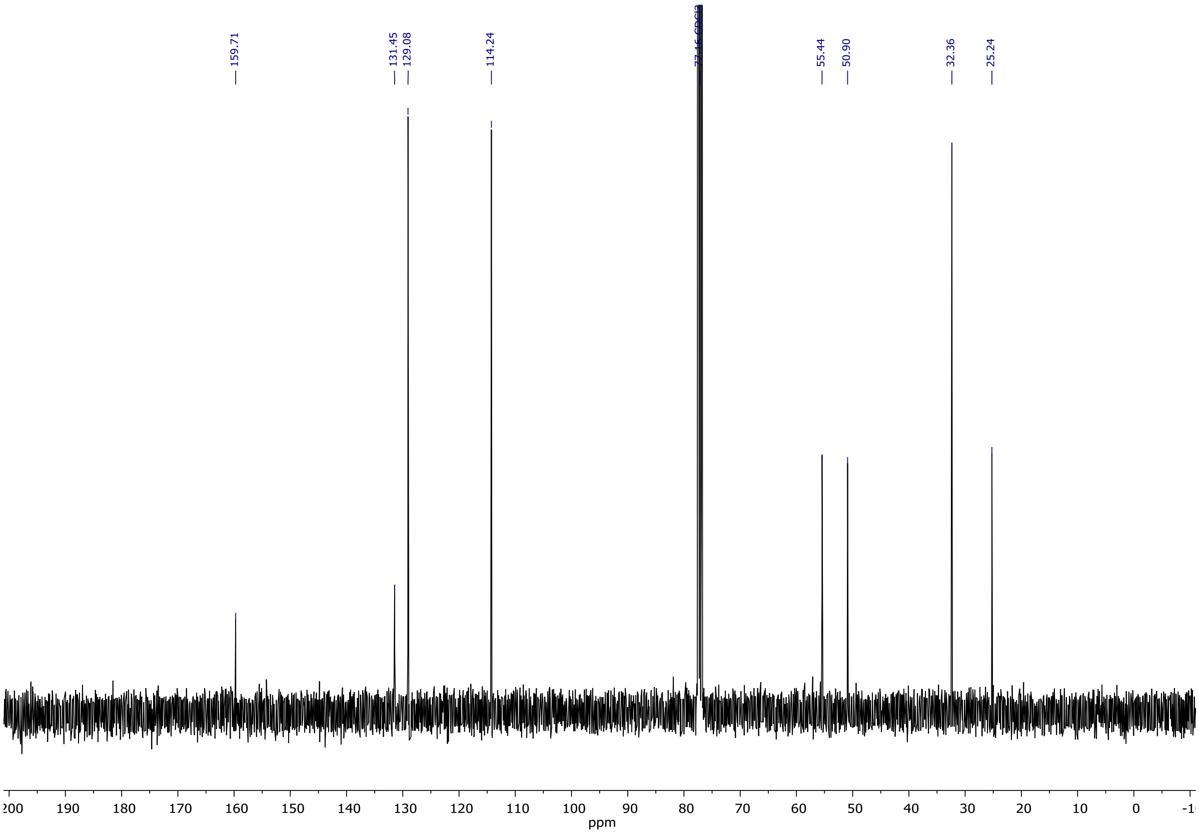

Compound **1n**, ^{1}H- and ^{13}C-NMR ($CDCl_3$):

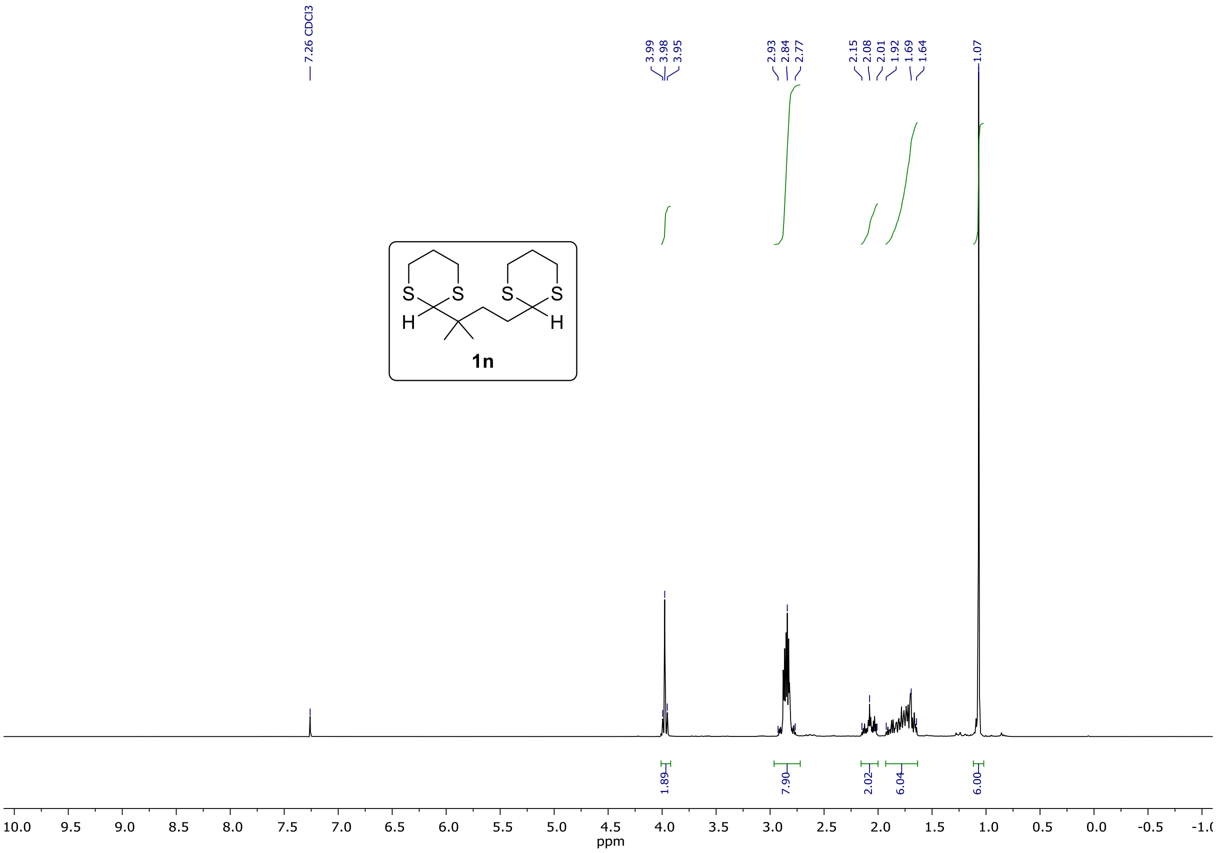

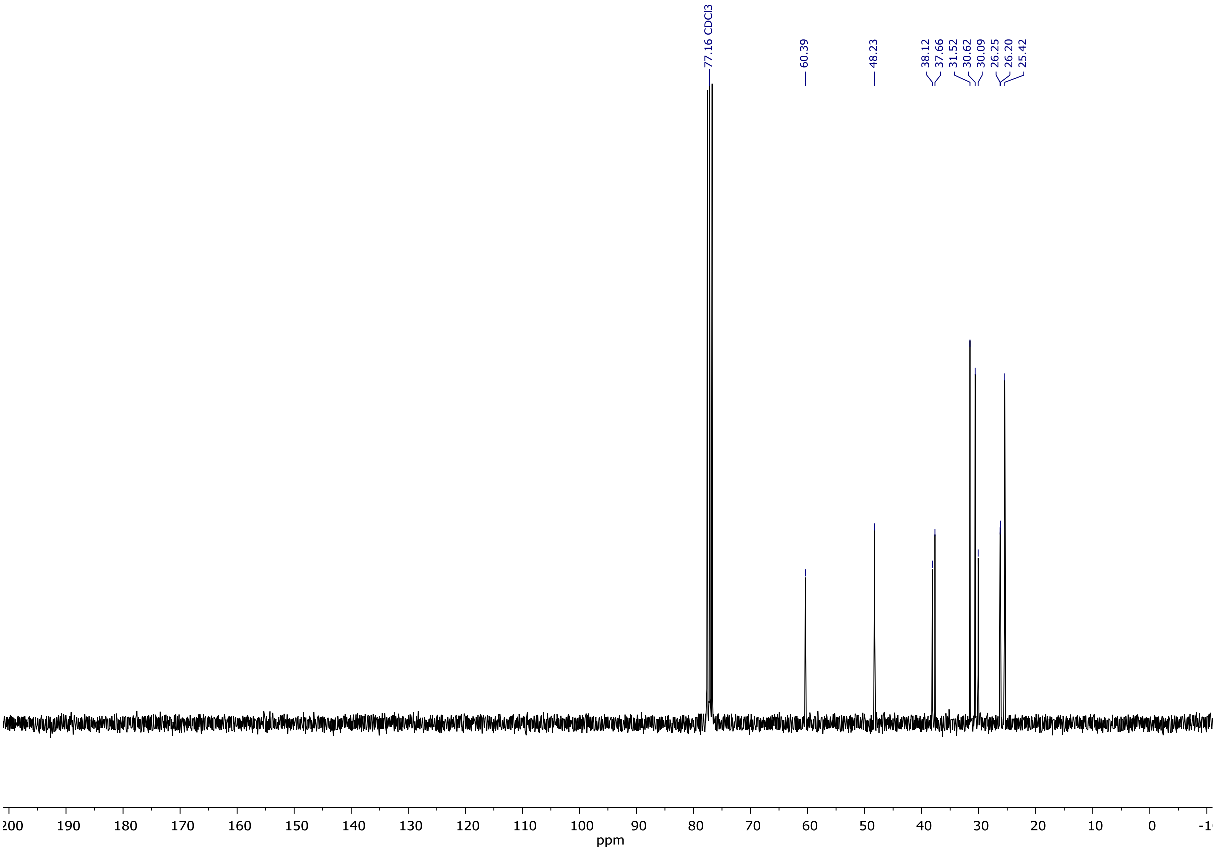

Compound **1o**, ^{1}H- and ^{13}C-NMR ($CDCl_3$):

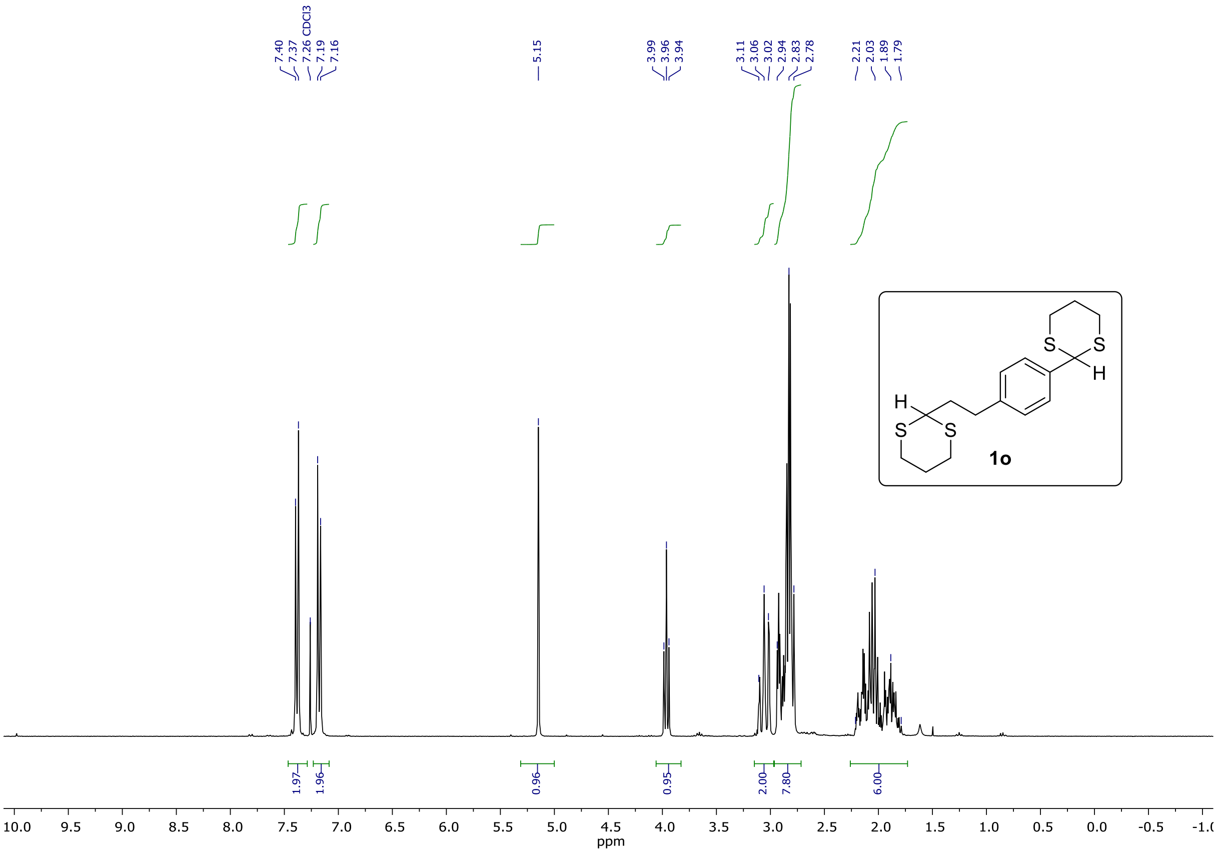

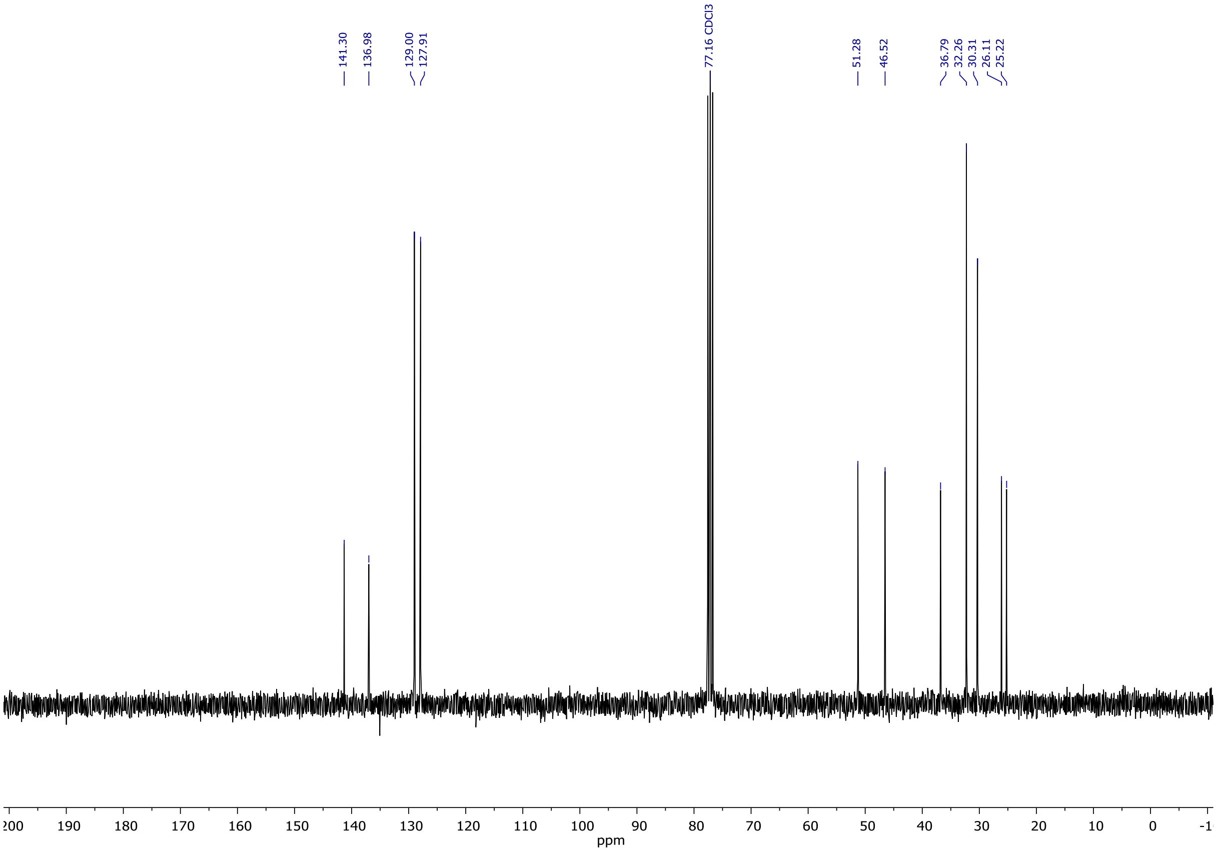

Compound **2i**, ^{1}H- and ^{13}C-NMR ($CDCl_3$):

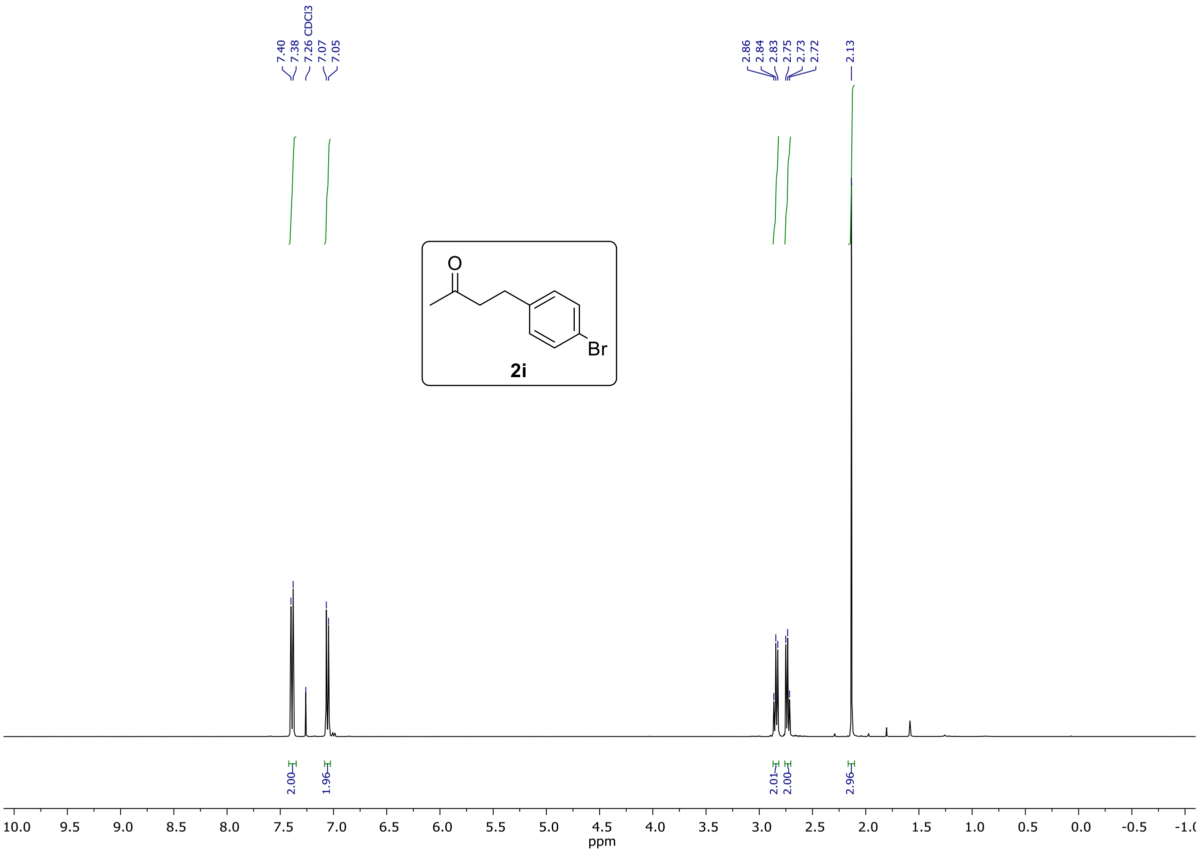

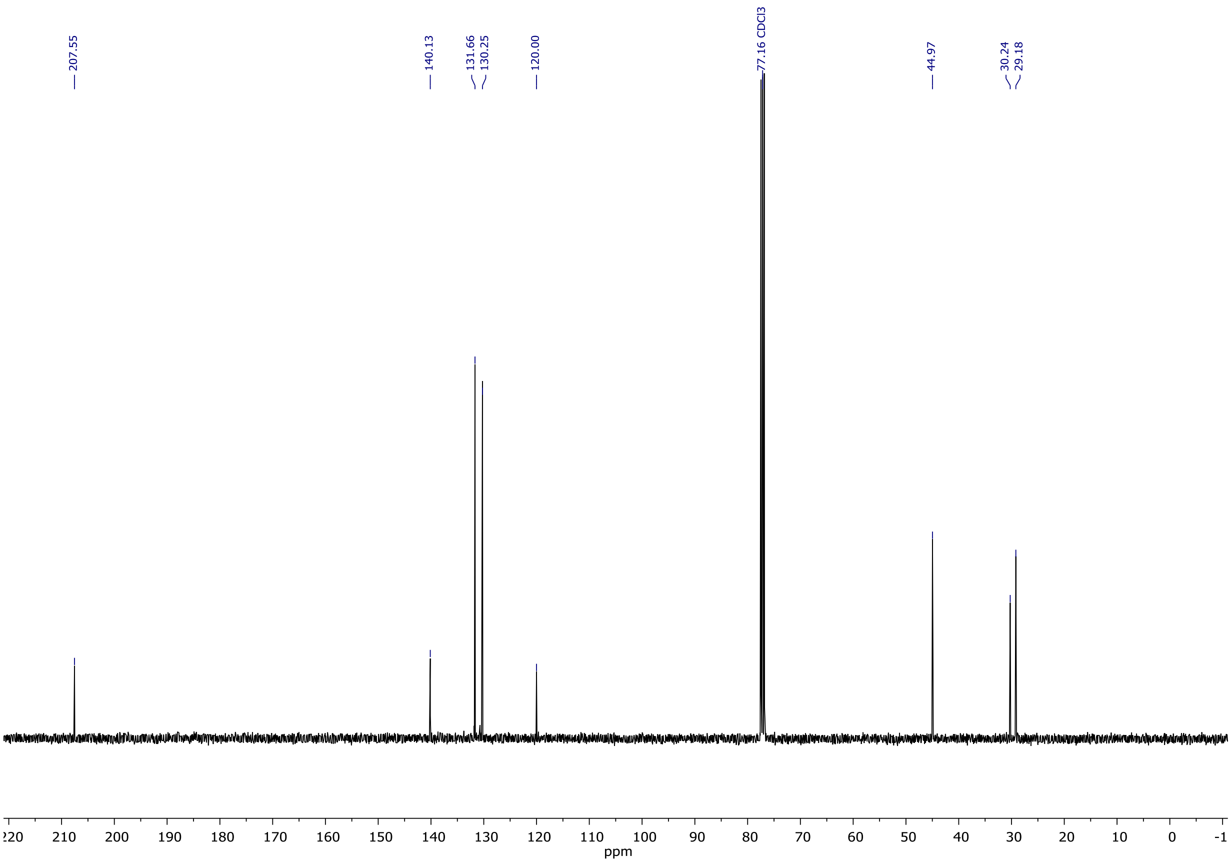

Compound **2j**, ^{1}H- and ^{13}C-NMR ($CDCl_3$):

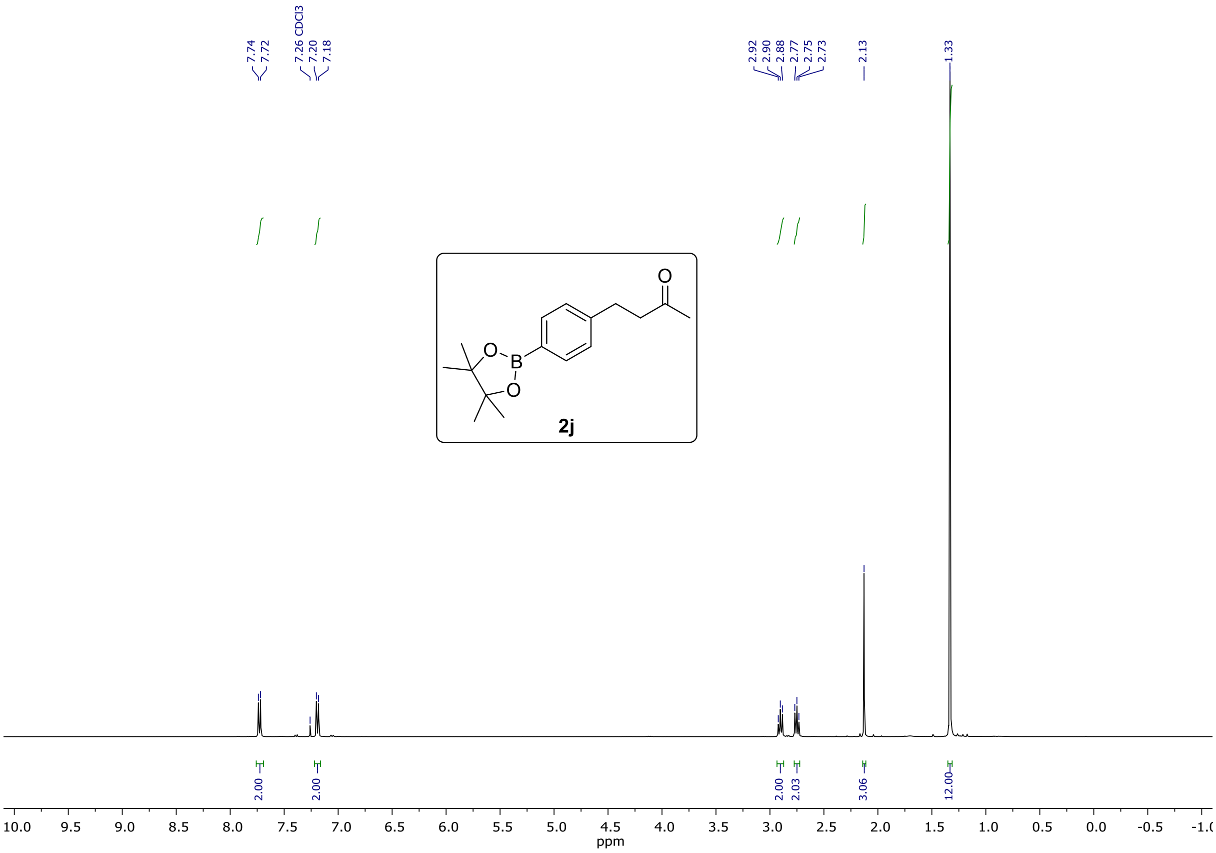

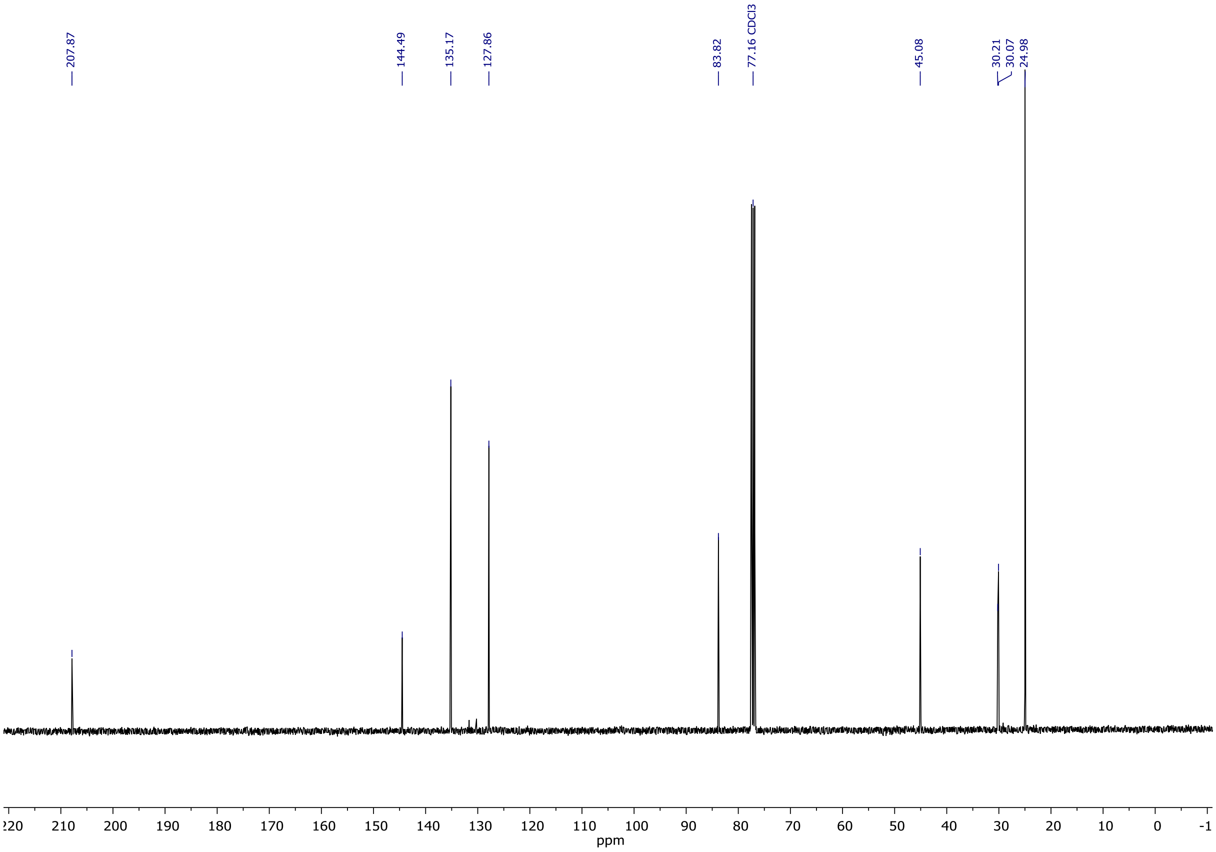

Compound **2k**, ^{1}H- and ^{13}C-NMR ($CDCl_3$):

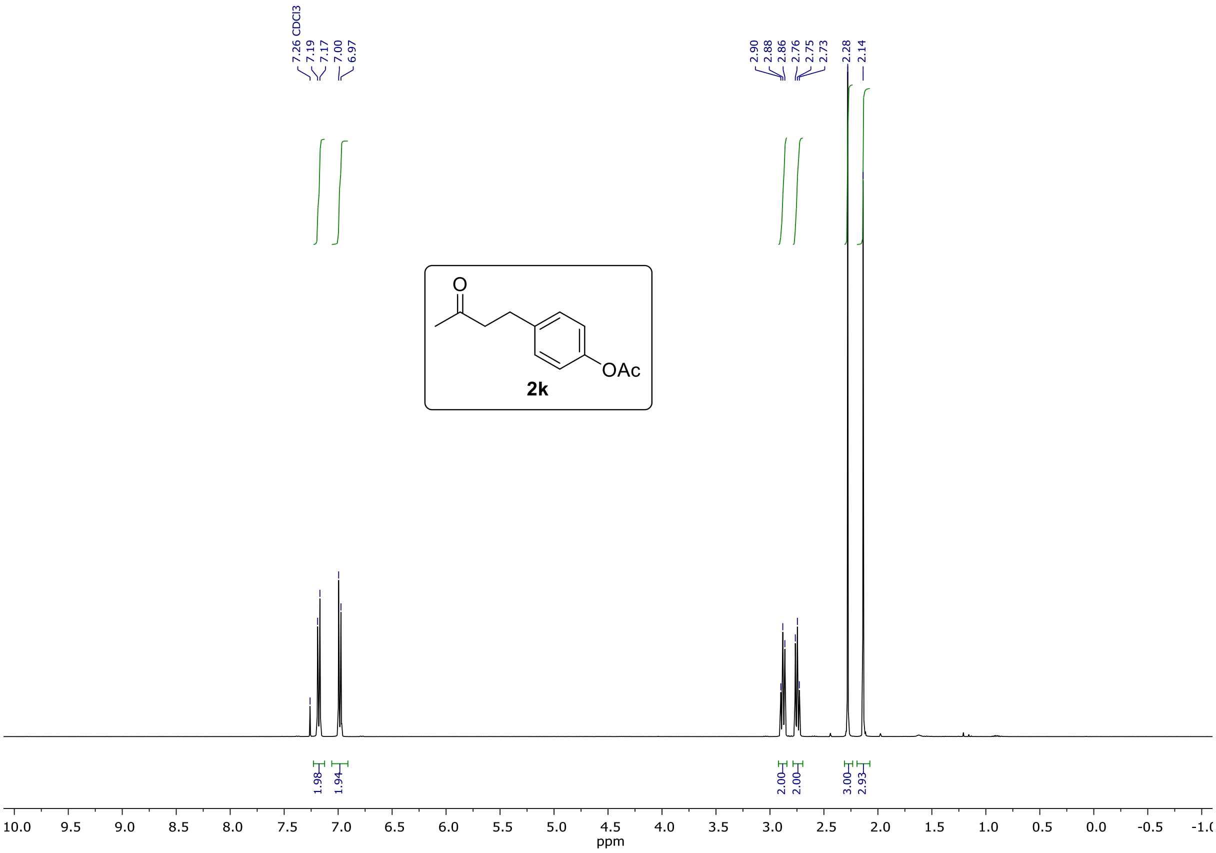

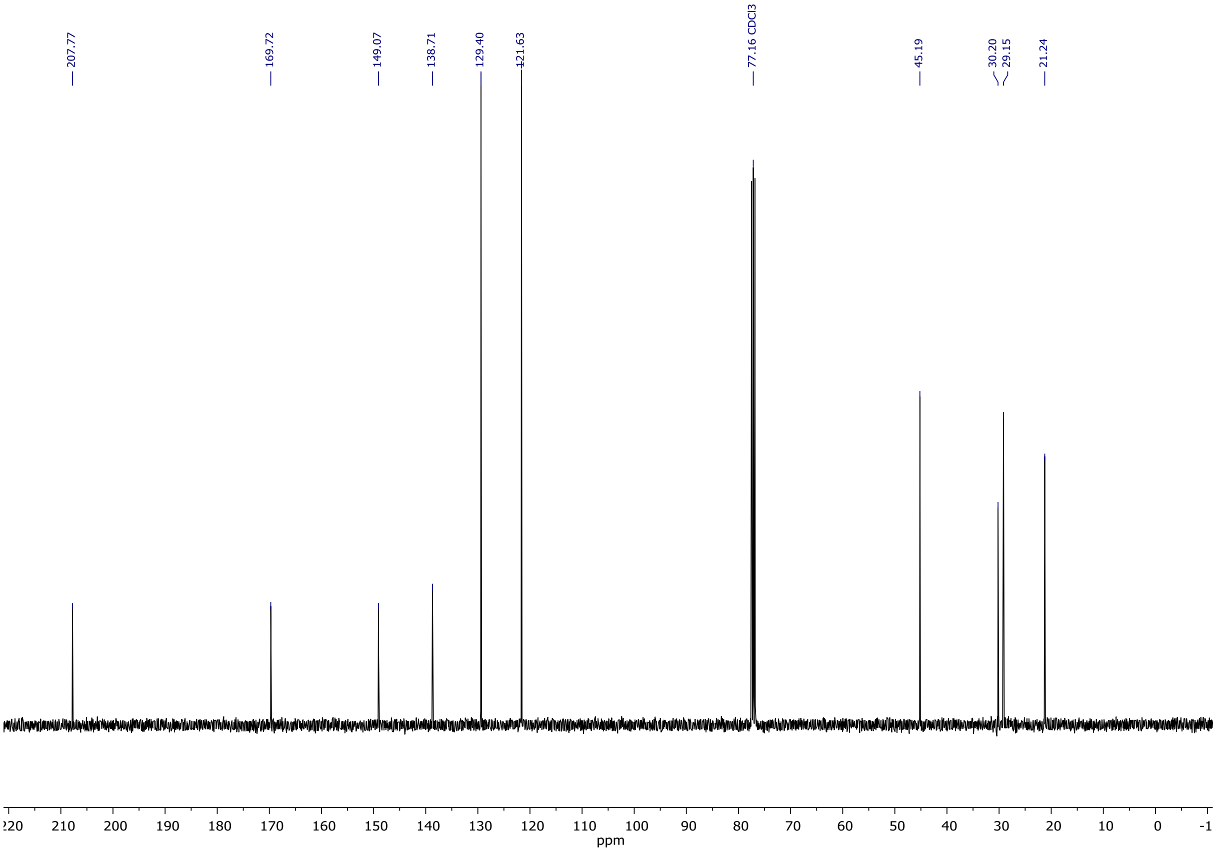

Compound **3aa**, ^{1}H- and ^{13}C-NMR ($CDCl_3$):

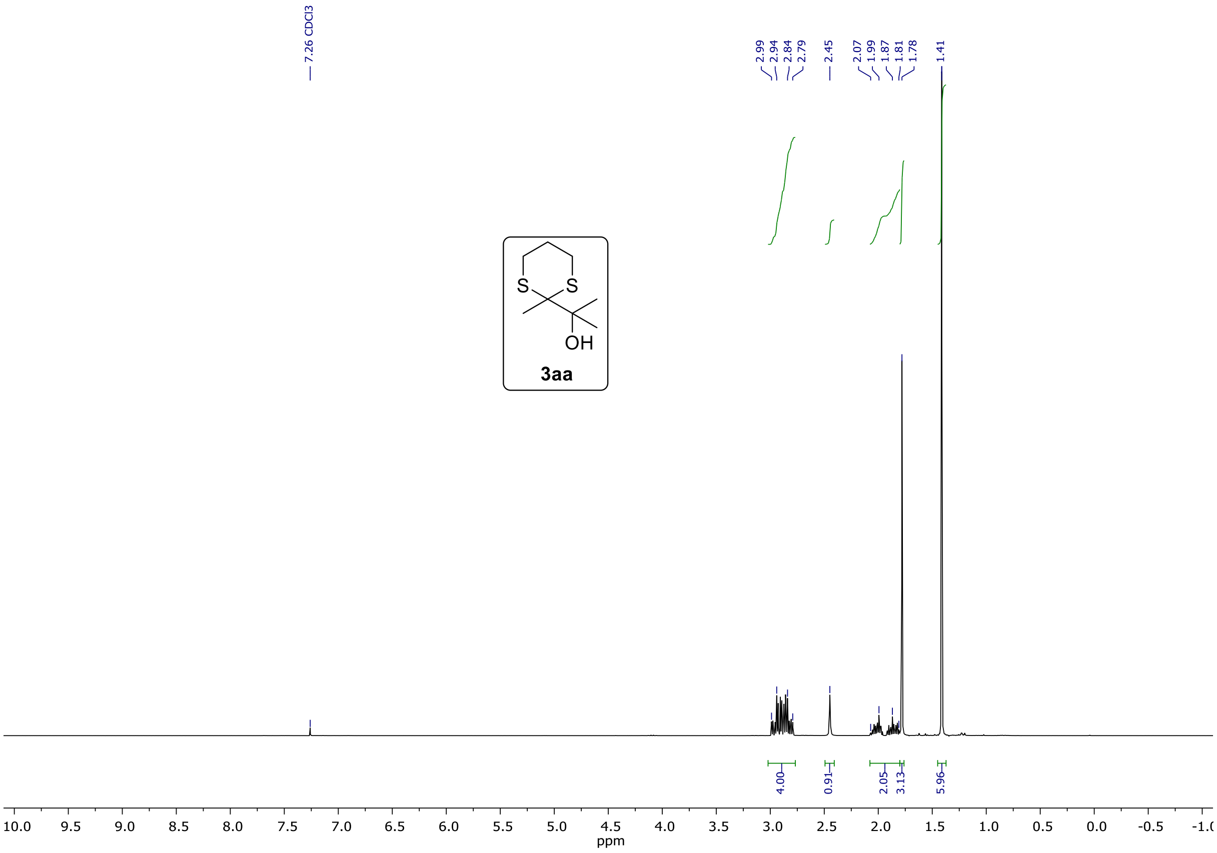

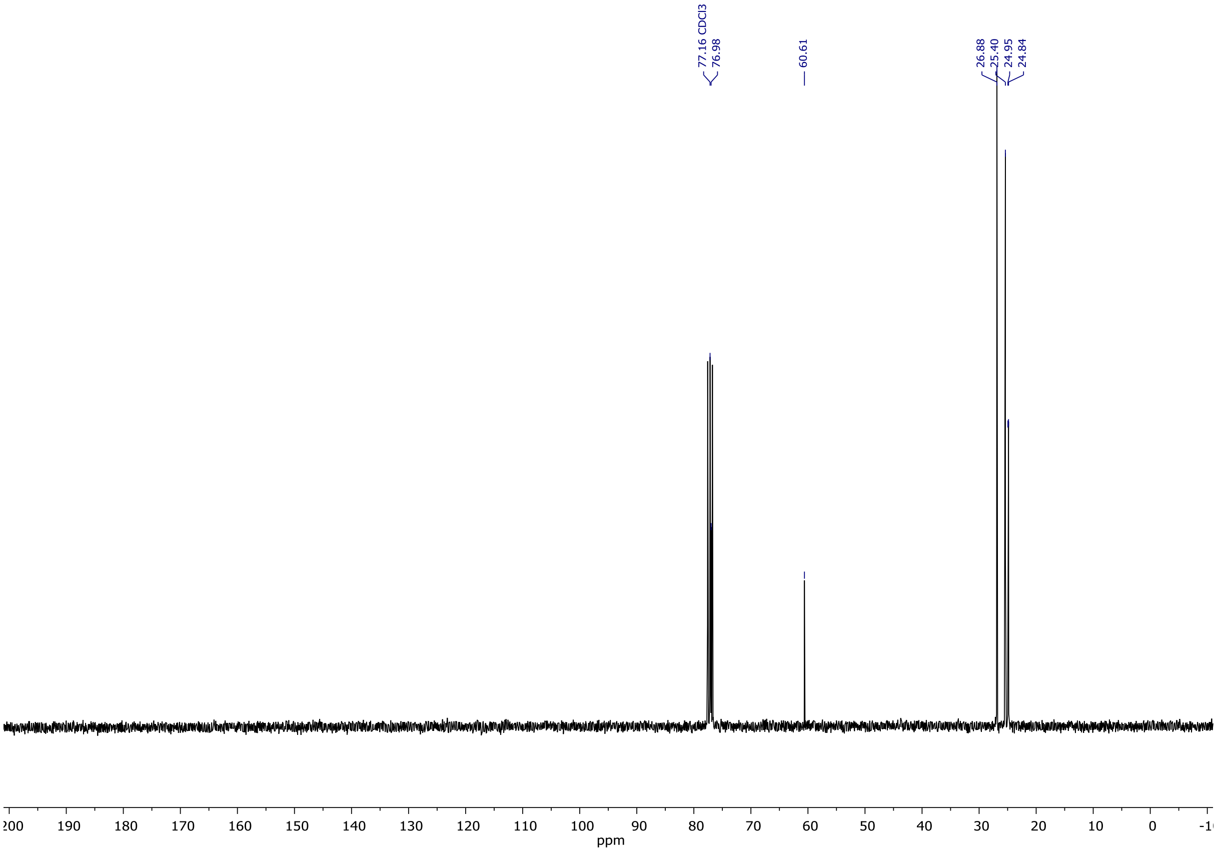

Compound **3ba**, ^{1}H- and ^{13}C-NMR ($CDCl_3$):

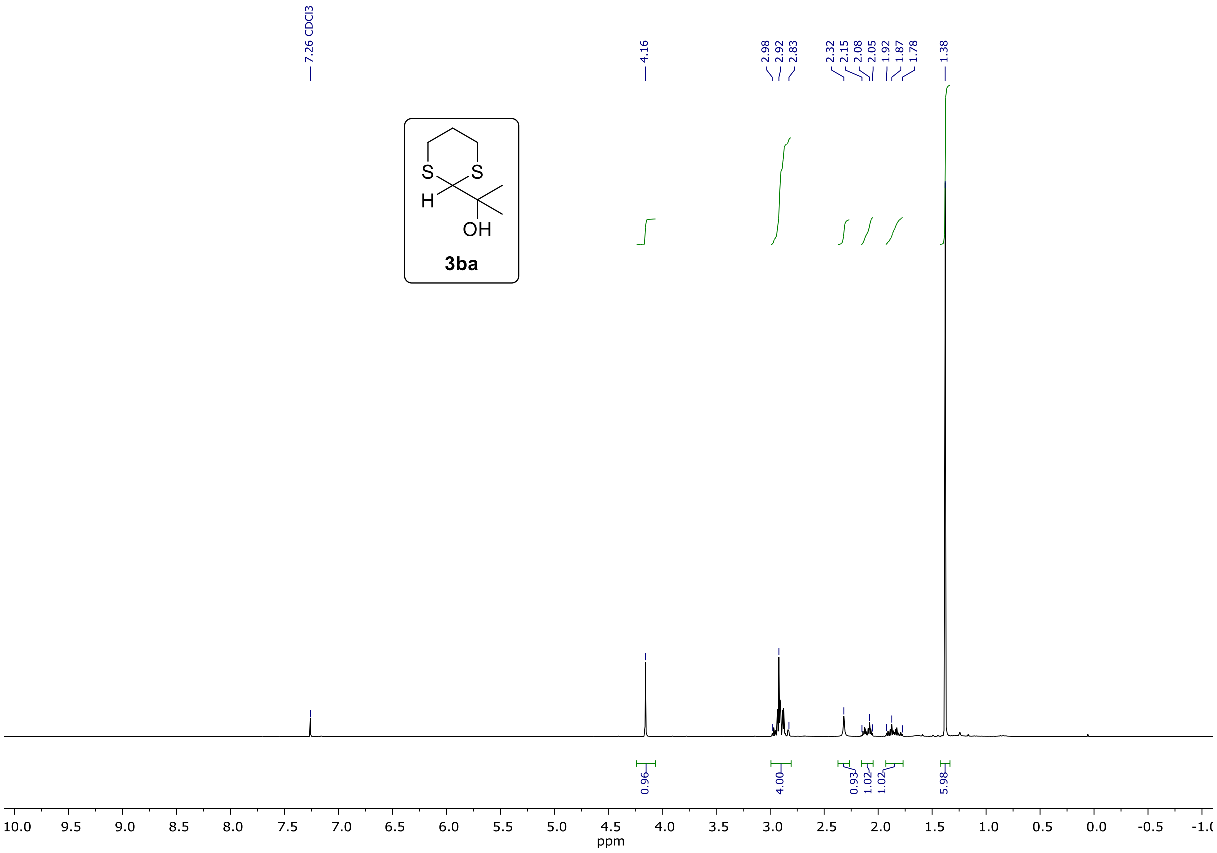

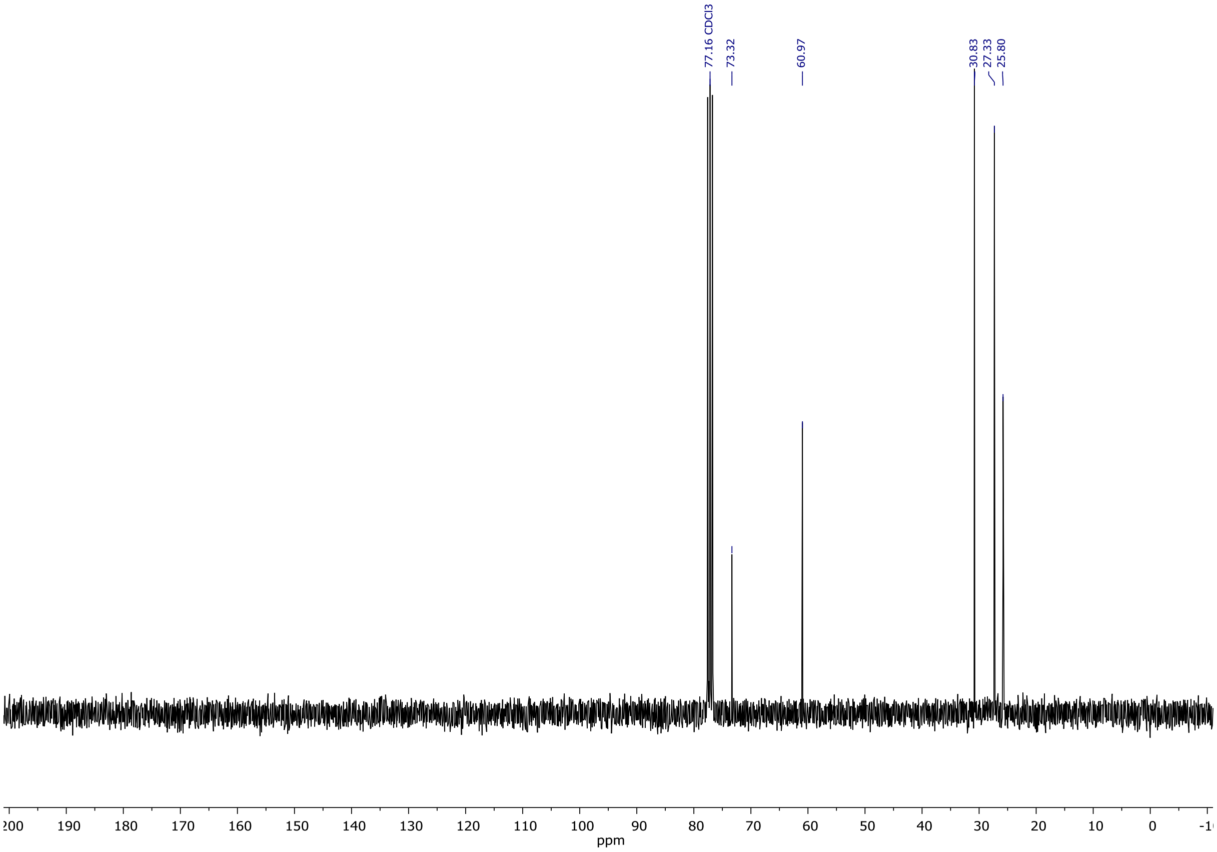

Compound **3ca**, ^{1}H- and ^{13}C-NMR ($CDCl_3$):

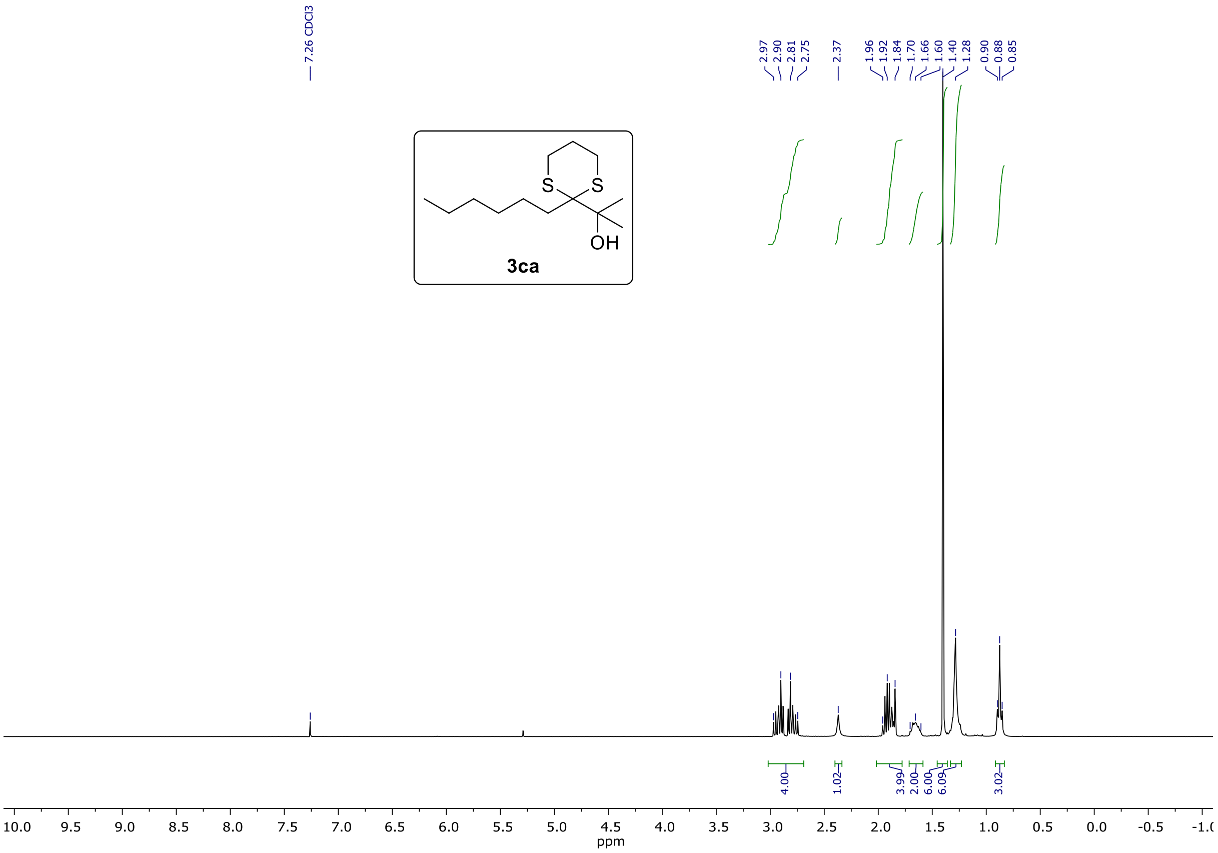

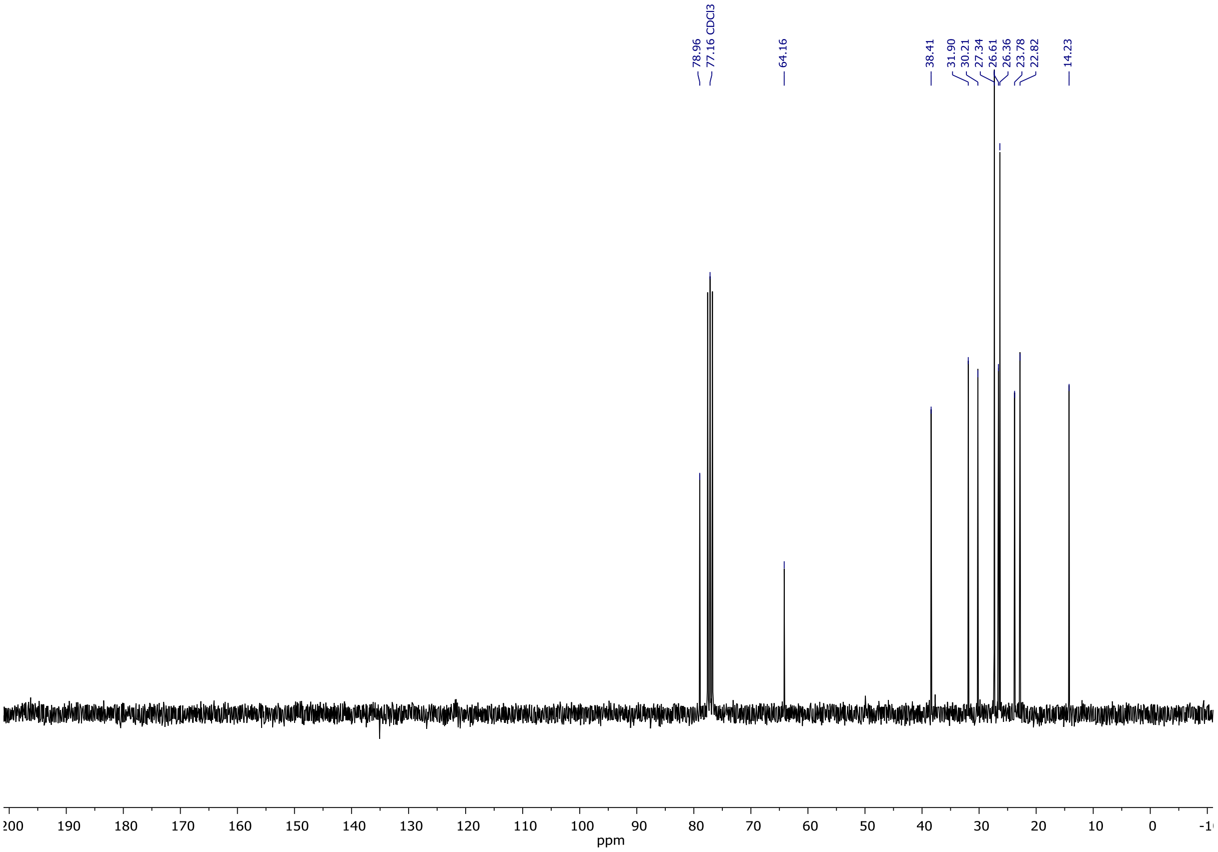

Compound **3da**, ^{1}H- and ^{13}C-NMR ($CDCl_3$):

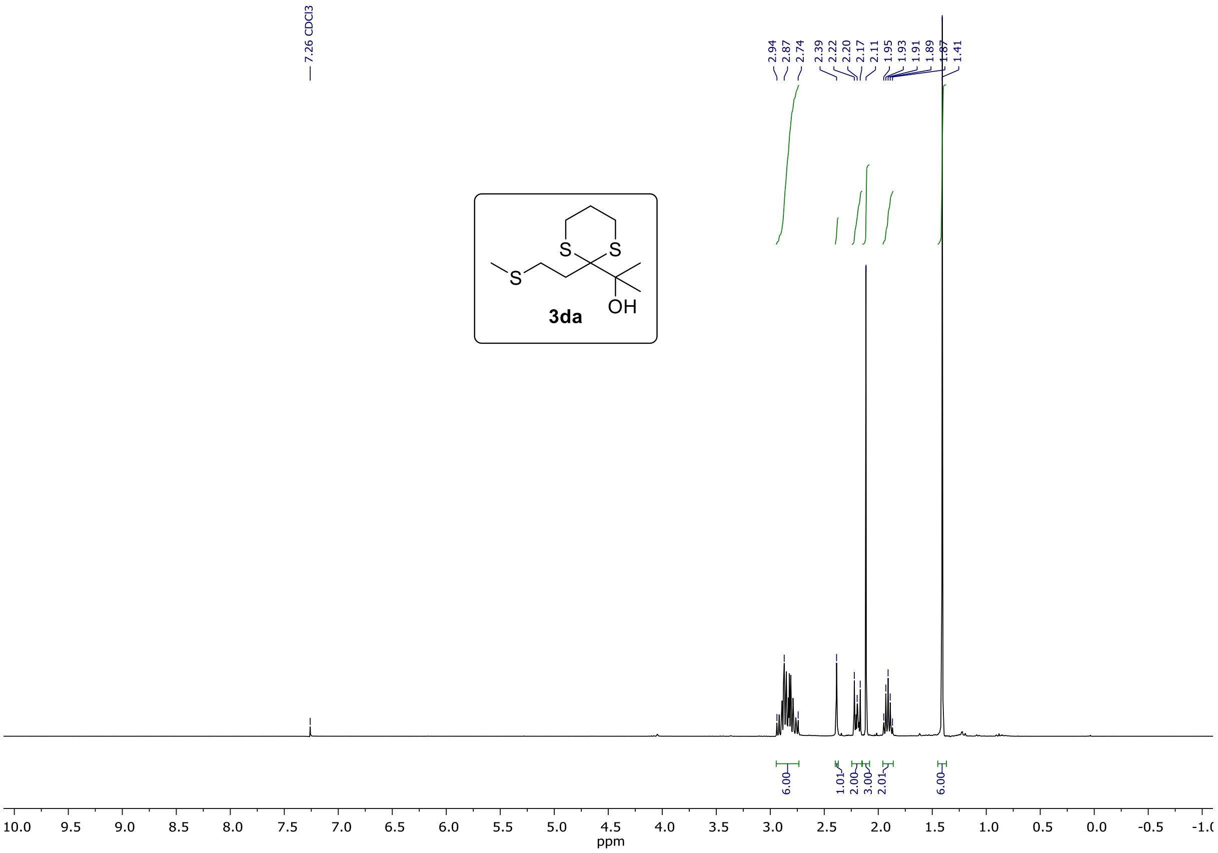

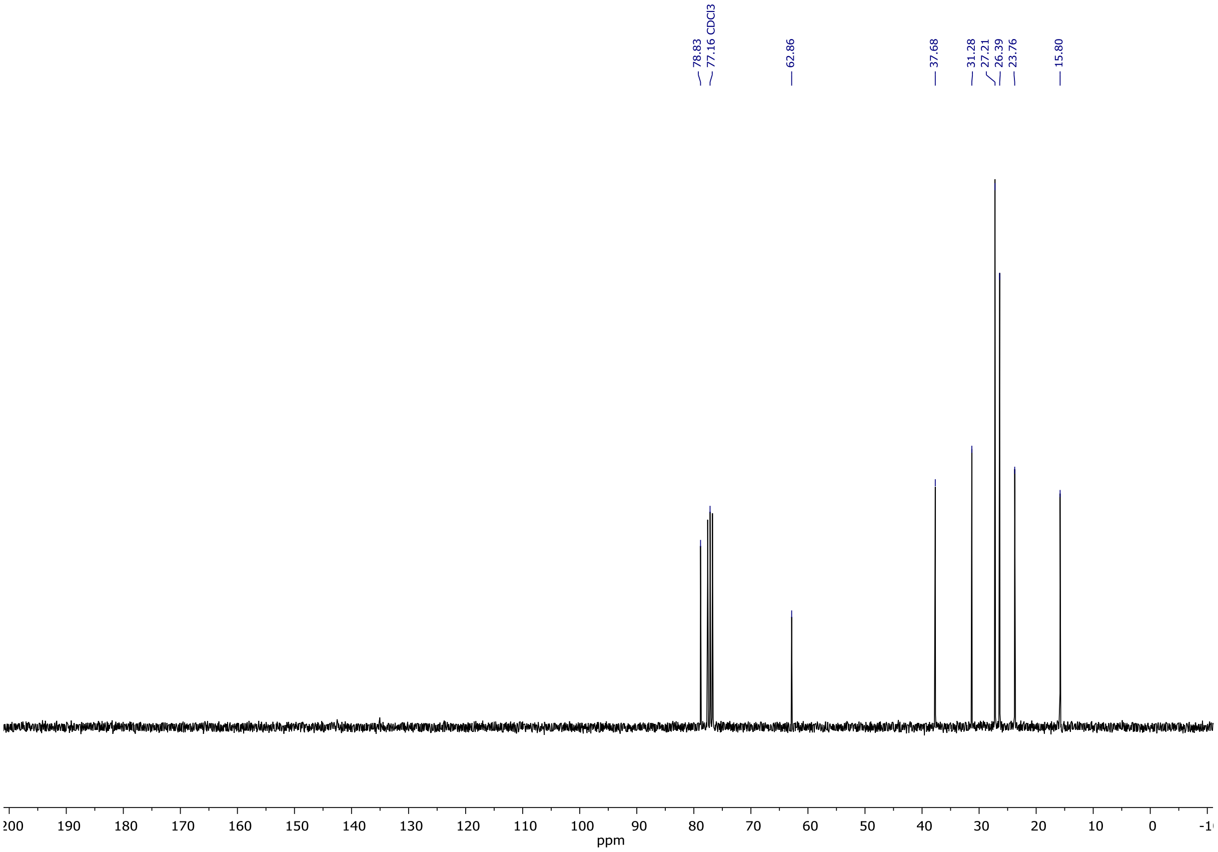

Compound **3ea**, ^{1}H- and ^{13}C-NMR ($CDCl_3$):

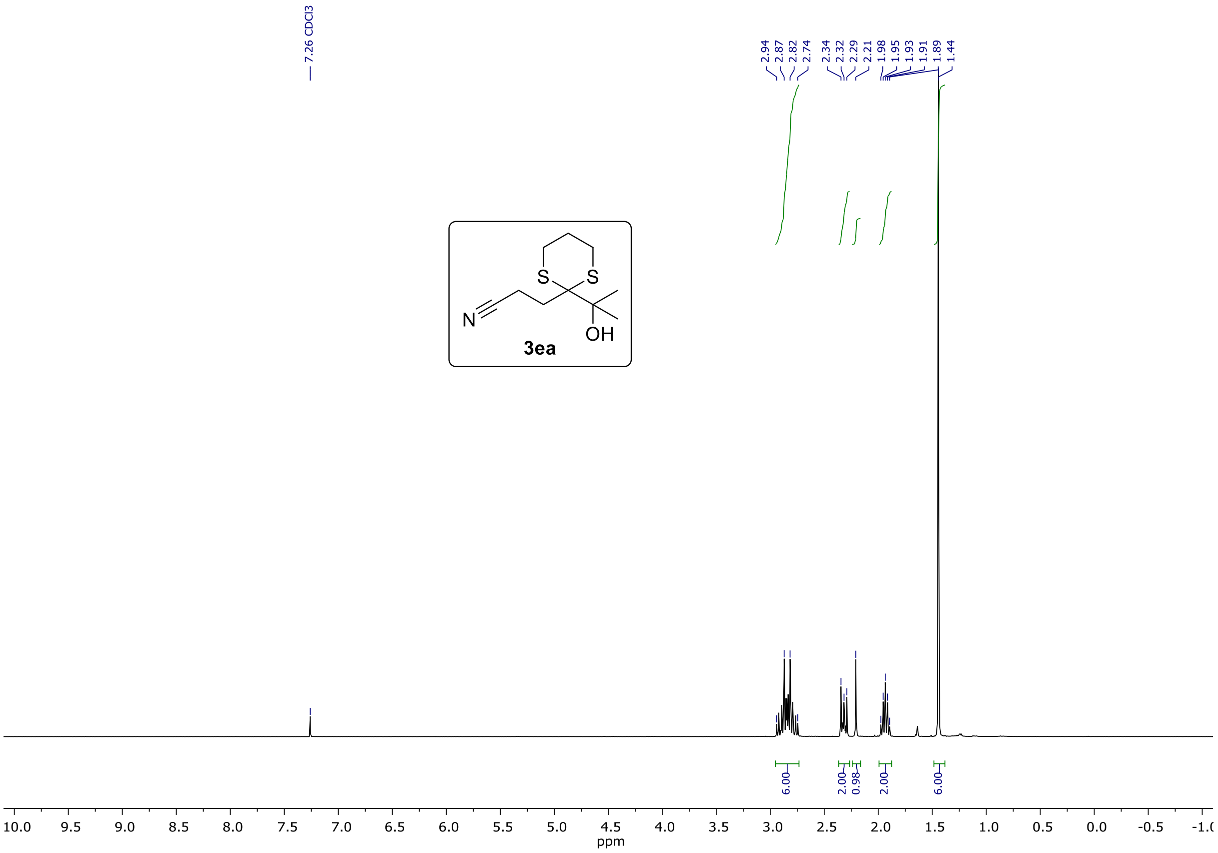

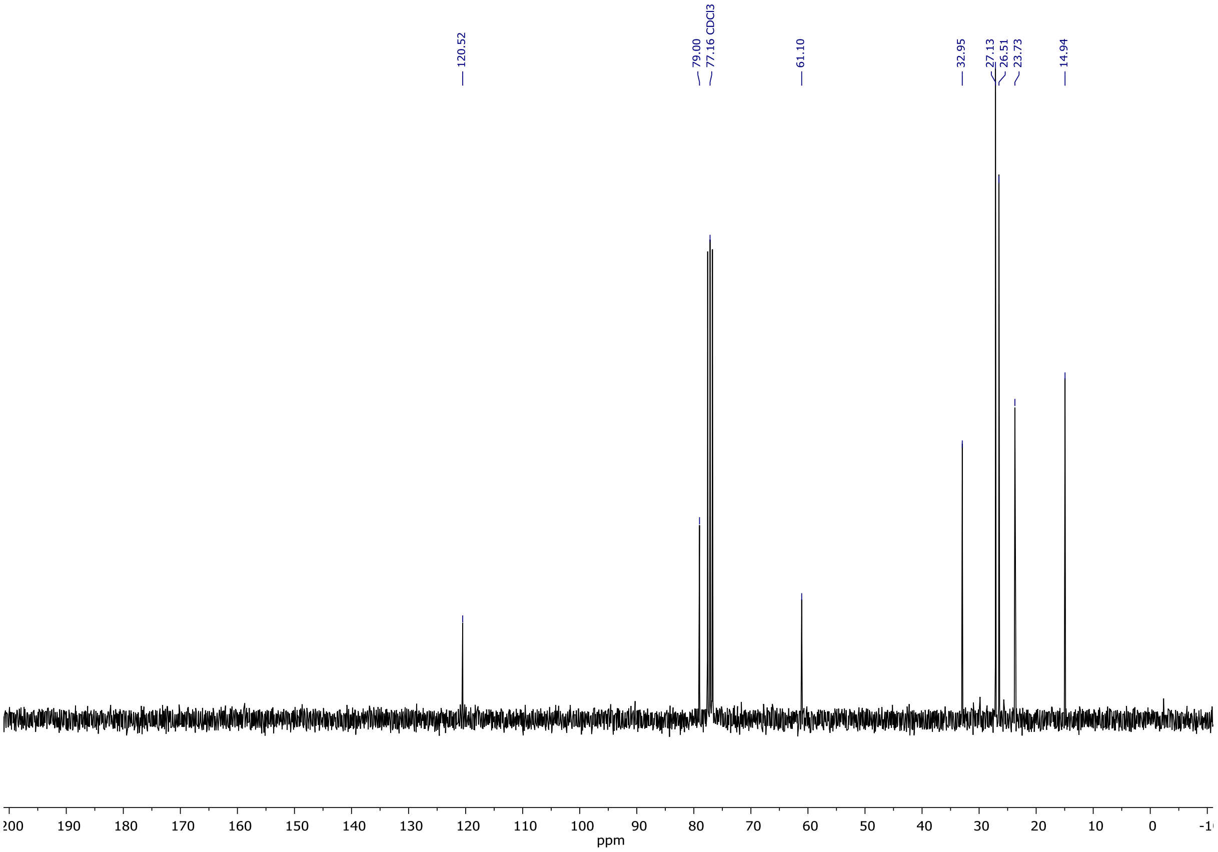

Compound **3fa**, ^{1}H- and ^{13}C-NMR ($CDCl_3$):

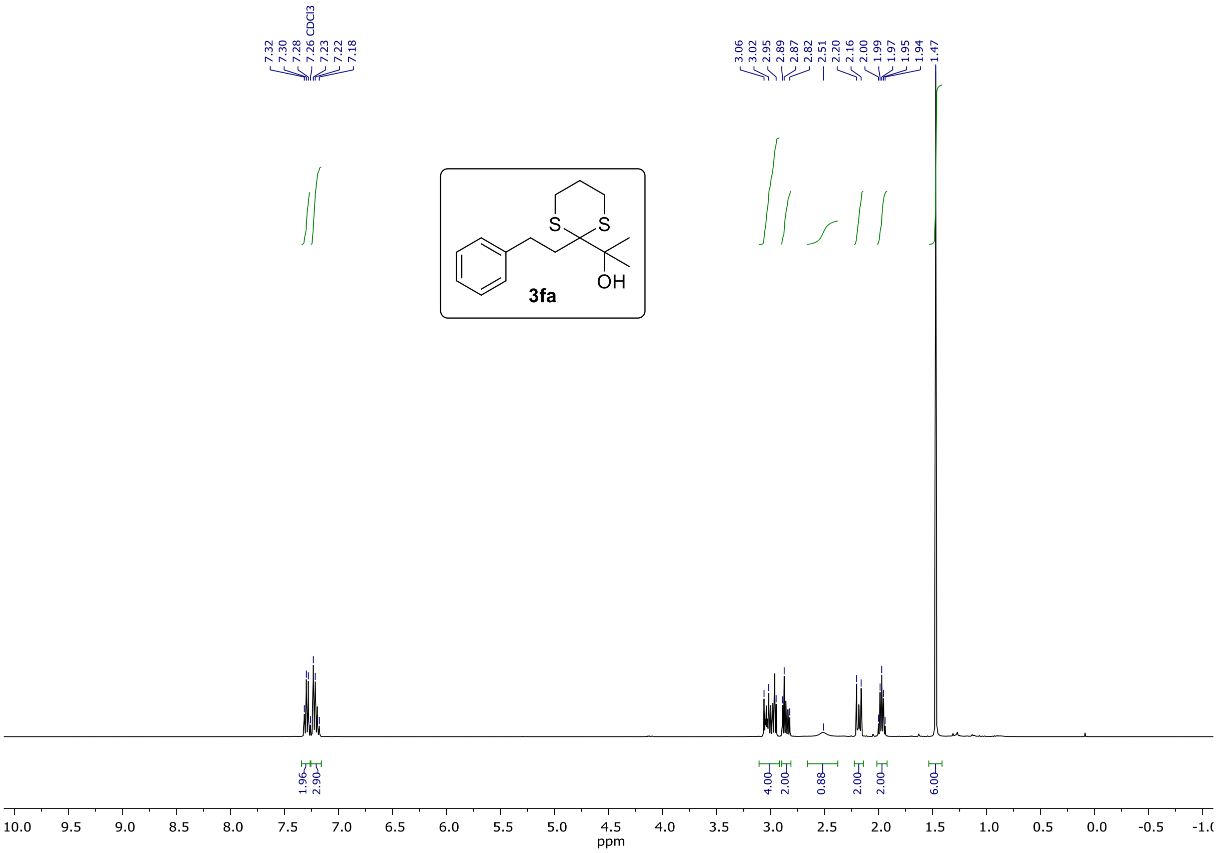

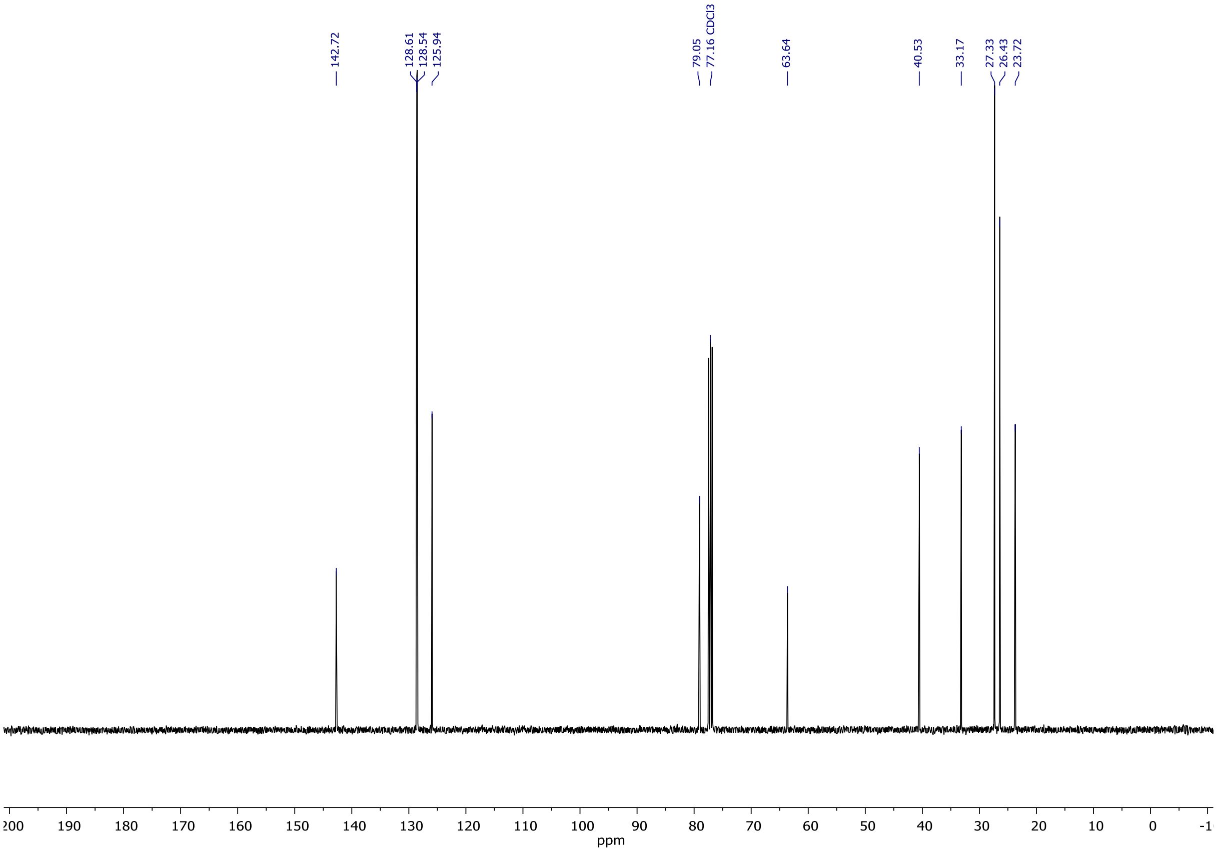

Compound **3ga**, ^{1}H- and ^{13}C-NMR ($CDCl_3$):

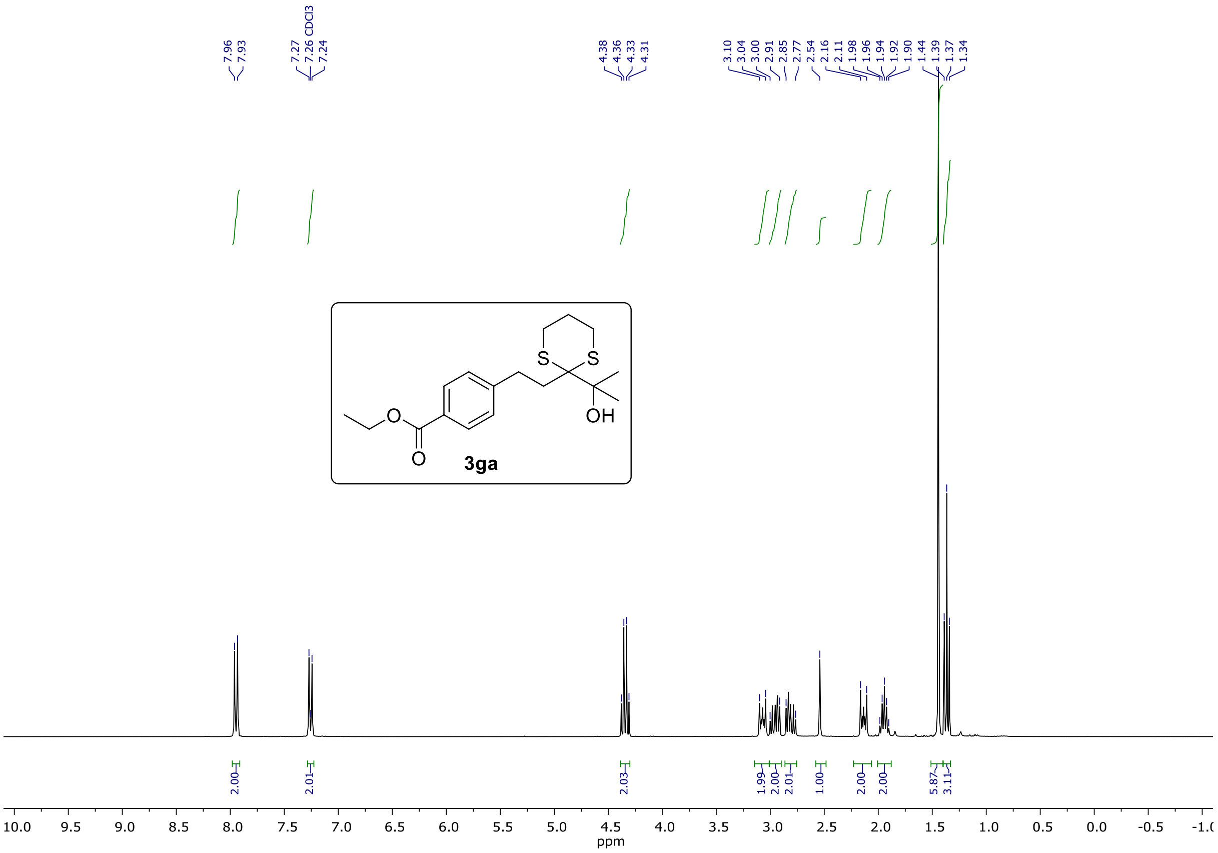

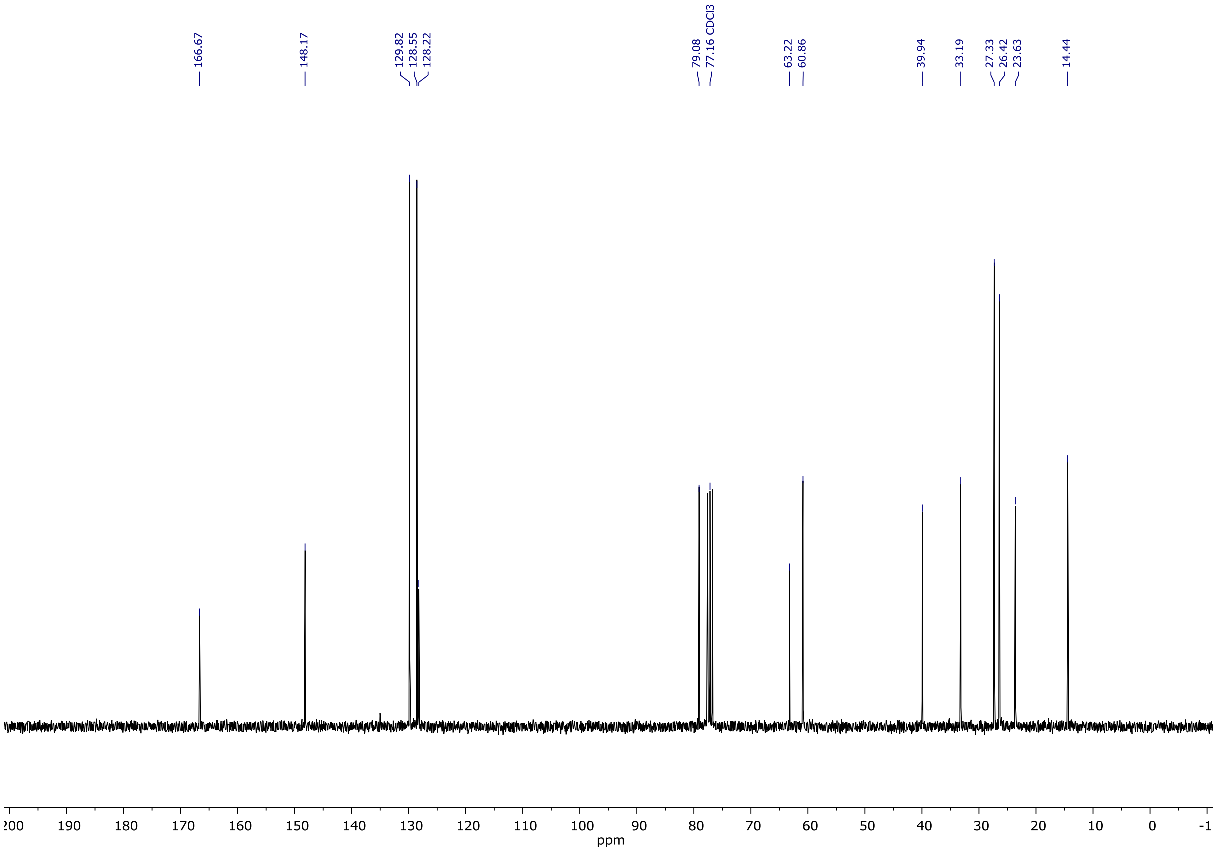

Compound **3ha**, ^{1}H- and ^{13}C-NMR ($CDCl_3$):

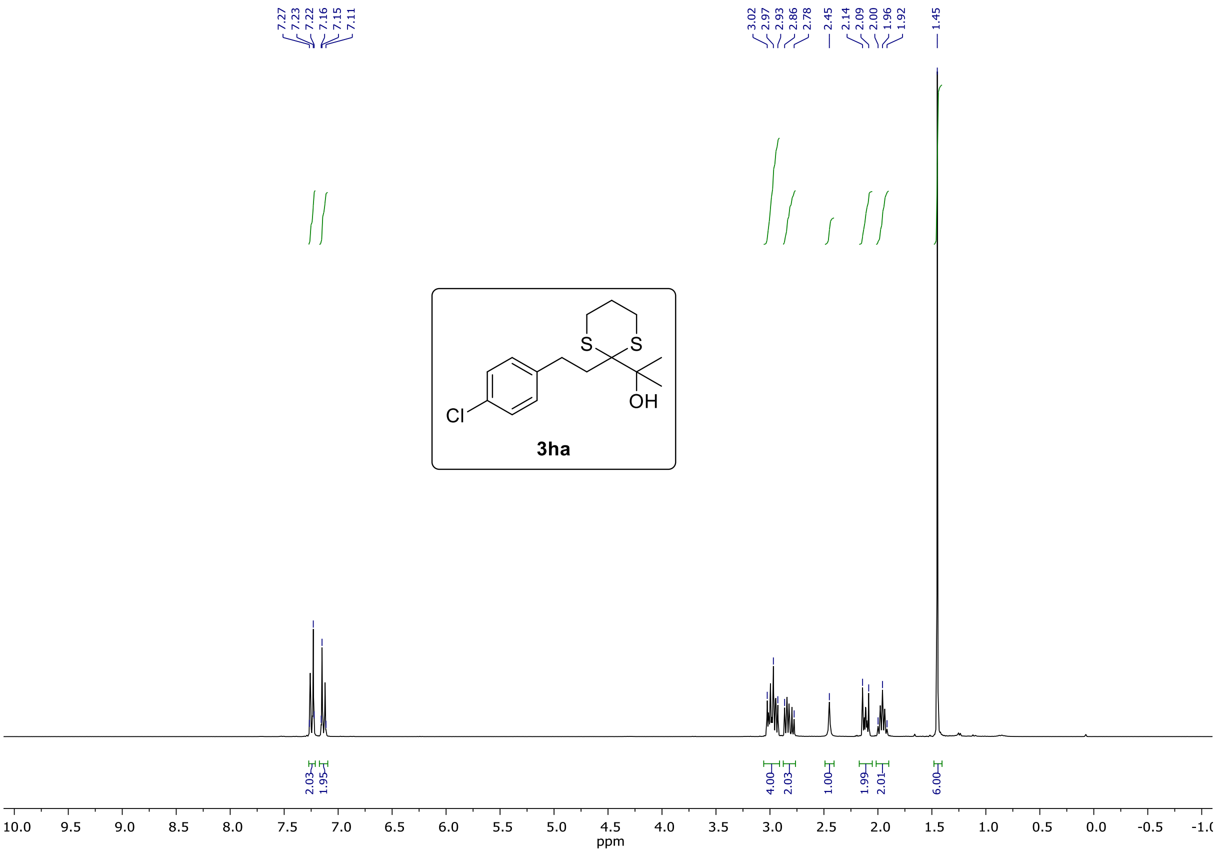

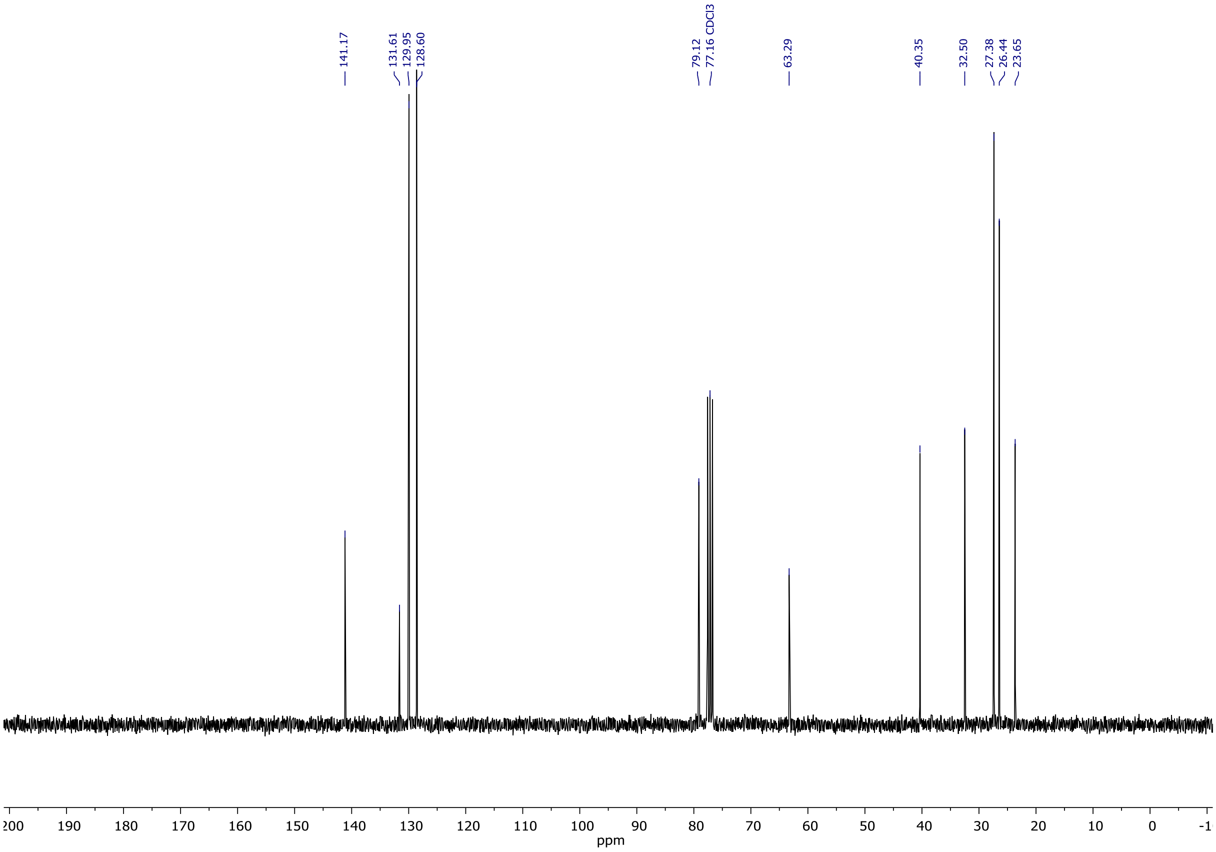

Compound **3ia**, ^{1}H- and ^{13}C-NMR ($CDCl_3$):

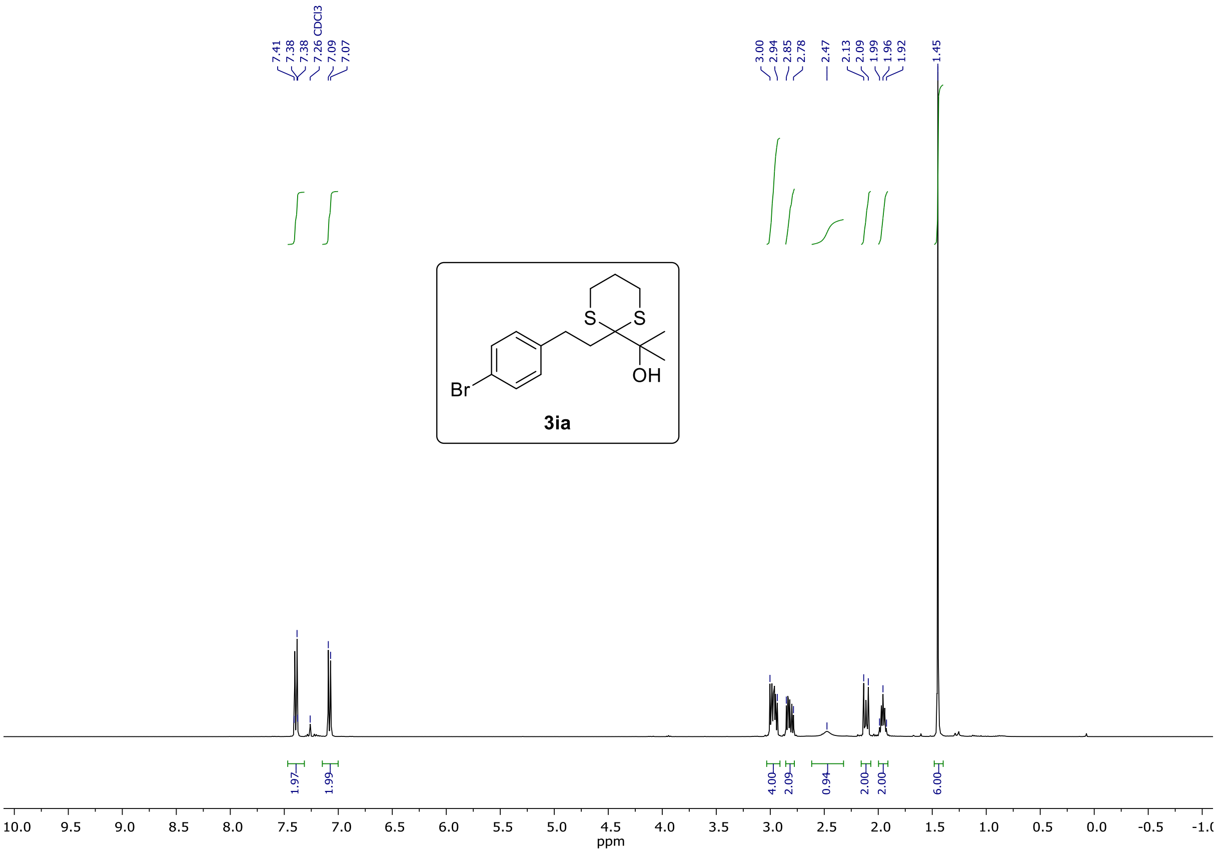

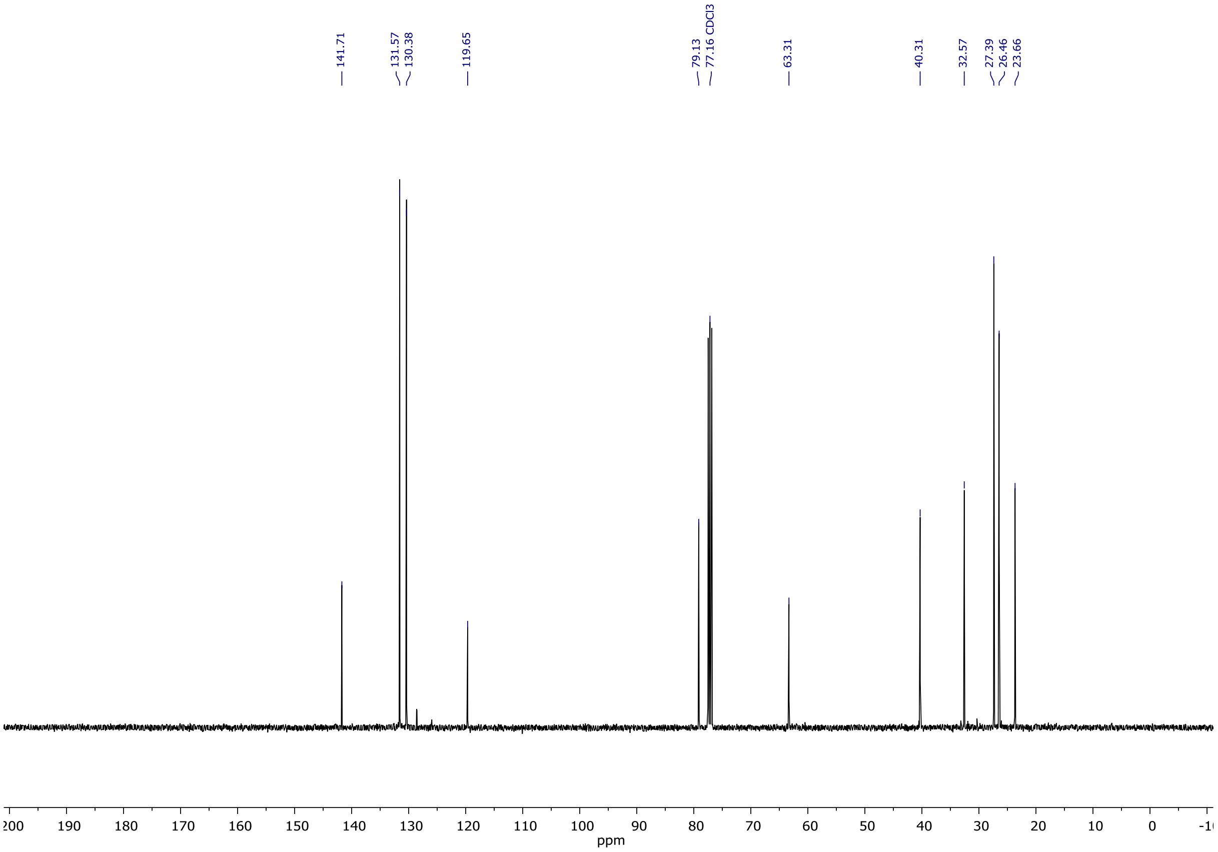

Compound **3ja**, ^{1}H- and ^{13}C-NMR ($CDCl_3$):

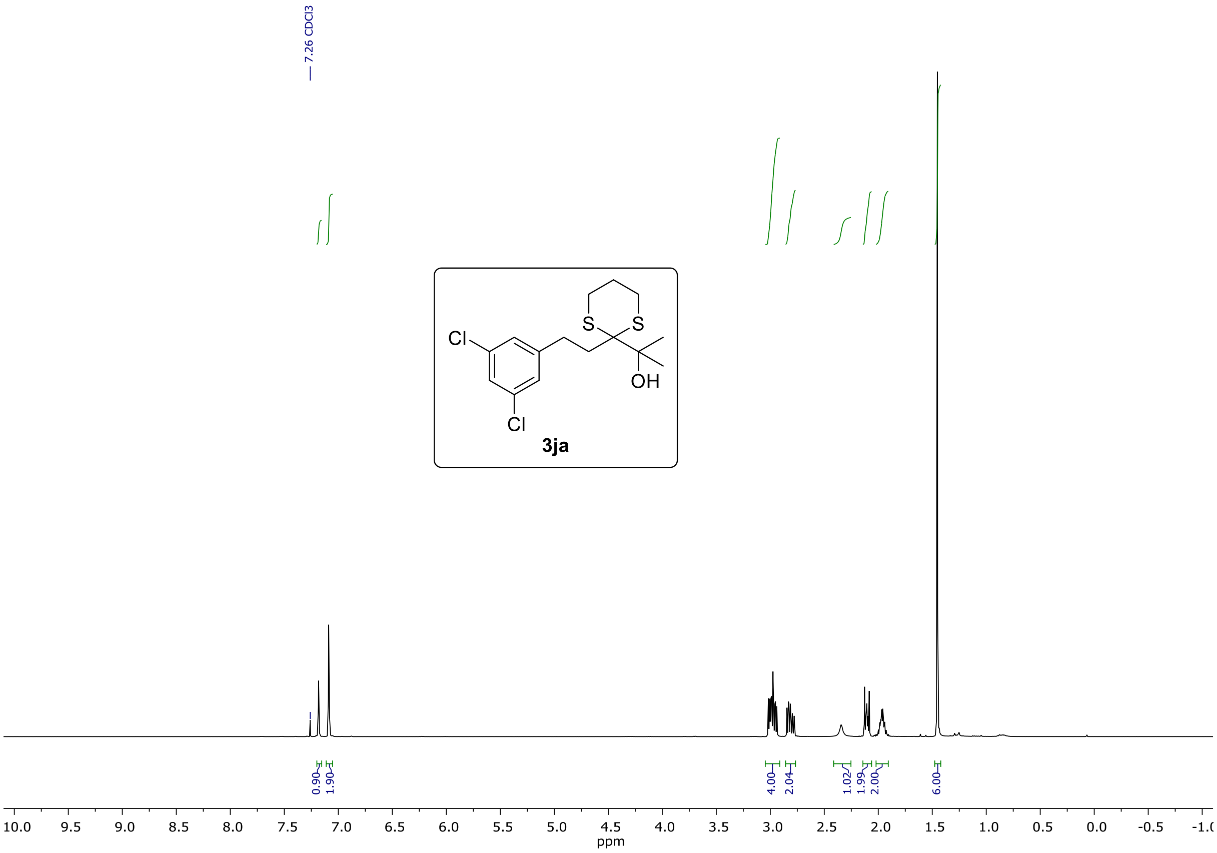

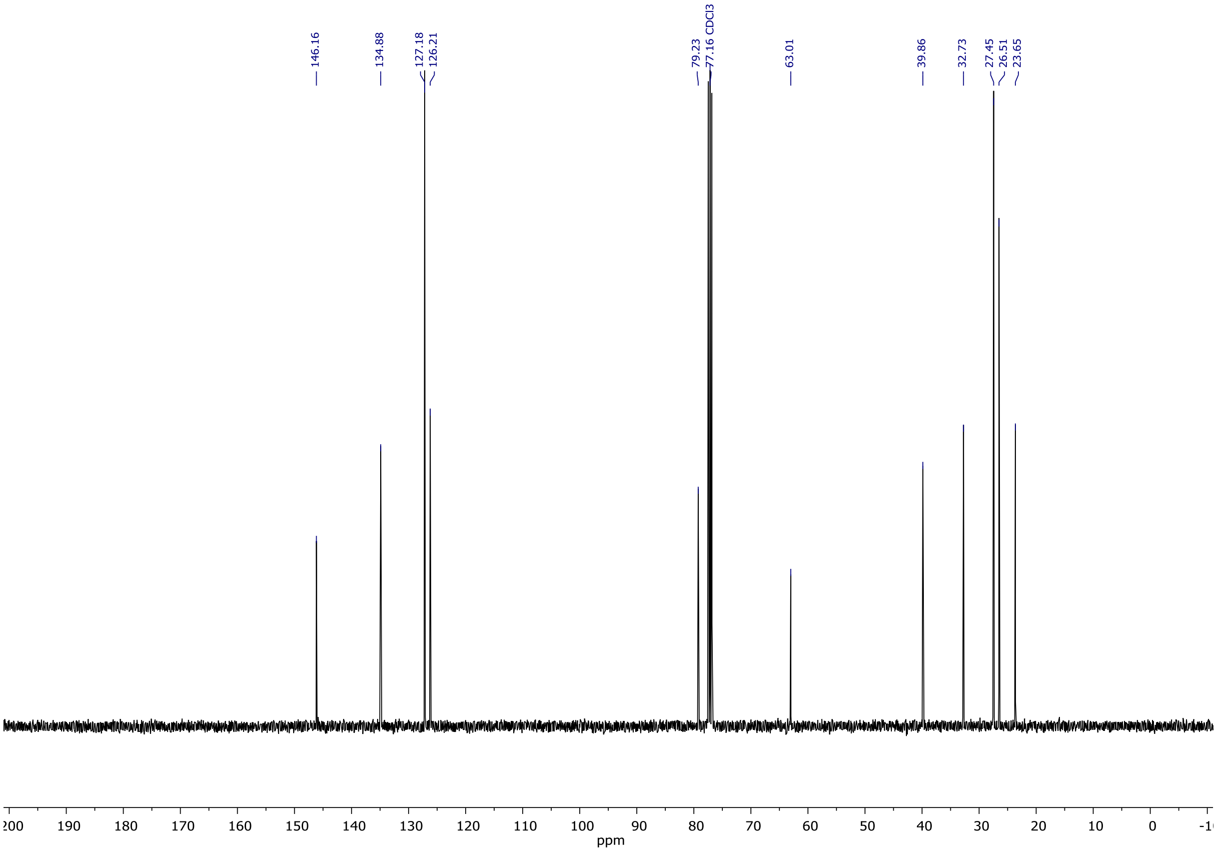

Compound **3ma**, ^{1}H-NMR and ^{13}C-NMR ($CDCl_3$):

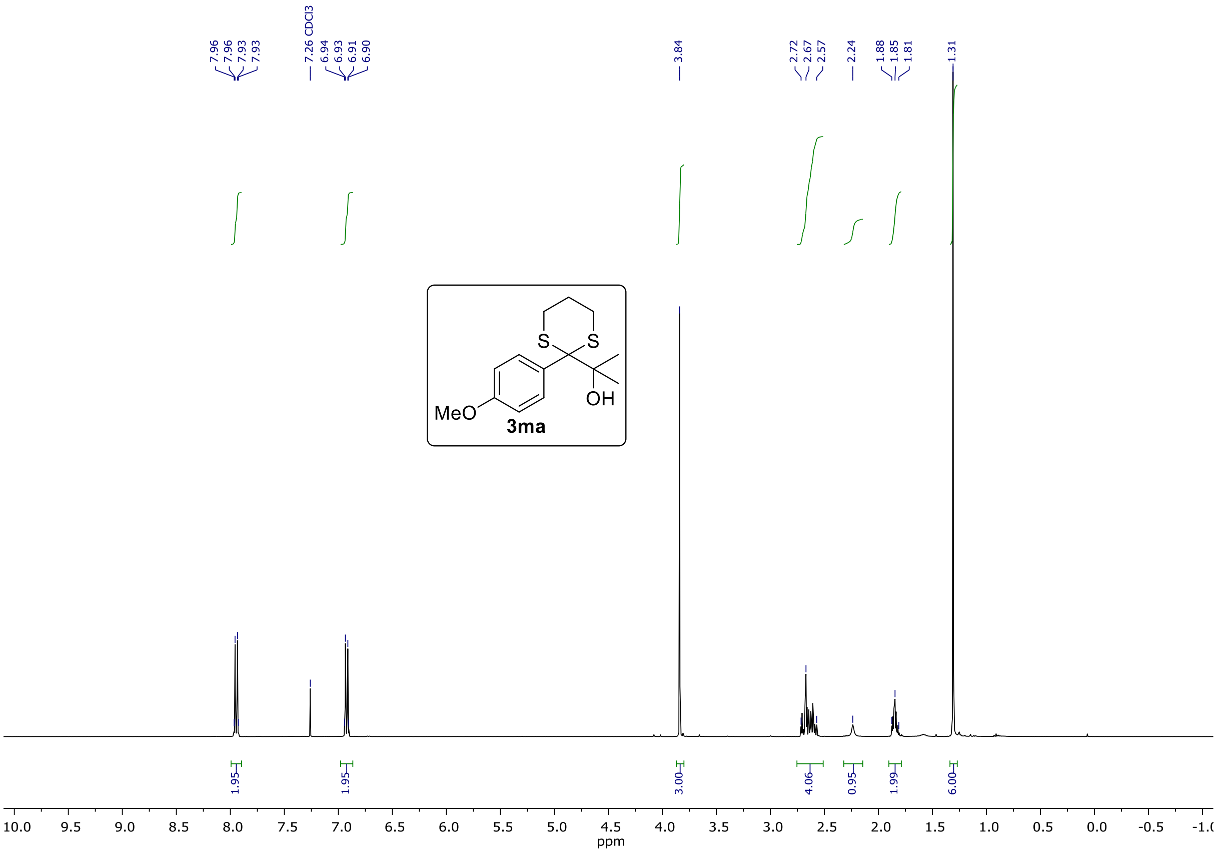

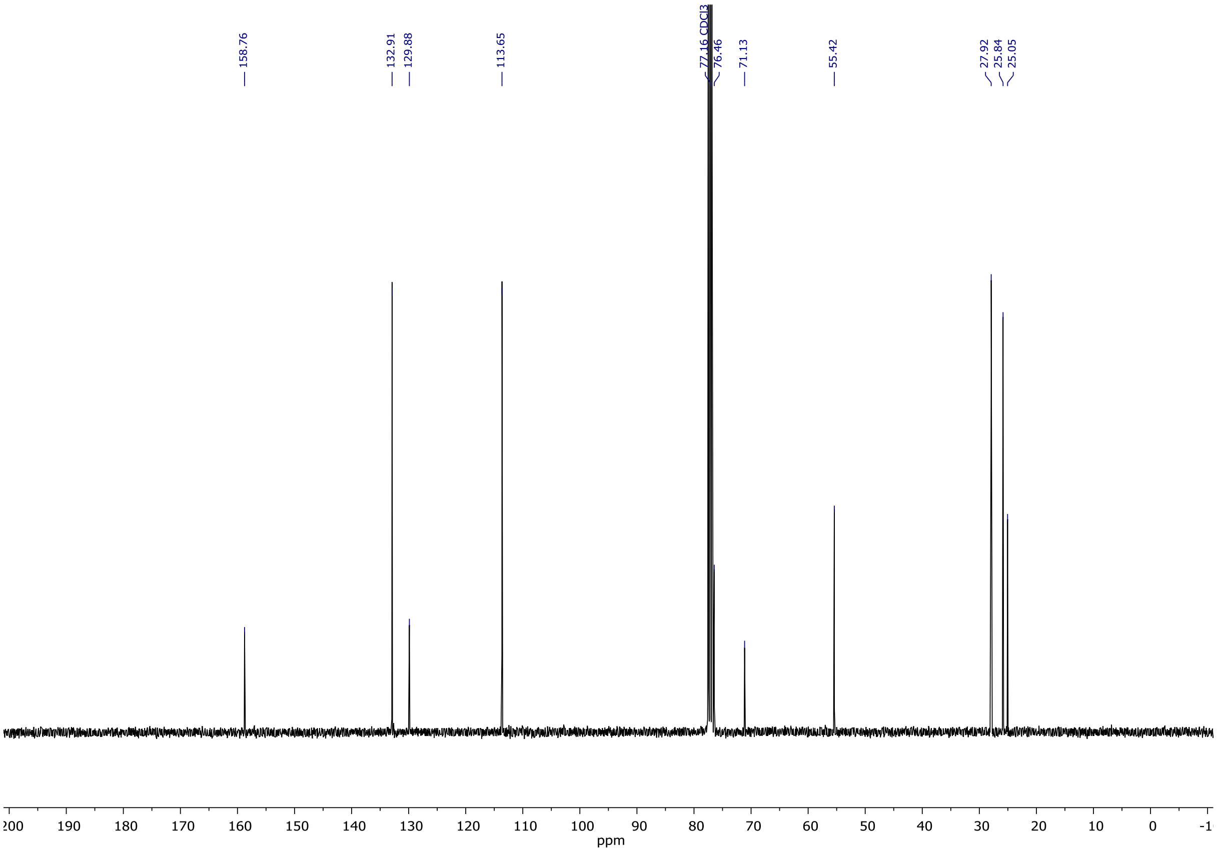

Compound **3na**, ^{1}H-NMR, ^{13}C-NMR and HMBC ($CDCl_3$):

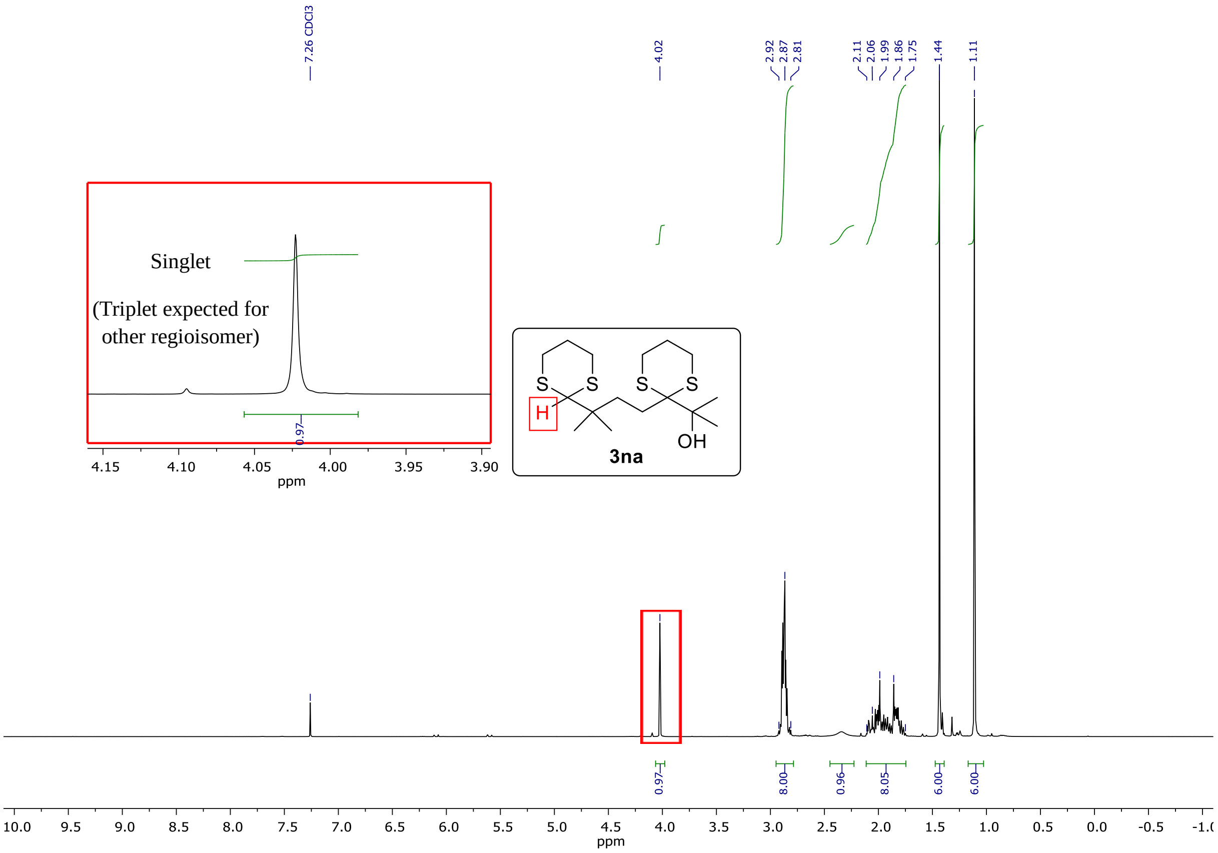

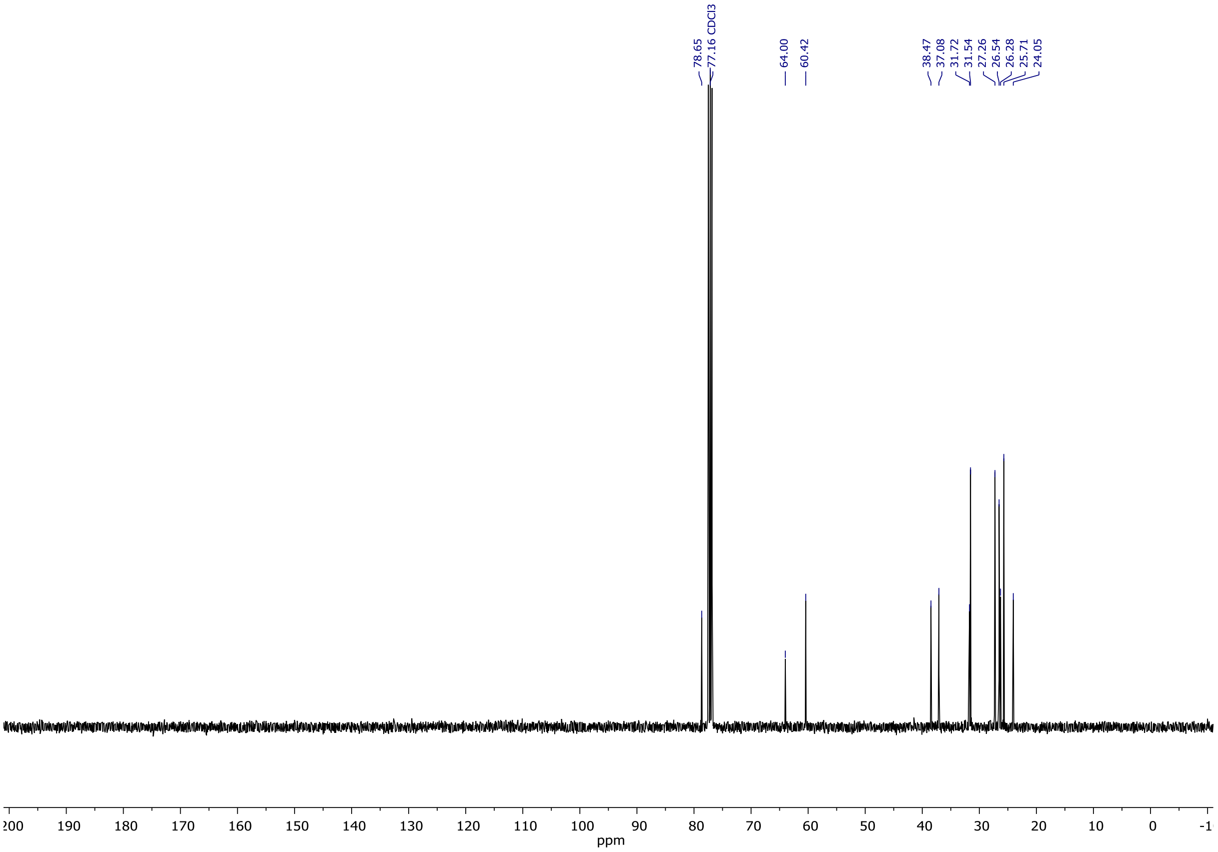

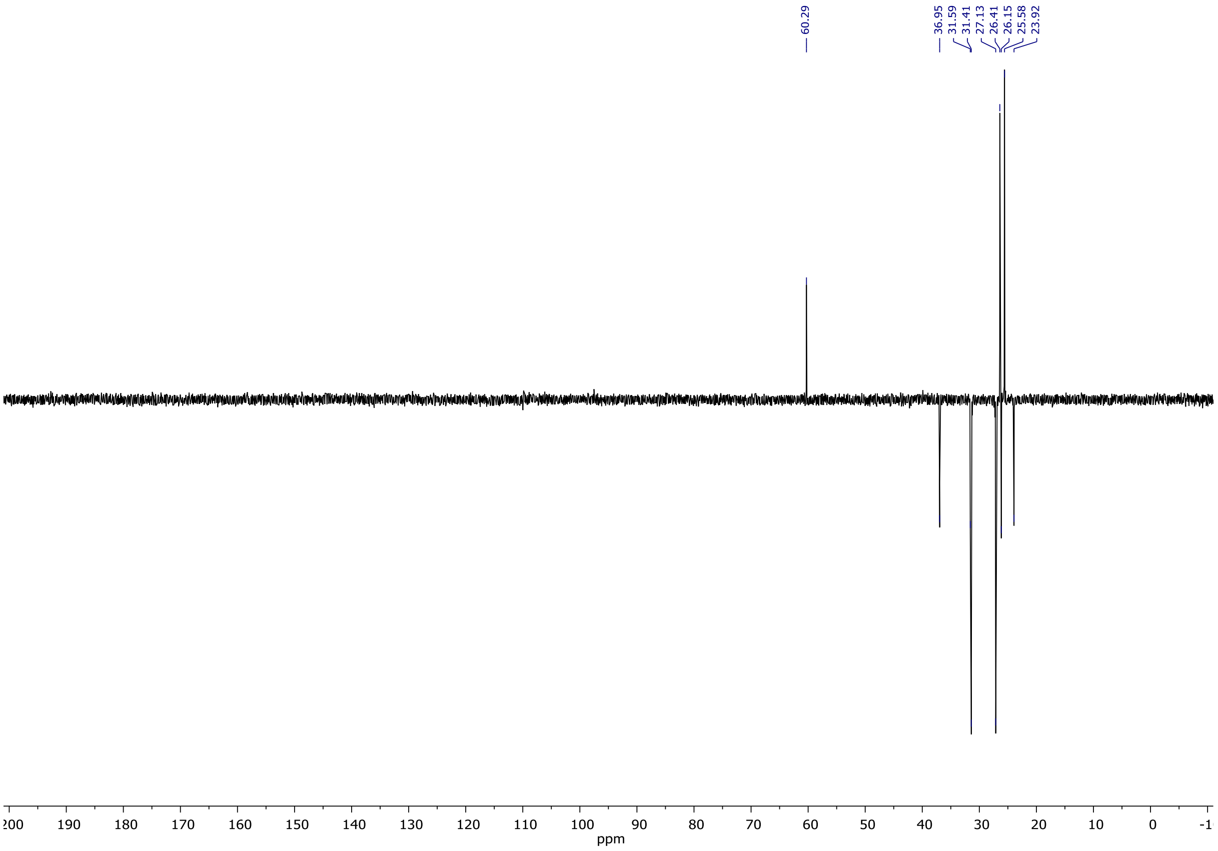
60.29
36.95
31.59
31.41
27.13
26.41
26.15
25.58
23.92
ppm

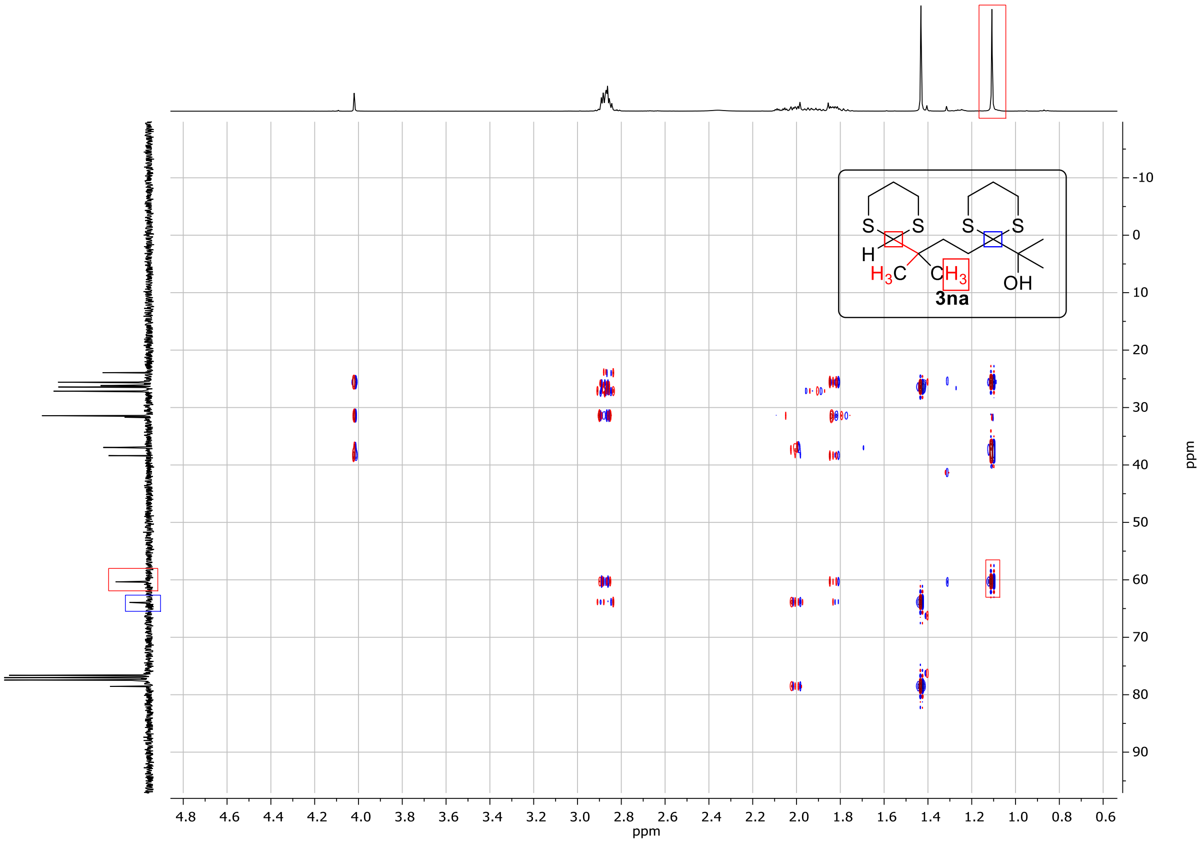
3na
OH
ppm

Compound **3ab**, ^{1}H- and ^{13}C-NMR ($CDCl_3$):

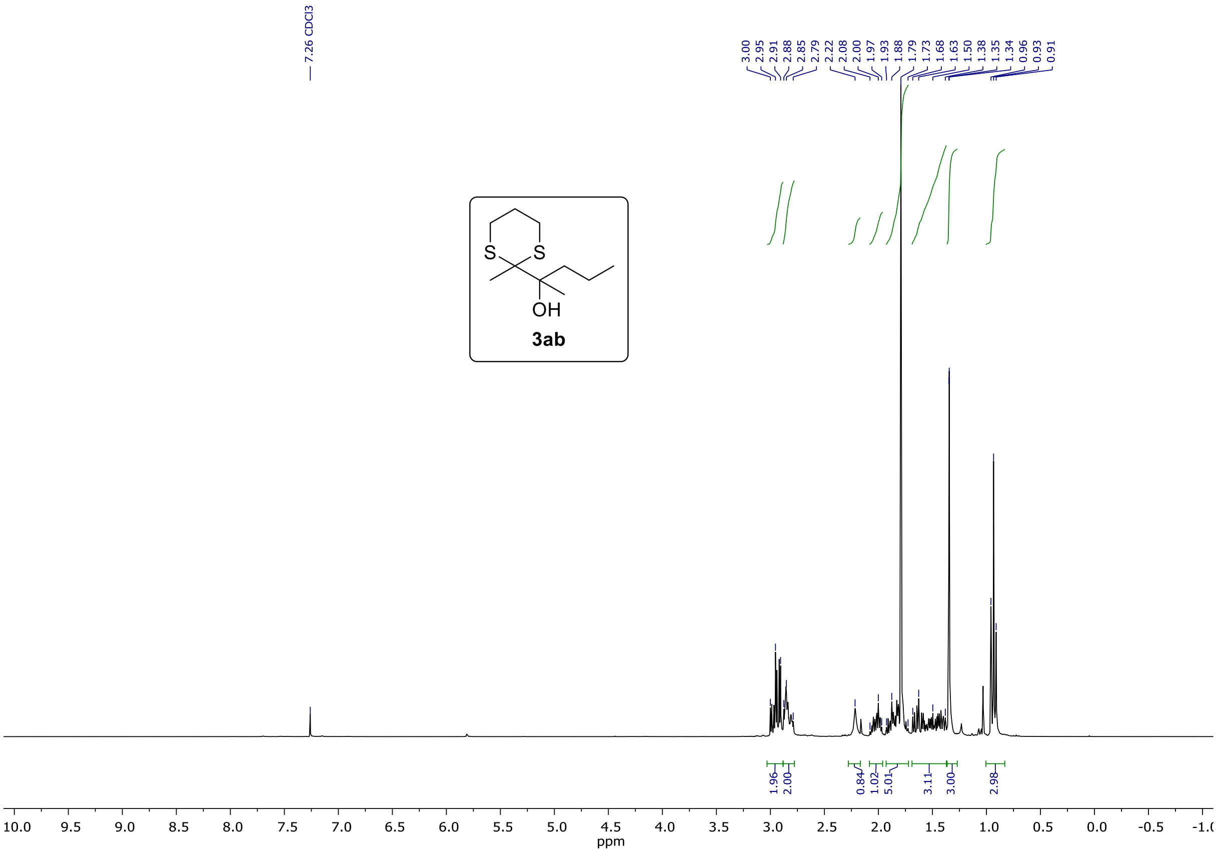

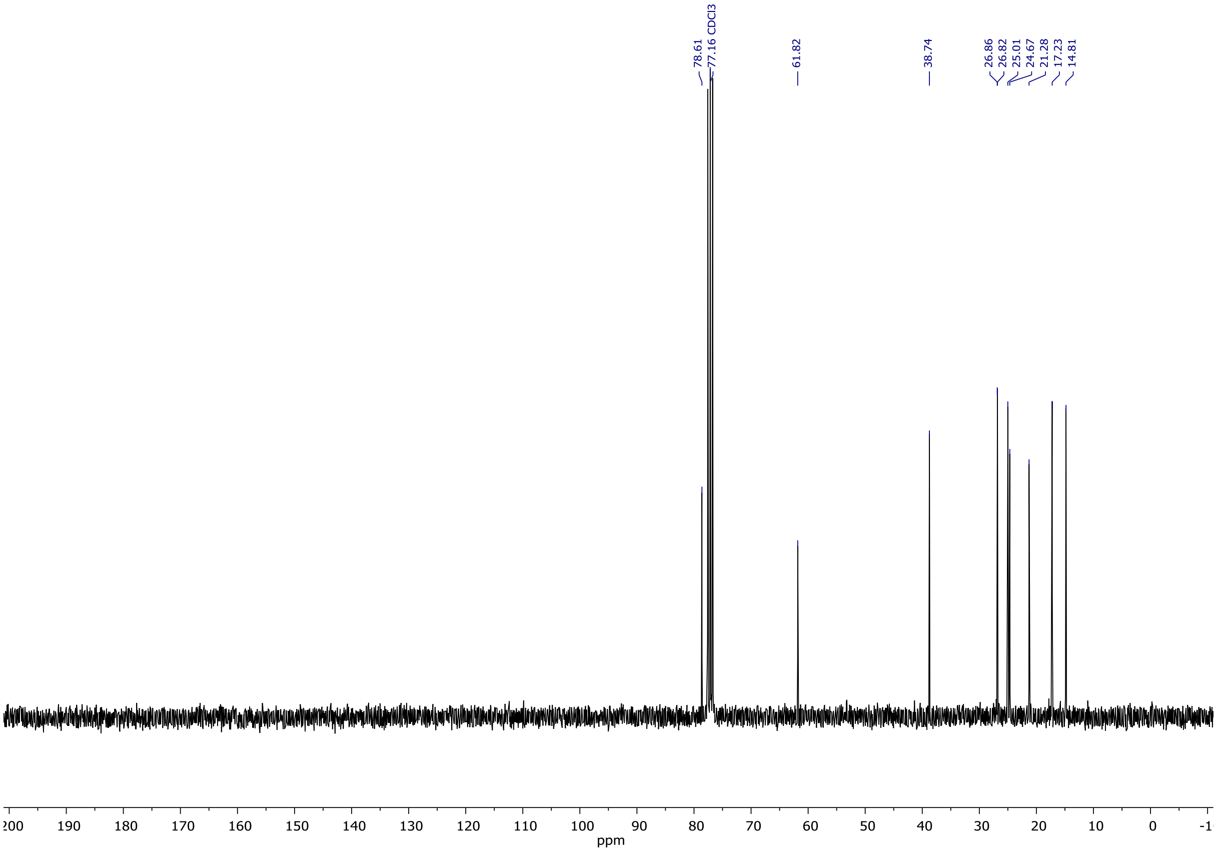

Compound **3ac**, ^{1}H- and ^{13}C-NMR ($CDCl_3$):

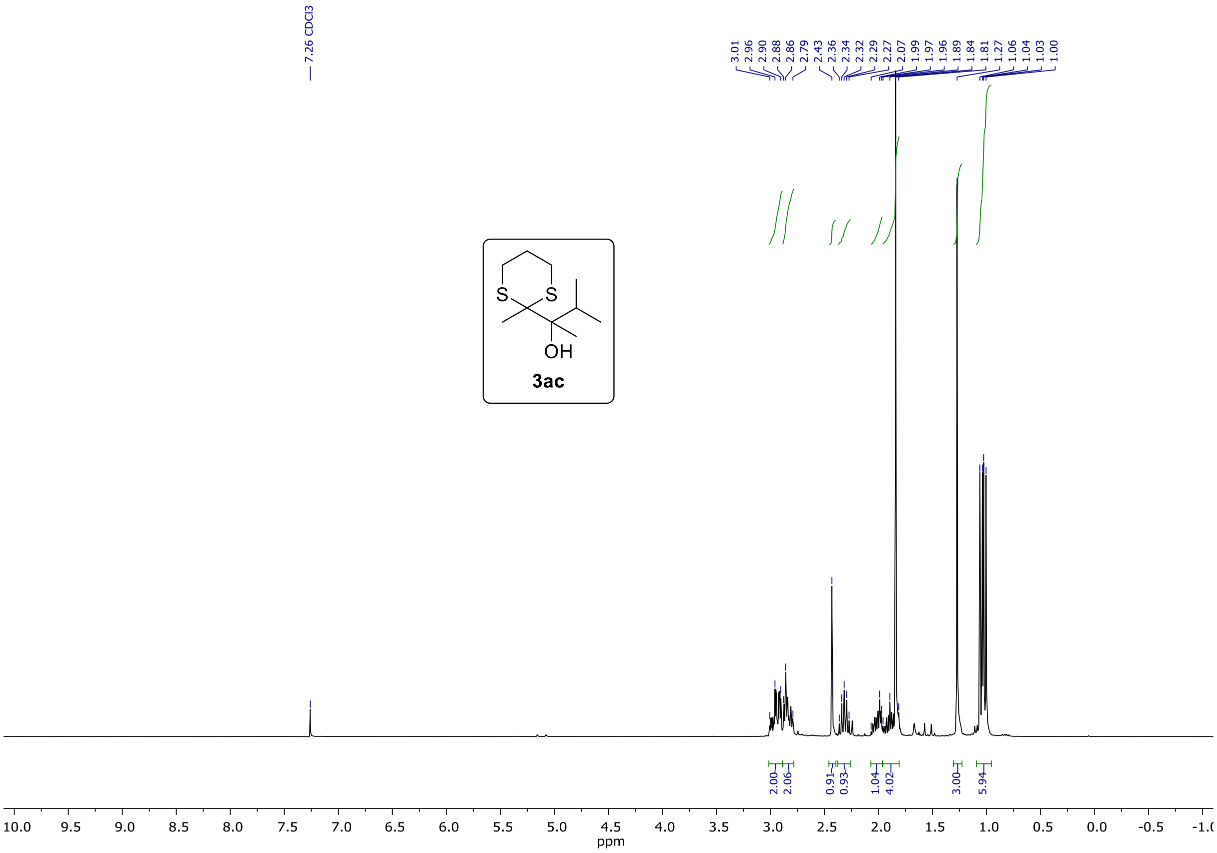

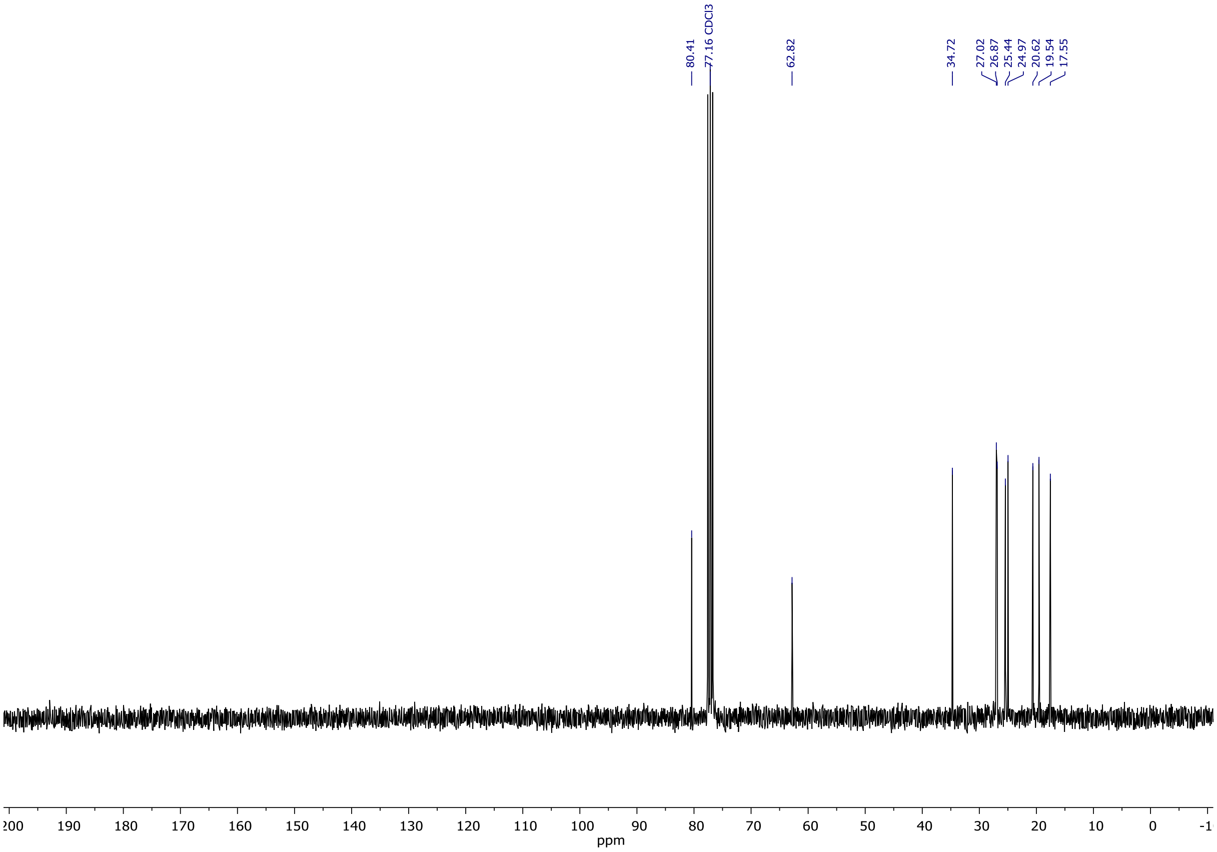

Compound **3ad**, ^{1}H- and ^{13}C-NMR ($CDCl_3$):

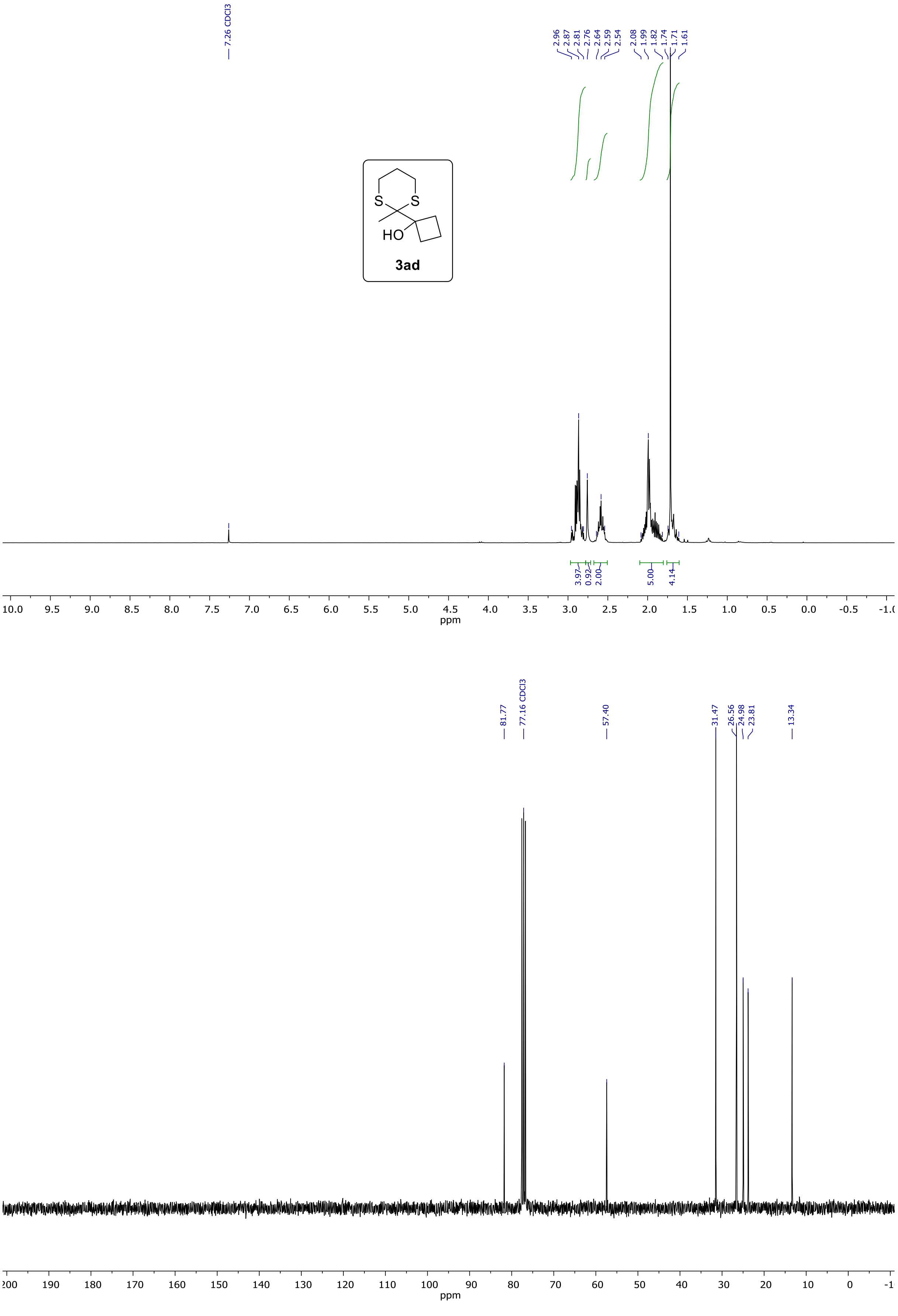

Compound **3ae**, ^{1}H- and ^{13}C-NMR ($CDCl_3$):

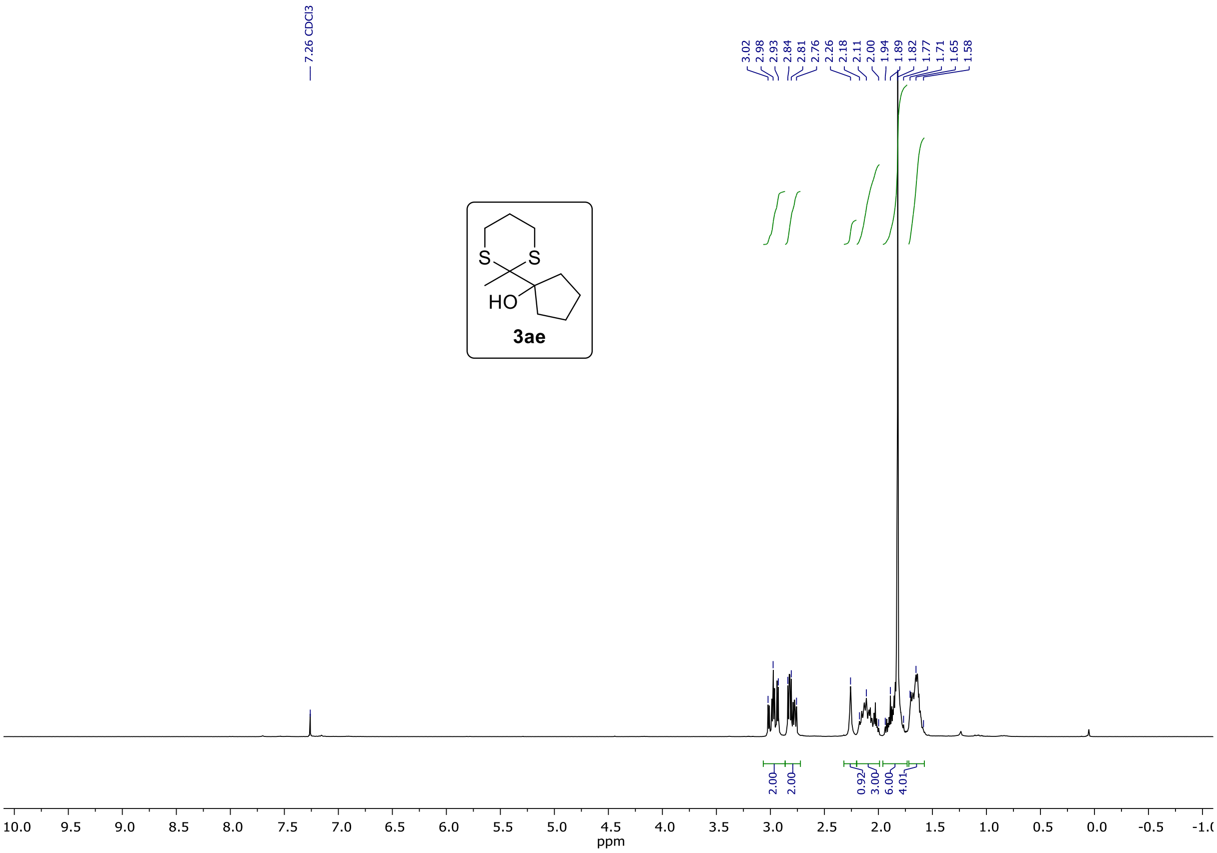

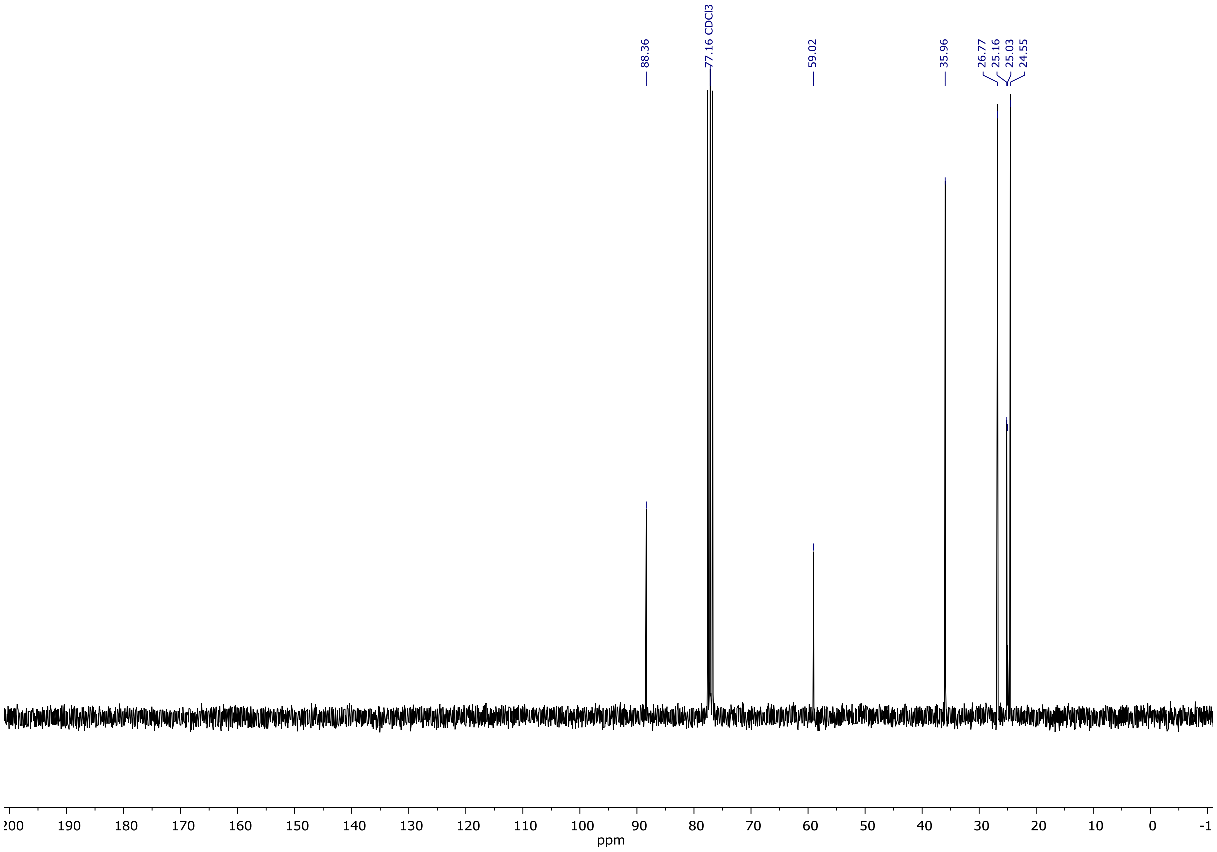

Compound **3af**, ^{1}H- and ^{13}C-NMR ($CDCl_3$):

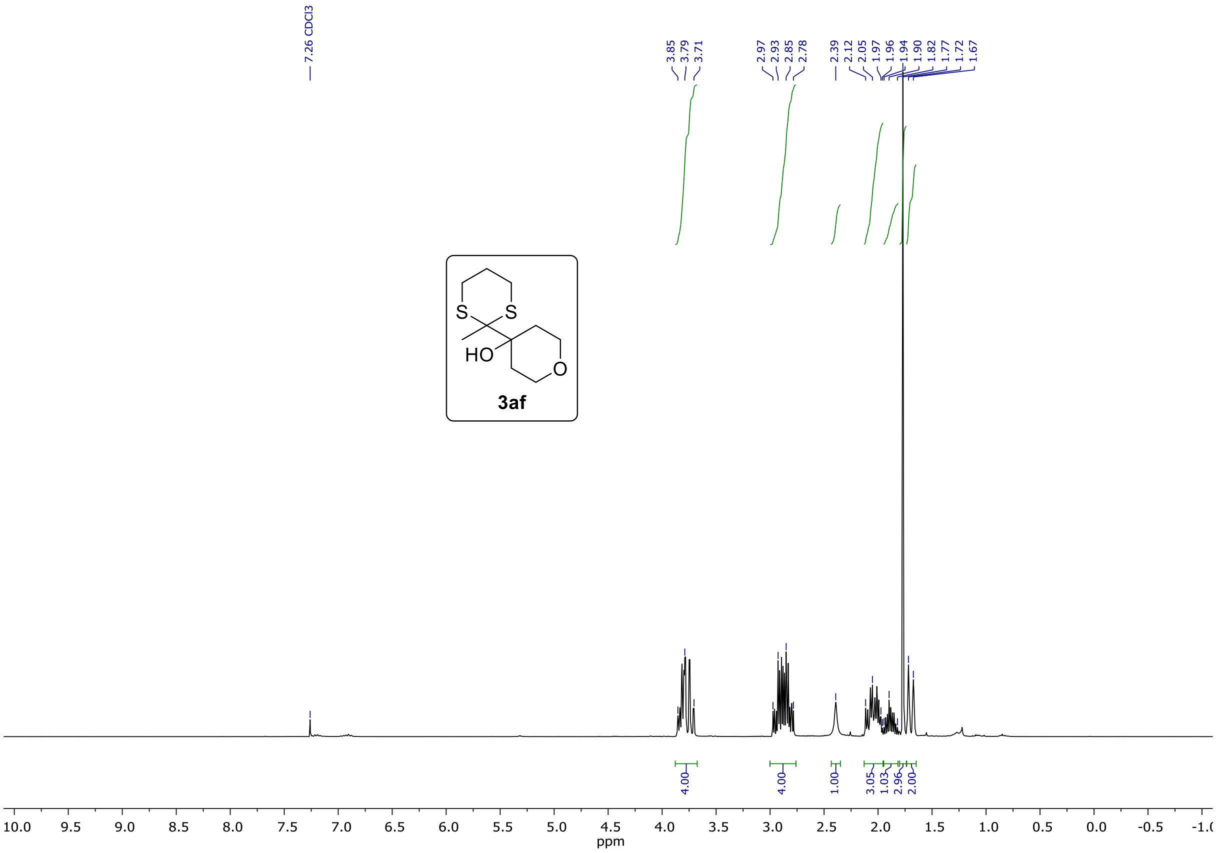

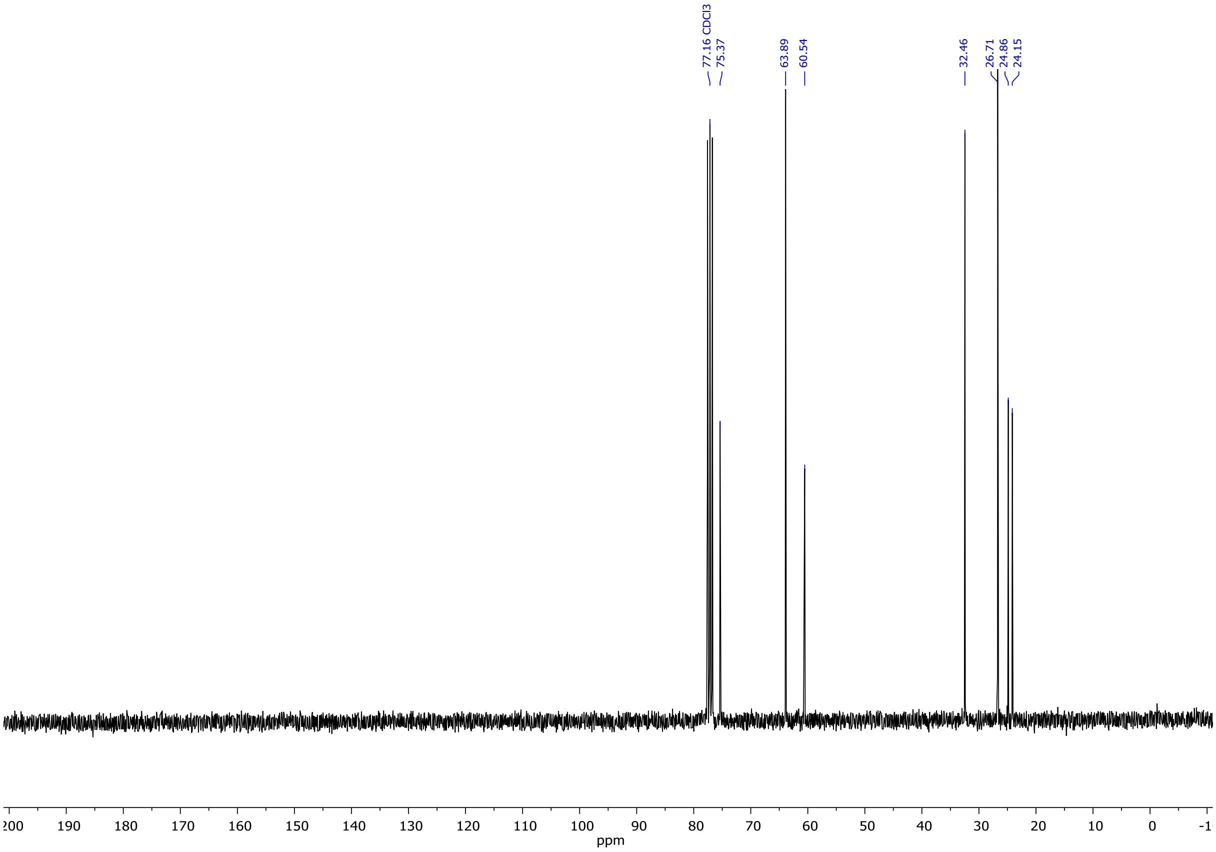

Compound **3ag**, ^{1}H- and ^{13}C-NMR ($CDCl_3$):

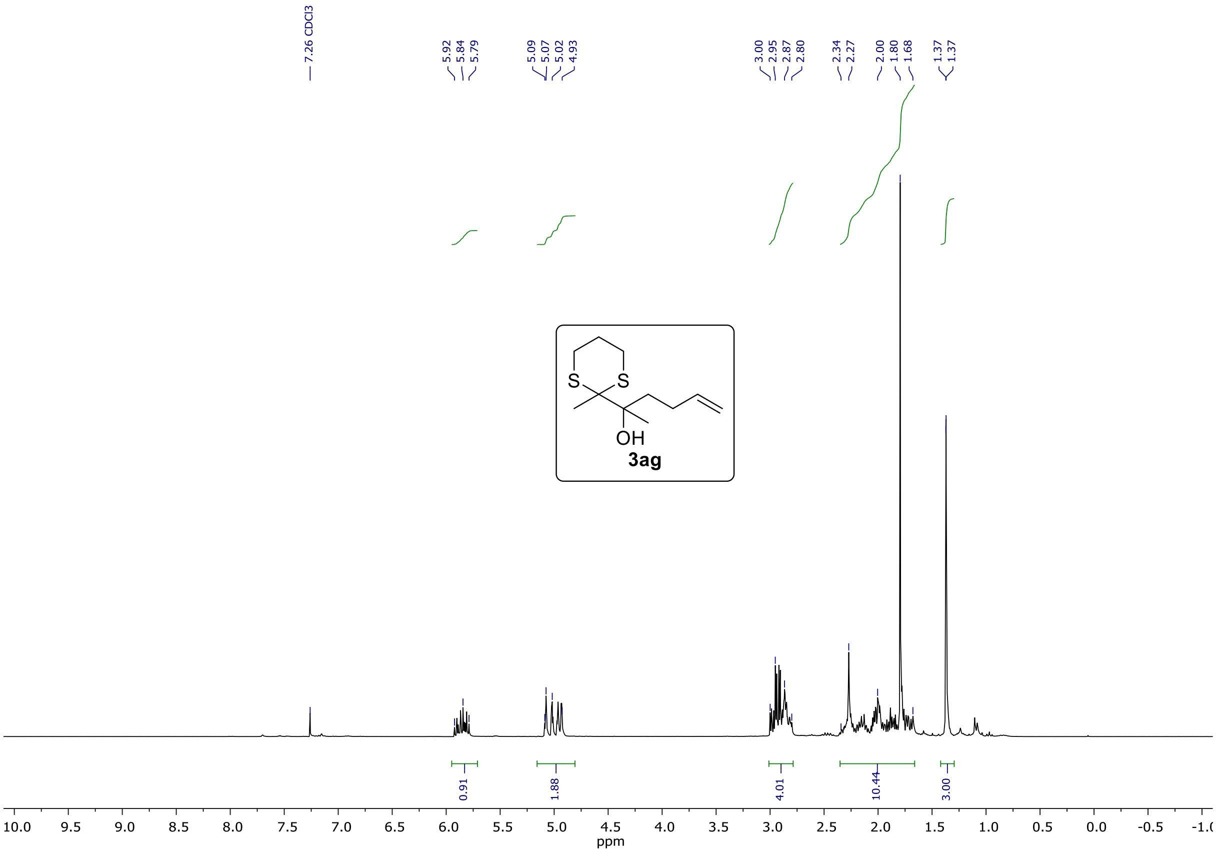

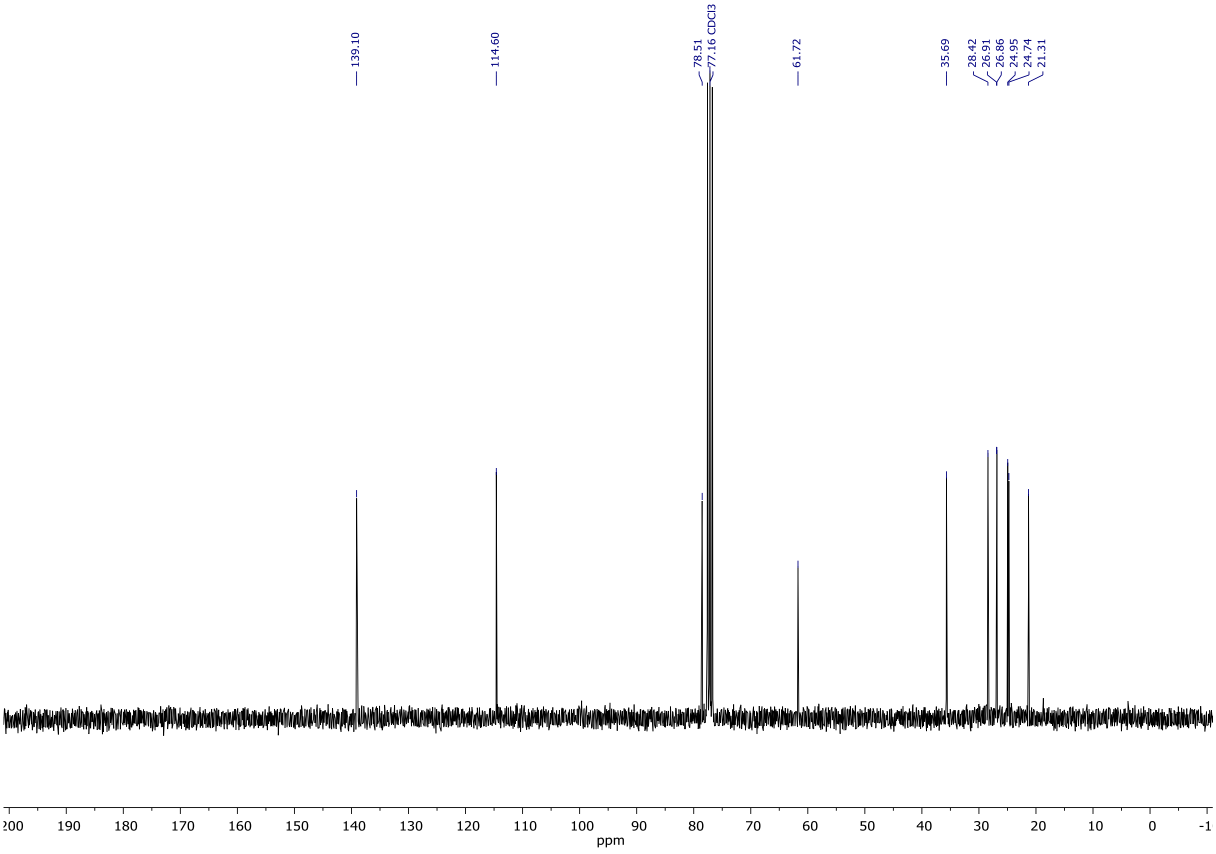

Compound **3ah**, ^{1}H- and ^{13}C-NMR ($CDCl_3$):

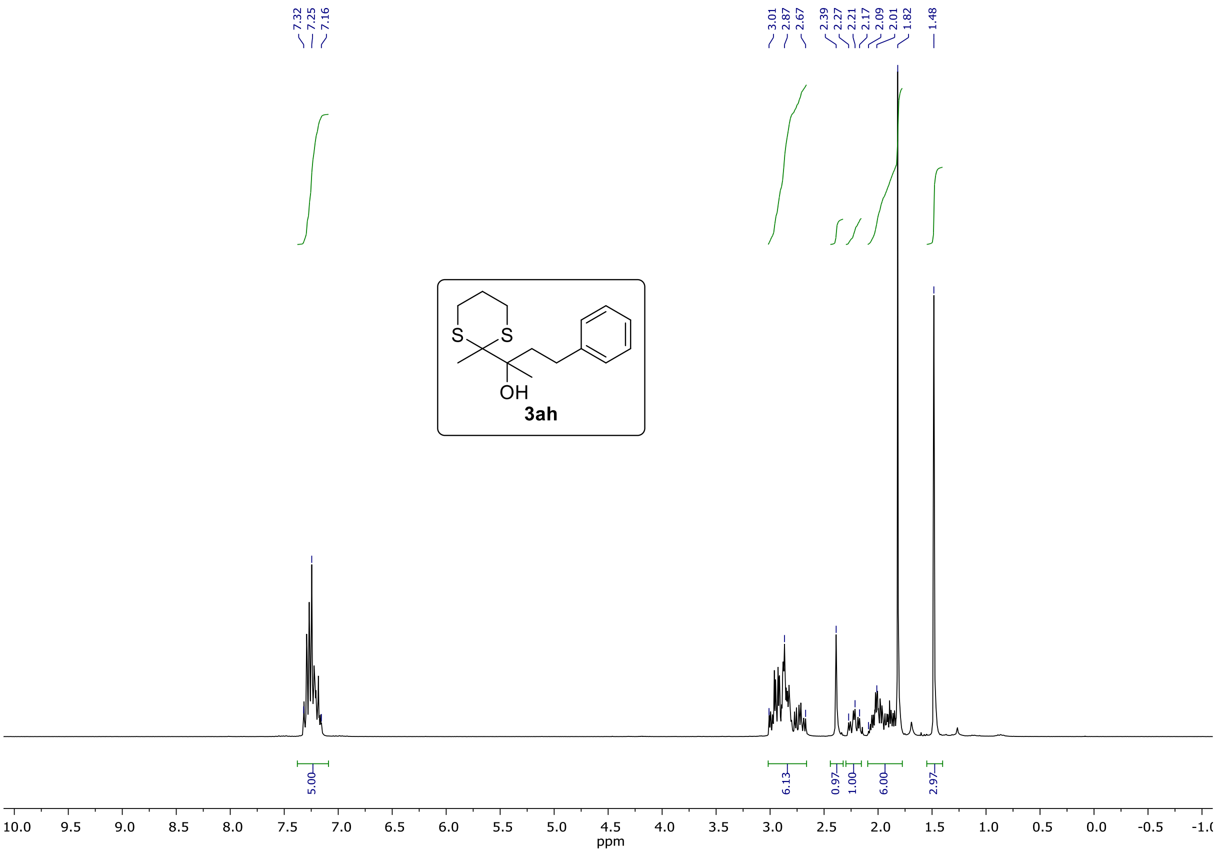

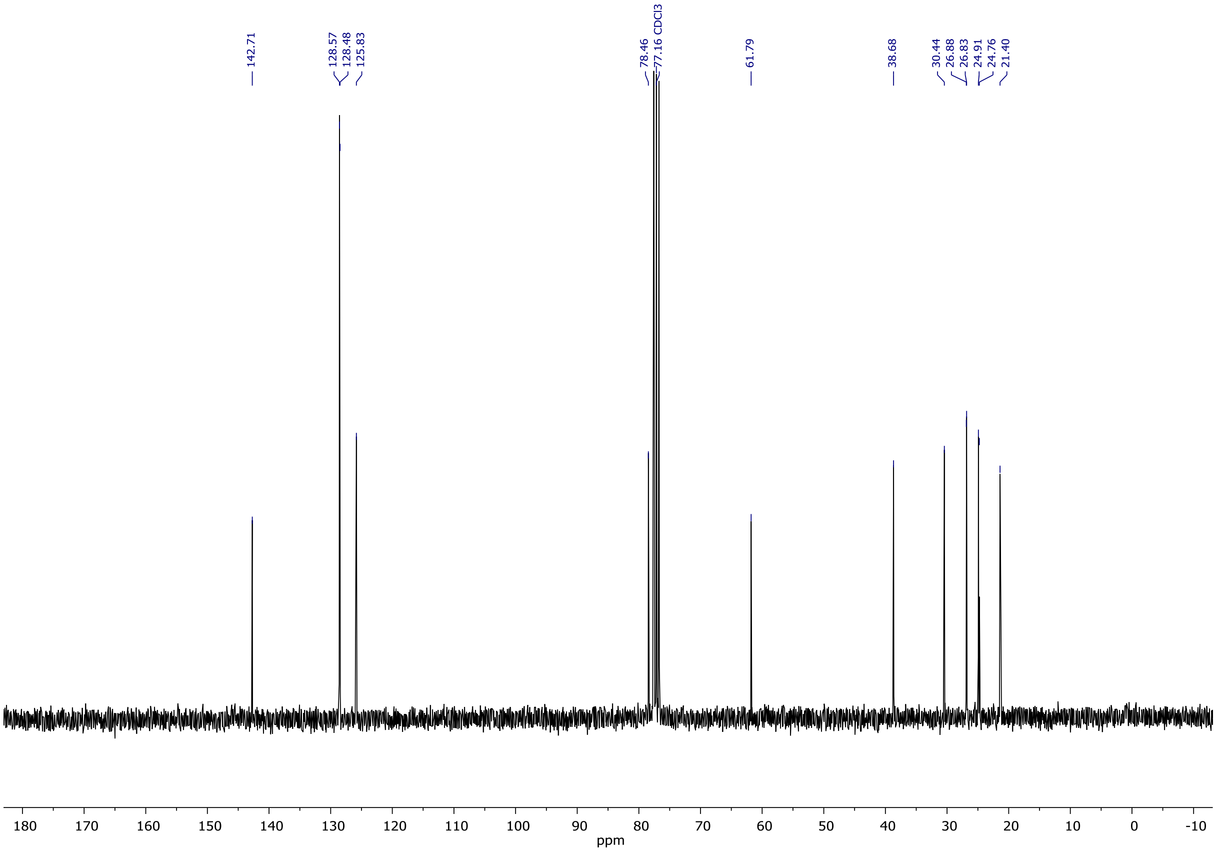

Compound **3ai**, ^{1}H- and ^{13}C-NMR ($CDCl_3$):

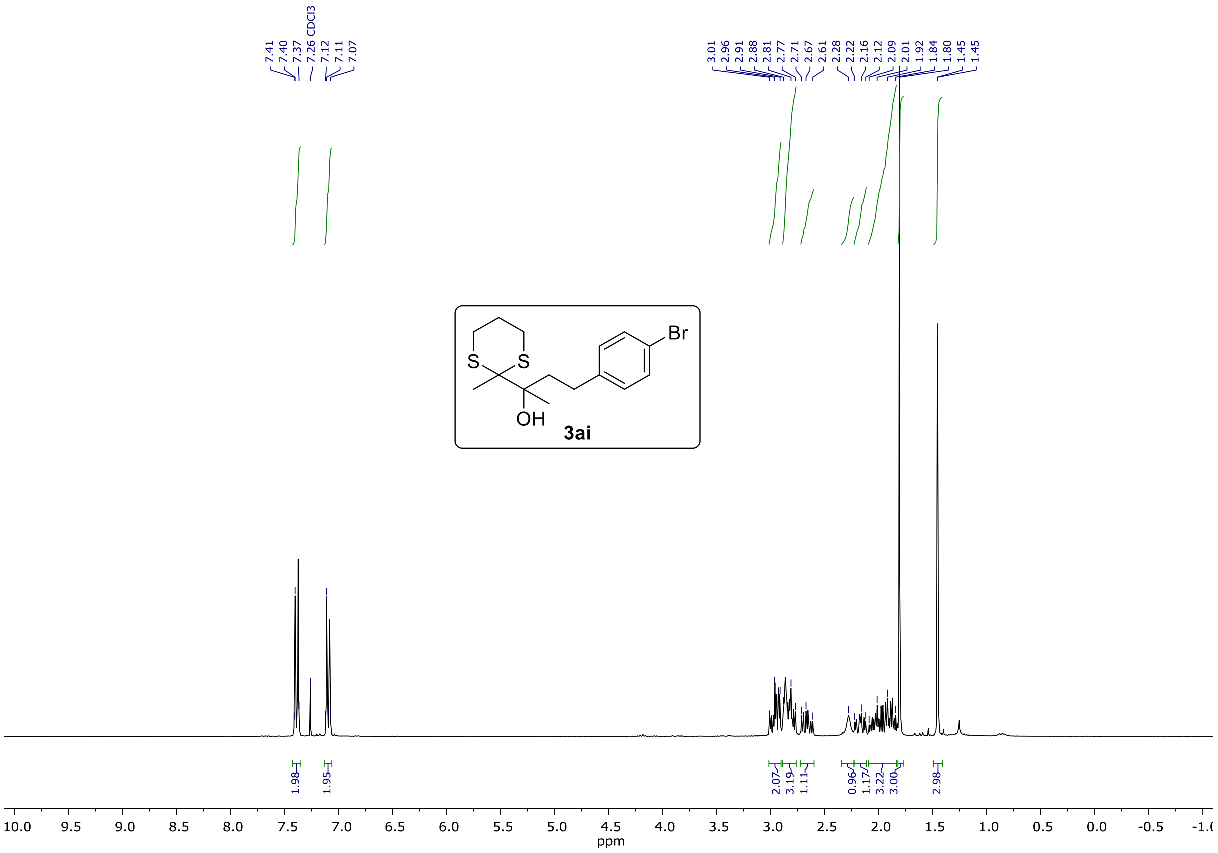

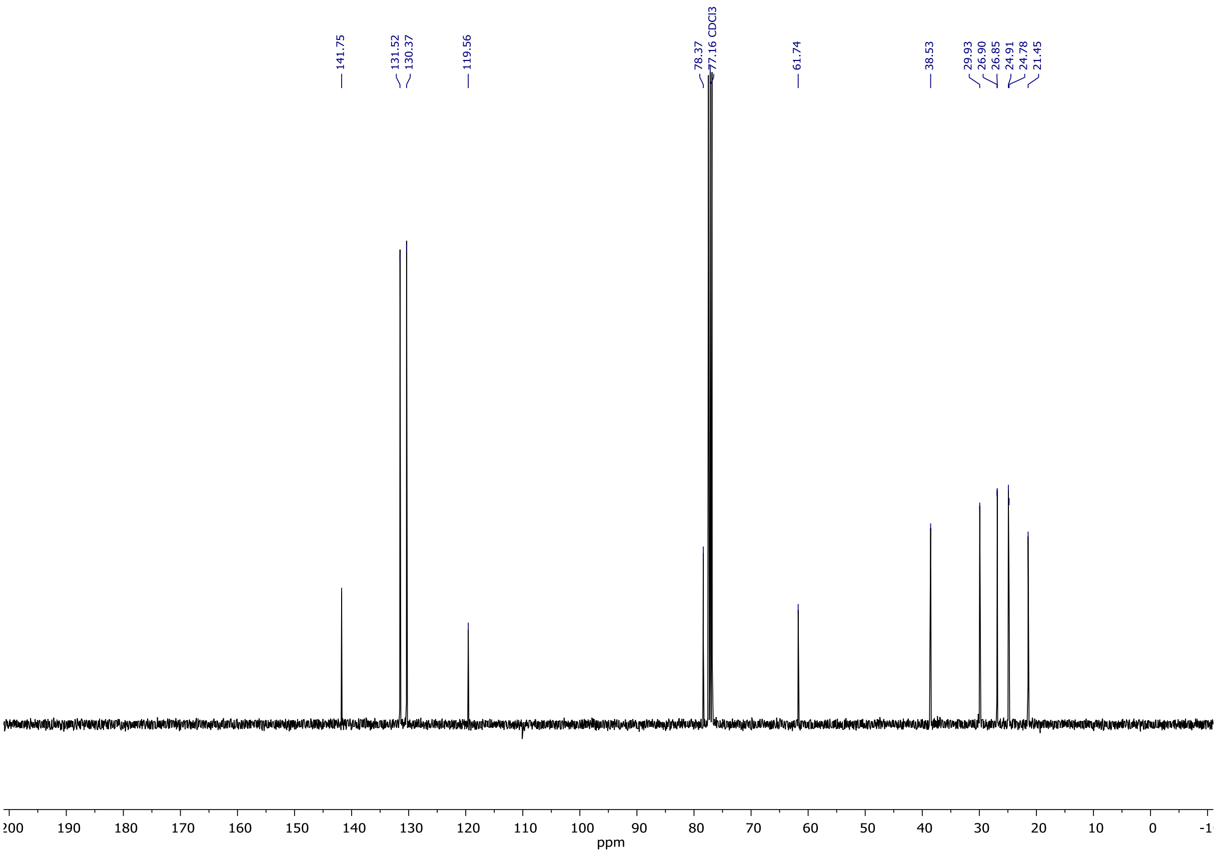

Compound **3aj**, ^{1}H- and ^{13}C-NMR ($CDCl_3$):

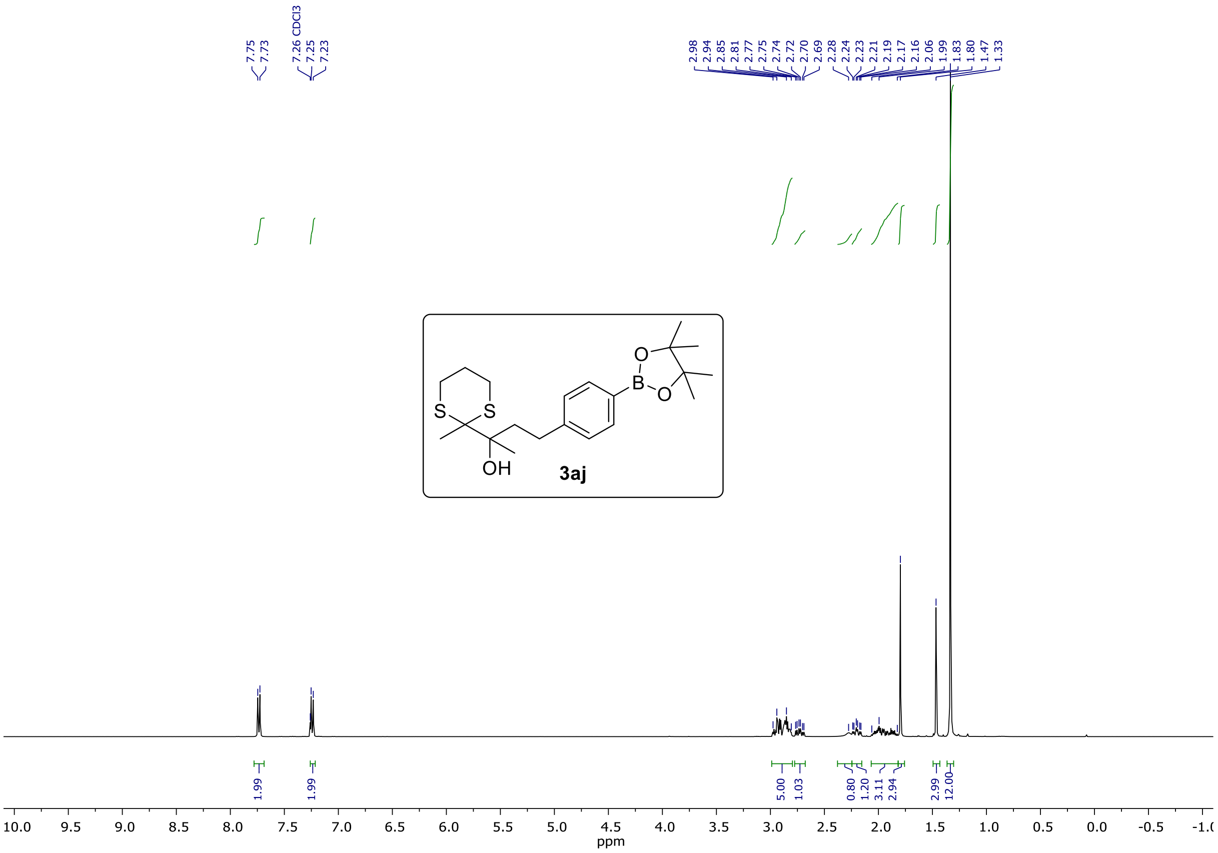

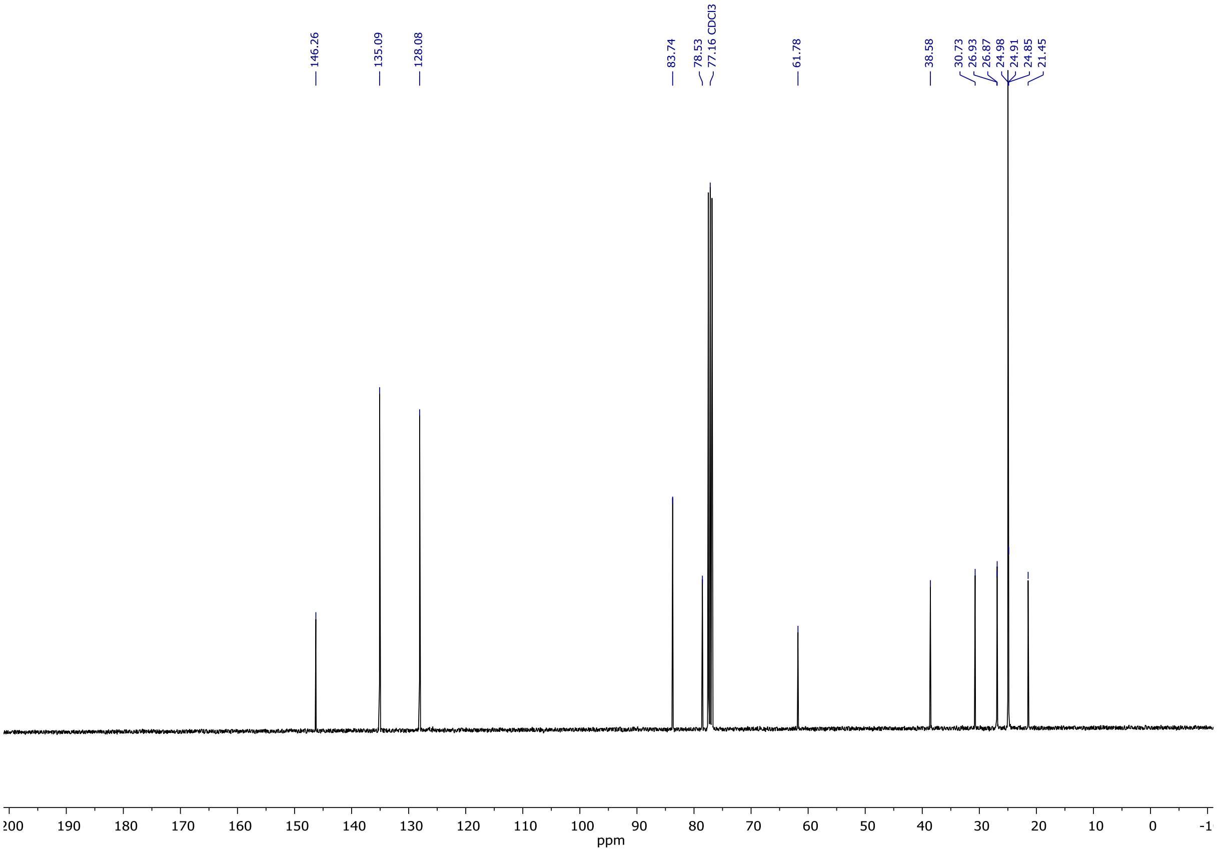

Compound **3ak**, ^{1}H- and ^{13}C-NMR ($CDCl_3$):

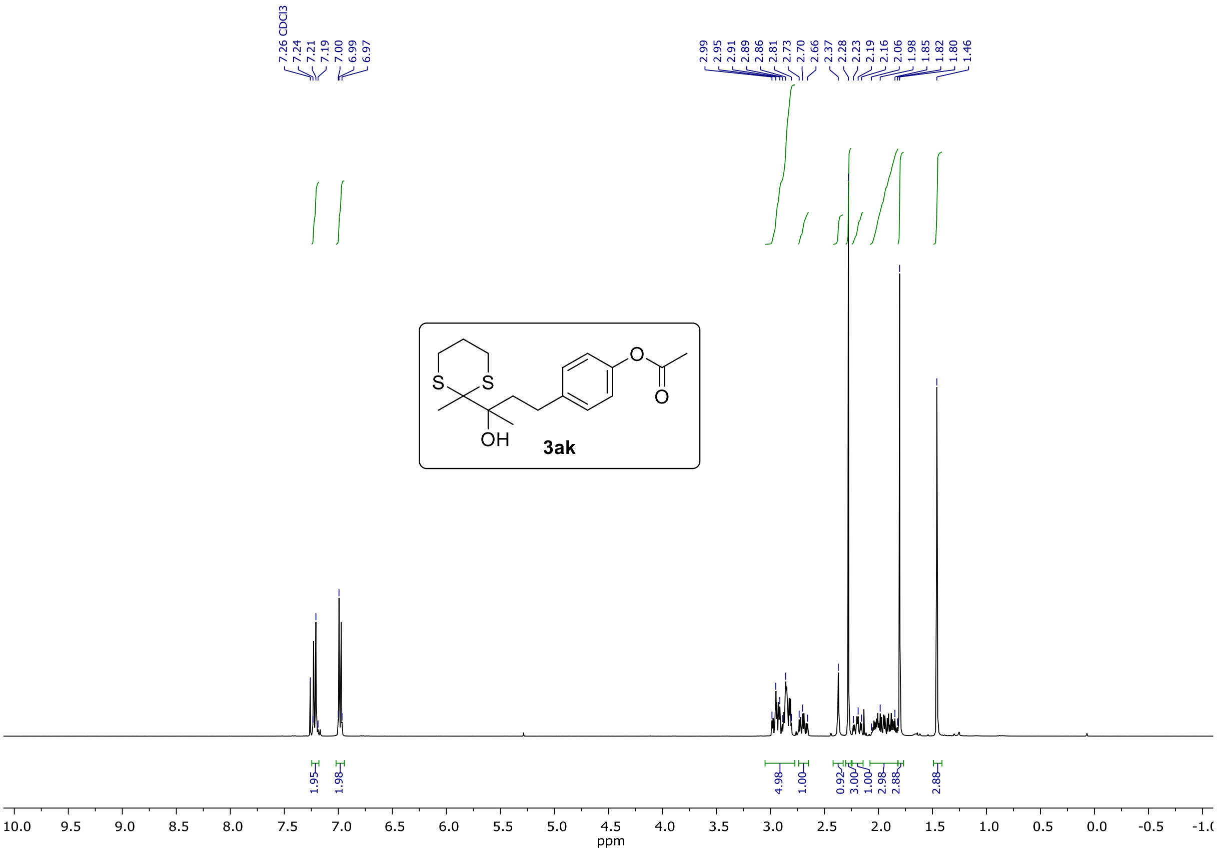

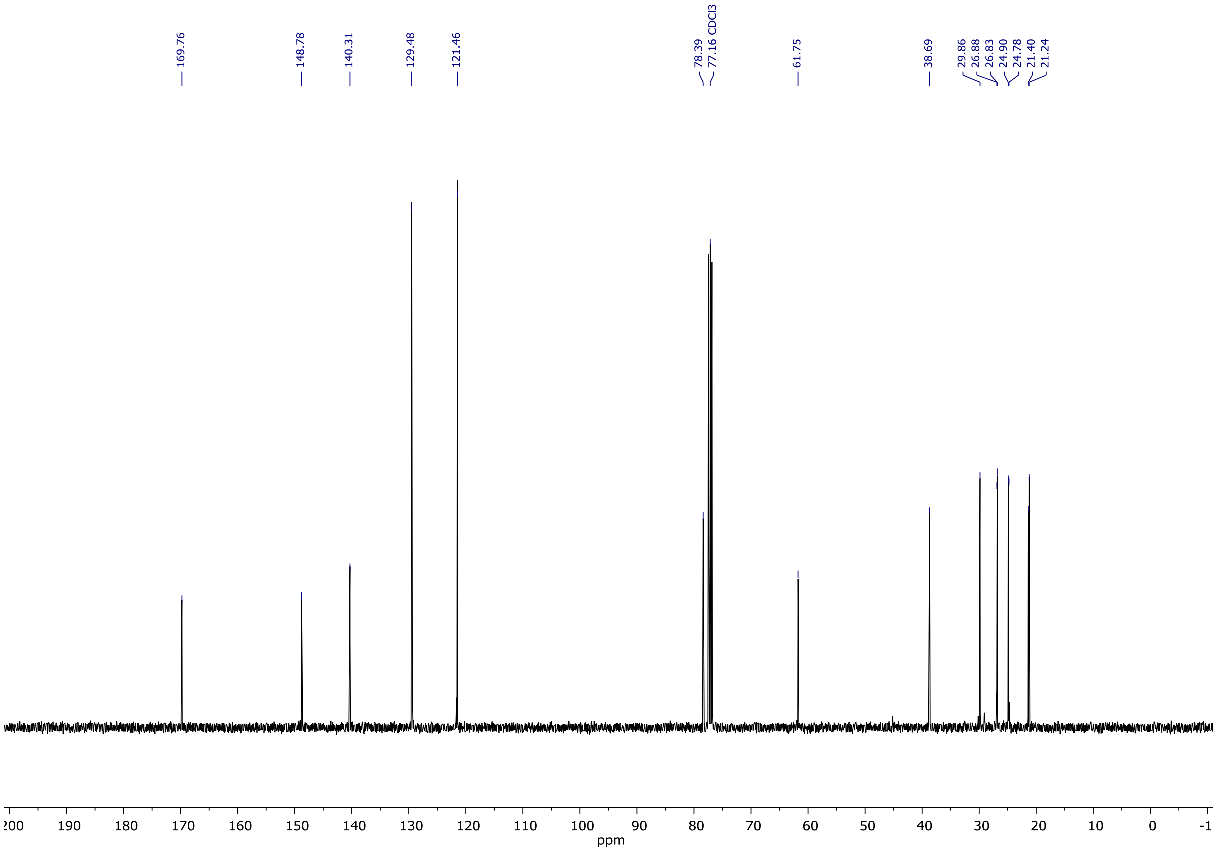

Compound **3am**, ^{1}H- and ^{13}C-NMR ($CDCl_3$):

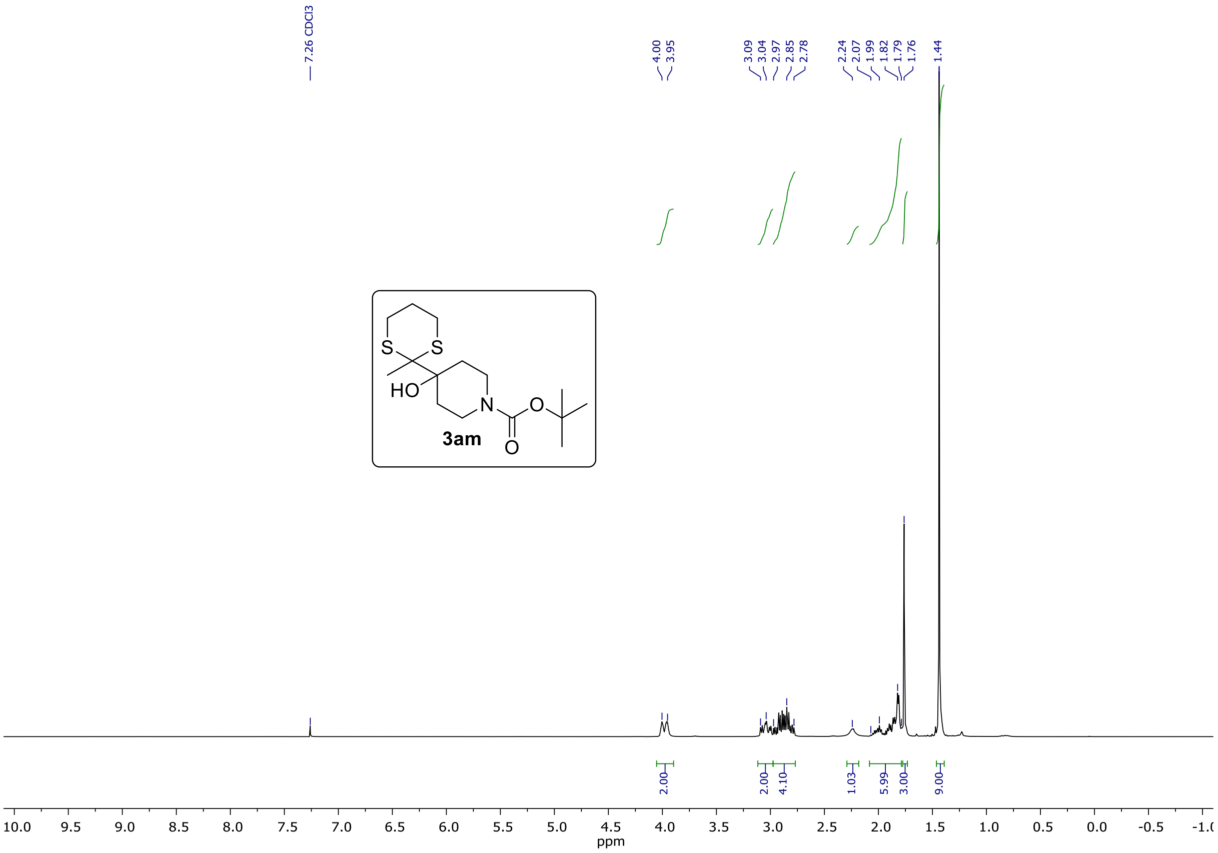

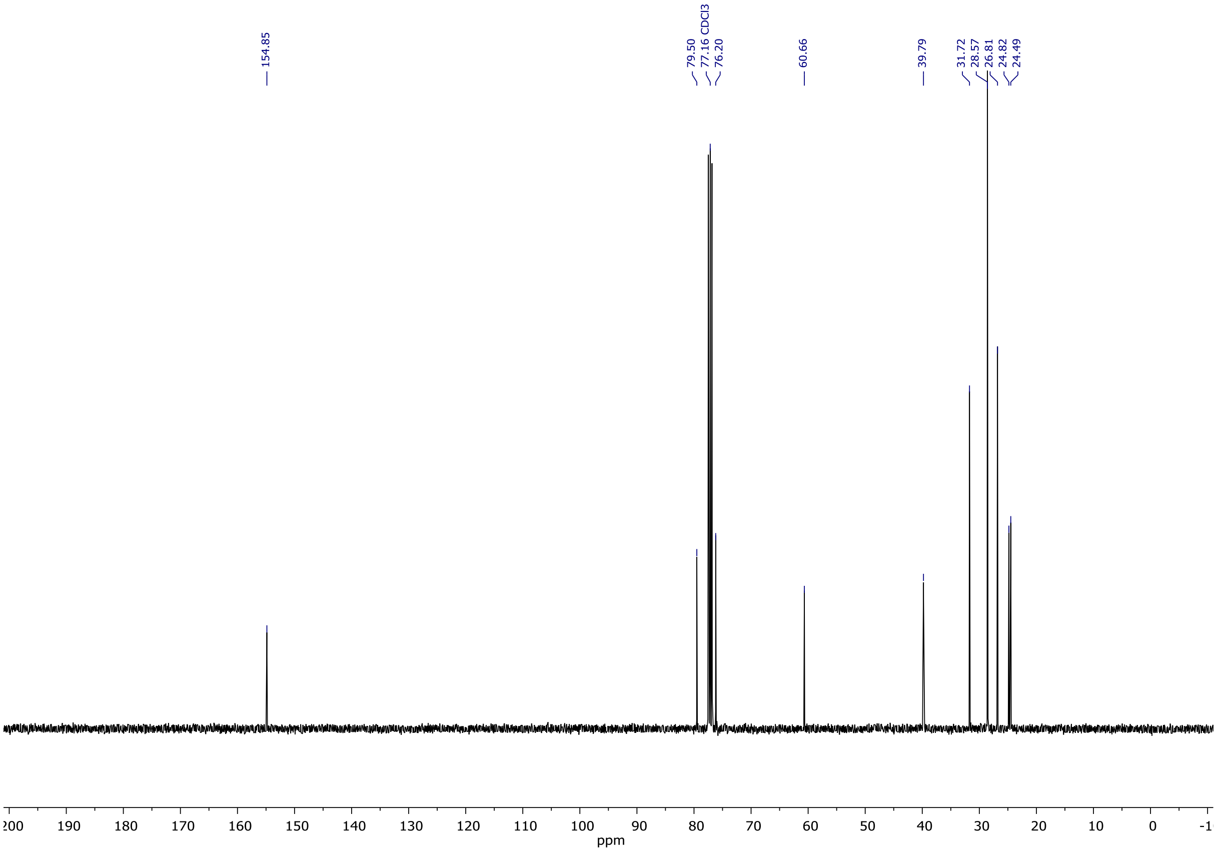

Compound **3an**, ^{1}H- and ^{13}C-NMR ($CDCl_3$):

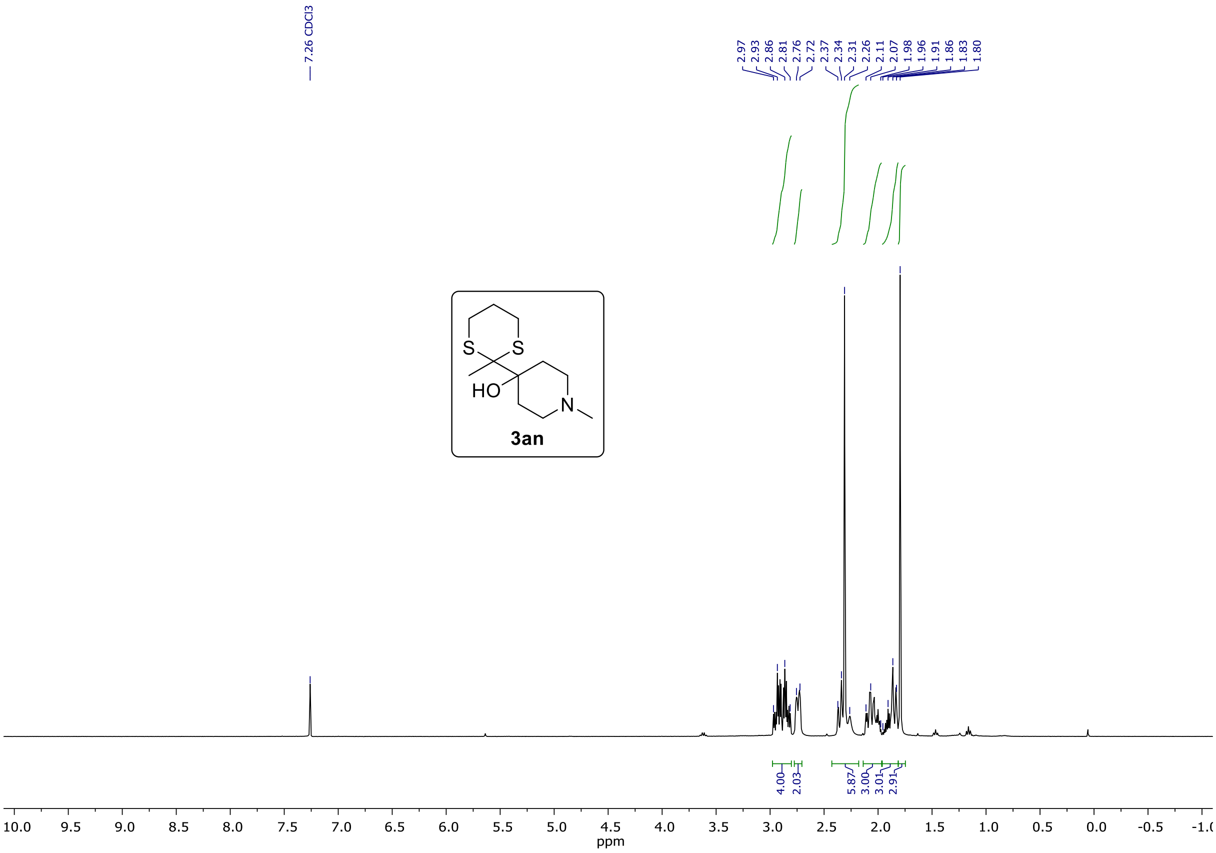

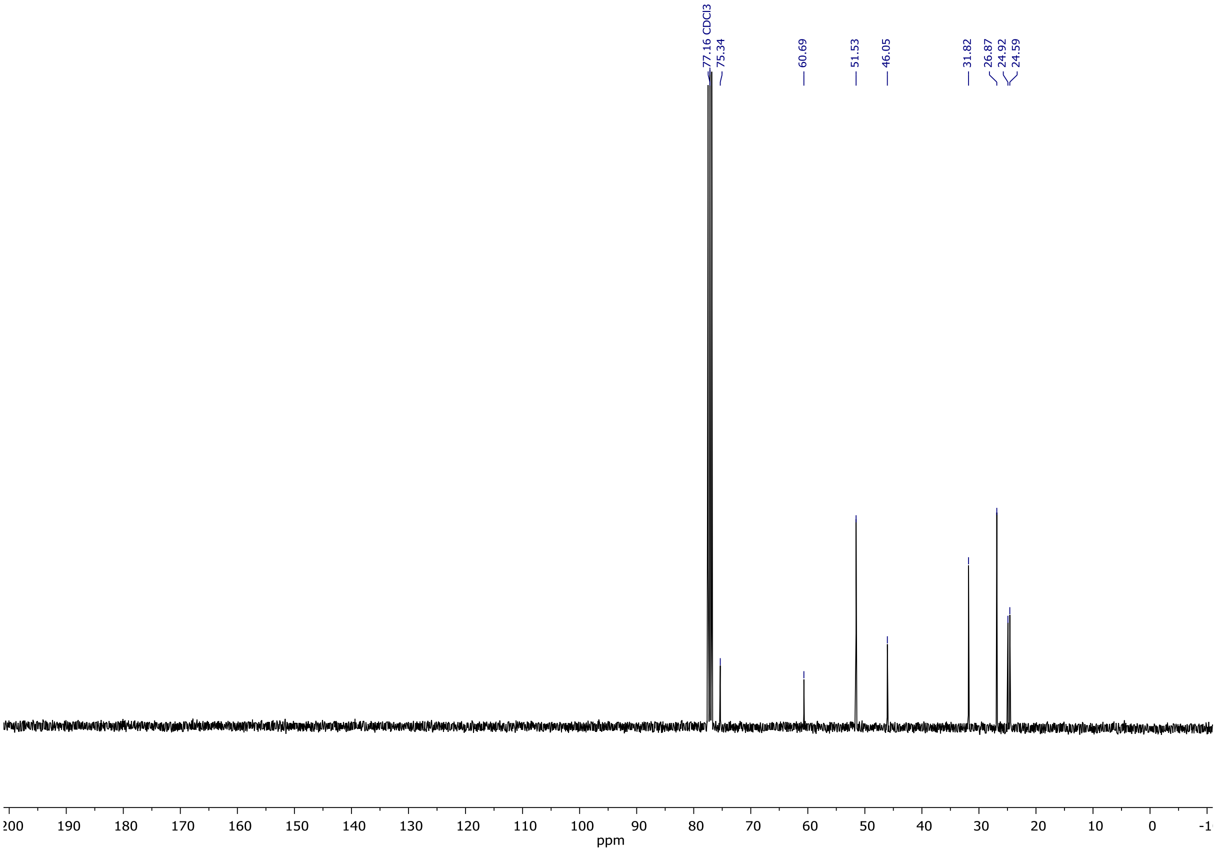

Compound **3ao**, ^{1}H-NMR (C_6D_6) and ^{13}C-NMR ($CDCl_3$):

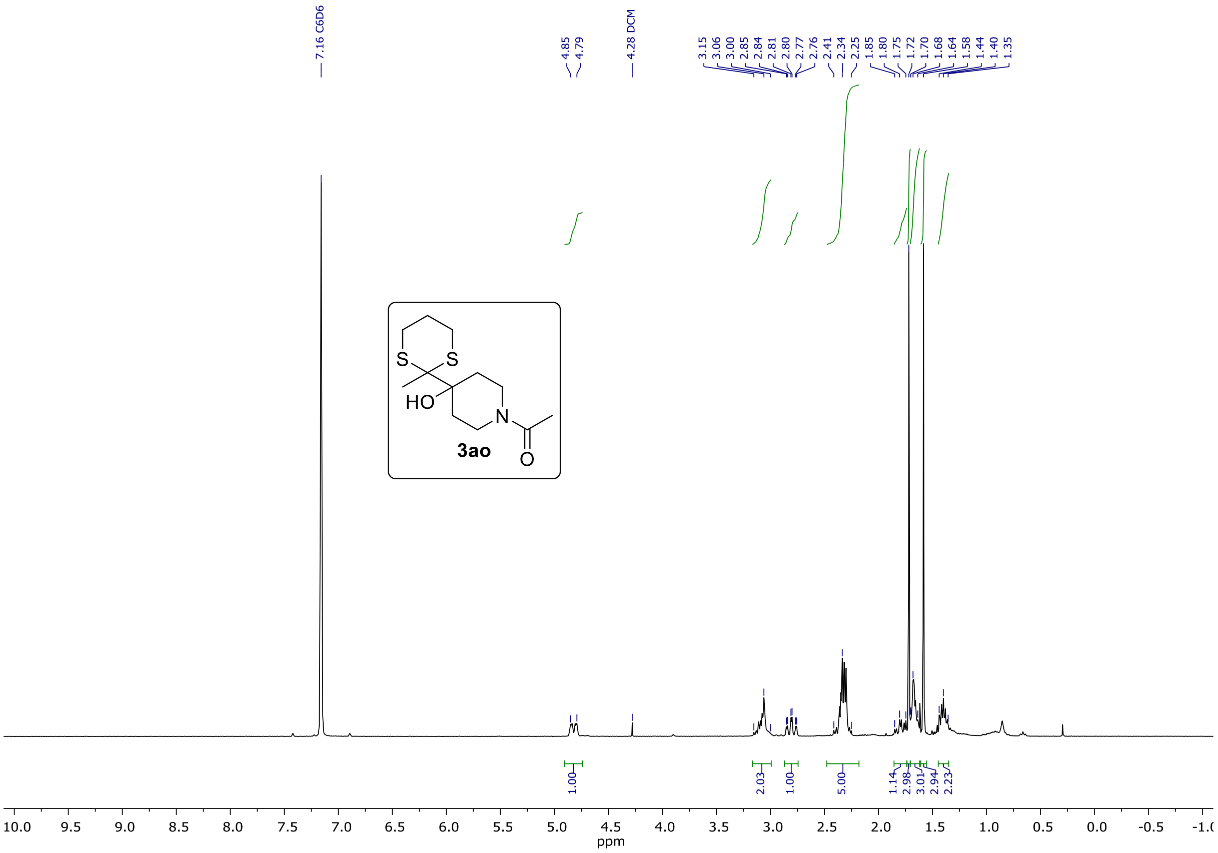

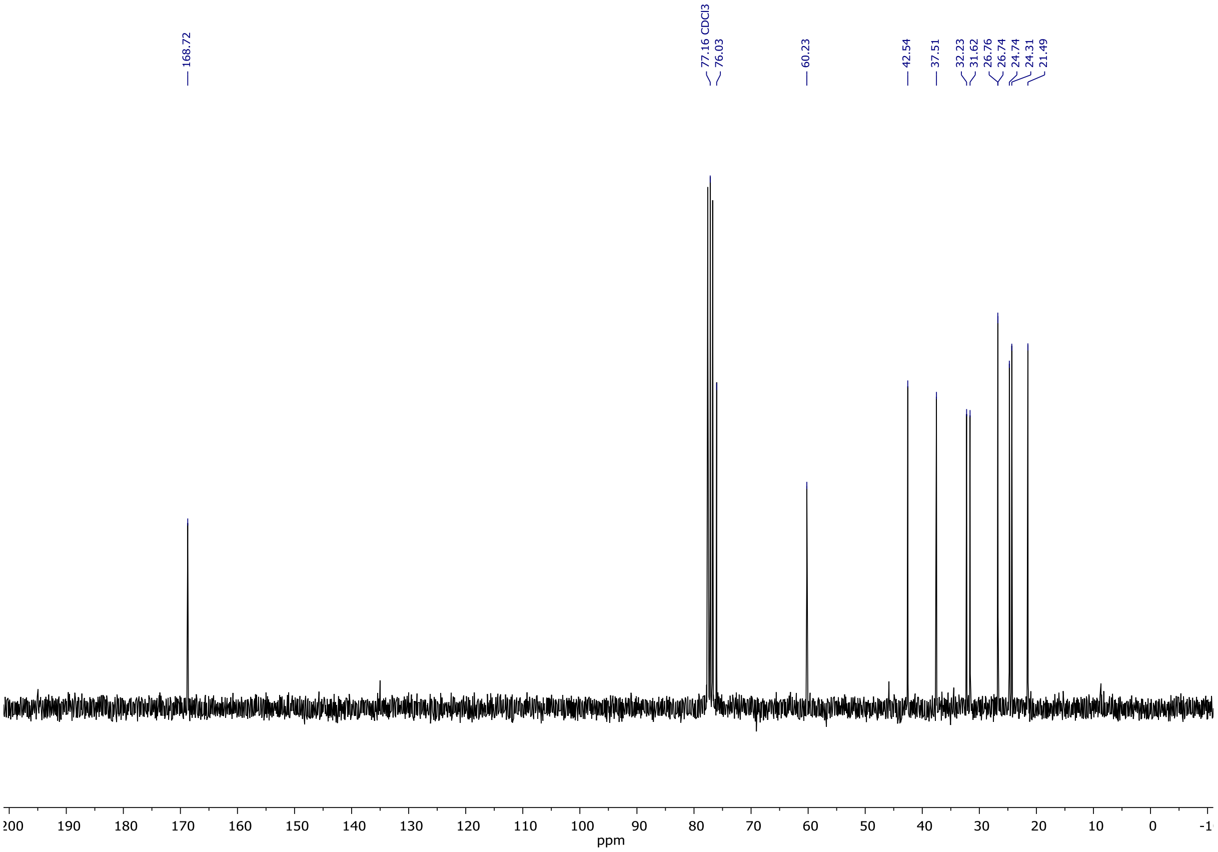

Compound *syn*-**3ap**, ^{1}H-NMR, ^{19}F-NMR (C_6D_6) and ^{13}C-NMR ($CDCl_3$):

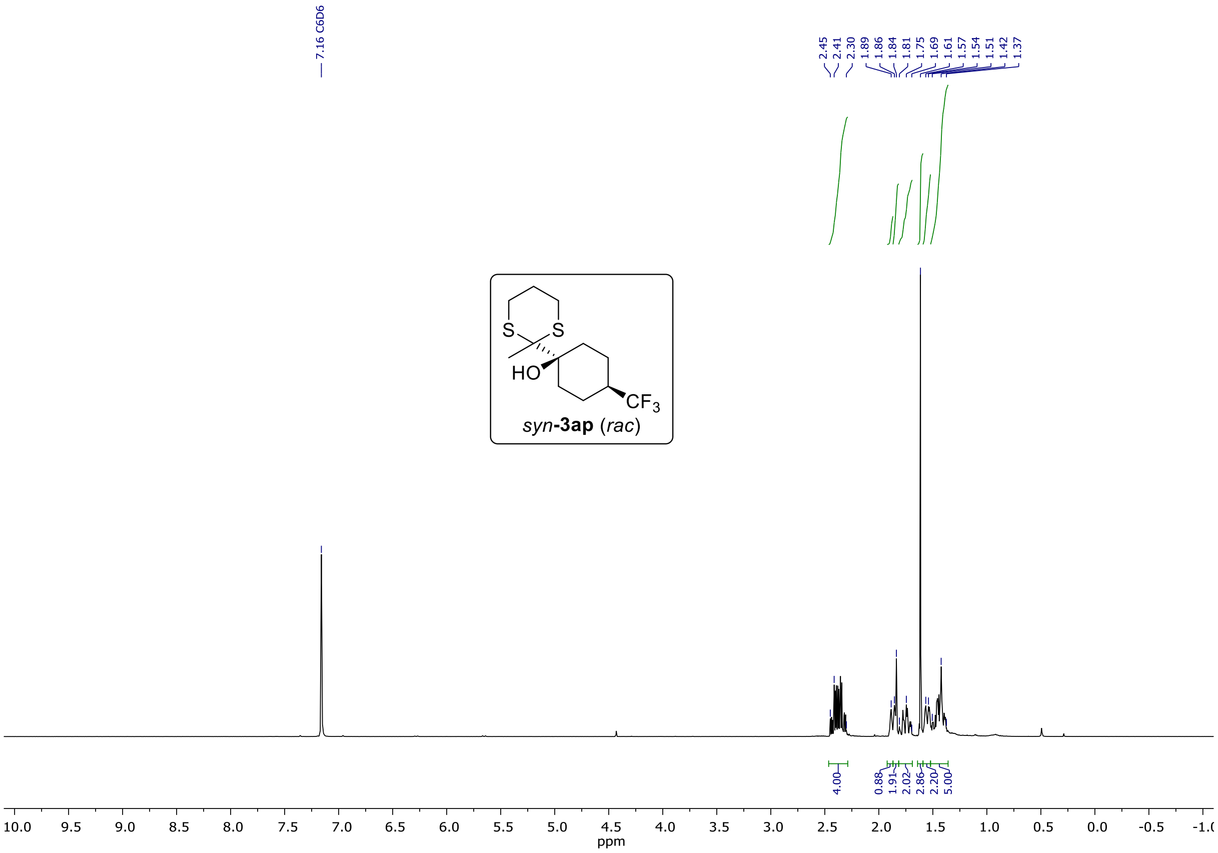

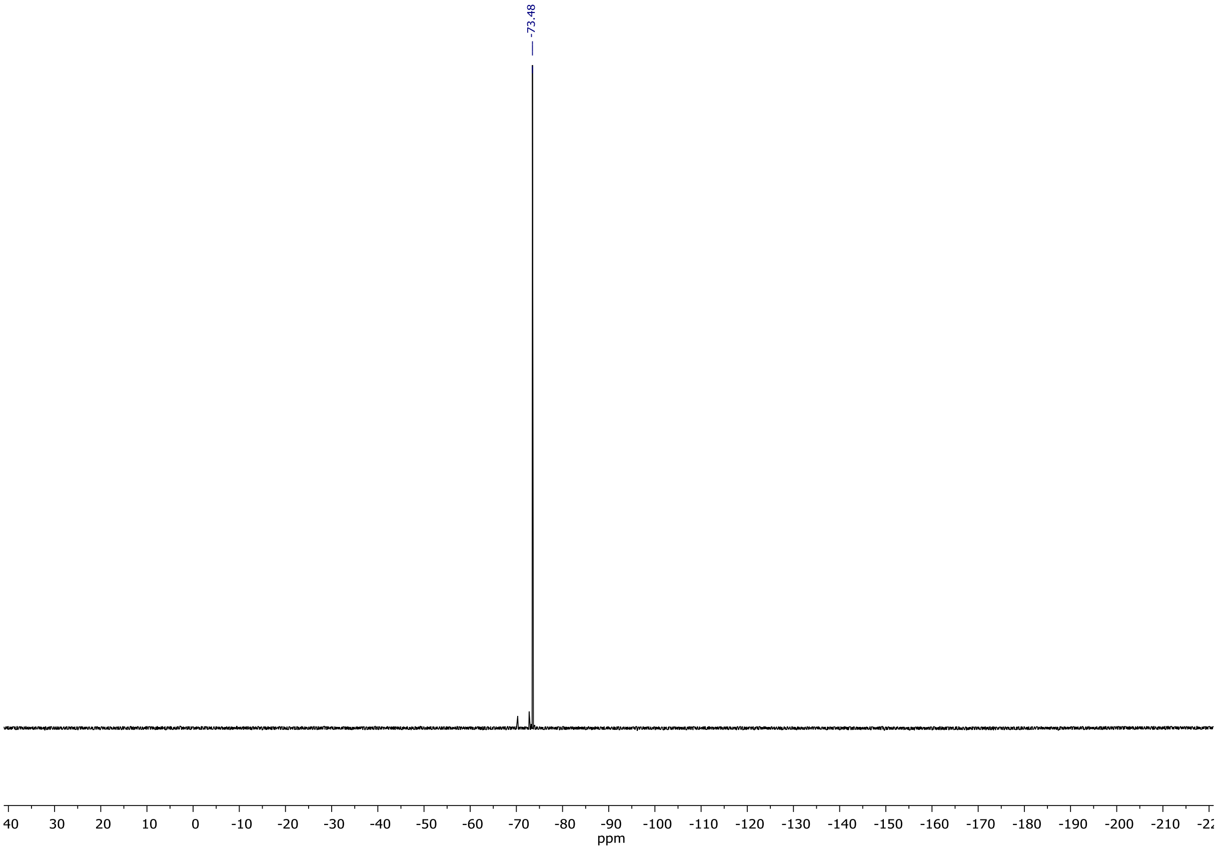

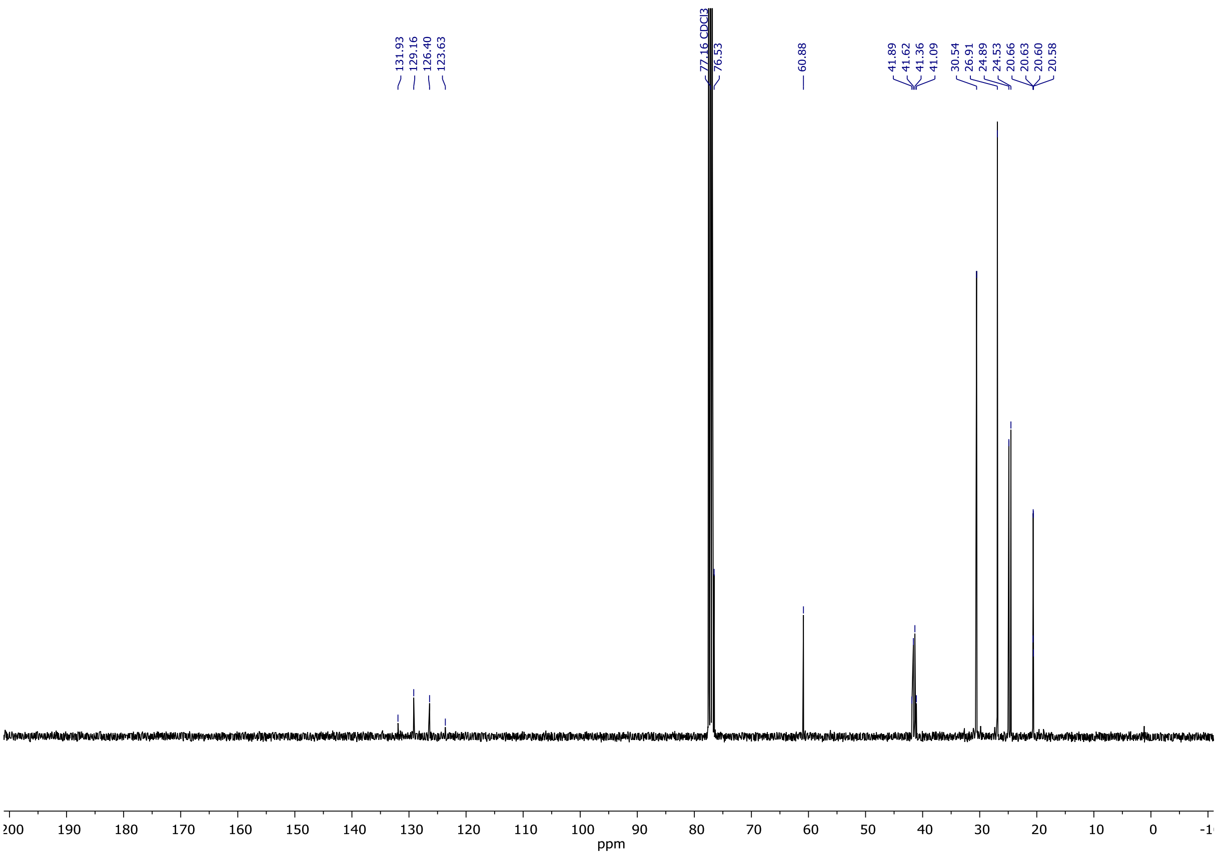

Compound *anti*-**3ap**, ^{1}H-NMR, ^{19}F-NMR (C_6D_6) and ^{13}C-NMR ($CDCl_3$):

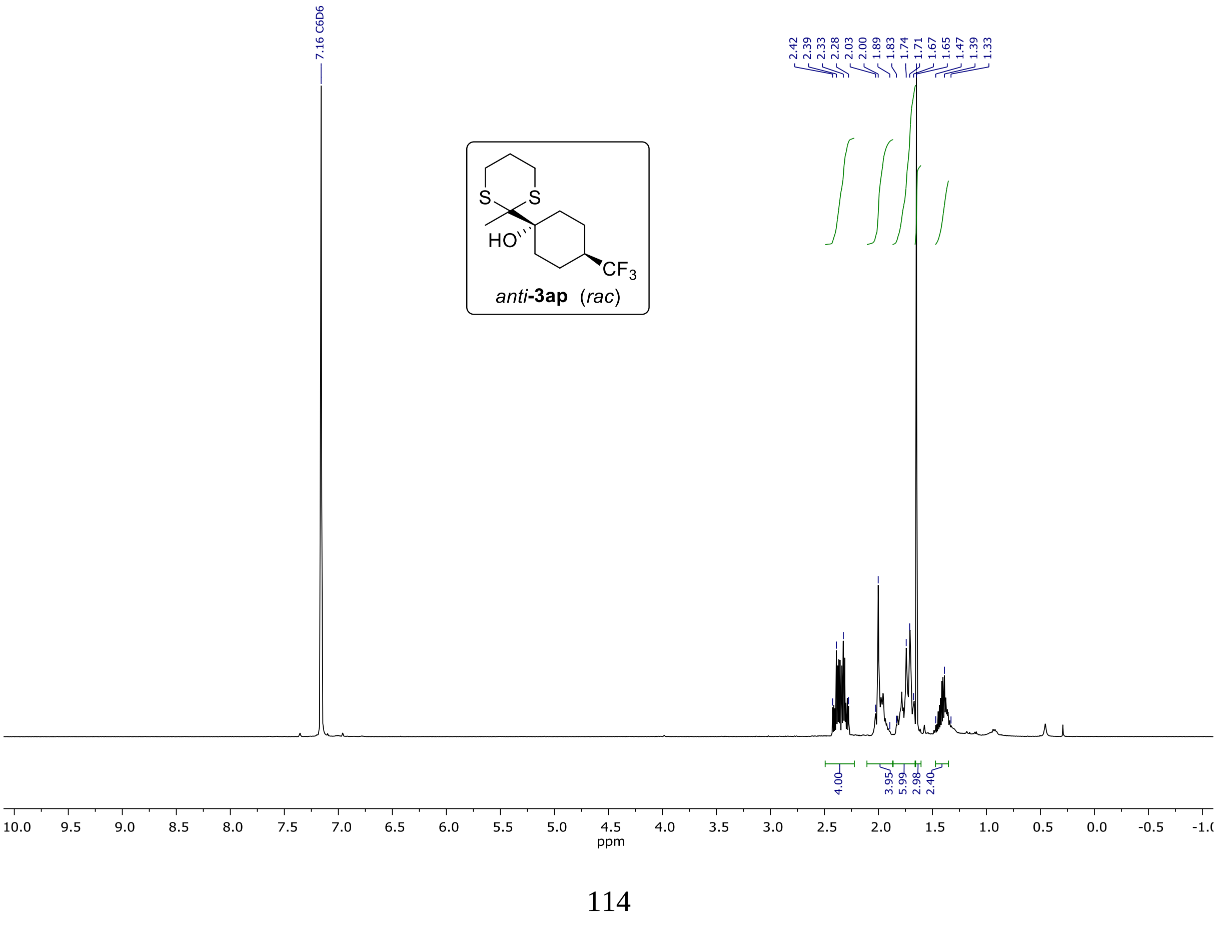

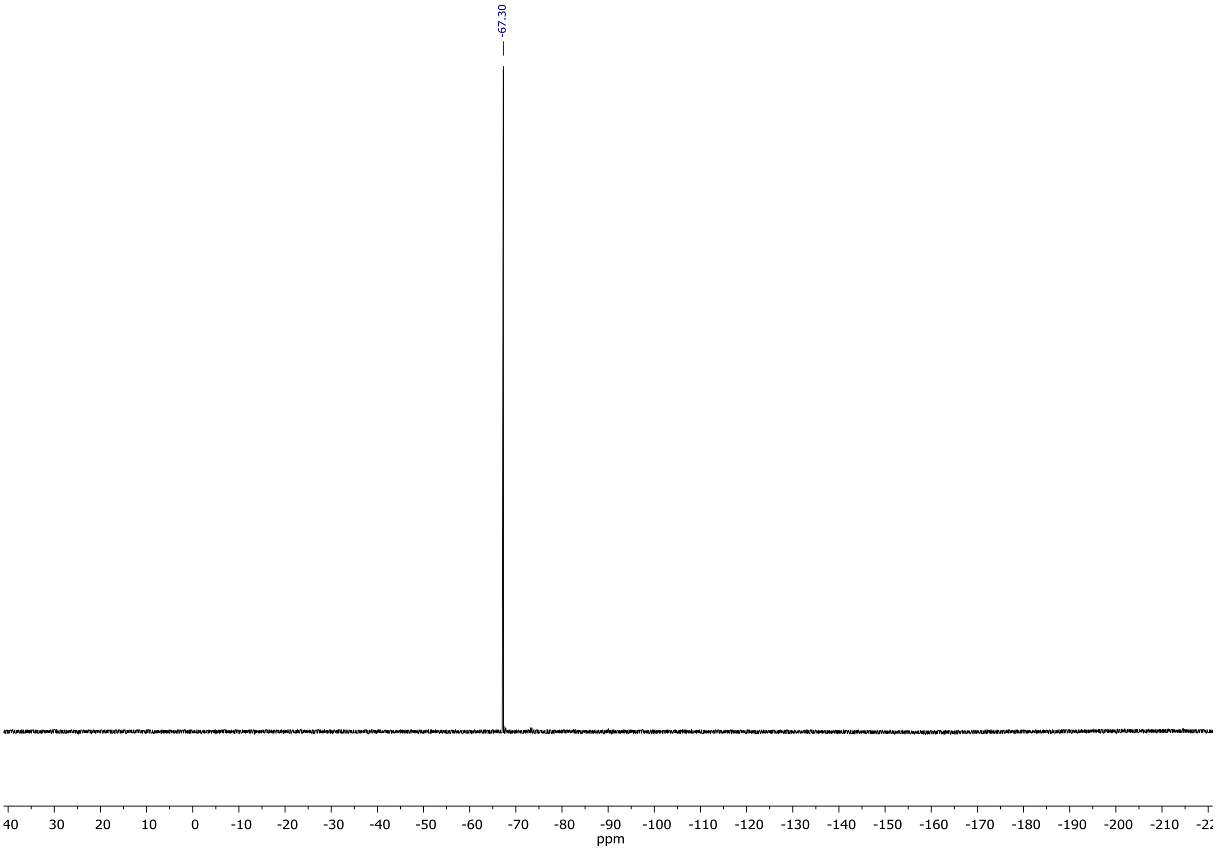
-67.30
40 30 20 10 0 -10 -20 -30 -40 -50 -60 -70 -80 -90 -100 -110 -120 -130 -140 -150 -160 -170 -180 -190 -200 -210
ppm

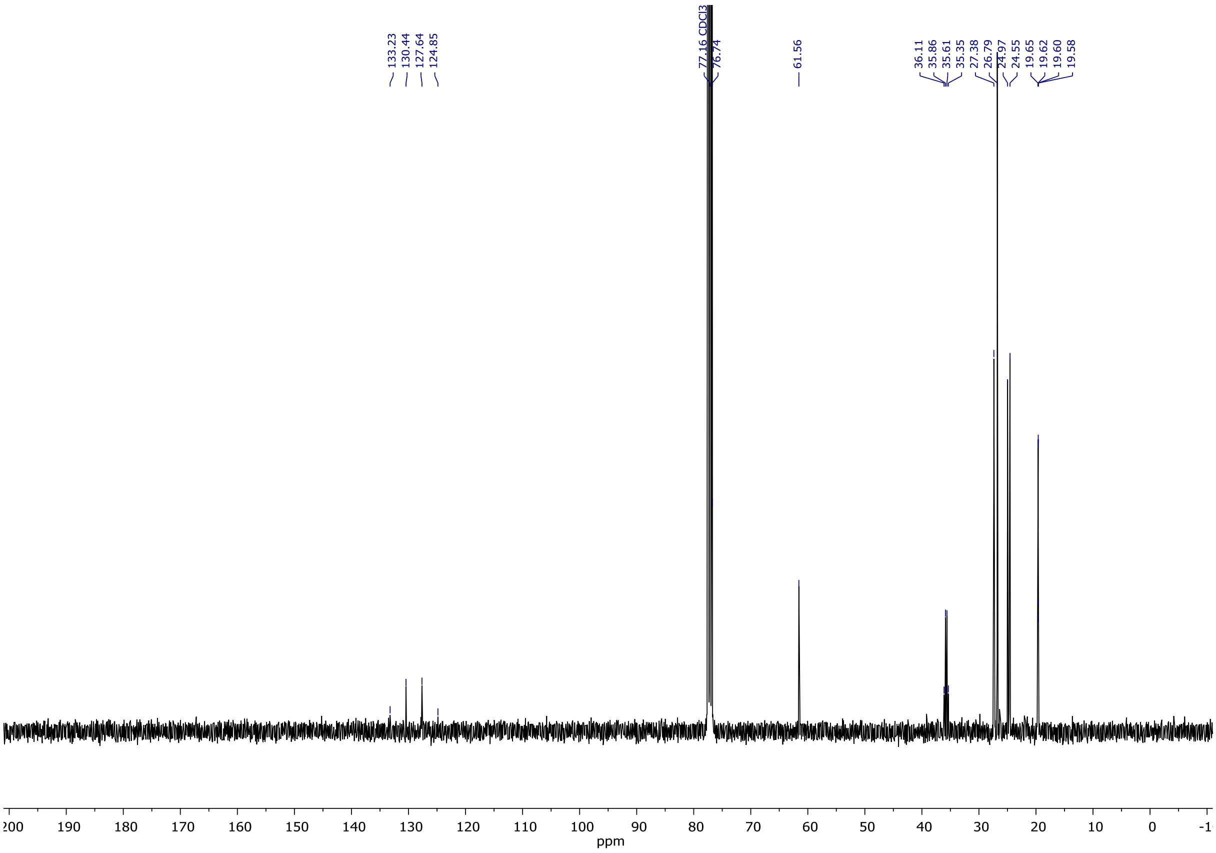
133.23
130.44
127.64
124.85
77.16 CDCl3
76.74
61.56
36.11
35.86
35.61
35.35
27.38
26.79
24.97
24.55
19.65
19.62
19.60
19.58
190 180 170 160 150 140 130 120 110 100 90 80 70 60 50 40 30 20 10 0
ppm

Compound **3aq**, ^{1}H- and ^{13}C-NMR ($CDCl_3$):

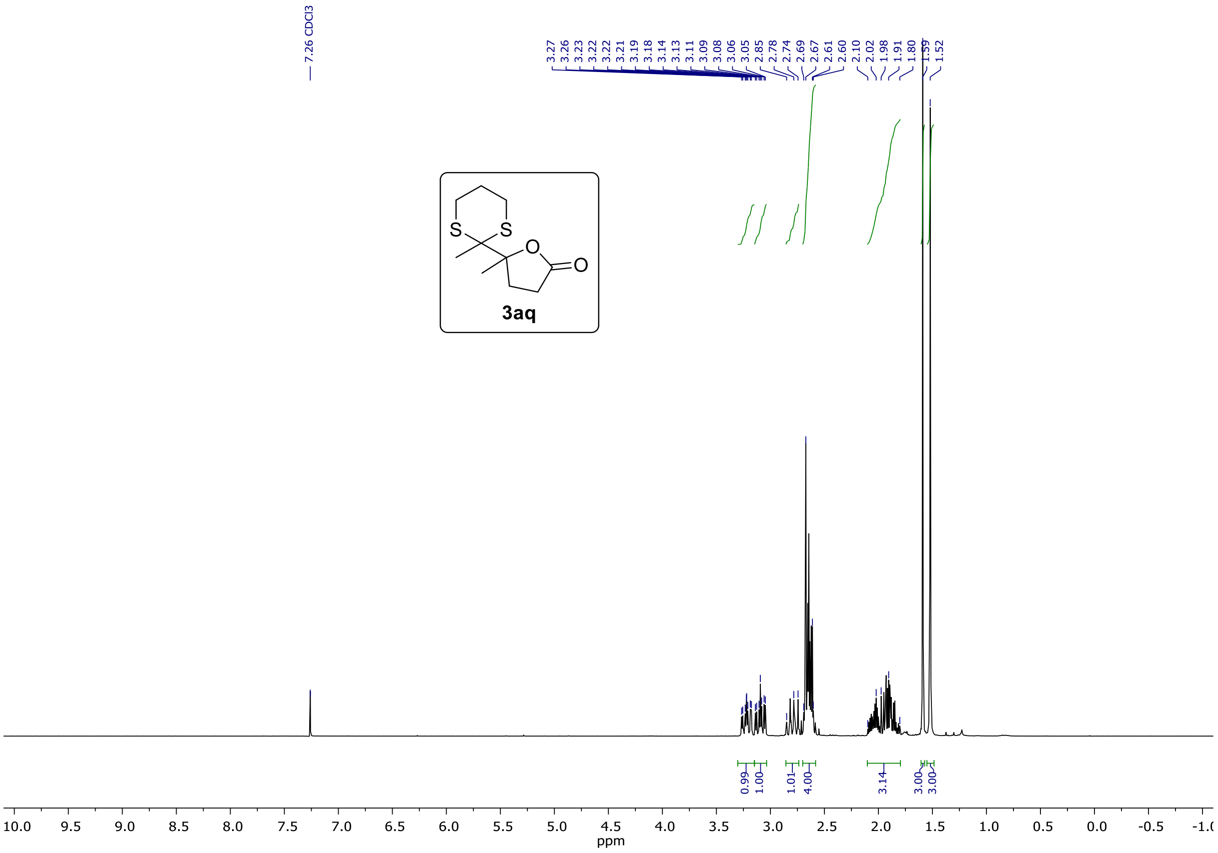

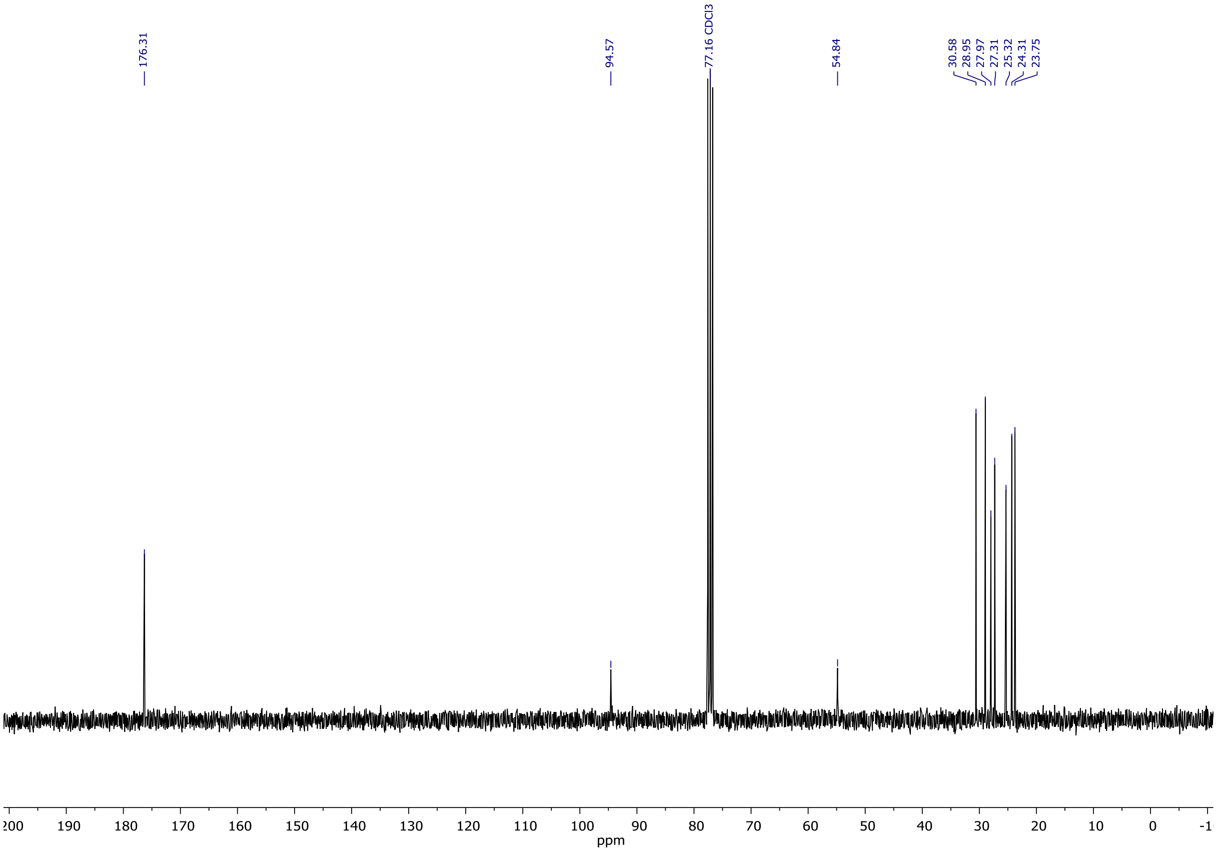

Compound **3ar**, ^{1}H-NMR and ^{13}C-NMR ($CDCl_3$):

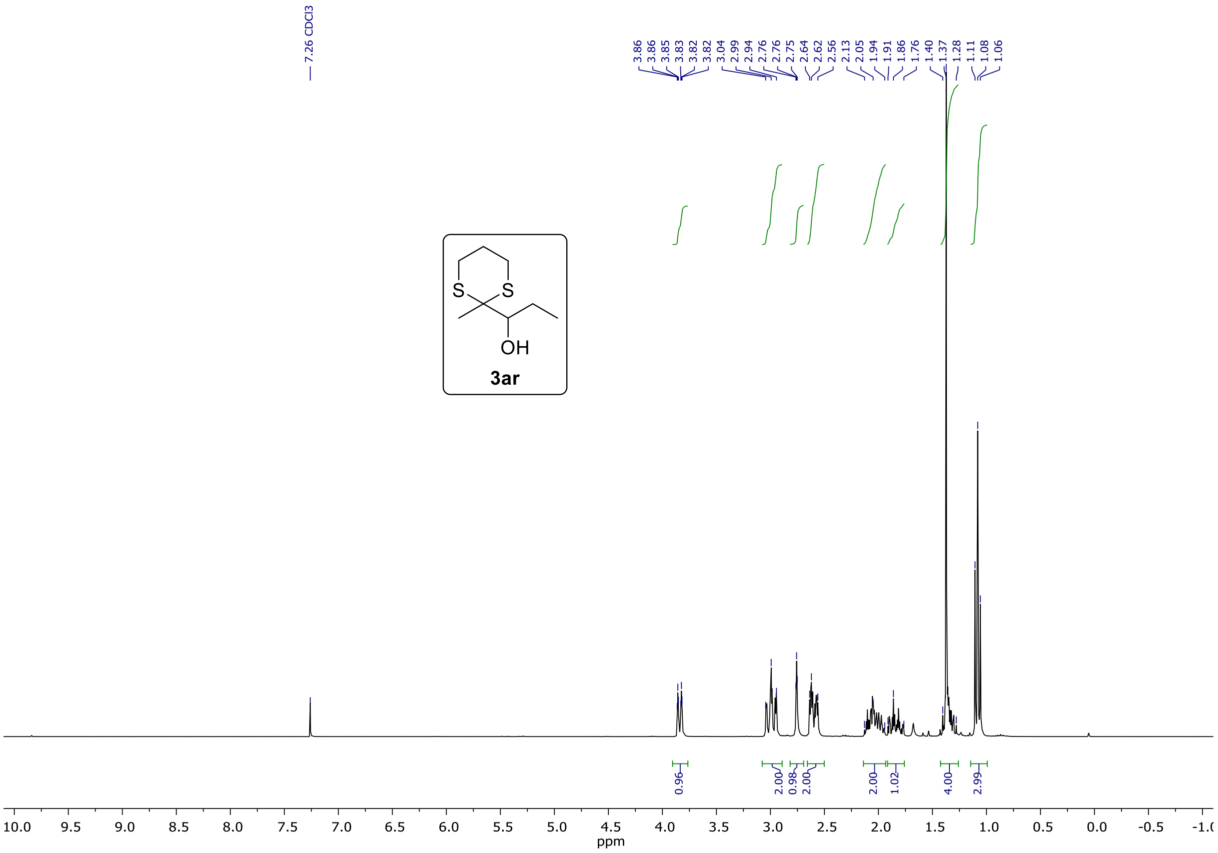

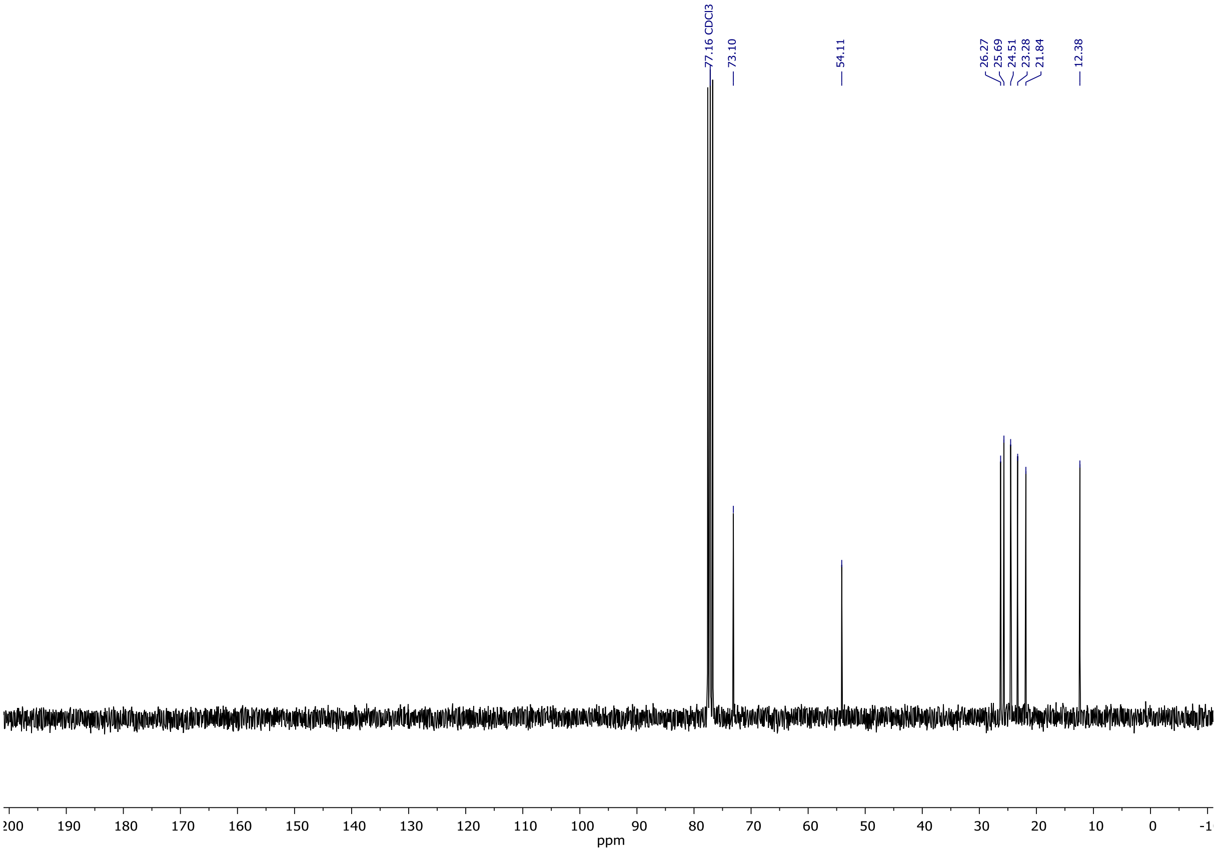

Compound **3as**, ^{1}H-NMR and ^{13}C-NMR ($CDCl_3$):

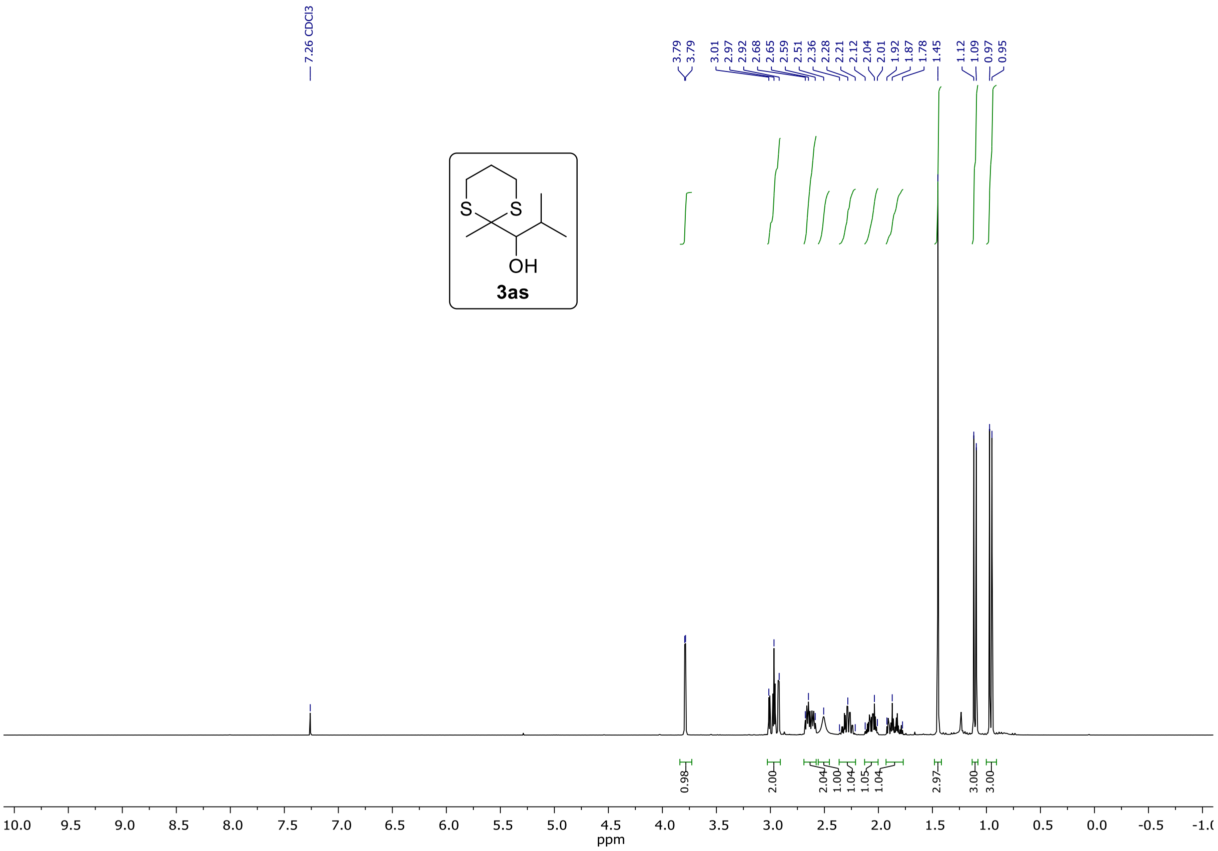

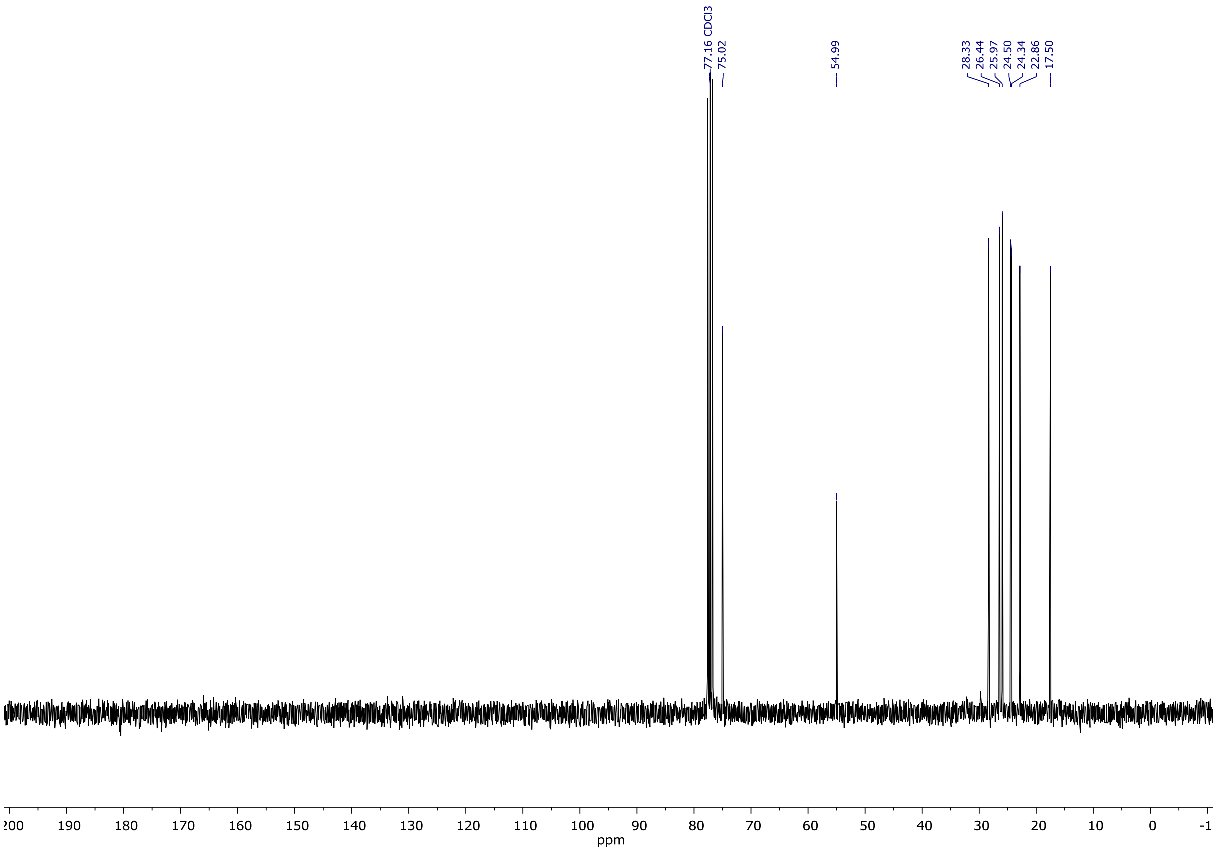

Compound **3at**, ^{1}H-NMR and ^{13}C-NMR ($CDCl_3$):

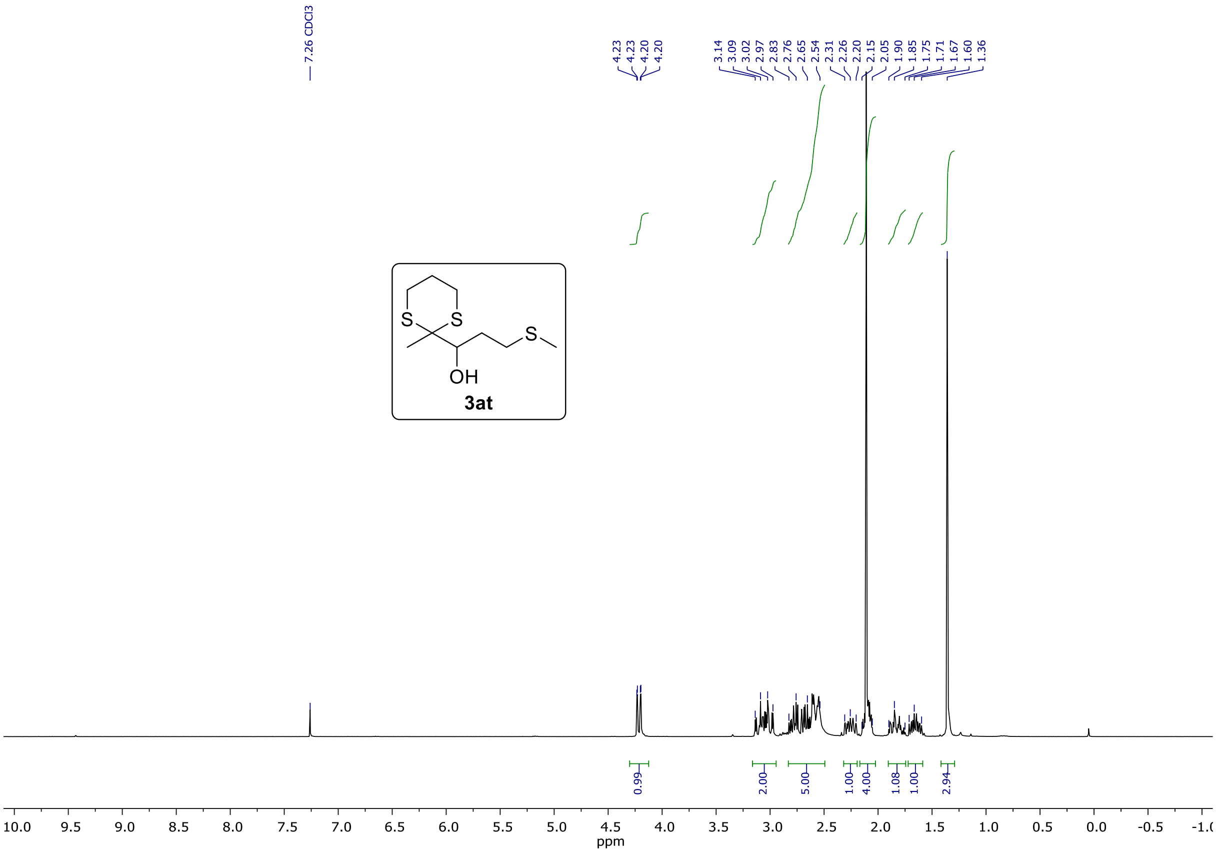

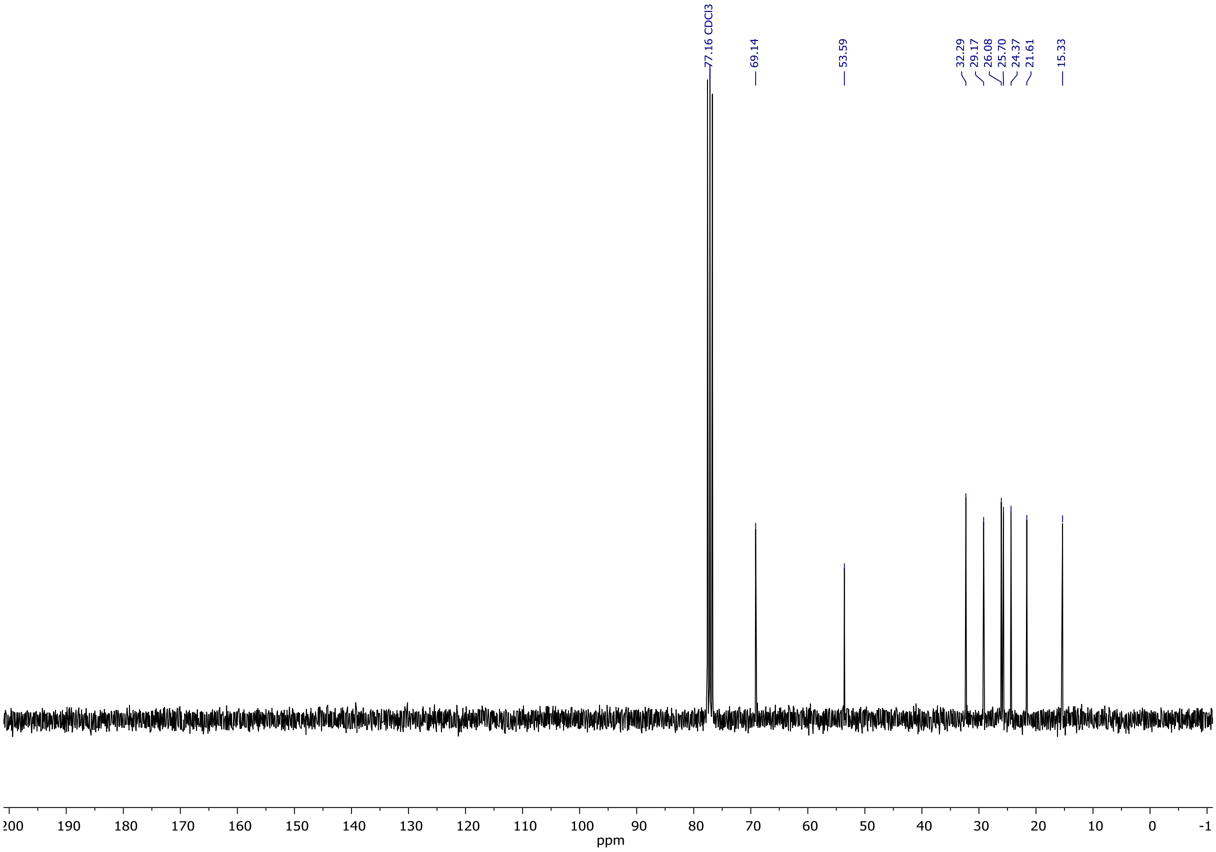

Compound **3au**, ^{1}H-NMR and ^{13}C-NMR ($CDCl_3$):

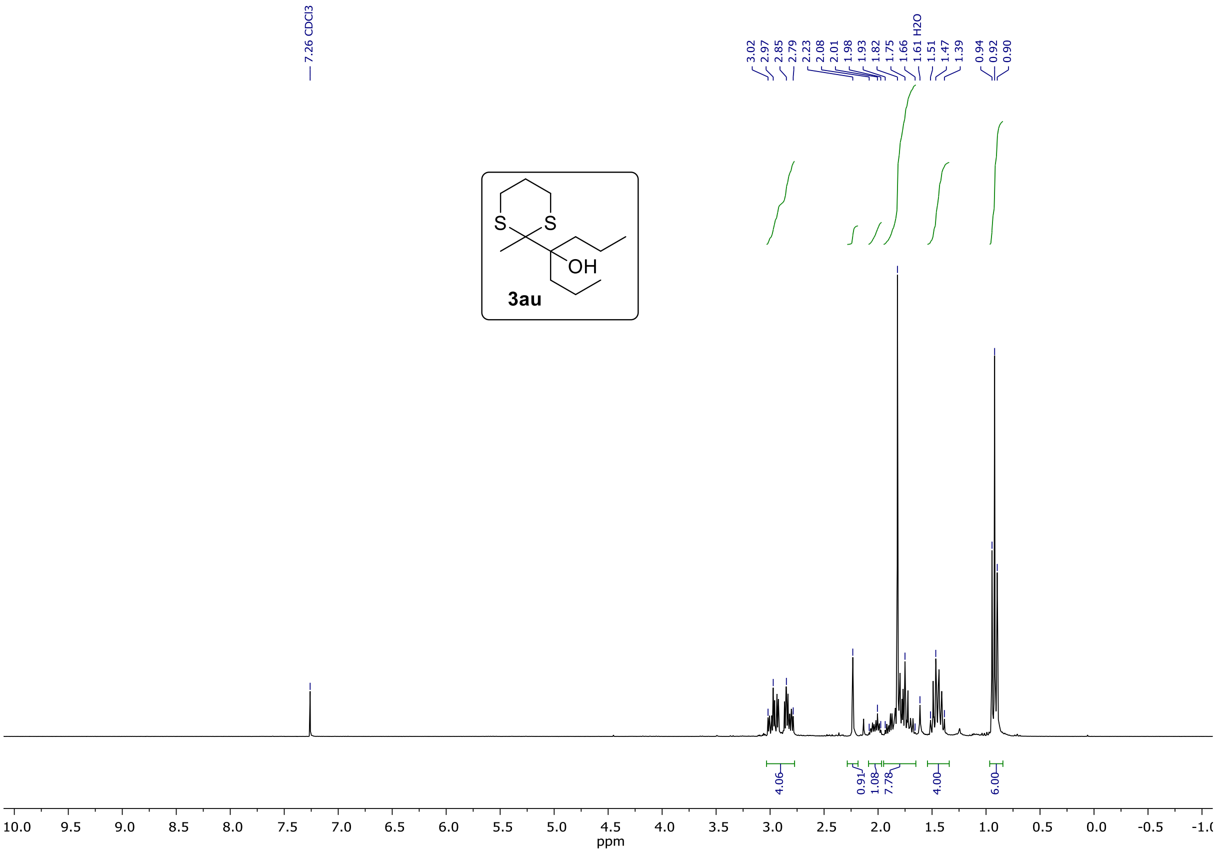

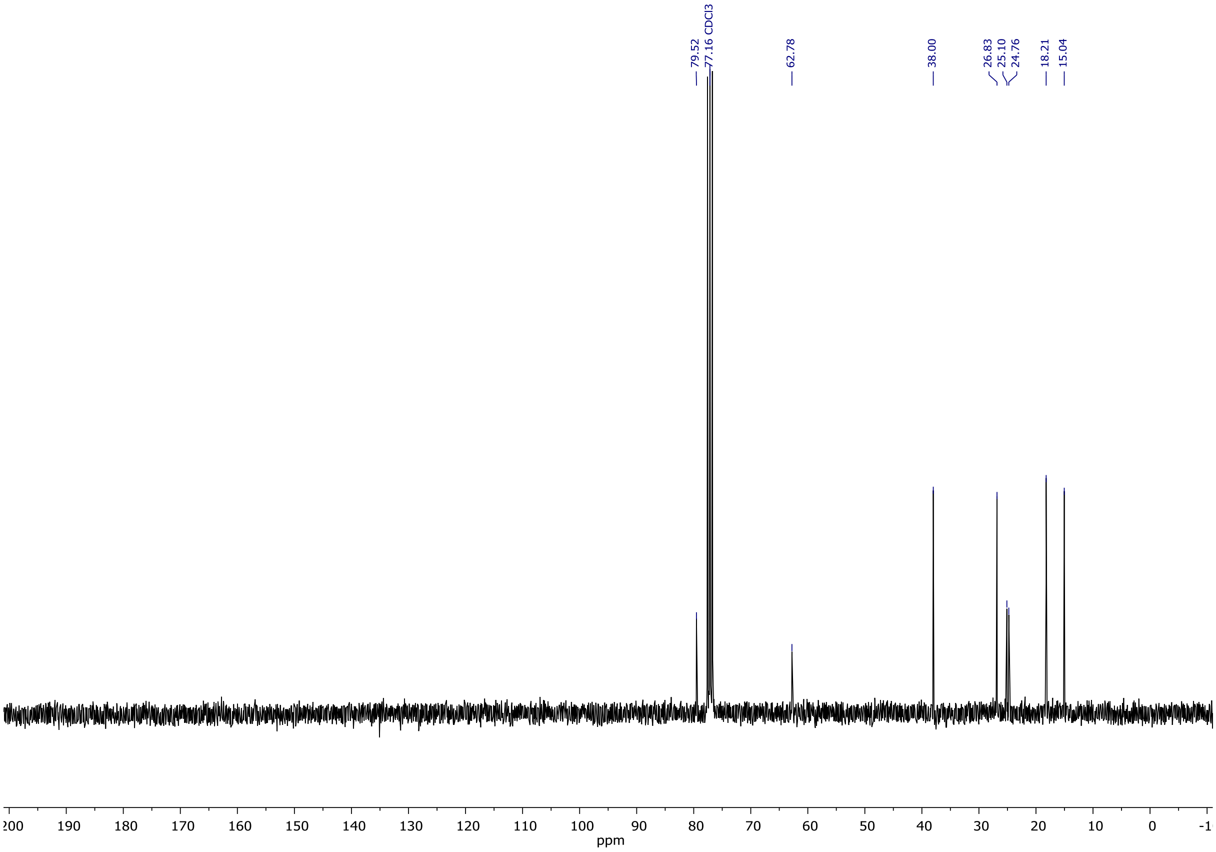

Compound **3av**, ^{1}H-NMR (C_6D_6) and ^{13}C-NMR ($CDCl_3$):

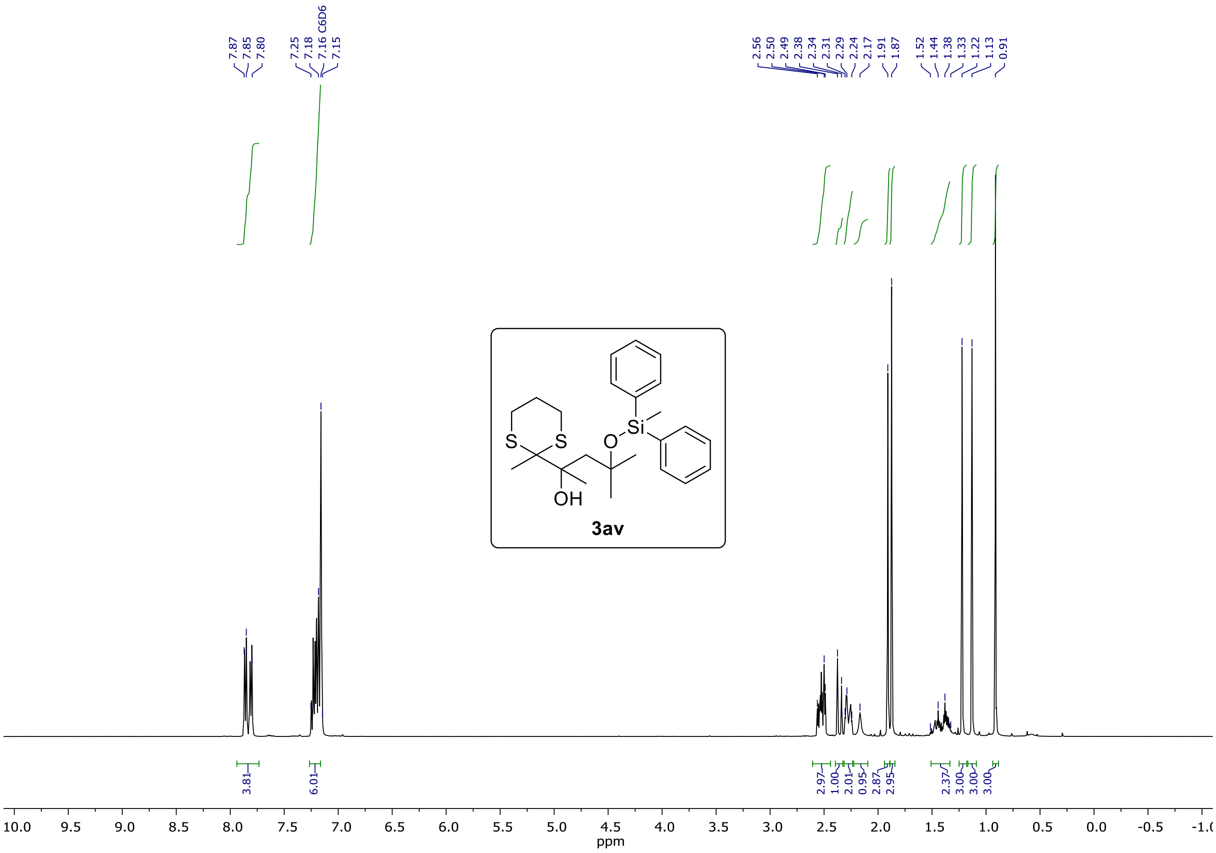

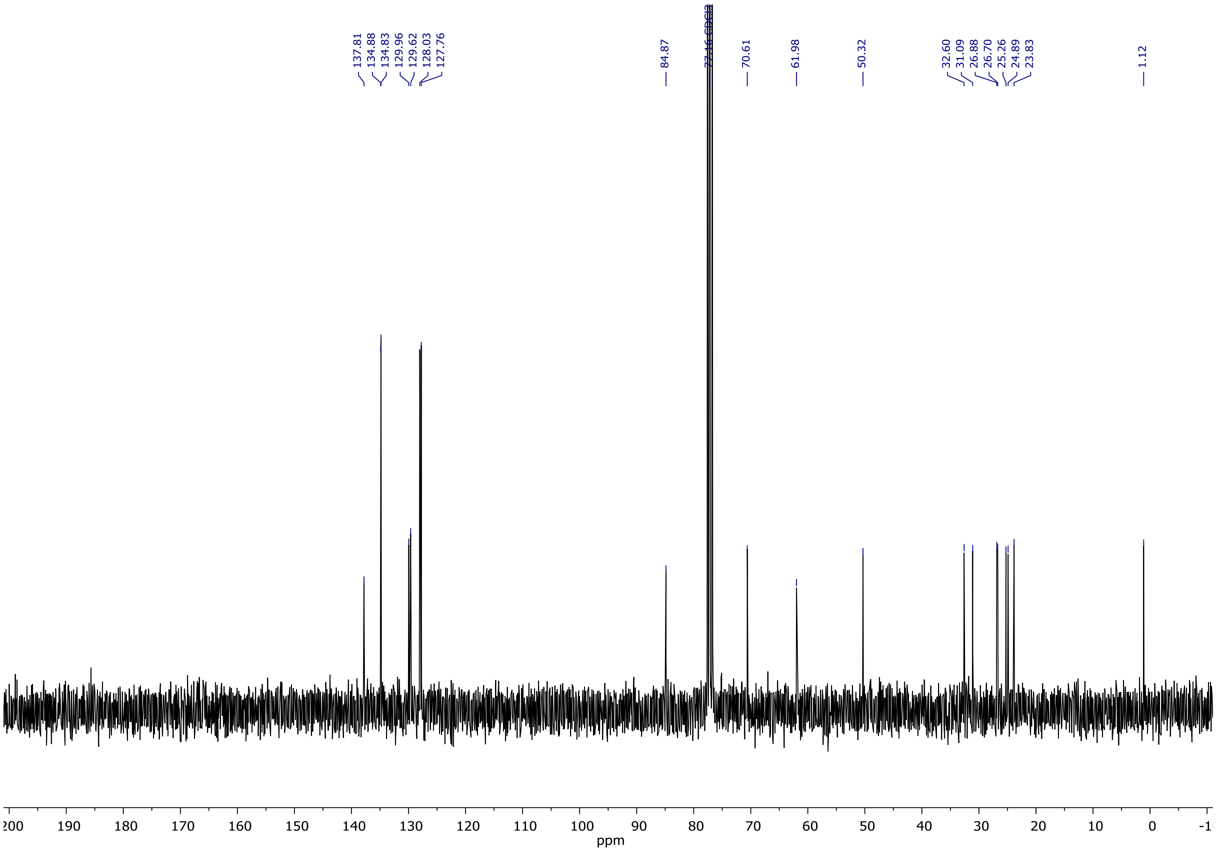

Compound **3aw**, ^{1}H-NMR (CD_2Cl_2) and ^{13}C-NMR ($CDCl_3$):

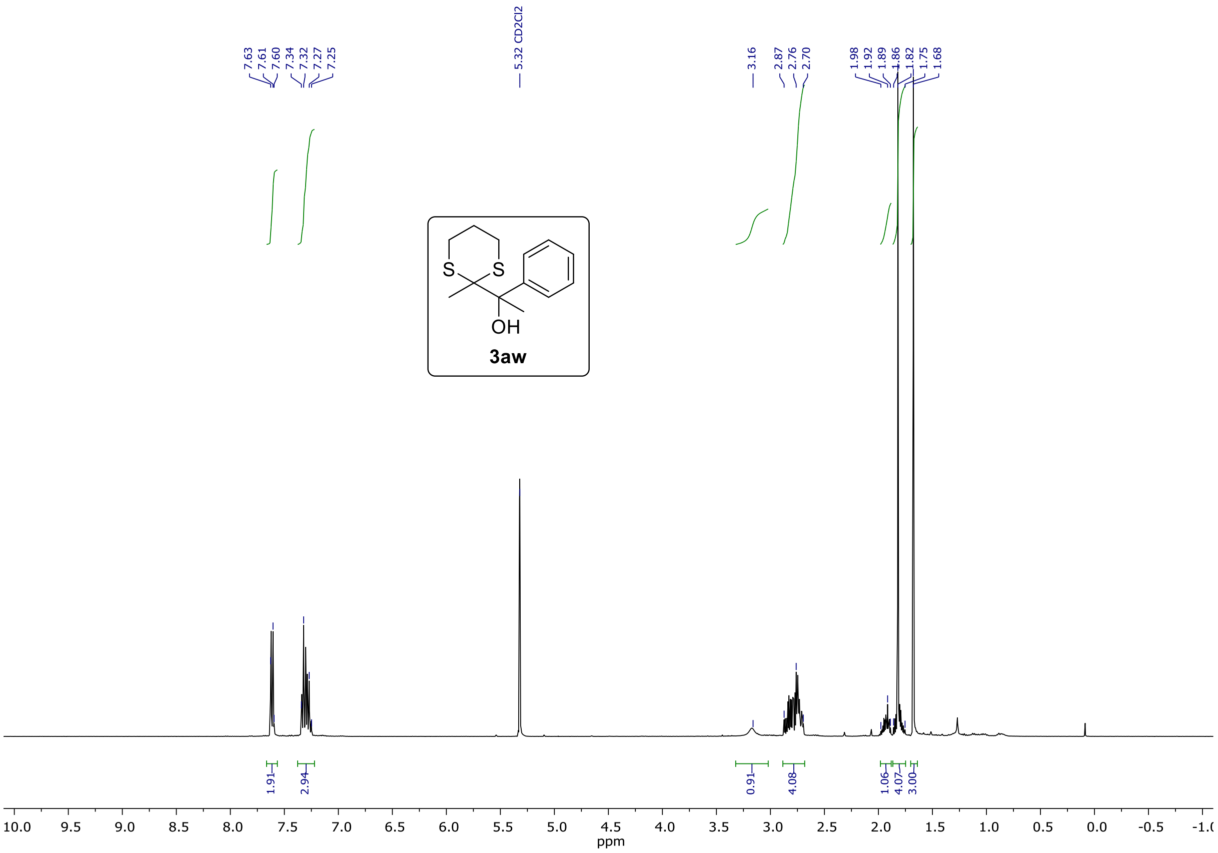

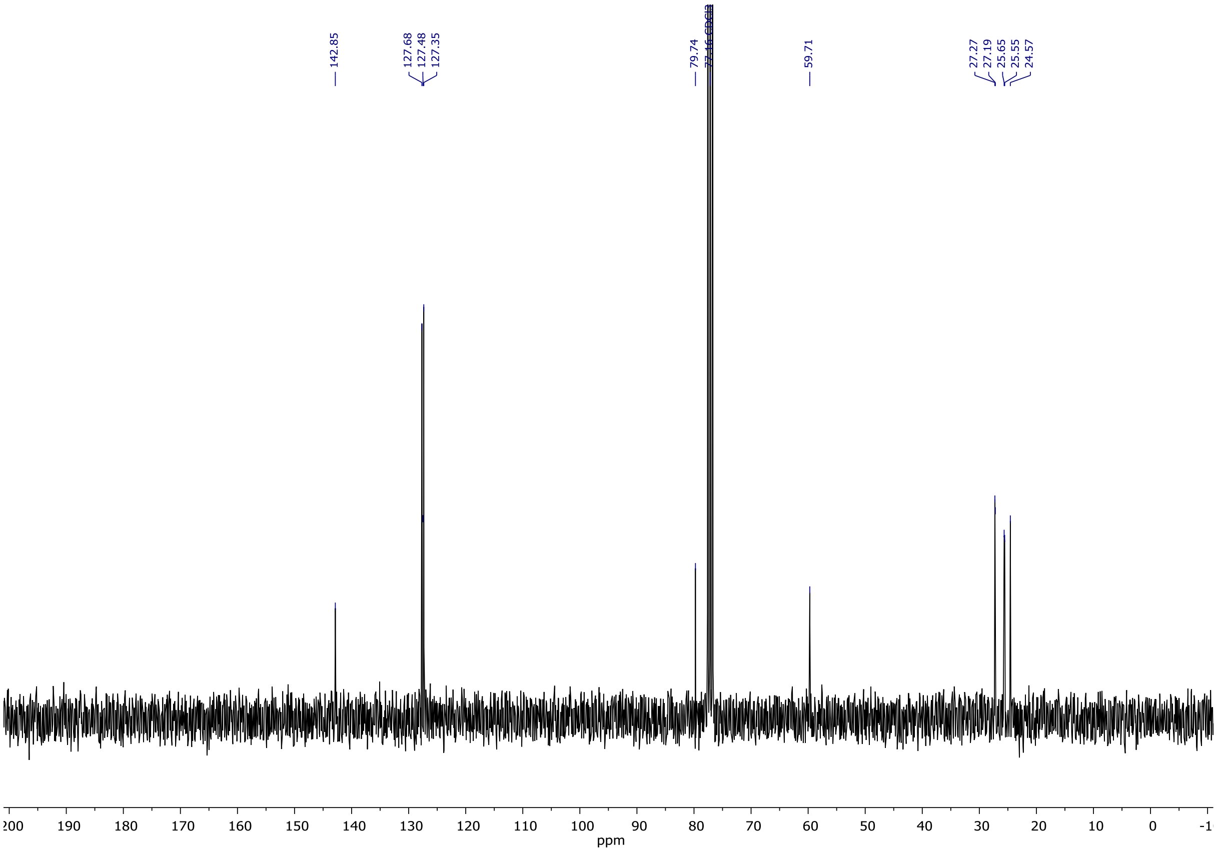

Compound **3ax**, ^{1}H-NMR and ^{13}C-NMR ($CDCl_3$):

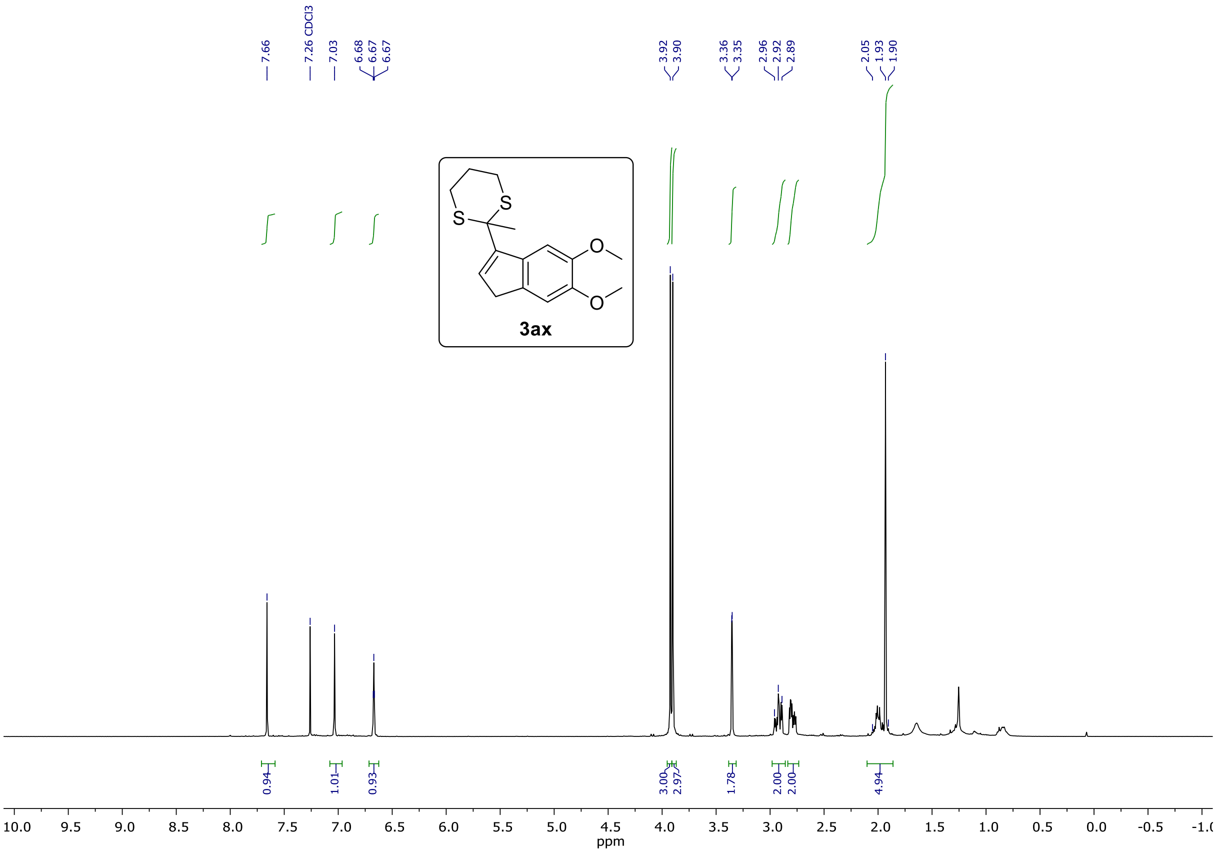

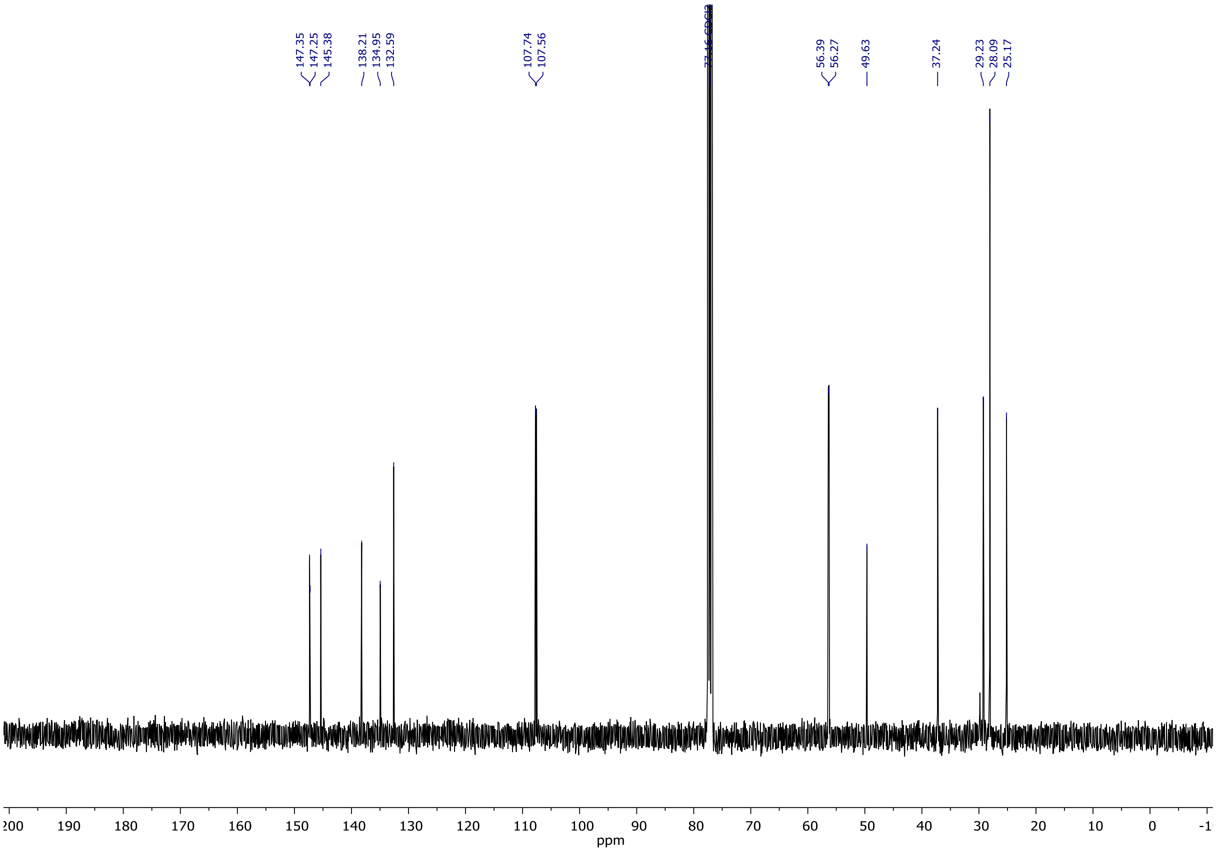

Compound **3kr**, ^{1}H-NMR and ^{13}C-NMR ($CDCl_3$):

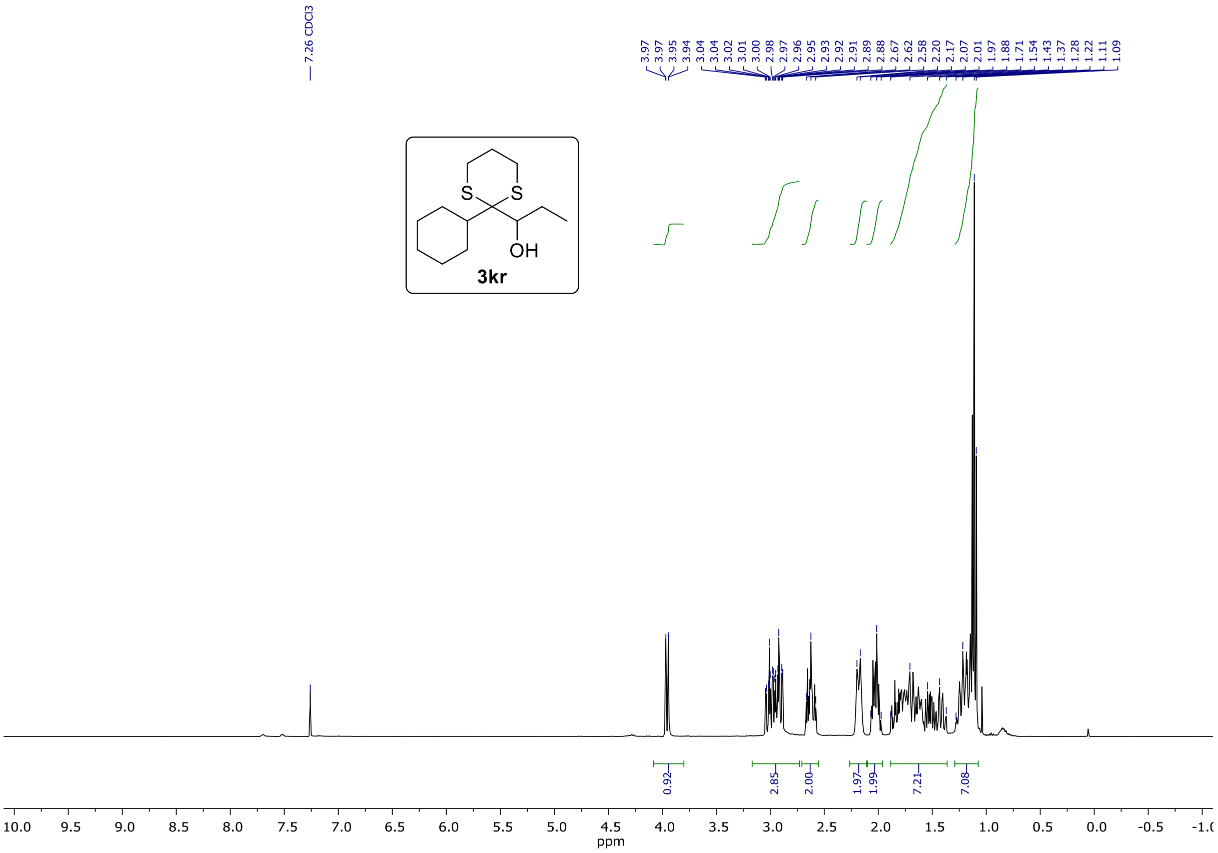

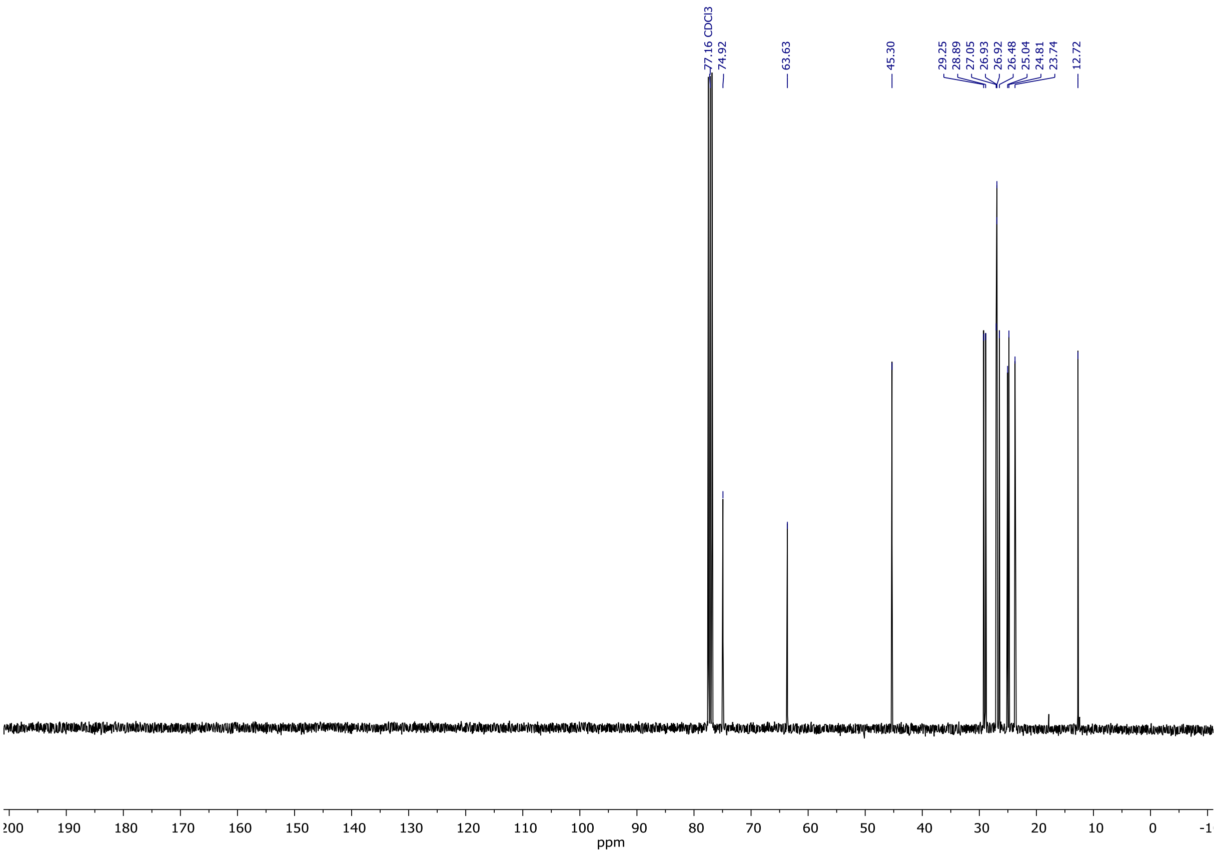

Compound **3lr**, ^{1}H-NMR and ^{13}C-NMR ($CDCl_3$):

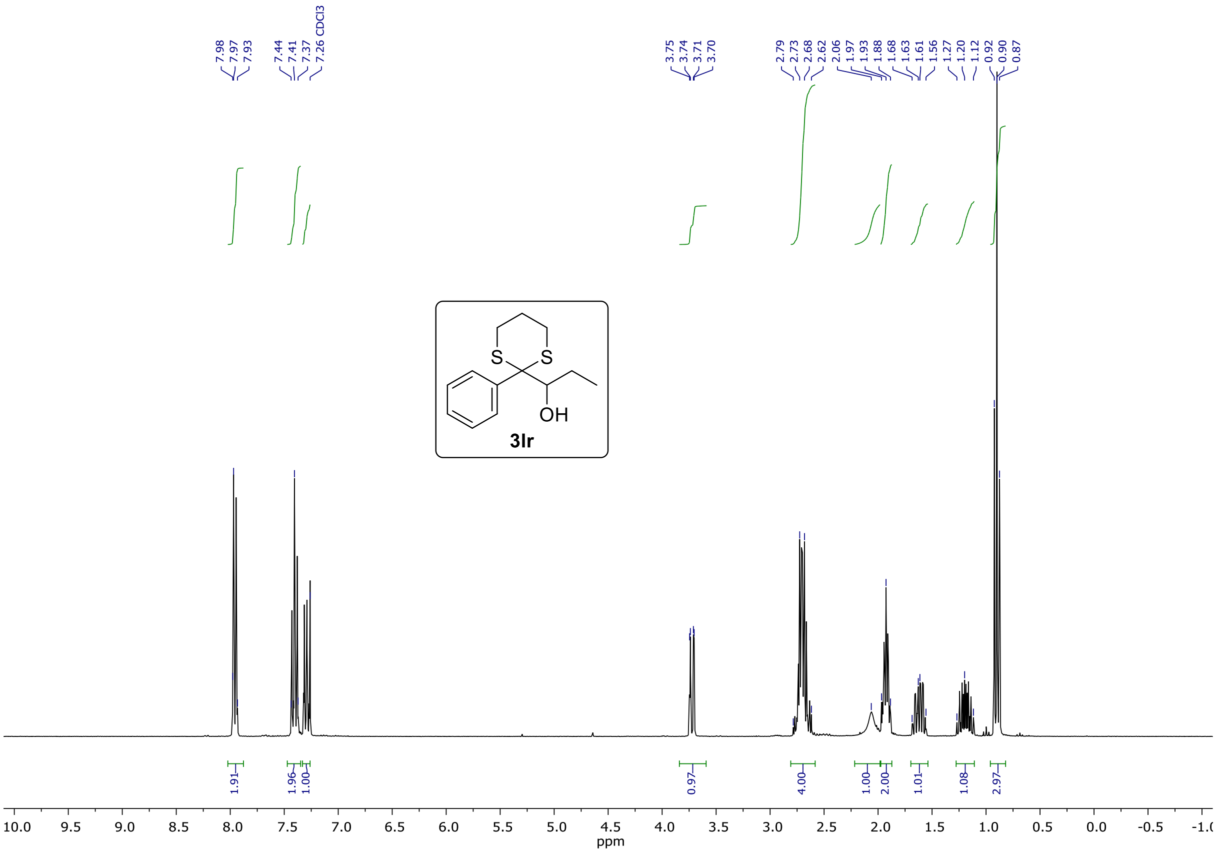

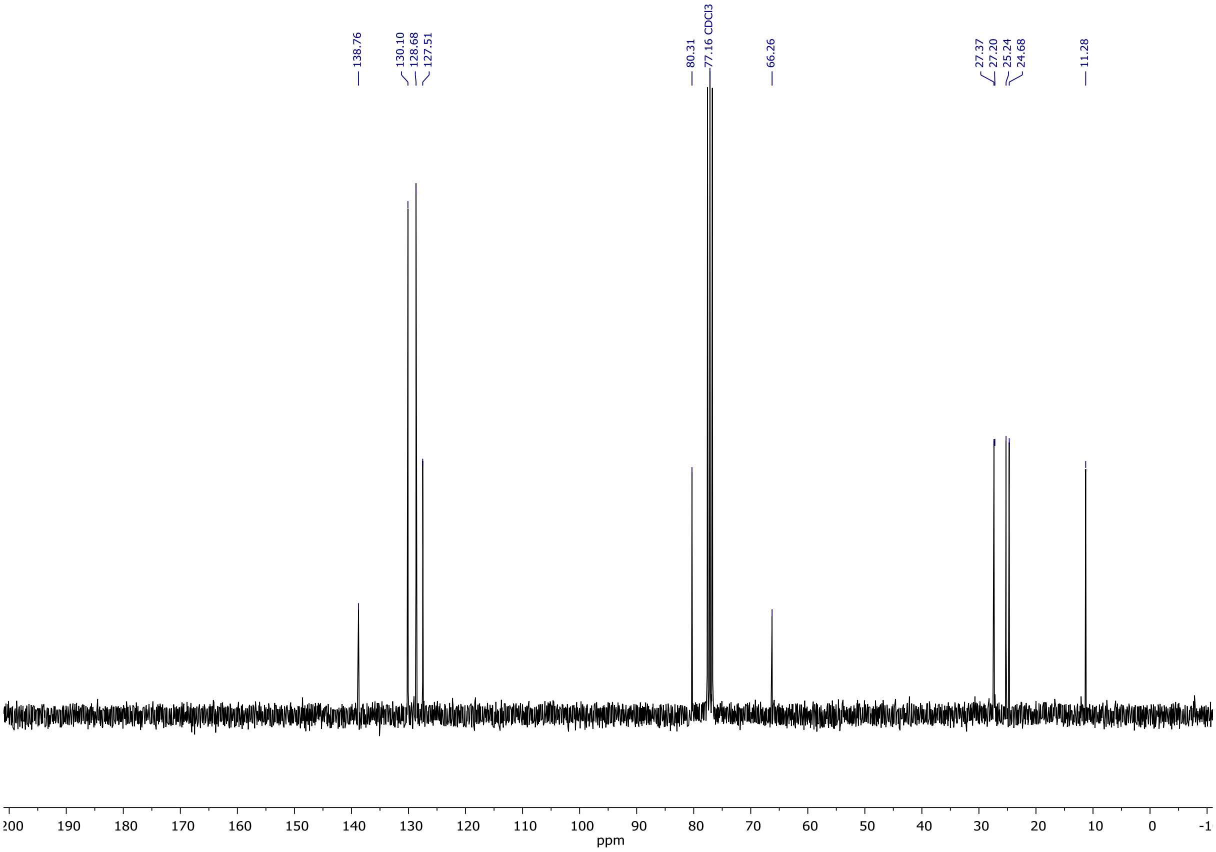

Compound **3mr**, ^{1}H-NMR and ^{13}C-NMR ($CDCl_3$):

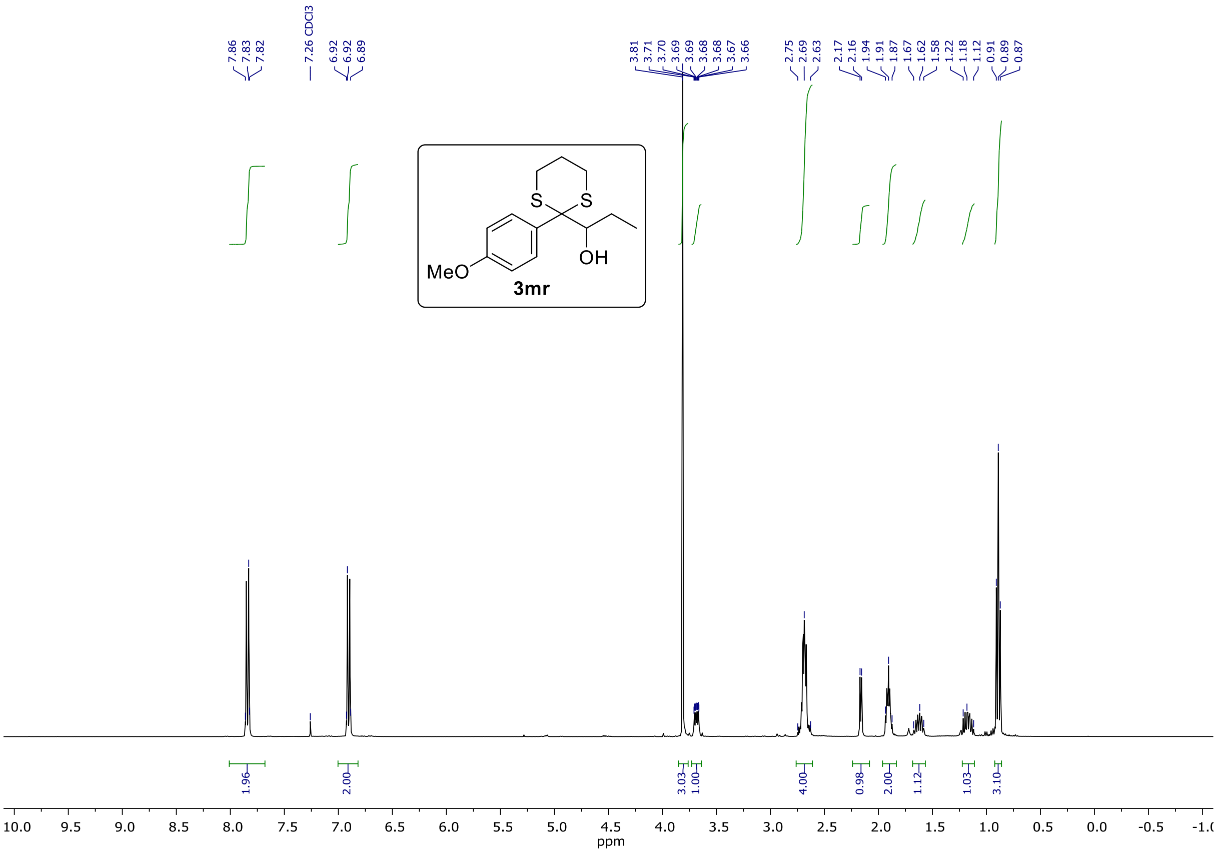

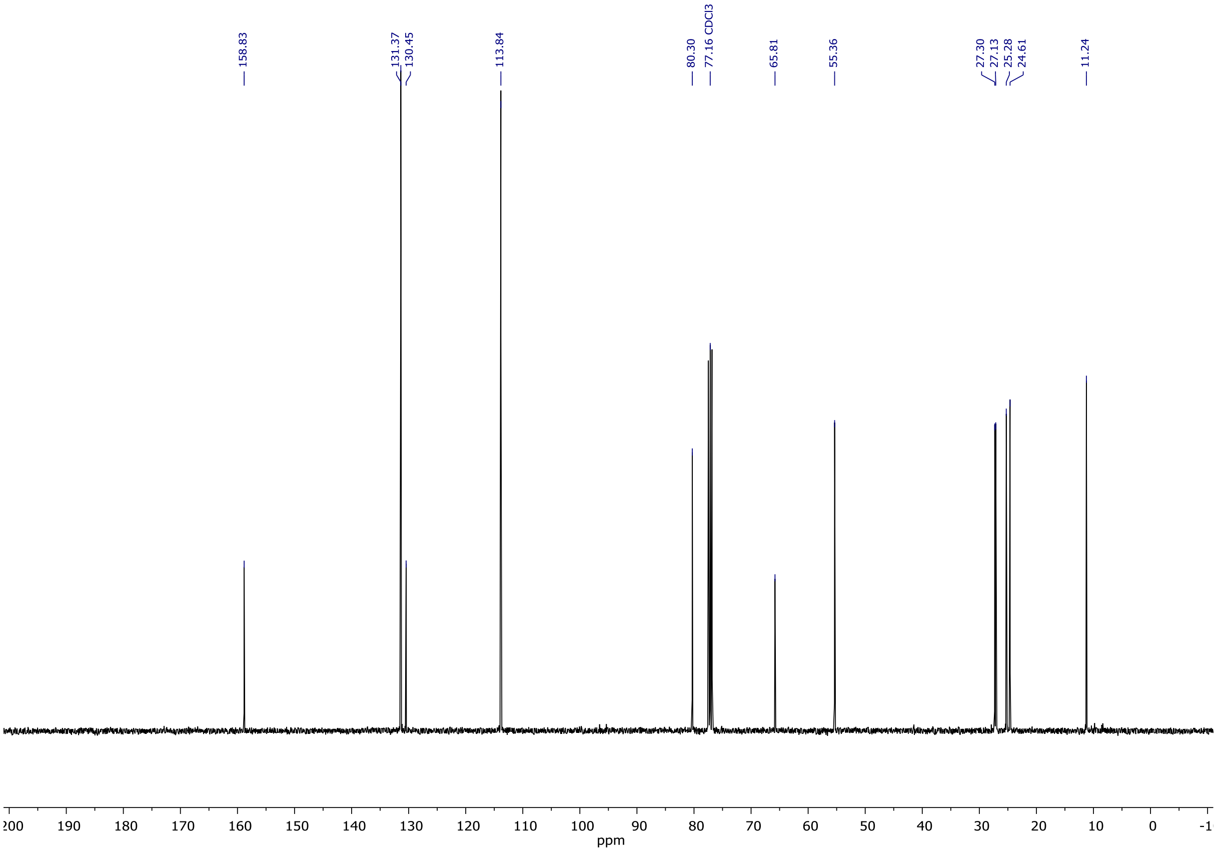

Compound **3or**, ^{1}H-NMR, ^{13}C-NMR, 135-DEPT and HMBC ($CDCl_3$):

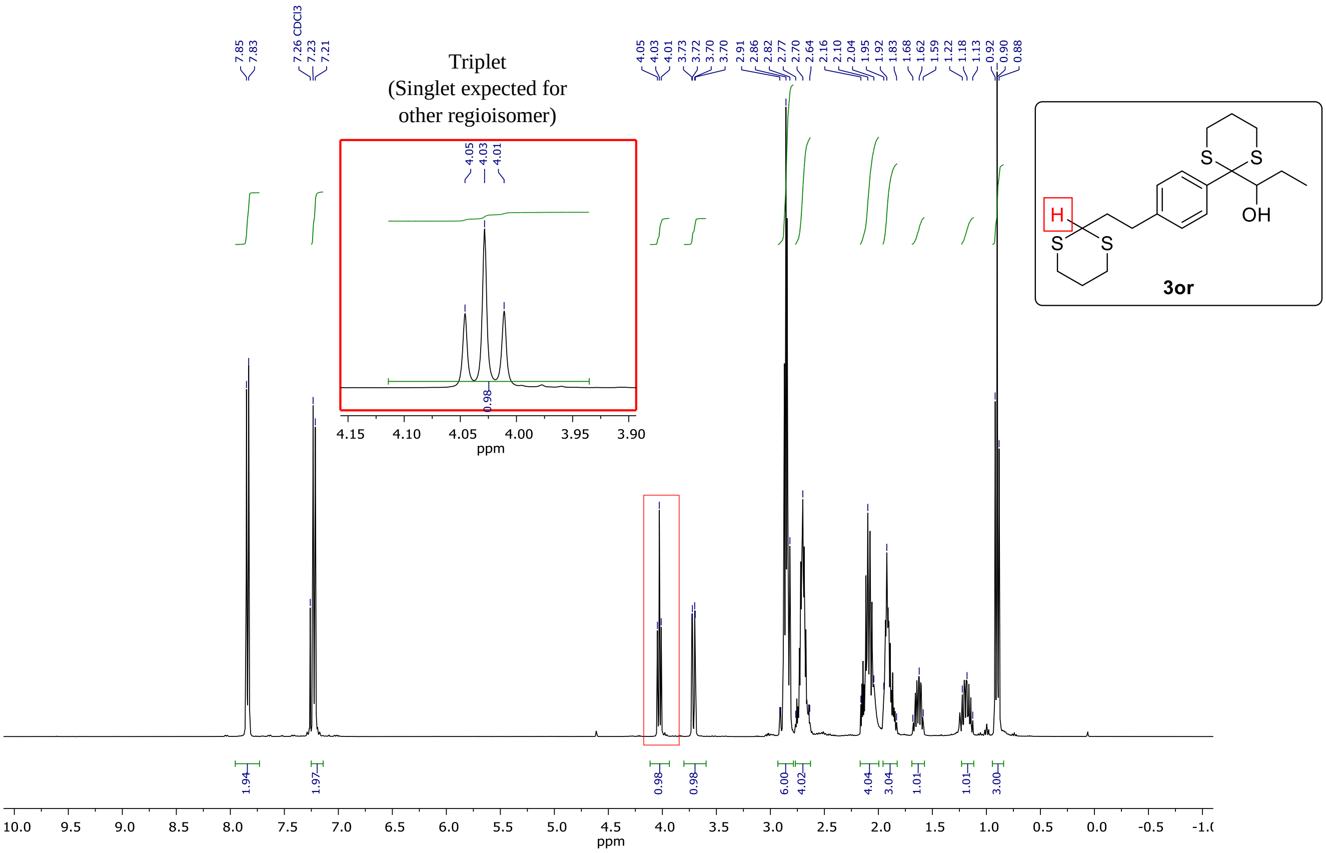

3or

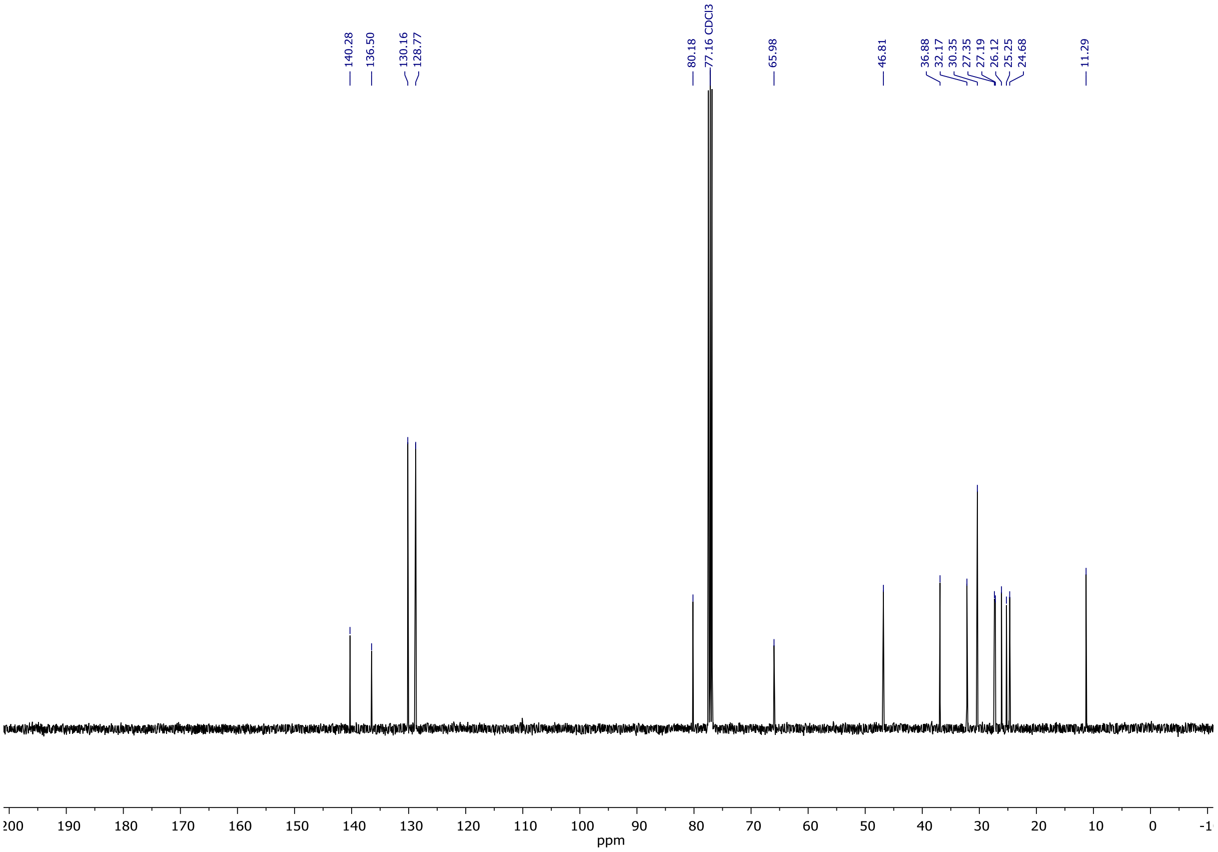

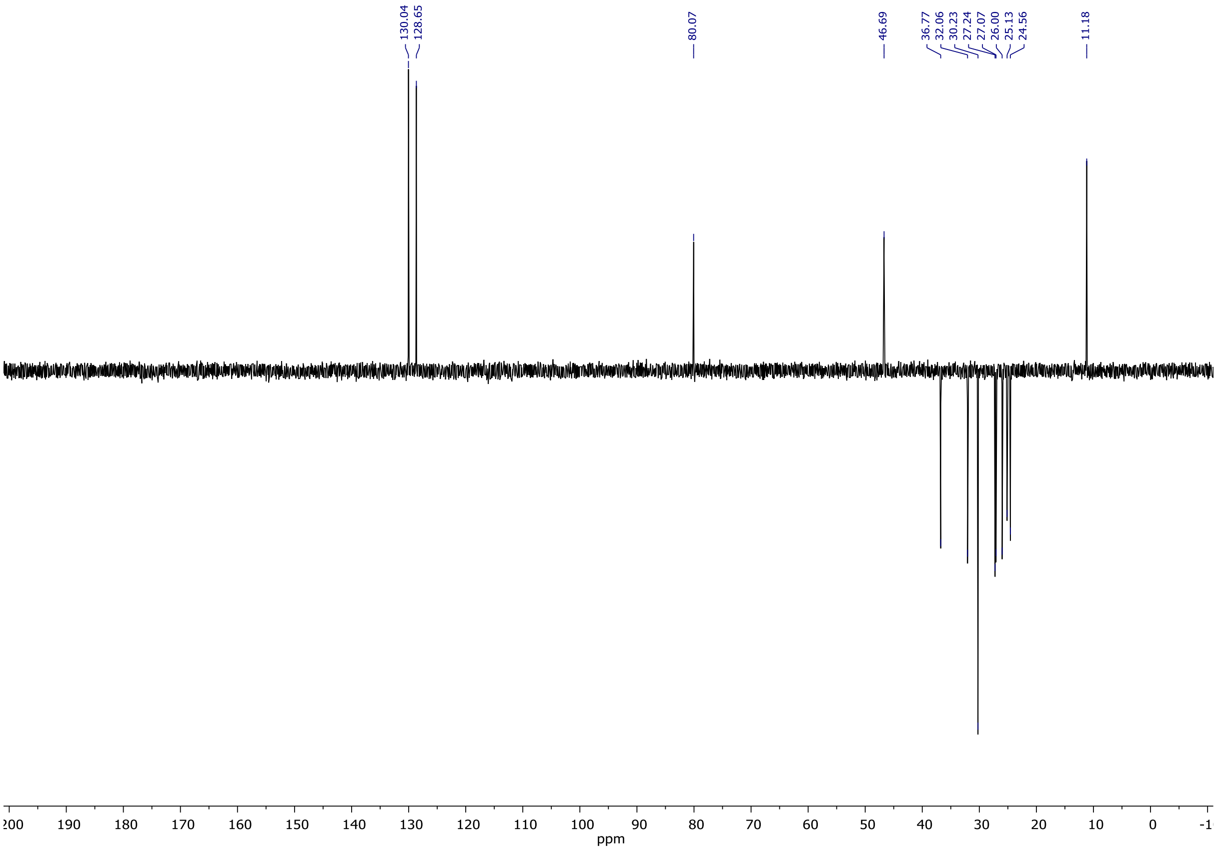
130.04
128.65
80.07
46.69
36.77
32.06
30.23
27.24
27.07
26.00
25.13
24.56
11.18
ppm

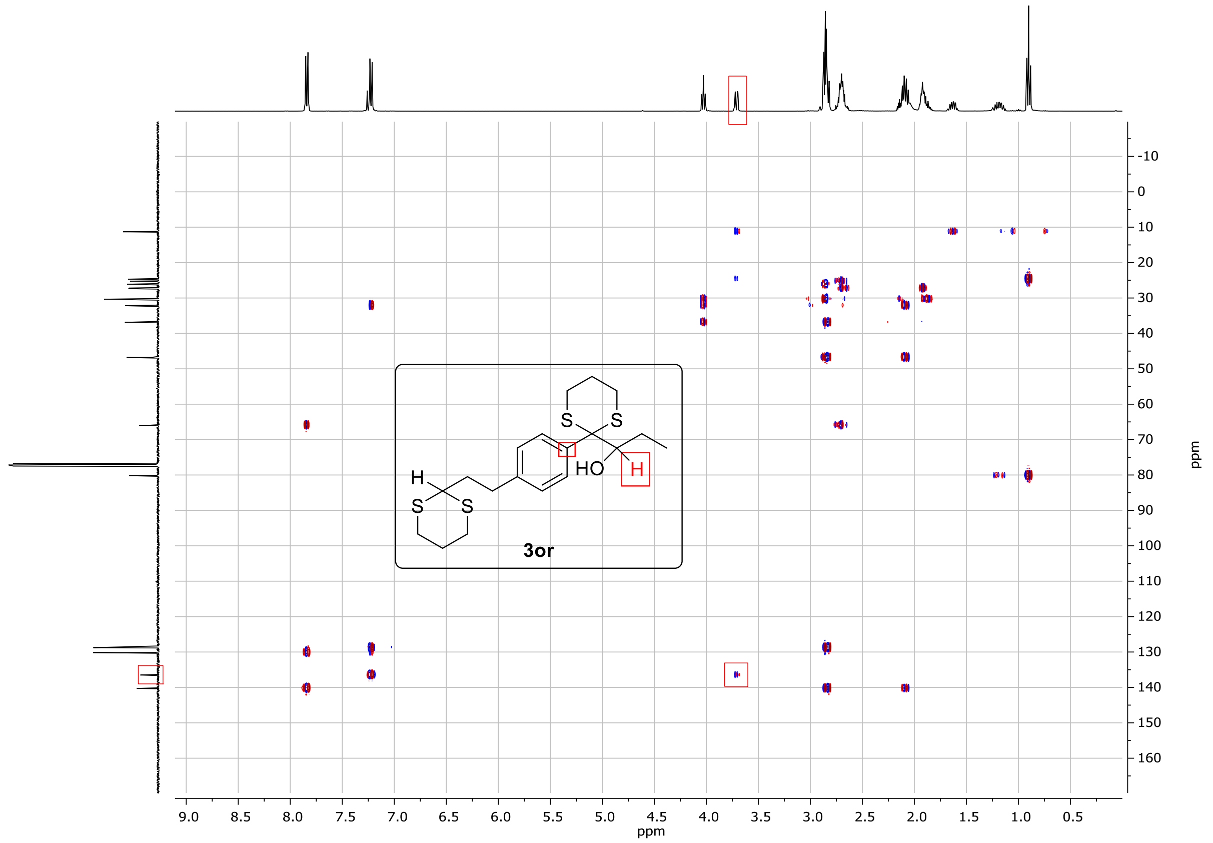
HO
H
3or
ppm
ppm

Compound **4ag**, ^{1}H-NMR and ^{13}C-NMR ($CDCl_3$):

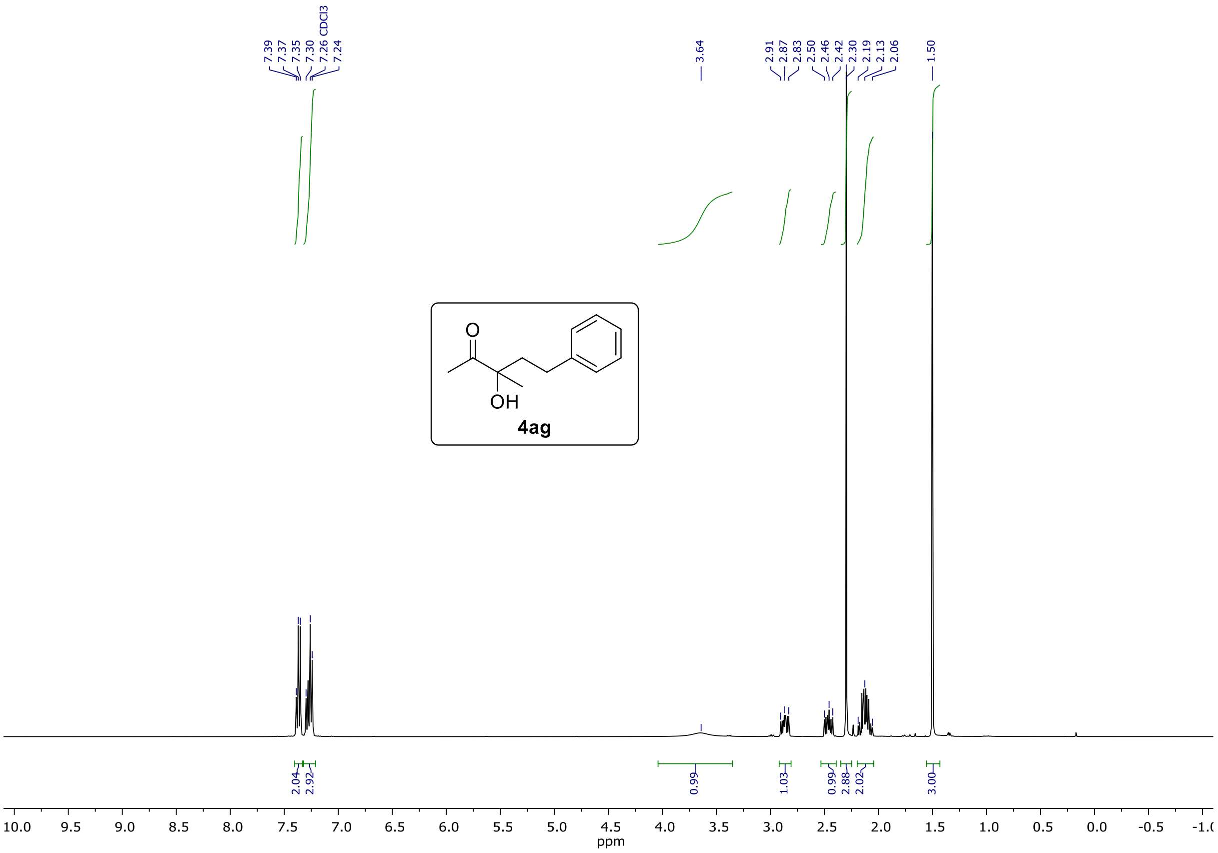

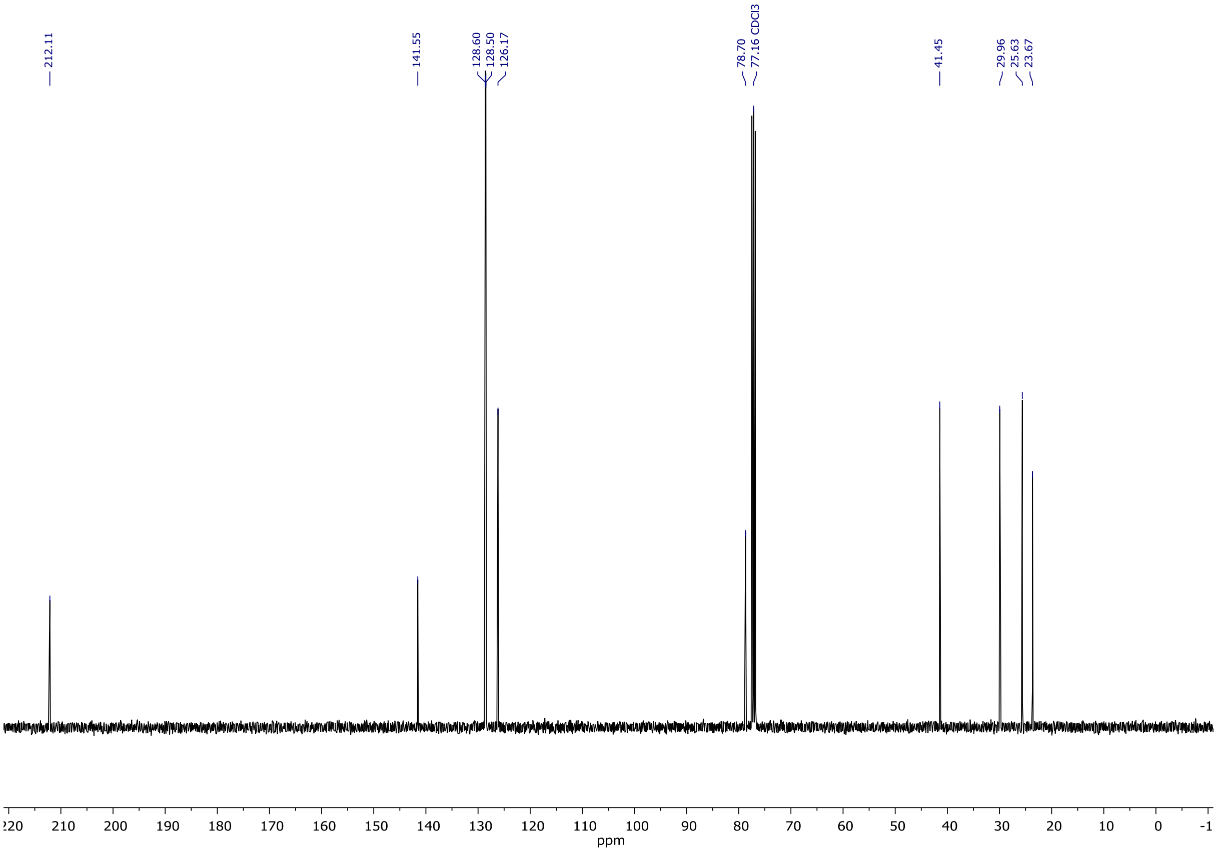

Compound **6**, ^{1}H-NMR and ^{13}C-NMR ($CDCl_3$):

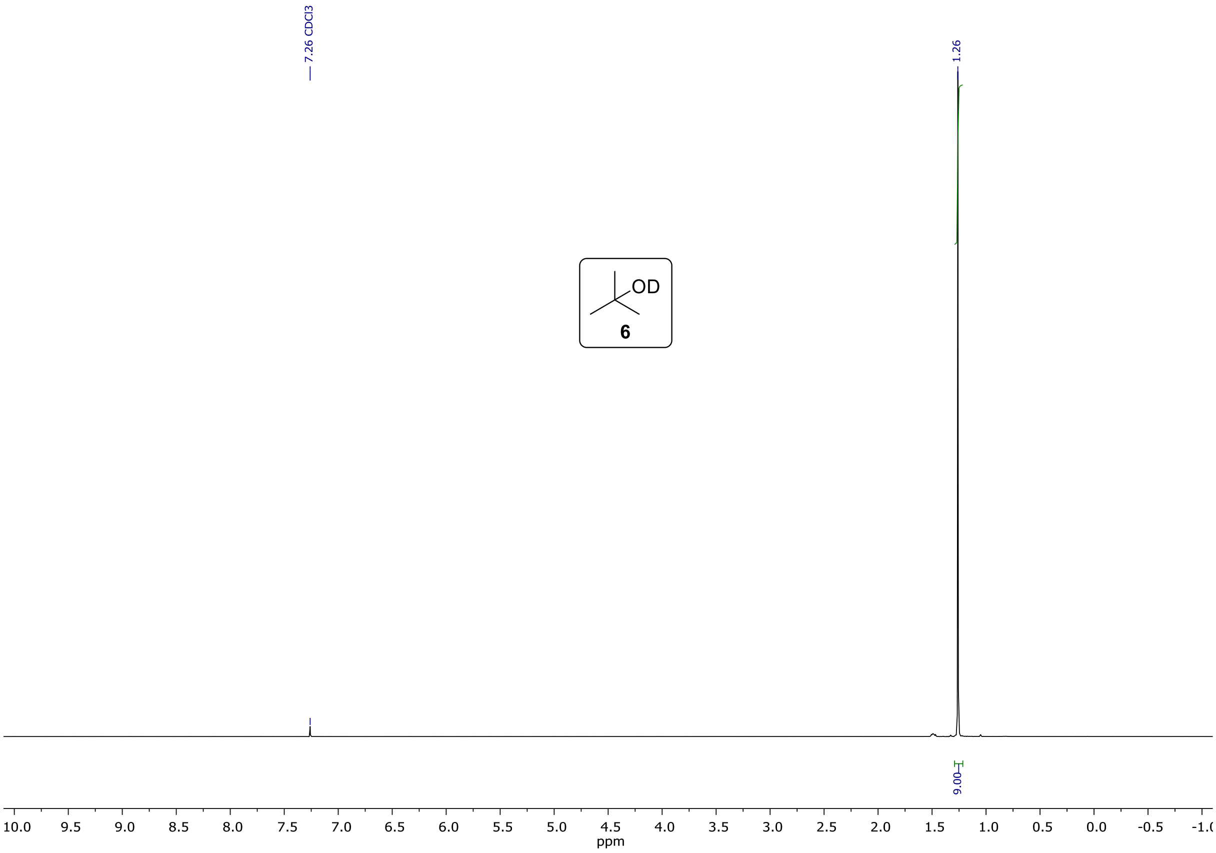

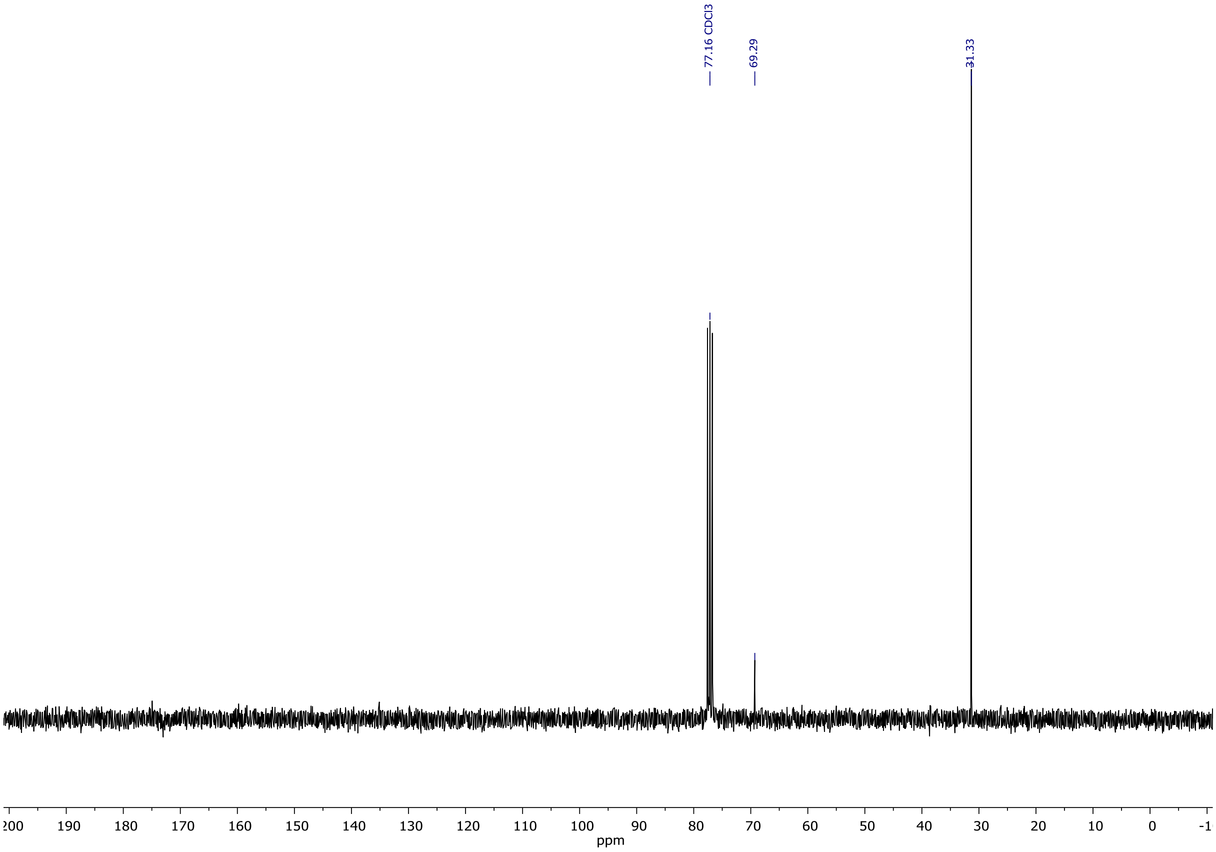

10. References

[1] R. K. Harris, E. D. Becker, S. M. Cabral de Menezes, R. Goodfellow, P. Granger, *Magn. Reson. Chem.* **2002**, *40*, 489-505.

[2] G. R. Fulmer, A. J. M. Miller, N. H. Sherden, H. E. Gottlieb, A. Nudelman, B. M. Stoltz, J. E. Bercaw, K. I. Goldberg, *Organometallics* **2010**, *29*, 2176-2179.

[3] E. Speckmeier, T. G. Fischer, K. Zeitler, *J. Am. Chem. Soc.* **2018**, *140*, 15353-15365.

[4] M. S. Lowry, J. I. Glodsmith, J. D. Slinker, R. Rohl, R. A. Pascal, G. G. Malliaras, S. Bernhard, *Chem. Mater.* **2005**, *17*, 5712-5719.

[5] S. A. Moteki, A. Usui, S. Selvakumar, T. Zhang, K. Maruoka, *Angew. Chem. Int. Ed.* **2014**, *53*, 11060-11064.

[6] Z. Xing, M. Yang, H. Sun, Z. Wang, P. Chen, L. Liu, X. Wang, X. Xie, X. She, *Green Chem.* **2018**, *20*, 5117-5122.

[7] Y. Nagao, K. Seno, E. Fujita, *Tetrahedron Lett.* **1979**, *20*, 3167-3168.

[8] Y. Li, K. Miyazawa, T. Koike, M. Akita, *Org. Chem. Front.* **2015**, *2*, 319-323.

[9] Y. Zhou, A. K. Gupta, M. Mukherjee, L. Zheng, W. D. Wulff, *J. Org. Chem.* **2017**, *82*, 13121-13140.

[10] Y.-S. Hon, T.-R. Sheu, C.-F. Lee, *Synth. Commun.* **2000**, *30*, 97-118.

[11] Y. Gan, W. Xu, Y. Liu, *Org. Lett.* **2019**, *21*, 9652-9657.

[12] Y. Shang, X. Jie, K. Jonnada, S. N. Zafar, W. Su, *Nat. Commun.* **2017**, *8*, 2273.

[13] R. Huang, X. Chen, C. Mou, G. Luo, Y. Li, X. Li, W. Xue, Z. Jin, Y. R. Chi, *Org. Lett.* **2019**, *21*, 4340-4344.

[14] Y. Zhang, K. T. Zhao, S. G. Fox, J. Kim, D. R. Kirsch, R. J. Ferrante, R. I. Morimoto, R. B. Silverman, *J. Med. Chem.* **2015**, *58*, 5942-5949.

[15] C. Liu, Y. Shen, Z. Xiao, H. Yang, X. Han, K. Yuan, Y. Ding, *Green Chem.* **2019**, *21*, 4030-4034.

[16] A. Samanta, B. J. Ravoo, *Chem. Eur. J.* **2014**, *20*, 4966-4973.

[17] D. Crich, H. Xu, F. Kenig, *J. Org. Chem.* **2006**, *71*, 5016-5019.

[18] R. Shinohara, M. Morita, N. Ogawa, Y. Kobayashi, *Org. Lett.* **2019**, *21*, 3247-3251.

[19] Y. Xu, X. Qi, P. Zheng, C. C. Berti, P. Liu, G. Dong, *Nature* **2019**, *567*, 373-378.

[20] A. You, J. Zhou, S. Song, G. Zhu, H. Song, W. Yi, *Bioorg. Med. Chem.* **2015**, *23*, 924-931.

[21] A. Nickon, A. D. Rodriguez, V. Shirhatti, R. Ganguly, *J. Org. Chem.* **1985**, *50*, 4218-4226.

[22] S. Shahsavari, C. McNamara, M. Sylvester, E. Bromley, S. Joslin, B. Y. Lu, S. Fang, *Beilstein J. Org. Chem.* **2018**, *14*, 1750-1757.

[23] N. Komatsu, A. Taniguchi, S. Wada, H. Suzuki, *Adv. Synth. Catal.* **2001**, *343*, 473-480.

[24] G. A. Kraus, B. Roth, *J. Org. Chem.* **1978**, *43*, 2072-2073.

[25] O. Hartmann, M. Kalesse, *Org. Lett.* **2012**, *14*, 3064-3067.

[26] T. Sun, G. Hou, M. Ma, X. Zhang, *Adv. Synth. Catal.* **2011**, *353*, 253-256.

[27] M. J. Frisch, G. W. Trucks, H. B. Schlegel, G. E. Scuseria, M. A. Robb, J. R. Cheeseman, G. Scalmani, V. Barone, G. A. Petersson, H. Nakatsuji, X. Li, M. Caricato, J. B. A. V. Marenich, B. G. Janesko, R. Gomperts, B. Mennucci, H. P. Hratchian, J. V. Ortiz, A. F. Izmaylov, J. L. Sonnenberg, D. Williams-Young, , F. L. F. Ding, F. Egidi, J. Goings, B. Peng, A. Petrone, T. Henderson, D. Ranasinghe, V. G. Zakrzewski, J. Gao, N. Rega, G. Zheng, W. Liang, M. Hada, M. Ehara, K. Toyota, R. Fukuda, J. Hasegawa, M. Ishida, T. Nakajima, Y. Honda, O. Kitao, H. Nakai, , K. T. T. Vreven, J. A. Montgomery, Jr., J. E. Peralta, F. Ogliaro, M. J. Bearpark, J. J. Heyd, E. N. Brothers, K. N. Kudin, V. N. Staroverov, T. A. Keith, R. Kobayashi, J. Normand, K. Raghavachari, A. P. Rendell, J. C. Burant, S. S. Iyengar, J. Tomasi, M. Cossi, J. M. Millam, M. Klene, C. Adamo, R. Cammi, , R. L. M. J. W. Ochterski, K. Morokuma, O. Farkas, J. B. Foresman, and D. J. Fox, *Gaussian, Inc., Wallingford CT* **2016**.

[28] P. C. St John, Y. Guan, Y. Kim, S. Kim, R. S. Paton, *Nat. Commun.* **2020**, *11*, 2328.

[29] A. V. Marenich, J. Ho, M. L. Coote, C. J. Cramer, D. G. Truhlar, *Phys. Chem. Chem. Phys.* **2014**, *16*, 15068-15106.
